# A general synthesis of nitriles from nitroalkanes with bis(catecholato)diboron

**DOI:** 10.1039/d6sc04517k

**Published:** 2026-06-29

**Authors:** Xiaojie Liu, Biping Xu, Martin Oestreich

**Affiliations:** a Institut für Chemie, Technische Universität Berlin Strasse des 17. Juni 115 10623 Berlin Germany martin.oestreich@tu-berlin.de

## Abstract

A metal-free method for accessing nitriles from nitroalkanes is reported. The reaction is mediated by 1,8-diazabicyclo[5.4.0]undec-7-ene with bis(catecholato)diboron as the deoxygenation reagent. Both boron units are engaged, therefore only requiring slightly more than stoichiometric amounts of the diboron reagent. A broad range of nitroalkanes can be converted into the corresponding nitriles, including structurally complex molecules. No erosion of enantiopurity is seen with a β-chiral nitroalkane to arrive at an α-chiral nitrile. The value of the new method is demonstrated by a short synthesis of 2-arylpropionic acids (profens) to potentially replace the Boots–Hoechst–Celanese route.

## Abstract 2

A combination of bis(catecholato)diboron and 1,8-diazabicyclo[5.4.0]undec-7-ene (DBU) mediates the dehydration of nitroalkanes into the corresponding nitriles, thereby allowing for a short synthesis of 2-arylpropionic acids (profens).

## Graphical abstract

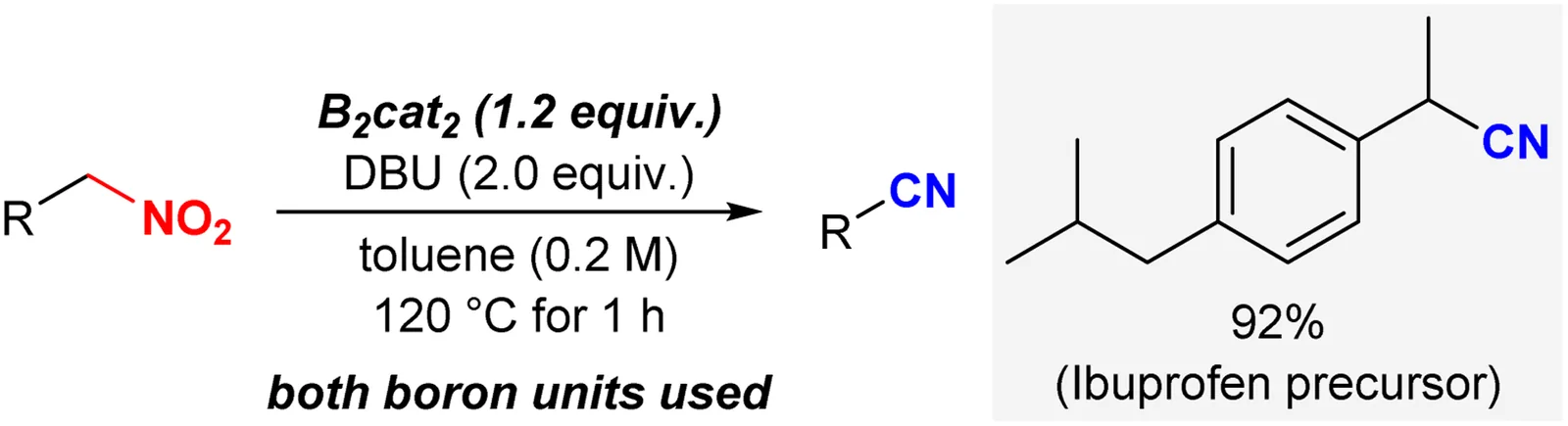

## Introduction

Nitriles represent an extraordinarily versatile functional motif in organic synthesis, acting as flexible precursors to a diverse array of functional groups, including carbonyl compounds of any type, alcohols, and amines as well as nitrogen-containing heterocycles.^1^ Furthermore, the cyano group is frequently part of bioactive pharmaceuticals.^2^ Consequently, their prevalence across organic and medicinal chemistry has driven continuous research interest in developing straightforward and efficient strategies for the construction of nitrile building blocks.

**Scheme 1:**
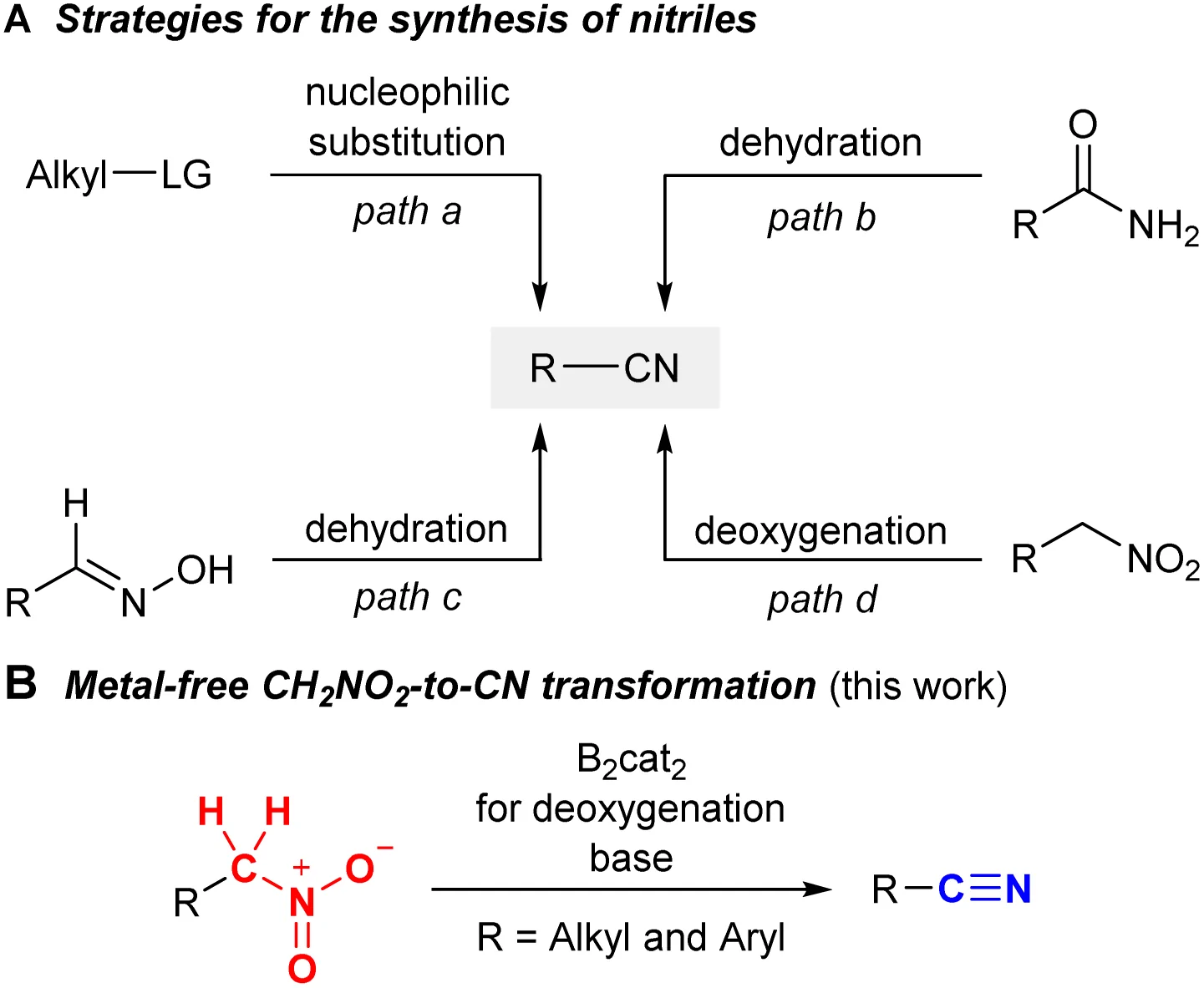
Synthesis of nitriles; LG = leaving group.

Conventional strategies to synthesize alkyl nitriles typically rely on nucleophilic substitution of alkyl (pseudo)halides with cyanide sources, often involving highly toxic reagents and being plagued with competing β-elimination (Scheme 1A, path a).^3^ As an alternative to address these issues, amides^4^ and oximes^5^ represent other classes of qualified nitrile precursors and react in the presence of acidic dehydrating agents (Scheme 1A, paths b and c). Despite substantial advances in dehydrative nitrile synthesis with transition-metal catalysts, these approaches remain intrinsically dependent on preinstalled carbonyl functionality and often require additional activation steps. Consequently, the challenges associated with these protocols highlight the need for orthogonal approaches that can deliver nitriles without relying on toxic cyanide reagents or harsh dehydrating conditions from more readily accessible starting materials.

Nitroalkanes represent attractive, easily available precursors in this context (Scheme 1A, path d).^6^ However, their conversion into nitriles necessitates merging deoxygenation and dehydration. Trivalent phosphine reagents have been systematically explored for this purpose.^7^ Similarly, low-valent sulfur reagents are also competent to promote the desired transformation.^8^ Recently, a novel and elegant nitro-to-nitrile conversion has been achieved through mild visible-light catalysis by using trialkylamines as the reductant.^9^ Nevertheless, boron-based systems, notwithstanding their well-established intrinsic deoxygenative and reductive capabilities,^10^ still remain largely unexploited in this field. We hypothesized that a suitably activated diboron reagent could simultaneously engage in deoxygenation and dehydration, thereby enabling a direct nitro-to-nitrile conversion (Scheme 1B).

## Results and discussion

Following this idea, we initiated our optimization using 3-nitropropylbenzene (1a) as the model substrate and bis(catecholato)diboron as the deoxygenation reagent (Table 1). After systematic optimization of the reaction parameters (see the SI for details), 3-phenylpropionitrile (2a) was obtained in near-quantitative yield in the presence of DBU (entry 1). Intriguingly, other commonly used diboron reagents, including B_2_pin_2_, B_2_nep_2_ and B_2_(OH)_4_, exhibited no reactivity under otherwise identical conditions, highlighting the need for more Lewis acidic boron centers as is the case with B_2_cat_2_ (entries 1–4).^11^ Only 8% yield is obtained when catecholborane is used instead of B_2_cat_2_ (entry 5). Replacing DBU with triethylamine led to a significant decrease in yield (24%), while inorganic bases such as K_2_CO_3_ and K_3_PO_4_ completely suppressed product formation (entries 6–8). These observations suggest that the combination of favorable basicity and solubility is the key factor that enables DBU to outcompete other bases in this transformation. The yield collapsed to 22% with half of the amount of DBU (entry 9), and no reaction occurred in the absence of a base (entry 10). Solvent effects were also pronounced: aromatic solvents outperformed polar Lewis basic solvents such as ethers and amides (entries 11–14). Lowering the reaction temperature afforded only 6% of the desired product with 37% starting material recovered (entry 15), proving the necessity of elevated temperature to facilitate the reaction conversion. Notably, the reaction proceeded efficiently even in ambient air with a slightly lower yield (entry 16).

**Table 1:** Selected examples of the optimization^a^

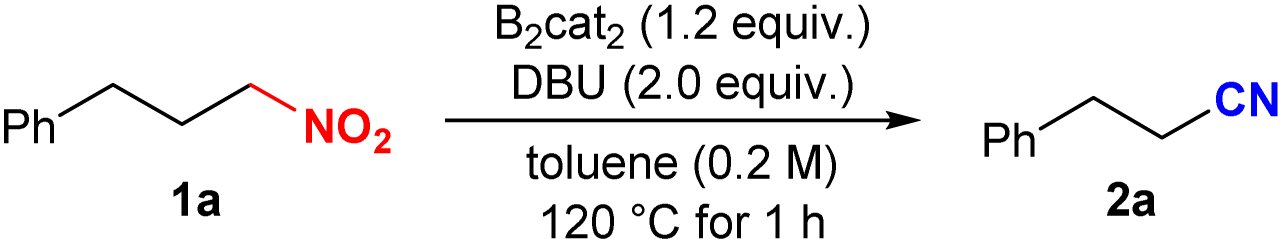		
Entry	Variation from the standard conditions	Yield of 2a (%)^b^
1	None	99 (93)^c^
2	B_2_pin_2_ instead of B_2_cat_2_	ND
3	B_2_nep_2_ instead of B_2_cat_2_	ND
4	B_2_(OH)_4_ instead of B_2_cat_2_	ND
5	Catecholborane instead of B_2_cat_2_	8
6	Et_3_N instead of DBU	24
7	K_2_CO_3_ instead of DBU	ND
8	K_3_PO_4_ instead of DBU	ND
9	1.0 equiv. of DBU	22
10	Without DBU	ND
11	Benzene instead of toluene	48
12	PhCF_3_ instead of toluene	97
13	THF or 1,4-dioxane instead of toluene	ND
14	MeCN or DMF instead of toluene	ND
15	60 °C instead of 120 °C	6
16	Air atmosphere instead of N_2_	85

a Reaction conditions: 3-nitropropylbenzene (1a; 0.20 mmol) and the indicated solvent (1.0 mL) were mixed, DBU (2.0 equiv.) and B_2_cat_2_ (1.2 equiv.) were subsequently added, and the reaction was then maintained at 120 °C for 1 h.

b The yield was determined by gas–liquid chromatography (GLC) analysis with methyl benzoate as an internal standard.

c Isolated yield. ND = not detected. DMF = *N*,*N*-dimethylformamide. DBU = 1,8-diazabicyclo[5.4.0]undec-7-ene. B_2_cat_2_ = bis(catecholato)diboron. B_2_pin_2_ = bis(pinacolato)diboron. B_2_nep_2_ = bis(neopentylglycolato)diboron. B_2_(OH)_4_ = tetrahydroxydiboron.

The striking success with the B_2_cat_2_/DBU combination made us consider Ingleson's discovery^12^ that B_2_cat_2_ can isomerize from its “usual” 1,1- to the far less common 1,2-form by interaction with a Lewis base. This often overlooked phenomenon could be operative here but known cases involve polar (Lewis basic) solvents,^12,13^ whereas the present reaction requires an arene solvent. Monitoring the interaction of B_2_cat_2_ and DBU in toluene by NMR spectroscopy was inconclusive, not providing any hint as to whether the 1,1- or the 1,2-isomer is the actual deoxygenation agent.

**Scheme 2:**
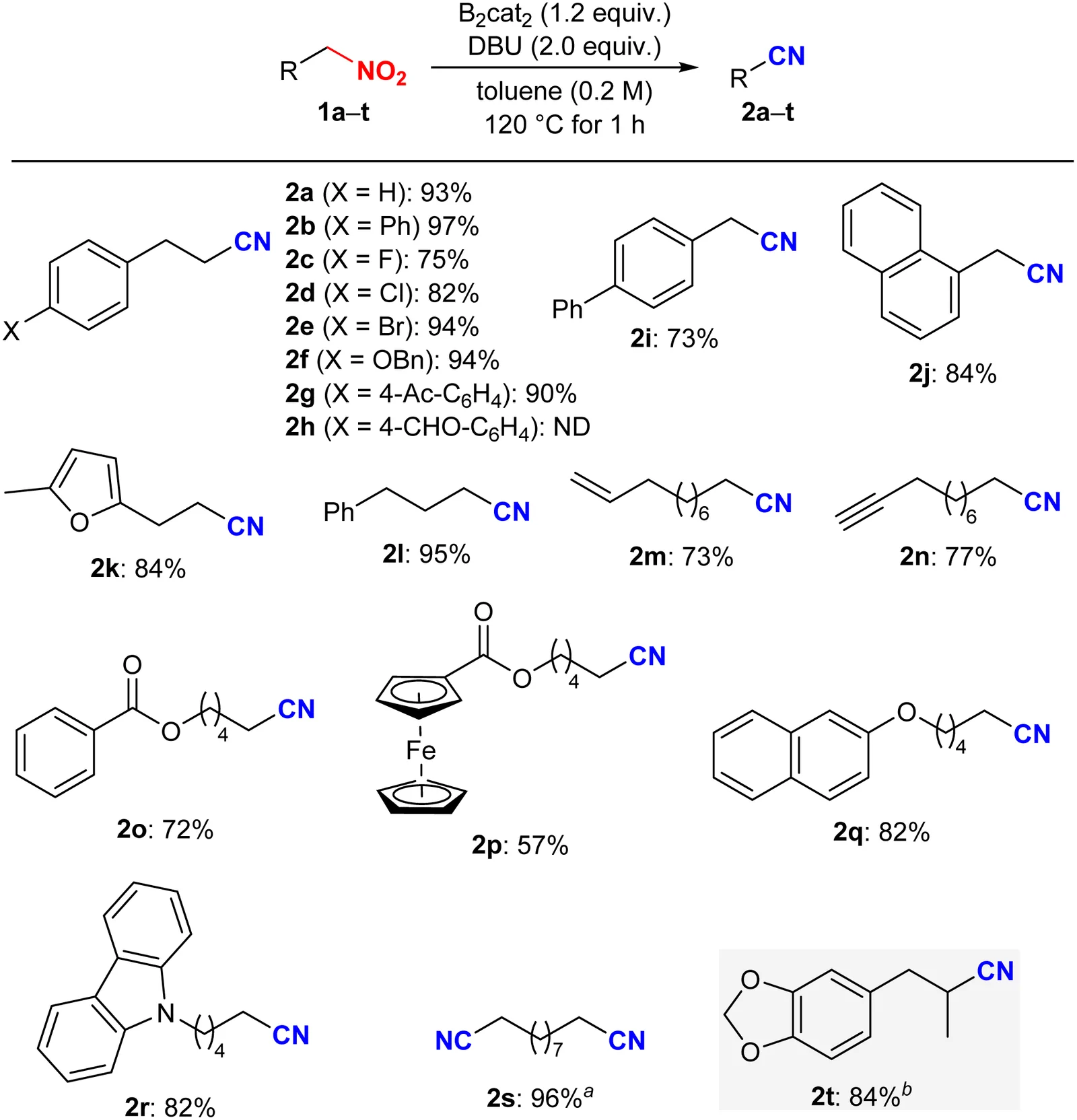
Scope I: synthesis of aliphatic nitriles from nitroalkanes. Reaction conditions: the indicated nitroalkane (0.20 mmol) and toluene (1.0 mL) were mixed, DBU (2.0 equiv.) and B_2_cat_2_ (1.2 equiv.) were subsequently added, and the reaction was then maintained at 120 °C for 1 h. ND = not detected. ^*a*^Using a two-fold excess of reagents. ^*b*^The reaction was conducted at 150 °C for 3 h.

With the optimized conditions established, we next evaluated the generality of this deoxygenative conversion protocol across a broad range of nitroalkanes (Scheme 2). As anticipated, various 3-aryl-substituted nitropropanes 1b–g underwent smooth conversion to the corresponding nitriles 2b–g in good to excellent yields, irrespective of whether the aryl ring was decorated with an aryl (2b), a halogen (2c–e), or benzyloxy (2f), substituent. Of note, a substrate bearing an enolizable ketone as in 1g reacted cleanly but an aldehyde functionality as in 1h resulted in no product formation with full consumption of the starting material. Furthermore, 2-arylnitroethanes 1i and 1j bearing a biphenyl or a naphthyl group were also competent substrates, delivering the desired nitriles 2i and 2j in 73% and 84% yield, respectively. Likewise, heteroaryl-containing substrates were also compatible as exemplified by the efficient formation of furyl-substituted nitrile 2k from 1k. Elongation of the carbon chain was also well tolerated, with 4-phenylnitrobutane 1l affording the corresponding nitrile 2l in 95% yield. Importantly, functional groups such as terminal alkenes (as in 1m) and alkynes (as in 1n), which are sensitive to addition and reduction, remained intact, providing the desired products 2m and 2n in 73% and 77% yield at full conversion with no byproducts detected. The mildness and chemoselectivity of the protocol were further demonstrated by substrates containing oxygenated functionalities, including a benzoate ester (2o), a ferrocenecarboxylate (2p), and a naphthyl ether (2q). In addition, a carbazole as in 1r was also compatible, furnishing the corresponding nitrile product 2r in 82% yield. The protocol further exhibited excellent efficiency in more demanding settings: 1,11-dinitroundecane underwent smooth double deoxygenation to deliver the corresponding bis(nitrile) 2s in 96% yield when a two-fold excess of both B_2_cat_2_ and DBU were employed. Notably, the present strategy could also be extended to the synthesis of secondary alkyl nitriles. To illustrate, a benzodioxole-containing secondary nitrile 2t was successfully obtained when a β-branched nitroalkane 1t was subjected to the reaction conditions at a higher temperature of 150 °C (gray box).

**Scheme 3:**
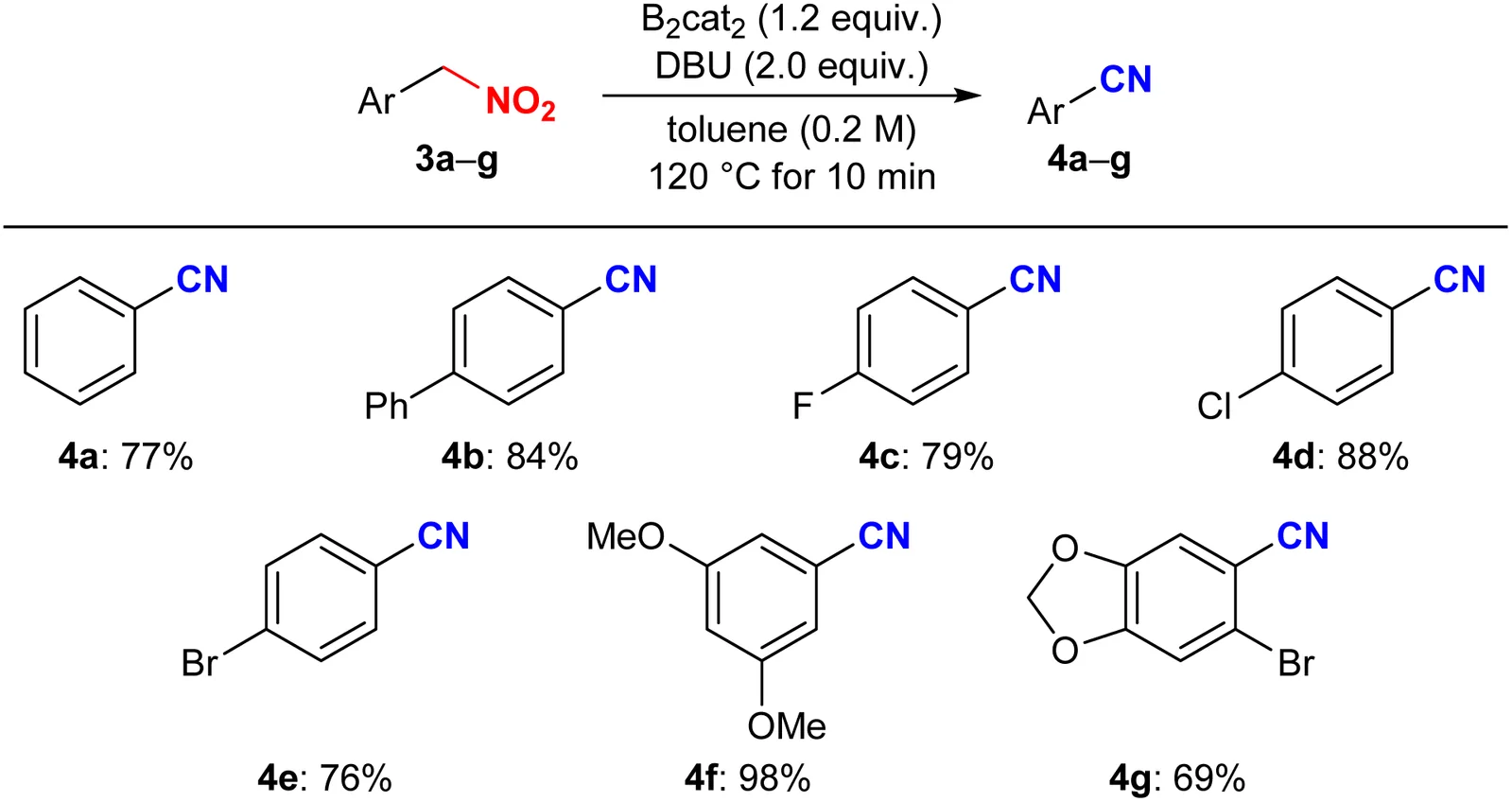
Scope II: synthesis of benzonitriles from benzylic nitro compounds. Reaction conditions: the indicated nitroalkane (0.20 mmol) and toluene (1.0 mL) were mixed, DBU (2.0 equiv.) and B_2_cat_2_ (1.2 equiv.) were subsequently added, and the reaction was then maintained at 120 °C for 10 min.

Benzonitriles are common building blocks in pharmaceuticals, agrochemicals and functional materials.^1*d*^ Building upon the successful deoxygenative conversion of aliphatic nitro compounds into nitriles, we asked ourselves whether benzylic nitro compounds are equally suitable nitrile precursors under the standard conditions. Indeed, substrates 3a–g underwent the desired functional-group interconversion in 10 min (instead of 1 h) to afford aryl nitriles 4a–g in good to excellent yields (Scheme 3).

**Fig. 1:**
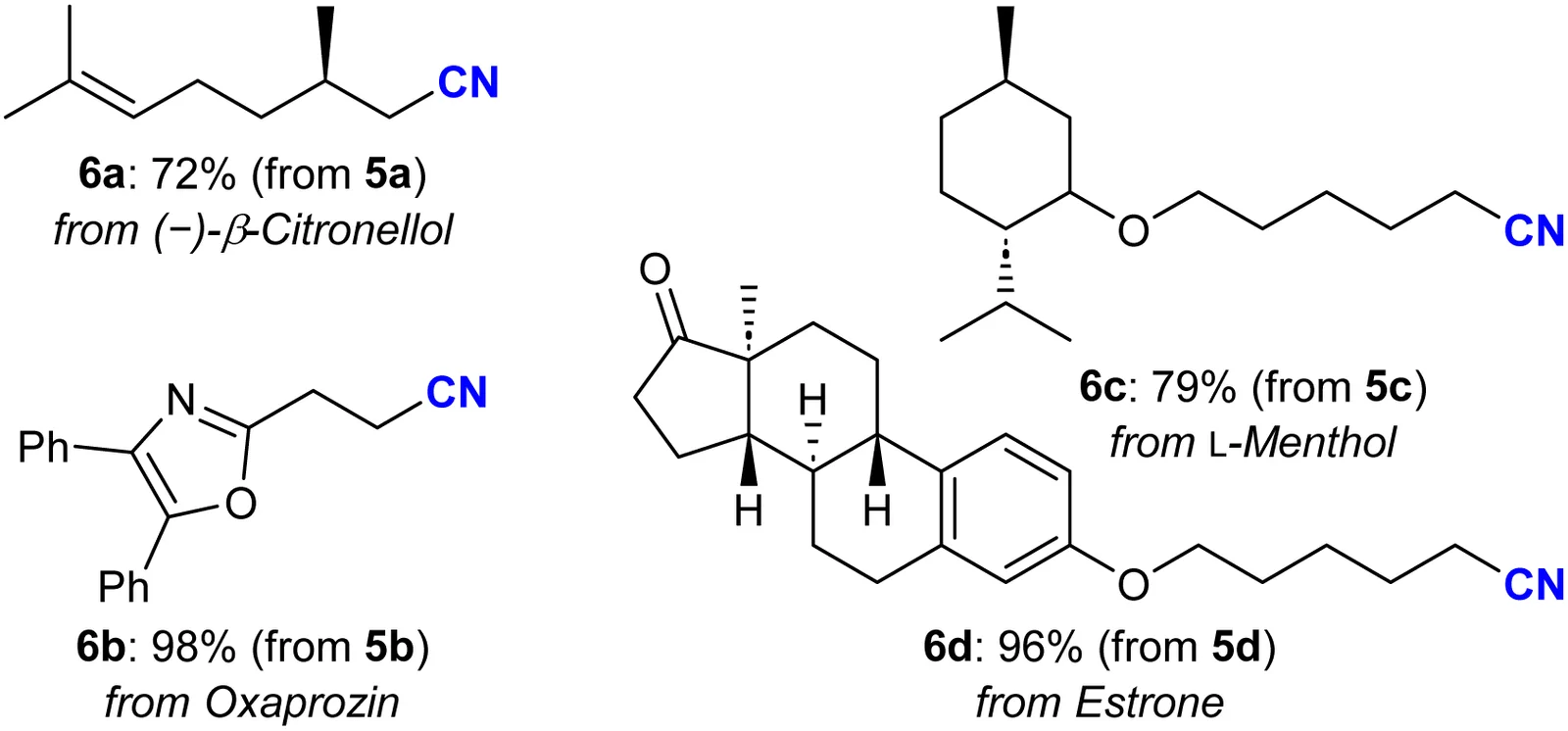
Scope III: transformation of structurally complex nitroalkanes. See Scheme 2 for details.

Encouraged by these results, we further explored structurally complex and biologically relevant substrates (Fig. 1). Gratifyingly, nitro derivatives 5a–d of natural products and pharmaceuticals, including (−)-β-citronellol (5a), oxaprozin (5b), l-menthol (5c), and estrone (5d), were readily converted into their corresponding nitriles 6a–d in good yields.

**Scheme 4:**
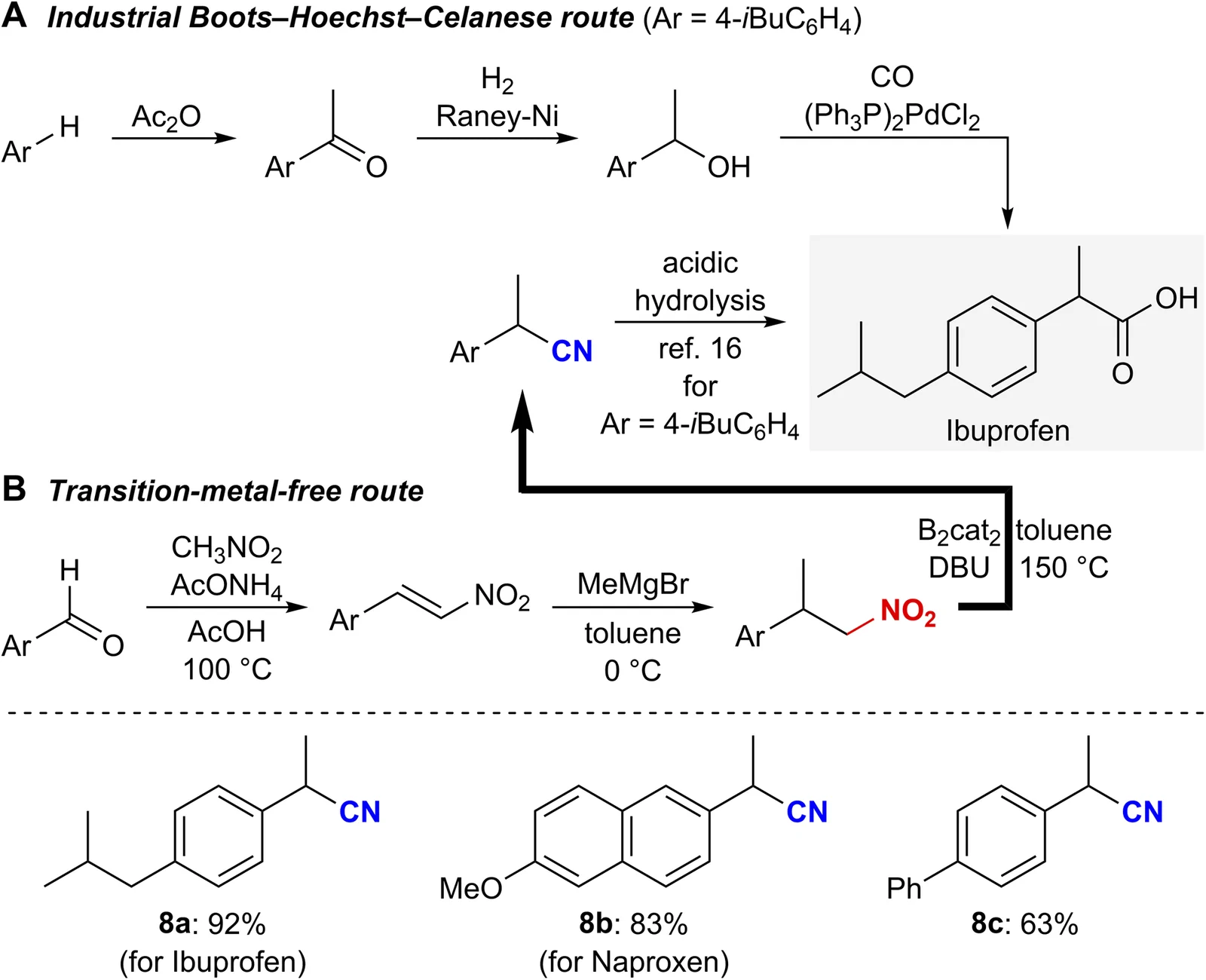
Scope IV: synthesis of 2-arylpropionic acids (profens).

To further showcase the method's value, we integrated the nitro-to-nitrile interconversion into the synthesis of 2-arylpropionic acids (profens). Their privileged structural motifs are widely used in nonsteroidal anti-inflammatory drugs such as ibuprofen (gray box) and naproxen.^14^ Traditionally, these compounds are industrially produced by the Boots–Hoechst–Celanese route, which relies on transition-metal catalysis (Scheme 4A).^15^ In contrast, our protocol enables rapid and efficient access to their corresponding nitriles 8a–c (Scheme 4B); these can be readily hydrolyzed to furnish the target pharmaceuticals.^16^ Notably, the new sequence proceeds entirely without the need for transition metals and is cyanide-free. This could offer a more sustainable and operationally simple alternative to the existing strategy.

**Scheme 5:**
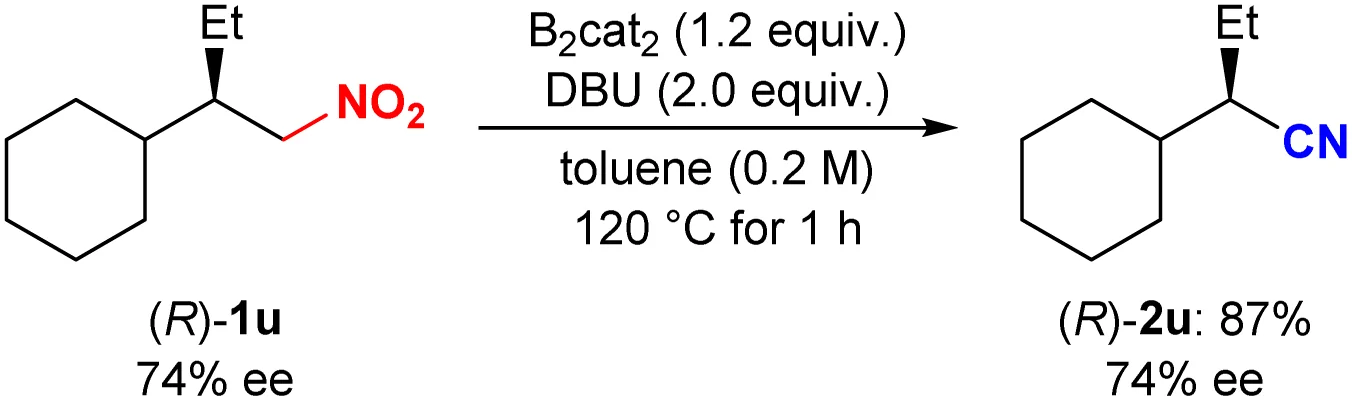
Racemization-free nitro-to-nitrile interconversion.

Beyond practical synthesis, this transformation also overcomes challenges in the preparation of α-chiral nitriles (Scheme 5). Although prevalent in biologically active molecules, direct methods for the enantioselective formation of C(sp^3^)–CN bonds without heteroatom substitution remain limited.^17^ In contrast, β-chiral nitro compounds are readily accessible by well-established asymmetric transformations such as the Henry reaction and Michael addition.^6*a*,*b*^ By using the new protocol, the enantioenriched nitroalkane (*R*)-1u was converted into the corresponding nitrile (*R*)-2u in 87% yield without erosion of the enantioenrichment. This result establishes a practical connection between β-chiral nitro compounds and α-chiral nitriles.

**Scheme 6:**
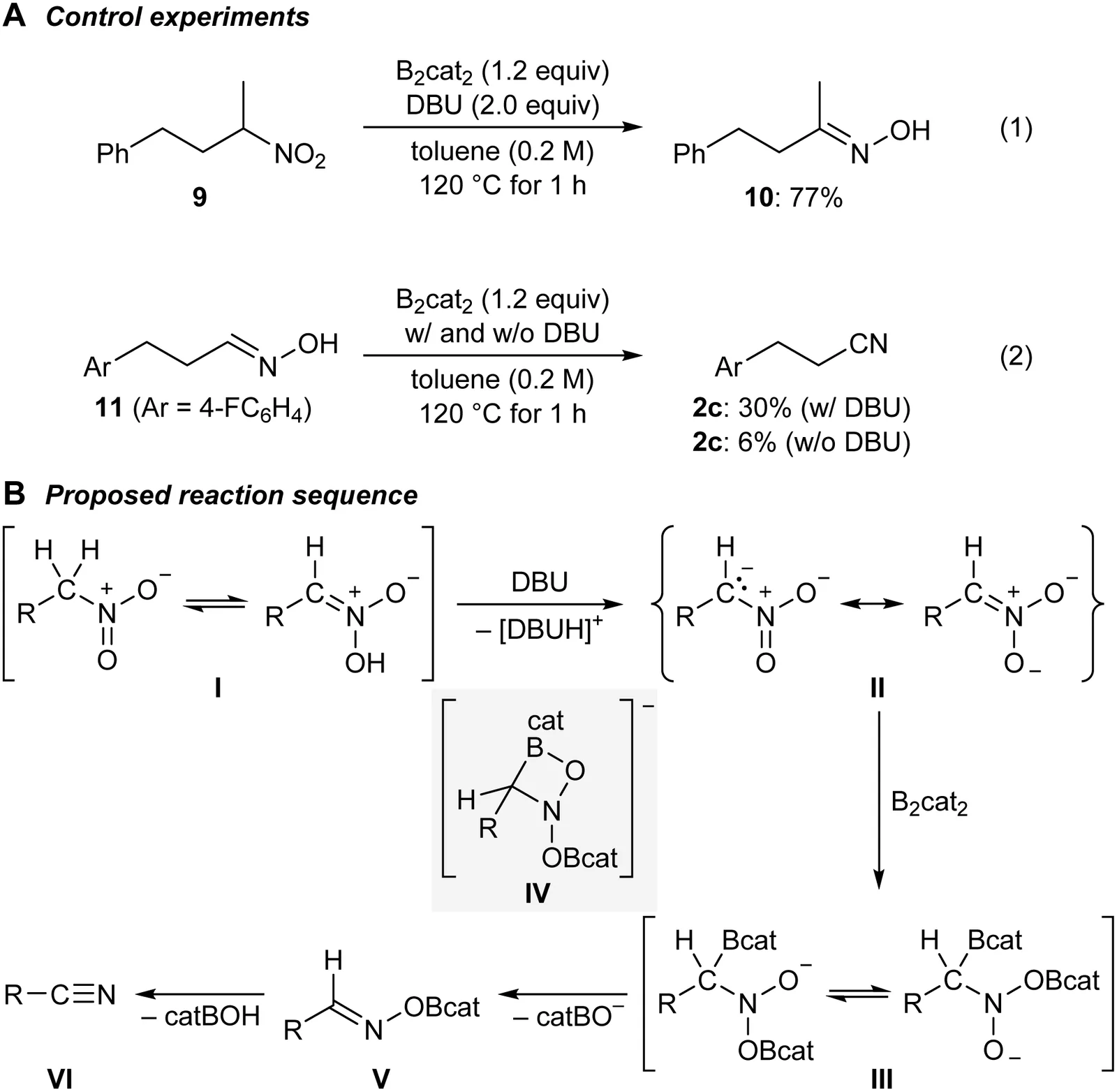
Control experiment and the plausible mechanism.

To probe for intermediates of this reaction, control experiments were performed (Scheme 6A). When secondary nitroalkane 9 was subjected to the standard protocol, the ketoxime 10 was obtained in 77% yield [Scheme 6A, eqn (1)], suggesting that the transformation of primary nitroalkanes to nitriles proceeds through the intermediacy of the corresponding aldoximes. We then subjected aldoxime 11 to the standard setup in the presence and absence of the base, respectively [Scheme 6A, eqn (2)]. With DBU, the nitrile 2c was obtained in 30% yield with 47% of 11 recovered. Conversely, the reaction starting from aldoxime 11 without DBU furnished only 6% yield of nitrile 2c with the rest of the material being decomposed. Collectively, these experiments support aldoxime formation followed by base-mediated elimination. On this basis, along with precedent in boron-mediated transformations,^18^ a plausible mechanism is proposed for the assumed 1,1-isomer of B_2_cat_2_ (ref. 12 and 13) (Scheme 6B). The nitroalkane and nitronic acid tautomer (*aci* form) I is deprotonated by DBU to generate the nitronate intermediate II. The diboron reagent B_2_cat_2_ subsequently adds across II to form adduct III with both boron moieties incorporated. We suggest that intermediate III, featuring a synperiplanar arrangement of the C–B and N–O bonds, is likely to form the four-membered cyclic ate complex IV (gray box).^19^ Its formal cycloreversion results in β-elimination to afford the *O*-borylated aldoxime V. As demonstrated above, V undergoes another β-elimination to arrive at the nitrile product VI.

## Conclusions

In summary, we reported a simple and efficient method for the direct conversion of nitroalkanes into nitriles. This transformation proceeds smoothly under metal- and cyanide-free conditions, employing only commercially available, bench-stable reagents (DBU and B_2_cat_2_). The method exhibits broad substrate scope with excellent functional-group tolerance. The value was demonstrated by the streamlined synthesis of 2-arylpropionic acids (profens) as a potential alternative to the Boots–Hoechst–Celanese route to this important motif. Also, β-chiral nitro compounds can be converted into α-chiral nitriles without any racemization.

## Author contributions

X. L., B. X. and M. O. conceptualized this work. X. L. performed the experiments and B. X. analysed the data. M. O. supervised the research and acquired funding. All authors contributed to the writing and editing of the manuscript.

## Conflicts of interest

There are no conflicts to declare.

## Supplementary Material

SC-017-D6SC04517K-s001

SC-017-D6SC04517K-s002

## Data Availability

The data supporting this article have been included as part of the supplementary information (SI). Supplementary information: reaction optimizations, experimental procedures, full characterization data and copies of NMR spectra. See DOI: https://doi.org/10.1039/d6sc04517k.
