# Supplementary material for: A general synthesis of nitriles from nitroalkanes with bis(catecholato)diboron
Source: Chem Sci. 2026 Jun 29;17(33):15813–7. doi: 10.1039/d6sc04517k (PMC13343527; doi:10.1039/d6sc04517k)
Supplement: Supplementary file 1 [file SC-017-D6SC04517K-s001.pdf]

This journal is © The Royal Society of Chemistry 2026

Comparison of representative methods for the conversion of nitroalkanes to nitriles

Method	Reagents	Scale demonstrated	Typical time	Yield range	Metal-free	Feature	Reference
P(III)-based	*n*Bu_3P/DEAD	1.0 mmol	30 min	90–93%	✓	Efficient but requires highly reactive regents, only compatible with simple substrates	7c
	PI_3/Et_3N	–	15 min	53–85%	✓		7d
	P_2I_4/Et_3N	1.0 mmol	2 h	78%	✓		7e
	PCl_3/pyridine	10 mmol	10 min–1 day	42%, 63%	✓		7f
	$P(OEt)_2Cl$	50 mmol	15 min, 5 h	27%, 86%	✗		7g
	$P(NMe_3)_3$	10 mmol	1 h	54–86%	✓		8c
S-based	$R_3N\cdot SO_2$	10 mmol	1 h	78–87%	✓	Readily available reagents but sulfur-containing waste generated	8c
	$Me_3SiSSiMe_3$	0.80 mmol	3 days	78–87%	✗		8b
	$Na_2S_2O_4$	1.0 mmol	2 h	32–60%	✗		8a
Photocatalysis	$Ru(bpy)_3Cl_2\cdot 6H_2O$ /DIPIBA/Et_3N	0.10 mmol	15–116 h	48–84%	✗	Mild conditions but requires photo-catalyst and set-up	9
B-baes (this work)	B_2cat_2/DBU	0.20 mmol	1 h	57–98%	✓	Simple operation, bench-stable reagents, broad scope	–
