# Supplementary material for: A general synthesis of nitriles from nitroalkanes with bis(catecholato)diboron
Source: Chem Sci. 2026 Jun 29. Online ahead of print. doi: 10.1039/d6sc04517k (PMC13343527; doi:10.1039/d6sc04517k)
Supplement: SC-OLF-D6SC04517K-s002 [file SC-OLF-D6SC04517K-s002.pdf]

Supplementary Information (SI) for Chemical Science.
This journal is © The Royal Society of Chemistry 2026

Supplementary Information for

A general synthesis of nitriles from nitroalkanes with bis(catecholato)diboron

Xiaojie Liu, Biping Xu and Martin Oestreich*

Institut für Chemie, Technische Universität Berlin

Straße des 17. Juni 115, 10623 Berlin, Germany

martin.oestreich@tu-berlin.de

Supplementary Information

Table of Contents

**1**	**General Information**	**S3**
**2**	**Optimization Study**	**S5**
2.1	Optimization of reaction parameters	S5
2.2	General procedure for synthesis of nitriles from nitroalkanes	S9
**3**	**Syntheses of Starting Materials**	**S10**
3.1	Syntheses of alcohols	S10
3.2	Syntheses of alkyl iodides	S12
3.3	Syntheses of nitro-alkyl compounds	S15
**4**	**Characterization Data of Products**	**S21**
4.1	Characterization data of alcohols and alkyl iodides	S21
4.2	Characterization data of nitroalkenes and nitroalkanes	S40
4.3	Characterization data of nitrile products	S67
**5**	**GLC Traces of Starting Material 1u and Product 2u**	**S91**
**6**	**NMR Spectra of Starting Materials and Products**	**S93**
**7**	**References**	**S249**

1 General Information

All reactions were performed in flame-dried glassware using conventional Schlenk techniques under a static pressure of nitrogen unless stated otherwise. Liquids and solutions were transferred with syringes. THF, toluene and Et_2O were distilled from metal Na under nitrogen following standard procedures; 1,4-dioxane, CH_2Cl_2, DMA, DMF and CH_3CN were distilled from CaH_2 under nitrogen following standard procedures; DCE and 1,2-dichlorobenzene were distilled from P_2O_5 under nitrogen following standard procedures. Technical grade solvents for extraction or chromatography (*n*-hexane, *n*-pentane, ethyl acetate, CH_2Cl_2) were distilled prior to use. All commercially available starting materials were purchased from Sigma Aldrich, TCI, ABCR, Fisher, Strem and BLD and used as received unless otherwise noted. Product spots were visualized under UV light (λ_{max} = 254 nm) and with $KMnO_4$ stain. Flash column chromatography was performed on silica gel 60 (40-63 µm, 230-400 mesh, ASTM) by Grace using the indicated solvents. ^1H, ^{13}C NMR spectra were recorded in $CDCl_3$ on Bruker AV400 or AV500 instruments. Chemical shifts were reported in parts per million (ppm) and were referenced to the residual solvent resonance as the internal standard ($CHCl_3$: δ = 7.26 ppm for ^1H NMR and $CDCl_3$: δ = 77.00 ppm for ^{13}C NMR). All other nuclei (^{19}F) was referenced in compliance with the unified scale for NMR chemical shifts as recommended by the IUPAC stating the chemical shift relative to $BF_3{\cdot}Et_2O$, CCl_3F, and Me_4Si. Data were reported as follows: chemical shift, multiplicity (s = singlet, d = doublet, t = triplet, q = quartet, m = multiplet), coupling constants (Hz), and integration. Gas liquid chromatography (GLC) was performed on an *Agilent Technologies 7820A* gas chromatograph equipped with a HP-5 capillary column (30 m × 0.32 mm, 0.25 µm film thickness) by *Agilent Technologies/CS-Chromatographie Service* using the following program: N_2 carrier gas, injection temperature 250 °C, detector temperature 300 °C, flow rate: 1.7 mL/min; temperature program: start temperature 40 °C, heating rate 10 °C/min, end temperature 280 °C for 10 min. Gas-liquid chromatography mass spectrometry (GLC–MS) measurements were conducted on an Agilent Technologies 5975C TAD–GC/MSD-System with electron impact ionization (EI) connected to a fused silica HP-5ms capillary column (length: 30 m, inner diameter: 0.25 mm, thickness of the stationary phase: 0.25 µm). Measurements were performed using the following protocol: Carrier gas: He, injector temperature: 280 °C, detector

temperature: 280 °C, flow rate: 4 mL/min, temperature program: starting temperature: 40 °C, heating rate: 10 °C/min, final temperature: 280 °C for 10 min. Infrared (IR) spectra were recorded on an *Agilent Technologies Cary* 630 FT-IR spectrometer equipped with an ATR unit and the signals were reported in wave-numbers (cm^{-1}). Melting points (M.p.) were determined with a Stuart Scientific SMP20 melting point apparatus and were not corrected. High resolution mass spectra (HRMS) were obtained from the Analytical Facility at the *Institut für Chemie, Technische Universität Berlin* on a Thermo Fisher Scientific LTQ Orbitrap XL apparatus using APCI techniques with a linear ion trap analyzer.

2 Optimization Study

2.1 Optimization of reaction parameters

Table S1. Screening of Nitro-compound activation reagents[a]

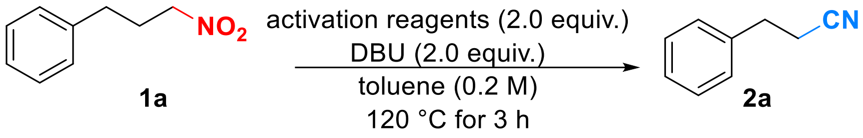

entry	Nitro-compound activation reagents	yield of **2a** (%)[b]
1	B_2cat_2	42
2	B_2pin_2	ND
3	B_2nep_2	ND
4	$B_2(OH)_4$	ND
5	catBH	8
6	catBCl	6

[a]Reaction conditions: 3-Nitropropylbenzene **1a** (0.20 mmol) and toluene (1.0 mL) were mixed, then DBU (2.0 equiv.) and activation reagents (2.0 equiv.) were added, and then the reaction was maintained at 120 °C for 3 h. [b]The yield was determined by gas−liquid chromatography (GLC) analysis with methyl benzoate as an internal standard. ND = not detected.

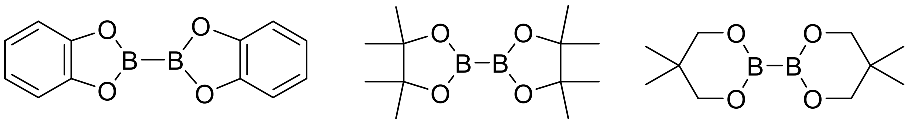

B_2cat_2 B_2pin_2 B_2nep_2

Table S2. Screening of the equivalent of the Nitro-compound activation reagents[a]

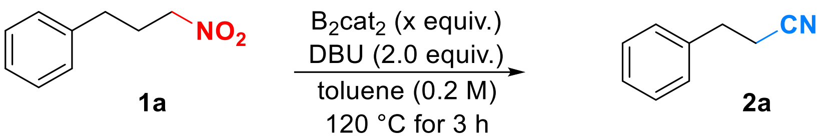

entry	Equivalent of B_2cat_2	yield of **2a** (%)[b]
1	2.0	42
2	1.5	63
3	1.2	99
4	1.0	89

[a]Reaction conditions: 3-Nitropropylbenzene **1a** (0.20 mmol) and toluene (1.0 mL) were mixed, then DBU (2.0 equiv.) and B_2cat_2 (x equiv.) were added, and then the reaction was maintained at 120 °C for 3 h. [b]The yield was determined by gas−liquid chromatography (GLC) analysis with methyl benzoate as an internal standard.

Table S3. Screening of the base[a]

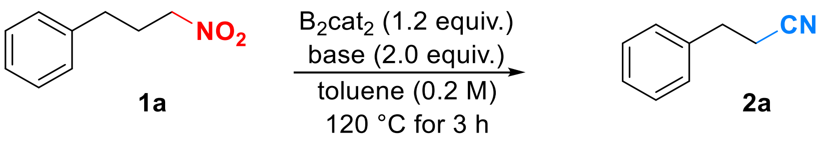

entry	Base	yield of **2a** (%)[b]
1	DBU	99
2	Et_3N	24
3	DIPEA	29
4	DABCO	ND
5	Pyridine	ND
6	K_3PO_4	ND
7	KF	ND
8	DMAP	ND
9	No base	ND
10	DBU (1.0 equiv.)	22

[a]Reaction conditions: 3-Nitropropylbenzene **1a** (0.20 mmol) and toluene (1.0 mL) were mixed,

then base (2.0 equiv.) and B_2cat_2 (1.2 equiv.) were added, and then the reaction was maintained at 120 °C for 3 h. [b]The yield was determined by gas−liquid chromatography (GLC) analysis with methyl benzoate as an internal standard. ND = not detected.

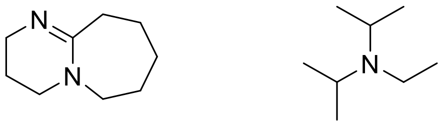

DBU DIPEA DABCO

Table S4. Screening of the solvent[a]

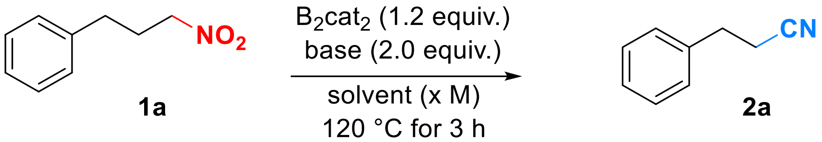

entry	Solvents	yield of **2a** (%)[b]
1	toluene	99
2	$PhCF_3$	97
3	*o*-DCB	95
4	THF	trace
5	CH_3CN	trace
6	DMF	ND
7	DCE	33
8	DMA	ND
9	toluene (0.4 M)	93
10	toluene (0.1 M)	95

[a]Reaction conditions: 3-Nitropropylbenzene **1a** (0.20 mmol) and solvent (1.0 mL) were mixed, then DBU (2.0 equiv.) and B_2cat_2 (1.2 equiv.) were added, and then the reaction was maintained at 120 °C for 3 h. [b]The yield was determined by gas−liquid chromatography (GLC) analysis with methyl benzoate as an internal standard. ND = not detected. *o*-DCB = 1,2-Dichlorobenzene. DMF = *N*,*N*-dimethylformamide. DCE = 1,2-Dichloroethane.

Table S5. Screening of the reaction temperature[a]

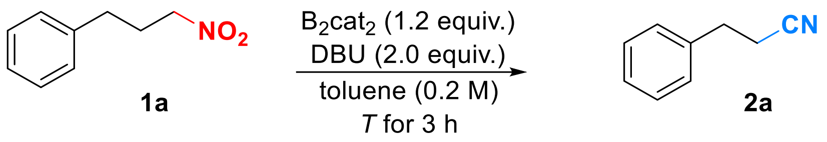

entry	T (Reaction temperature, °C)	yield of **2a** (%)[b]
1	25	trace
2	60	6
3	100	72
4	120	99
5	150	99

[a]Reaction conditions: 3-Nitropropylbenzene **1a** (0.20 mmol) and toluene (1.0 mL) were mixed, then DBU (2.0 equiv.) and B_2cat_2 (1.2 equiv.) were added, and then the reaction was maintained at different temperature for 3 h. [b]The yield was determined by gas−liquid chromatography (GLC) analysis with methyl benzoate as an internal standard.

Table S6. Screening of the reaction time[a]

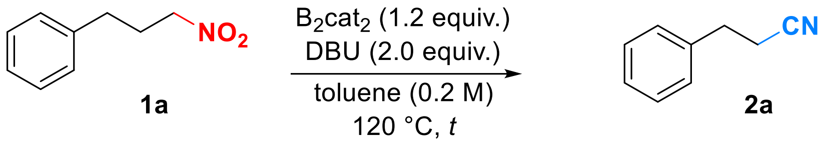

entry	*t* (Reaction time)	yield of **2a** (%)[b]
1	10 min	53
2	30 min	82
3	1 h	99
4	3 h	99
5	12 h	99

[a]Reaction conditions: 3-Nitropropylbenzene **1a** (0.20 mmol) and toluene (1.0 mL) were mixed, then DBU (2.0 equiv.) and B_2cat_2 (1.2 equiv.) were added, and then the reaction was maintained at 120 °C for expected time. [b]The yield was determined by gas−liquid chromatography (GLC) analysis with methyl benzoate as an internal standard.

2.2 General procedure (GP) for deoxygenation of nitro-compounds

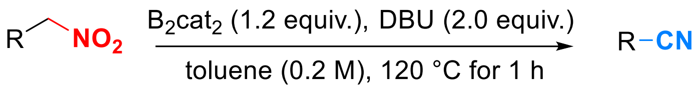

Inside the glove box, a 35 mL pressure tube was equipped with a stir bar, nitro-compounds (0.20 mmol) and toluene (1.0 mL) were mixed, then DBU (61 mg, 0.40 mmol) and B_2cat_2 (57 mg, 0.24 mmol) were subsequently added. Subsequently, the reaction was transferred from the glove box to a 120 °C heating block within the fume hood for 1 h. After the indicated reaction time, the mixture was cooled to room temperature and diluted with ethyl acetate (10 mL). The residue was subjected to GLC analysis with methyl benzoate as the internal standard.

3 Syntheses of Starting Materials

3.1 Syntheses of alcohols

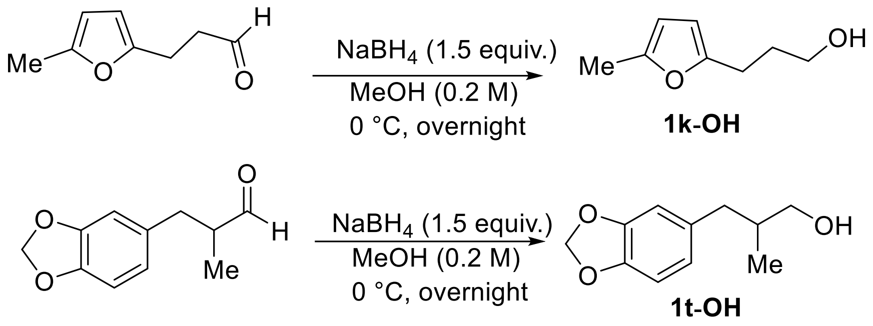

To a 100 mL flame-dried round-bottom Schlenk flask charged with a Teflon-coated magnetic stir-bar and 3-(5-methylfuran-2-yl) propanal (2.1 g, 15 mmol) or 3-(benzo[*d*] [1,3] dioxol-5-yl)-2-methylpropanal (2.9 g, 15 mmol) was added to dry MeOH 75 mL. When the solution was cooled to 0 °C with ice bath, sodium borohydride (0.90 g, 23 mmol) was portion wise added over 15 minutes under a nitrogen flow. Upon finishing addition of sodium borohydride, the reaction was stirred overnight. After the reaction was cooled to room temperature, it was quenched with saturated NH_4Cl solution and extracted with CH_2Cl_2 twice. Then the combined organic layer was dried with anhydrous Na_2SO_4, followed by evaporation under reduced pressure. The residue was purified by flash chromatography on silica gel (eluent = *n*-pentane:ethyl acetate = 5:1) to obtain **1k-OH** as colorless oil with 97%yield and **1t-OH** as colorless oil with 95% yield.

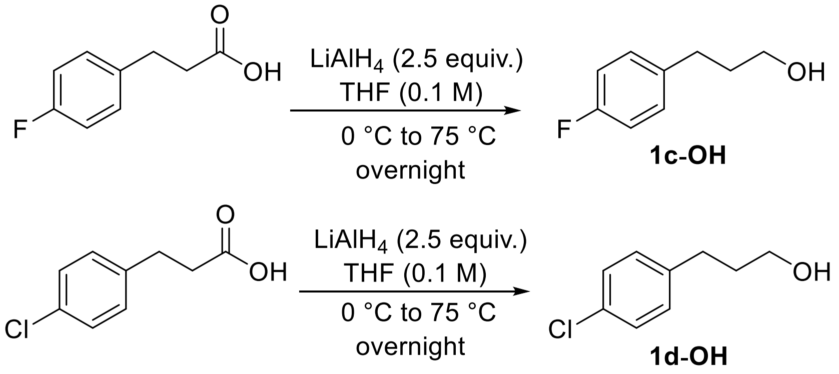

To a 250 mL flame-dried round-bottom Schlenk flask charged with a Teflon-coated magnetic

stir-bar and lithium aluminum hydride ($LiAlH_4$) (1.0 g, 25 mmol) was added dry THF 100 mL. When the solution was cooled to 0 °C with ice bath, 3-(4-fluorophenyl) propanoic acid (1.7 g, 10 mmol) or 3-(4-chlorophenyl) propanoic acid (1.8 g, 10 mmol) was portion wise added over 30 minutes under a nitrogen flow. Upon finishing addition of acid, the reaction mixture was allowed to warm up to 75 °C and stirred for overnight. After the reaction was finished, it was quenched with saturated NH_4Cl solution and extracted with CH_2Cl_2 twice. Then the combined organic layer was dried with anhydrous Na_2SO_4, followed by evaporation under reduced pressure. The residue was purified by flash chromatography on silica gel (eluent = *n*-pentane/ ethyl acetate = 5:1) to obtain the product **1c-OH** as colorless oil with 89% yield and **1d-OH** as colorless oil with 93% yield.

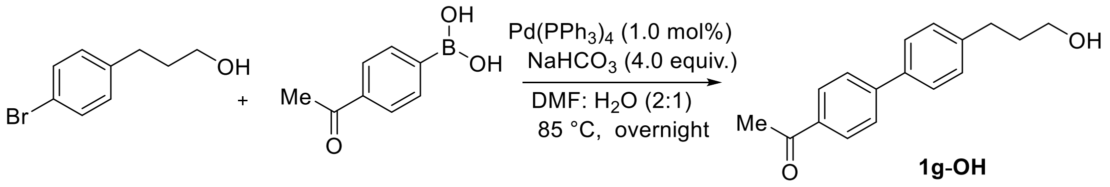

1g-OH

To a 250 mL nitrogen filled round-bottom flask equipped with a Teflon-coated magnetic stir-bar was added 3-(4-Bromophenyl)propan-1-ol (2.2 g, 10 mmol), 4-Acetylphenylboronic acid (2.5 g, 15 mmol), $Pd(PPh_3)_4$ (115 mg, 0.10 mmol) and Sodium bicarbonate (3.4 g, 40 mmol), then DMF 40 mL and water 20 mL were added and the reaction mixture was conducted in 85 °C for overnight. After the indicated reaction time, the reaction mixture was quenched by slow addition of Ethyl acetate (20 mL), the aqueous reaction mixture was extracted three times with Ethyl acetate (20 mL). The combined organic layers were washed with brine, dried over Na_2SO_4 and concentrated under reduced pressure, and the residue was purified by flash chromatography on silica gel (eluent = *n*-pentane: ethyl acetate = 5:1 to 3:1) to obtain the product **1g-OH** as a white solid with 85% yield.

3.2 Syntheses of alkyl iodides

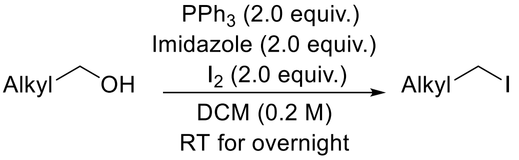

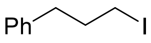
1a-I

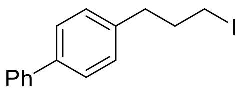
1b-I

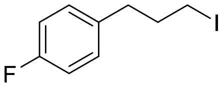
1c-I

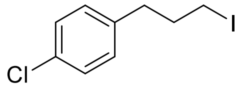
1d-I

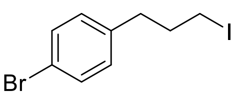
1e-I

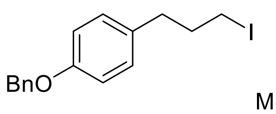
1f-I

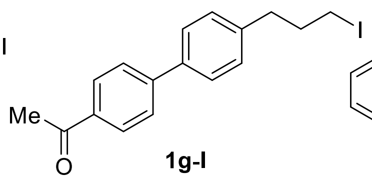
1g-I

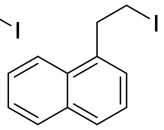
1j-I

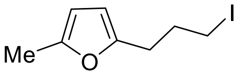
1k-I

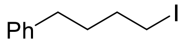
1l-I

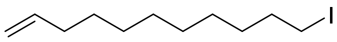
1m-I

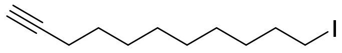
1n-I

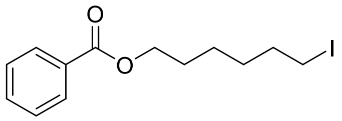
1o-I

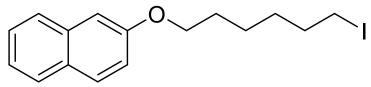
1q-I

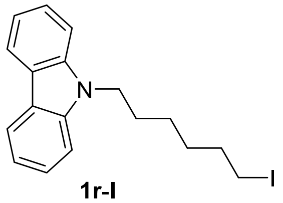
1r-I

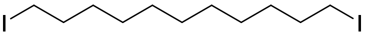
1s-I

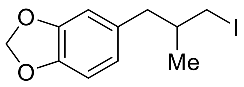
1t-I

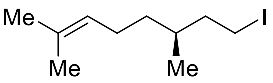
5a-I

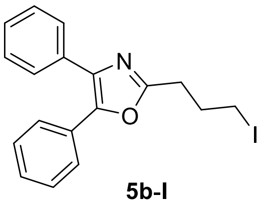
5b-I

To a 100 mL nitrogen filled round-bottom flask equipped with a Teflon-coated magnetic stir-bar was added corresponding alcohols (10 mmol), PPh_3 (5.2 g, 20 mmol) and Imidazole (1.4 g, 20 mmol), I_2 (5.1 g, 20 mmol), anhydrous CH_2Cl_2 50 mL was added and the reaction mixture was cooled down to 0 °C with ice bath, later in room temperature for overnight. After the indicated reaction time, the reaction mixture was filtered through a pad of silica gel. The filtrate was concentrated under reduced pressure and the residue was purified by flash chromatography

on silica gel (eluent = *n*-pentane:ethyl acetate = 10:1 to 5:1) to obtain the corresponding alkyl iodide compounds.

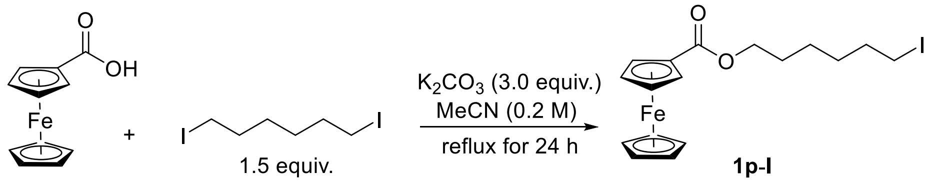

1p-I

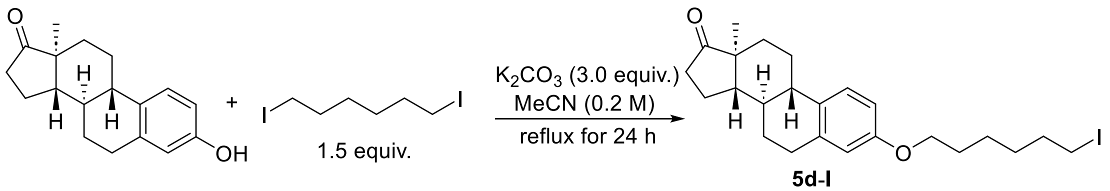

5d-I

To a 100 mL nitrogen filled round-bottom flask equipped with a Teflon-coated magnetic stir-bar was added Ferrocene carboxylic acid (2.3 g, 10 mmol) or Estrone (2.7 g, 10 mmol), 1,6-Iodohexane (5.1 g, 15 mmol) and K_2CO_3 (4.1 g, 30 mmol), then CH_3CN (50 mL) was added *via* syringe before the reaction mixture was allowed to heat to reflux in an oil bath for 24 h. After the indicated reaction time, the reaction mixture was cooled to room temperature, filtered through a pad of silica gel and washed with ethyl acetate. The filtrate was concentrated under reduced pressure and the residue was purified by flash chromatography on silica gel (eluent = *n*-pentane: ethyl acetate = 5:1) to obtain the corresponding products.

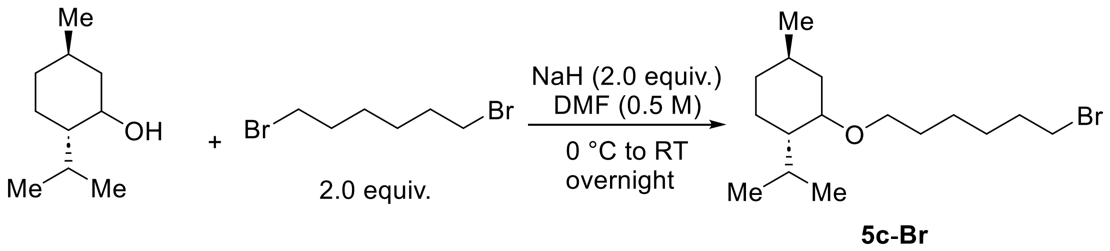

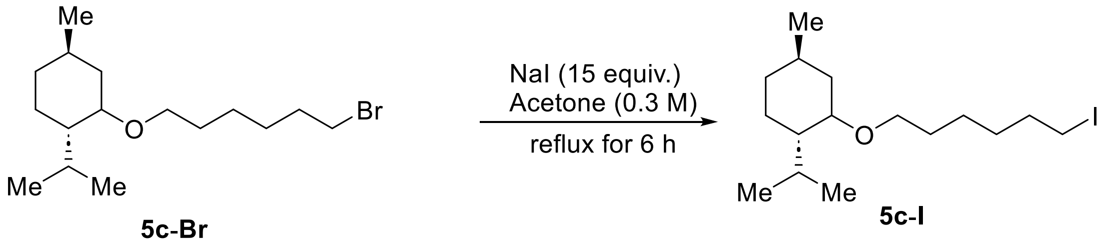

Step 1: An oven-dried Schlenk flask was charged with NaH (60% in mineral oil, 800 mg, 40 mmol). The flask was evacuated and refilled with Argon (three times). Then anhydrous DMF (40 mL) was added. The reaction mixture was cooled to 0 °C, L-menthol (3.1 g, 20 mmol) was added slowly. The reaction mixture was stirred at 0 °C for 30 minutes. 1,6-dibromohexane (9.8 g, 40 mmol) was added slowly and the reaction mixture was stirred at room temperature overnight. The reaction mixture was quenched by slow addition of water (20 mL) at 0 °C. The aqueous reaction mixture was extracted three times with Et_2O (20 mL). The combined organic layers were washed with brine, dried over $MgSO_4$ and concentrated under reduced pressure, and the residue was purified by flash chromatography on silica gel (eluent = *n*-pentane: ethyl acetate = 10:1) to obtain the corresponding product **5c-Br**.

Step 2: To a solution of **5c-Br** (1.6 g, 5.0 mmol) in 15 mL acetone was added NaI (4.2 g, 25 mmol). The mixture was heated to reflux for 6 h. After being allowed to cool down to room temperature, the solvent was evaporated under reduced pressure. Followed by the addition of 15 mL CH_2Cl_2. Then the mixture was filtered, and the filtrate was washed with aqueous Na_2S and brine, dried over Na_2SO_4, and concentrated in vacuo. The resulted residue was purified by short silica gel column chromatography to give the product **5c-I**.

3.3 Syntheses of nitro-alkyl compounds

Method A:

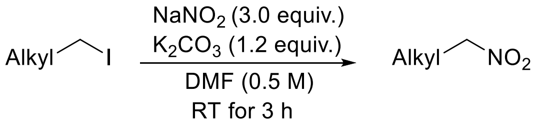

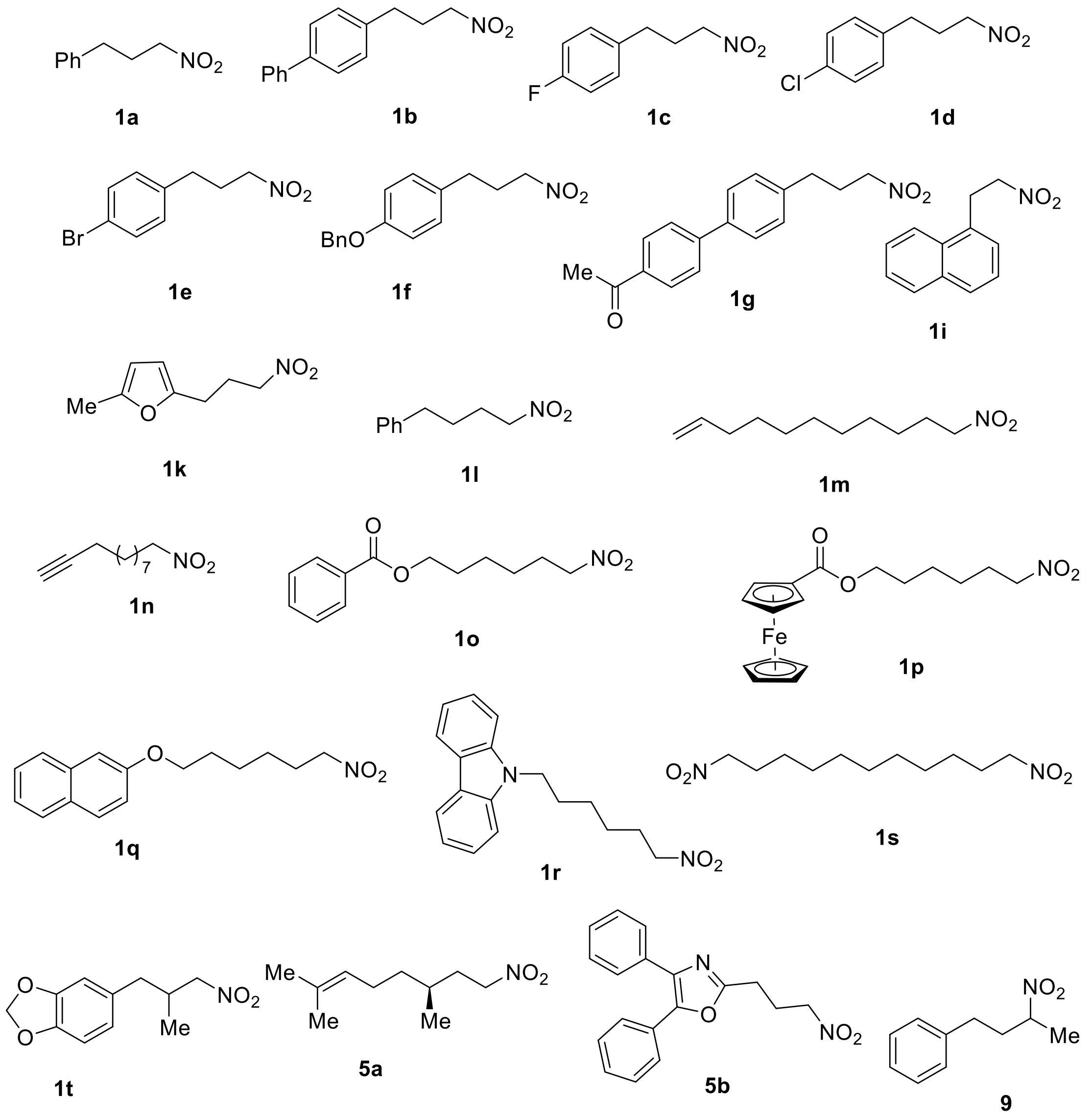

An oven-dried round-bottom flask was charged with corresponding alkyl iodides (5.0 mmol), $NaNO_2$ (1.0 g, 15 mmol), then anhydrous DMF (10 mL) was added. The reaction mixture was stirred at room temperature for 3 h. Then the reaction mixture was quenched by slow addition of water (20 mL), the aqueous reaction mixture was extracted three times with Ethyl acetate (20 mL). The combined organic layers were washed with brine, dried over Na_2SO_4 and

concentrated under reduced pressure, and the residue was purified by flash chromatography on silica gel (eluent = *n*-pentane: ethyl acetate = 10:1 to 5:1) to obtain the corresponding nitro-alkyl products.

Method B:

(a)

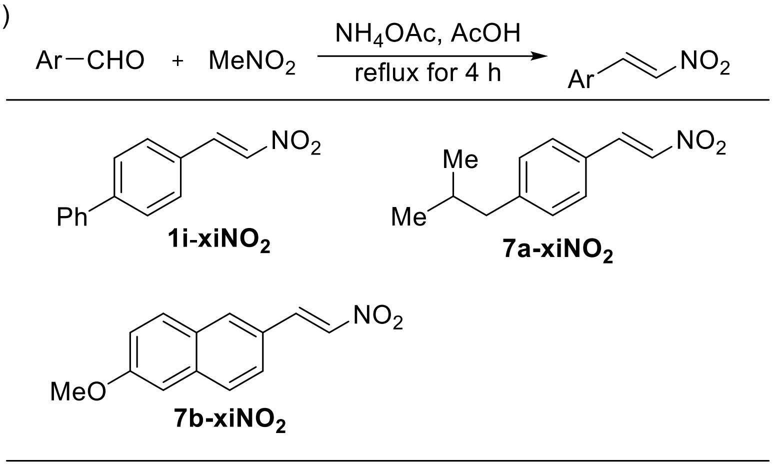

(b)

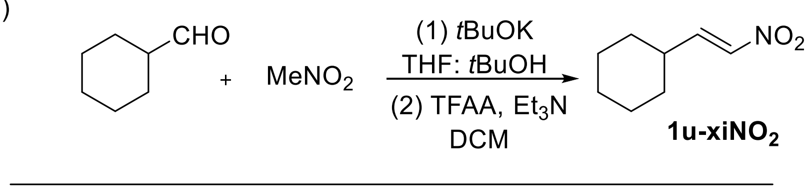

(d)

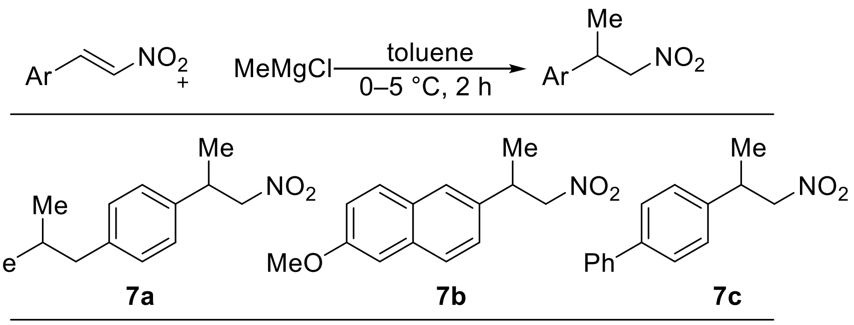

(e)

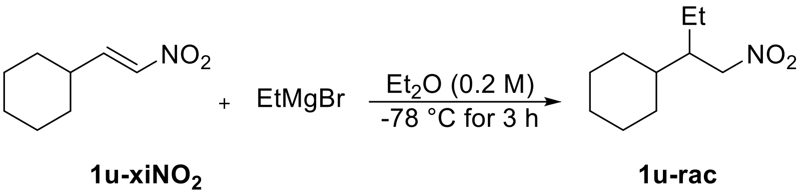

(f)

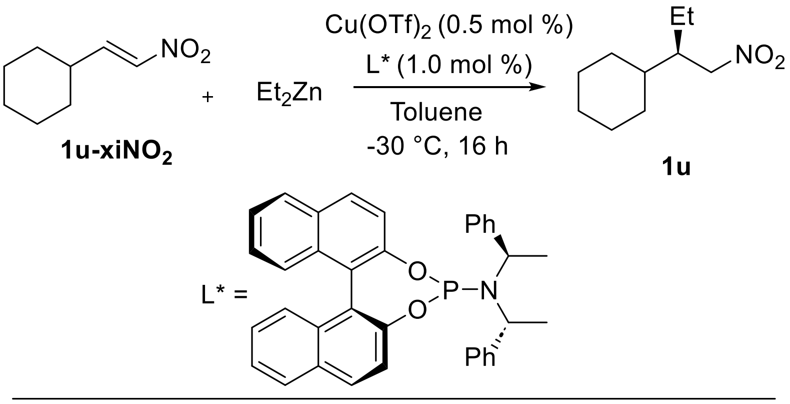

(a): According to the literature [S1], in a 100 mL round bottom flask were added aromatic aldehyde (30 mmol), ammonium acetate (18 mmol), nitromethane (36 mmol) and acetic acid (21 mL). The reaction was heated to reflux under oil-bath and stirred for 4 h, and after cooling to room temperature, the reaction mixture was poured into ice water, a large amount of solid was precipitated in the mixture, filtration, the solid was dried and recrystallized with ethanol, the solid was filtered and washed with ice ethanol, and dried to obtain corresponding nitroolefin.

(b): According to the literature [S2], to a dry round bottom flask was added cyclohexane carboxaldehyde (1.1 g, 10 mmol), nitromethane (840 μL, 16 mmol), and 1:1 THF/*t*-BuOH (10 mL). This solution was cooled to 0 °C and potassium tert-butoxide (0.24 g, 2.1 mmol) added in one portion. The reaction was then stirred at 0 °C for 1 h then warmed to room temperature

and stirred for 12 h. After completion, saturated aqueous NH_4Cl solution (20 mL) was added to quench the reaction and then extracted with CH_2Cl_2 (3 x 20 mL). The combined organic extracts were then dried over anhydrous Na_2SO_4 and concentrated *in vacuo*. After drying the crude residue under vacuum (4 mm) for 1 h, CH_2Cl_2 (20 mL) was added followed by cooling to 0 °C. Trifluoroacetic anhydride (1.5 mL, 11 mmol) was added followed by the slow dropwise addition of Et_3N (3.0 mL, 22 mmol). After stirring for 1 h at 0 °C the reaction was allowed to warm to room temperature and stirred an additional 2 h. The reaction was diluted with CH_2Cl_2 (20 mL) followed by the addition of water (20 mL). The organic layer was separated and washed with saturated aqueous NH_4Cl solution (3 x 20 mL), dried with Na_2SO_4 and concentrated *in vacuo* to give a yellow oil that was purified by column chromatography (eluent = hexane: ether = 20:1) to obtain the compound **1u-xiNO$_2$**.

(c): Under nitrogen atmosphere, a 25 mL round-bottom flask equipped with a Teflon-coated magnetic stir-bar was added **1i-xiNO$_2$** (0.23 g, 1.0 mmol), and anhydrous MeOH (4 mL) was added *via* syringe, then add the sodium borohydride (57 mg, 1.5 mmol) to the mixture slowly over 10 minutes. After that, stir the reaction mixture at room temperature for 8 h. After the indicated reaction time, add an aqueous solution of hydrochloric acid (0.2 M) to the mixture carefully until no gas evolution observe. Then filtered the reaction through a pad of silica gel and washed with ethyl acetate. The filtrate was concentrated under reduced pressure and the residue was purified by flash chromatography on silica gel (eluent = *n*-pentane: ethyl acetate = 5:1) to obtain the corresponding products **1i**.

(d): According to the literature [S1], a mixture of **7a-xiNO$_2$** or **7b-xiNO$_2$** (1.7 mmol) and toluene (2.0 mL) was charged into a round bottom flask under nitrogen atmosphere and cooled to 0–5°C. To this was added a solution of methyl magnesium chloride (0.24 g, 2.4 mol) in tetrahydrofuran (1.0 mL) slowly, and the mixture was stirred for 60 min. On the completion of the reaction (TLC) and the reaction mass was quenched with 10% ammonium chloride solution (4.0 mL), stirred about 15 min, product was extracted into toluene (3 X 2.0 mL) and solvent was

removed under reduced pressure at below 70 °C to afford the corresponding product **7a** or **7b** after column chromatography.

(e): A mixture of **1u-xiNO$_2$** (5.0 mmol) and ether (25 mL) was charged into a round bottom flask under nitrogen atmosphere and cooled to –78°C. To this was added a solution of ethyl magnesium bromide (2.0 mL, 6.0 mol, 3.0 M in tetrahydrofuran) slowly, and the mixture was stirred for 3 h in the same temperature. Then the reaction was quenched with saturated NH_4Cl solution, and extracted with ethyl acetate twice. Then the combined organic layer was dried with anhydrous Na_2SO_4, followed by evaporation under reduced pressure to remove the solvent. The residue was purified by flash chromatography on silica gel to obtain **1u-rac** as yellow oil.

(f): According to the literature [S3], under nitrogen atmosphere, in a flame-dried Schlenk flask, $Cu(OTf)_2$ (9.0 mg, 25 μmol), chiral phosphor amidite ligand L* (27 mg, 50 μmol) was added, and together with 7.7 mL of dry toluene, then the solution was stirred at room temperature for 30 min and then cooled to -45°C. Diethyl zinc (6.0 mmol, 6.0 ml of 1N sol. in hexane) was added dropwise at such a rate as the temperature did not rise above –30°C. The solution was stirred for 15 min at –30°C. The **1u-xiNO$_2$** (0.78 g, 5.0 mmol) was then added dropwise in 5 min. The reaction mixture was stirred at –30°C for 3h before being quenched by aqueous NH_4Cl, and extracted with ethyl acetate twice. Then the combined organic layer was dried with anhydrous Na_2SO_4, followed by evaporation under reduced pressure to remove the solvent. The residue was purified by flash chromatography on silica gel to obtain **1u** as yellow oil. Enantiomeric excess was determined by chiral GC.

Method C:

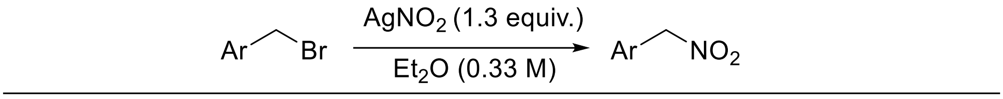

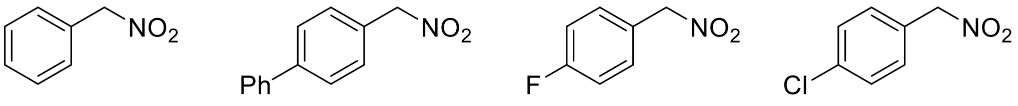

3a **3b** **3c** **3d**

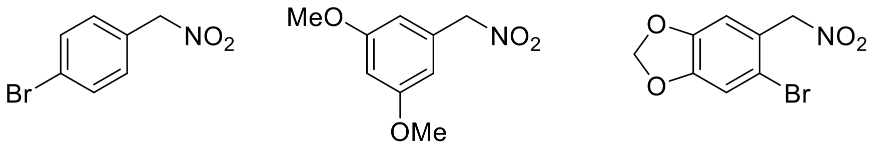

3e **3f** **3g**

According to the literature [S4], Silver nitrite (2.3 g, 13 mmol) was added to a round-bottom flask covered in aluminum foil containing anhydrous diethyl ether (26 mL). After stirring at room temperature for 15 min, the mixture was then cooled at 0 °C. A solution of corresponding benzyl bromide (10 mmol) in diethylether (1.7 mL) was added dropwise via addition funnel. The reaction was stirred at 0 °C for 1 h and then heated to reflux for 4 h. The mixture was filtered over Celite using ethyl acetate as eluent. The product was purified by column chromatography on silica gel (eluting = *n*-pentane: ethyl acetate =90:10).

4 Characterization Data of Corresponding Compounds

4.1 Characterization data of alcohols and alkyl iodides

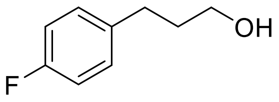

1c-OH

$C_9H_{11}FO$

M = 154.08 g/mol

3-(4-Fluorophenyl) propan-1-ol (**1c-OH**): Prepared from 3-(4-fluorophenyl) propanoic acid (1.7 g, 10 mmol) and lithium aluminum hydride ($LiAlH_4$) (1.0 g, 25 mmol) according to the method mentioned in Section 3. Purification by flash column chromatography on silica gel using *n*-pentane: ethyl acetate = 5:1 afforded **1c-OH** as a colorless oil (1.4 g, 89% yield).

$\mathbf{R_f}$ = 0.40 (*n*-pentane: ethyl acetate = 2:1)

^{1}H NMR (500 MHz, $CDCl_3$) δ 7.16 – 7.13 (m, 2H), 6.96 (t, *J* = 8.7 Hz, 2H), 3.67 (t, *J* = 6.5 Hz, 2H), 2.70 – 2.67 (m, 2H), 1.90 – 1.84 (m, 2H) ppm.

^{13}C NMR (126 MHz, $CDCl_3$) δ 161.30 (d, $J_{C,F}$ = 243.4 Hz), 137.38 (d, $J_{C,F}$ = 3.1 Hz), 129.72 (d, $J_{C,F}$ = 7.8 Hz), 115.10 (d, $J_{C,F}$ = 21.1 Hz), 62.0, 34.3, 31.2 ppm.

^{19}F NMR (471 MHz, $CDCl_3$) δ -117.8 ppm.

IR (ATR): $\tilde{v}$/cm^{-1} = 3325, 2936, 2864, 1886, 1600, 1507, 1449, 1376, 1218, 1156, 1038, 911, 818, 701.

HRMS (APCI) for $C_9H_{12}FO^+$ $[M+H]^+$: calculated 155.0867, found 155.0868.

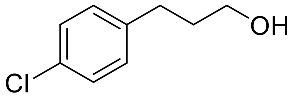

1d-OH

$C_9H_{11}ClO$

M = 170.05 g/mol

3-(4-Chlorophenyl) propan-1-ol (**1d-OH**): Prepared from 3-(4-chlorophenyl) propanoic acid (1.8 g, 10 mmol) and lithium aluminum hydride ($LiAlH_4$) (1.0 g, 25 mmol) according to the method mentioned in Section 3. Purification by flash column chromatography on silica gel using

n-pentane: ethyl acetate = 5:1 afforded **1d-OH** as a colorless oil (1.6 g, 93% yield).

R$_f$ = 0.40 (*n*-pentane: ethyl acetate = 2:1)

^{1}H NMR (400 MHz, $CDCl_3$) δ 7.27 (d, *J* = 8.3 Hz, 2H), 7.15 (d, *J* = 8.4 Hz, 2H), 3.71 – 3.67 (m, 2H), 2.71 (t, 2H), 1.92 – 1.85 (m, 2H) ppm.

^{13}C NMR (101 MHz, $CDCl_3$) δ 140.2, 131.5, 129.8, 128.4, 62.0, 34.0, 31.4 ppm.

IR (**ATR**): ṽ/cm^{-1} = 3305, 3040, 2935, 2863, 2283, 2128, 2073, 1934, 1894, 1490, 1450, 1406, 1350, 1231, 1160, 1091, 1038, 912, 833, 797, 712, 659.

GLC-MS (**EI**) for $C_9H_{11}ClO$ [M$^+$]: calculated 170.05, found 170.0.

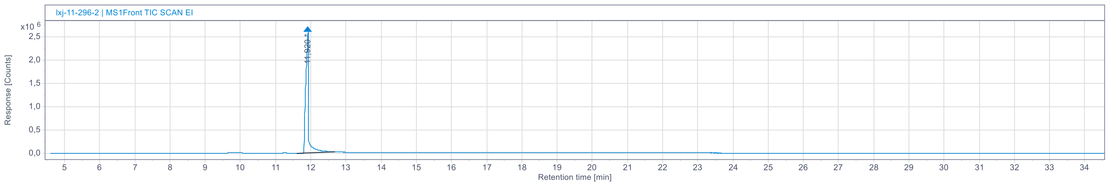

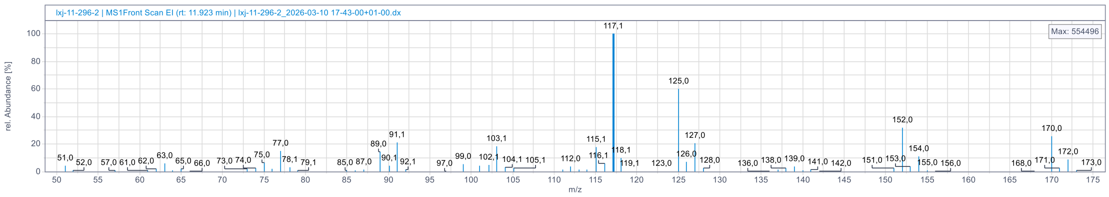

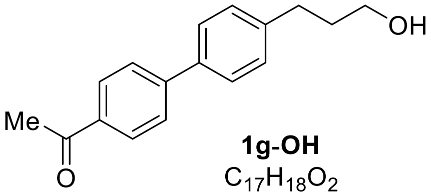

1g-OH
$C_{17}H_{18}O_2$
M = 254.33 g/mol

1-(4'-(3-Hydroxypropyl)-[1,1'-biphenyl]-4-yl) ethan-1-one (**1g-OH**): Prepared from 3-(4-Bromophenyl) propan-1-ol (2.2 g, 10 mmol) and 4-Acetylphenylboronic acid (2.5 g, 15 mmol) according to the method mentioned in Section 3. Purification by flash column chromatography on silica gel using *n*-pentane:ethyl acetate = 2:1 afforded **1g-OH** as a white solid (2.2 g, 85% yield).

R$_f$ = 0.30 (*n*-pentane: ethyl acetate = 2:1)

M.p.: 152–154 °C.

^{1}H NMR (400 MHz, $CDCl_3$) δ 8.02 (d, *J* = 8.4 Hz, 2H), 7.67 (d, *J* = 8.5 Hz, 2H), 7.56 (d, *J* = 8.2 Hz, 2H), 7.31 (d, *J* = 8.0 Hz, 2H), 3.72 (t, *J* = 6.4 Hz, 2H), 2.78 (t, *J* = 7.7 Hz, 2H), 2.64 (s, 3H), 1.98 – 1.91 (m, 2H).

^{13}C NMR (101 MHz, $CDCl_3$) δ 197.8, 145.6, 142.2, 137.5, 135.7, 129.1, 128.9, 127.3, 127.0, 62.2, 34.1, 31.7, 26.6.

IR (**ATR**): ṽ/cm^{-1} = 3416, 2919, 2886, 2332, 2118, 2090, 1923, 1807, 1658, 1598, 1522, 1398, 1356, 1313, 1270, 1181, 1123, 1065, 1021, 961, 917, 820, 750.

HRMS (**APCI**) for $C_{17}H_{19}O_2^+$ [M+H]$^+$: calculated 255.1380, found 255.1377.

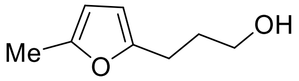

1k-OH

$C_8H_{12}O_2$

M = 140.08 g/mol

3-(5-Methylfuran-2-yl) propan-1-ol (**1k-OH**): Prepared from 3-(5-methylfuran-2-yl) propanal (2.1 g, 15 mmol) and sodium borohydride (0.9 g, 23 mmol) according to the method mentioned in Section 3. Purification by flash column chromatography on silica gel using *n*-pentane:ethyl acetate = 5:1 afforded **1k-OH** as a colorless oil (2.0 g, 97% yield).

R$_f$ = 0.30 (*n*-pentane: ethyl acetate = 2:1)

^{1}H NMR (400 MHz, $CDCl_3$) δ 5.87 (d, J = 3.0 Hz, 1H), 5.84 (d, J = 3.0 Hz, 1H), 3.69 (t, J = 6.4 Hz, 2H), 2.68 (t, J = 7.4 Hz, 2H), 2.25 (s, 3H), 1.92 – 1.85 (m, 2H) ppm.

^{13}C NMR (101 MHz, $CDCl_3$) δ 153.6, 150.4, 105.8, 105.6, 62.1, 31.1, 24.3, 13.5 ppm.

IR (**ATR**): ṽ/cm^{-1} = 3339, 2943, 2251, 1569, 1446, 1383, 1218, 1169, 1051, 943, 778.

HRMS (**APCI**) for $C_8H_{11}O_2^+$ [M-H]$^+$: calculated 139.0754, found 139.0754.

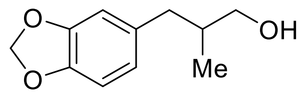

1t-OH
$C_{11}H_{14}O_3$
M = 194.09 g/mol

3-(Benzo[d] [1,3] dioxol-5-yl)-2-methylpropan-1-ol (**1t-OH**): Prepared from 3-(benzo[d] [1,3] dioxol-5-yl)-2-methylpropanal (2.9 g, 15 mmol) and sodium borohydride (0.9 g, 23 mmol) according to the method mentioned in Section 3. Purification by flash column chromatography on silica gel using *n*-pentane:ethyl acetate = 5:1 afforded **1t-OH** as a colorless oil (2.8 g, 95% yield).

R_f = 0.20 (*n*-pentane: ethyl acetate = 2:1)

^{1}H NMR (500 MHz, $CDCl_3$) δ 6.71 (d, *J* = 8.0 Hz, 1H), 6.66 (s, 1H), 6.60 (d, *J* = 7.9 Hz, 1H), 5.90 (s, 2H), 3.50 – 3.47 (m, 1H), 3.45 – 3.41 (m, 1H), 2.66 (dd, *J* = 13.6, 6.3 Hz, 1H), 2.34 – 2.26 (m, 2H), 1.89 – 1.83 (m, 1H), 0.89 (d, *J* = 6.9 Hz, 3H) ppm.

^{13}C NMR (126 MHz, $CDCl_3$) δ 147.3, 145.5, 134.3, 121.8, 109.3, 100.6, 67.3, 39.2, 37.7, 16.2 ppm.

IR (ATR): ṽ/cm^{-1} = 3337, 2874, 2299, 2121, 1846, 1716, 1607, 1486, 1439, 1360, 1241, 1186, 1097, 1031, 930, 862, 801.

HRMS (APCI) for $C_{11}H_{15}O_3^+$ $[M+H]^+$: calculated 195.1016, found 195.1014.

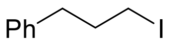

1a-I
$C_9H_{11}I$
M = 245.99 g/mol

(**3-Iodopropyl**) **benzene** (**1a-I**): Synthetized according to the method mentioned in Section 3 with a scale of 10 mmol. Purification by flash column chromatography on silica gel using *n*-pentane:ethyl acetate = 10:1 and obtained as a colorless oil.

R_f = 0.90 (*n*-pentane: ethyl acetate = 90:10).

^{1}H NMR (400 MHz, $CDCl_3$) δ 7.34 (t, *J* = 7.0 Hz, 2H), 7.28 – 7.25 (m, 3H), 3.22 (t, *J* = 6.8 Hz, 2H), 2.79 (t, *J* = 7.3 Hz, 2H), 2.22 – 2.15 (m, 2H) ppm.

^{13}C NMR (101 MHz, $CDCl_3$) δ 140.3, 128.5, 128.4, 126.1, 36.1, 34.8, 6.3 ppm.

IR (ATR): ṽ/cm^{-1} = 3024, 2930, 2296, 2088, 1601, 1494, 1451, 1347, 1263, 1212, 1164, 1072, 1028, 906, 850, 741, 696.

HRMS (APCI) for $C_9H_{11}I^+$ [M]$^+$: calculated 245.9900, found 245.9894.

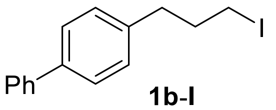

1b-I
$C_{15}H_{15}I$
M = 322.02 g/ mol

4-(3-Iodopropyl)-1,1'-biphenyl (**1b-I**): Synthetized according to the method mentioned in Section 3 with a scale of 10 mmol. Purification by flash column chromatography on silica gel using *n*-pentane:ethyl acetate = 10:1 and obtained as a white solid.

R_f = 0.85 (*n*-pentane: ethyl acetate = 90:10).

M.p.: 43–45 °C.

^{1}H NMR (400 MHz, CDCl3) δ 7.60 (d, J = 7.1 Hz, 2H), 7.55 (d, J = 8.2 Hz, 2H), 7.45 (t, J = 7.6 Hz, 2H), 7.38 – 7.33 (m, 1H), 7.30 – 7.28 (m, 2H), 3.22 (t, J = 6.8 Hz, 2H), 2.79 (t, J = 7.3 Hz, 2H), 2.22 – 2.15 (m, 2H) ppm.

^{13}C NMR (101 MHz, $CDCl_3$) δ 140.9, 139.5, 139.1, 129.0, 128.7, 127.2, 127.1, 127.0, 35.8, 34.8, 6.3 ppm.

HRMS (APCI) for $C_{15}H_{15}I^+$ [M]$^+$: calculated 322.0213, found 322.0206.

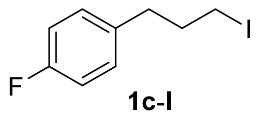

1c-I

$C_9H_{10}FI$
M = 263.98 g/mol

1-Fluoro-4-(3-iodopropyl) benzene (**1c-I**): Synthetized according to the method mentioned in Section 3 with a scale of 10 mmol. Purification by flash column chromatography on silica gel using *n*-pentane:ethyl acetate = 10:1 and obtained as a colorless oil.

$\mathbf{R_f}$ = 0.90 (*n*-pentane: ethyl acetate = 90:10).

^{1}H NMR (500 MHz, $CDCl_3$) δ 7.17 – 7.14 (m, 2H), 6.98 (t, J = 8.7 Hz, 2H), 3.16 (t, J = 6.8 Hz, 2H), 2.71 (t, J = 7.3 Hz, 2H), 2.13 – 2.08 (m, 2H) ppm.

^{13}C NMR (126 MHz, $CDCl_3$) δ 161.48 (d, $J_{C,F}$ = 244.0 Hz), 135.98 (d, $J_{C,F}$ = 3.2 Hz), 129.89 (d, $J_{C,F}$ = 7.7 Hz), 115.25 (d, $J_{C,F}$ = 21.2 Hz), 35.4, 34.9, 5.9 ppm.

^{19}F NMR (471 MHz, $CDCl_3$) δ -117.1 ppm.

IR (ATR): ṽ/cm^{-1} = 3037, 2931, 2855, 2270, 1882, 1760, 1600, 1506, 1446, 1348, 1216, 1156, 1088, 1015, 956, 821, 763, 718.

HRMS (APCI) for $C_9H_{10}FI^+$ $[M]^+$: calculated 263.9806, found 263.9803.

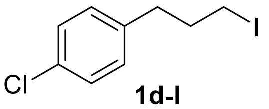

1d-I

$C_9H_{10}ClI$
M = 279.95 g/mol

1-Chloro-4-(3-iodopropyl) benzene (**1d-I**): Synthetized according to the method mentioned in Section 3 with a scale of 10 mmol. Purification by flash column chromatography on silica gel using *n*-pentane:ethyl acetate = 10:1 and obtained as a colorless oil.

$\mathbf{R_f}$ = 0.90 (*n*-pentane: ethyl acetate = 90:10).

^{1}H NMR (400 MHz, $CDCl_3$) δ 7.18 (d, J = 8.4 Hz, 2H), 7.05 (d, J = 8.3 Hz, 2H), 3.07 (t, J = 6.8

Hz, 2H), 2.63 (t, *J* = 7.3 Hz, 2H), 2.09 – 1.99 (m, 2H) ppm.

^{13}C NMR (101 MHz, $CDCl_3$) δ 138.8, 131.9, 129.9, 128.6, 35.5, 34.6, 6.0 ppm.

IR (**ATR**): ṽ/cm^{-1} = 3653, 3024, 2930, 2853, 2654, 2580, 2373, 2295, 2110, 2071, 1994, 1944, 1891, 1775, 1638, 1597, 1490, 1446, 1424, 1347, 1262, 1210, 1163, 1089, 1013, 957, 814, 744, 698, 669.

HRMS (APCI) for $C_9H_{10}Cl^+$ [M-I]$^+$: calculated 153.0466, found 153.0462.

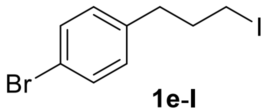

1e-I

$C_9H_{10}BrI$

M = 323.90 g/mol

1-Bromo-4-(3-iodopropyl) benzene (**1e-I**): Synthetized according to the method mentioned in Section 3 with a scale of 10 mmol. Purification by flash column chromatography on silica gel using *n*-pentane:ethyl acetate = 10:1 and obtained as a colorless oil.

R_f = 0.90 (*n*-pentane: ethyl acetate = 90:10).

^{1}H NMR (400 MHz, $CDCl_3$) δ 7.41 (d, *J* = 8.5 Hz, 2H), 7.08 (d, *J* = 8.5 Hz, 2H), 3.15 (t, *J* = 6.7 Hz, 2H), 2.69 (t, *J* = 7.3 Hz, 2H), 2.13 – 2.06 (m, 2H) ppm.

^{13}C NMR (101 MHz, $CDCl_3$) δ 139.3, 131.5, 130.3, 119.9, 35.5, 34.5, 5.9 ppm.

IR (ATR): ṽ/cm^{-1} = 3020, 2931, 2853, 2297, 1892, 1589, 1486, 1444, 1424, 1346, 1262, 1210, 1164, 1071, 1009, 958, 812, 724.

HRMS (APCI) for $C_9H_{10}BrI^+$ [M]$^+$: calculated 323.9005, found 323.9001.

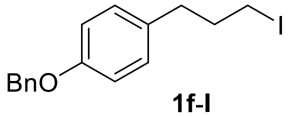

1f-I

$C_{16}H_{17}IO$

M = 352.03 g/mol

1-(Benzyloxy)-4-(3-iodopropyl) benzene (**1f-I**): Synthetized according to the method mentioned in Section 3 with a scale of 10 mmol. Purification by flash column chromatography on silica gel using *n*-pentane:ethyl acetate = 10:1 and obtained as a colorless oil.

R_f = 0.70 (*n*-pentane: ethyl acetate = 90:10).

^{1}H NMR (400 MHz, $CDCl_3$) δ 7.45 (d, *J* = 6.7 Hz, 2H), 7.42 – 7.38 (m, 2H), 7.36 – 7.32 (m, 1H), 7.13 (d, *J* = 8.6 Hz, 2H), 6.92 (d, *J* = 8.6 Hz, 2H), 5.06 (s, 2H), 3.17 (t, *J* = 6.8 Hz, 2H), 2.68 (t, *J* = 7.2 Hz, 2H), 2.14 – 2.07 (m, 2H) ppm.

^{13}C NMR (101 MHz, $CDCl_3$) δ 157.2, 137.1, 132.7, 129.5, 128.5, 127.9, 127.4, 114.8, 70.0, 35.2, 35.0, 6.5 ppm.

IR (ATR): ṽ/cm^{-1} = 3651, 3546, 3028, 2926, 2856, 2466, 2317, 2101, 1950, 1875, 1753, 1608, 1508, 1452, 1379, 1296, 1213, 1174, 1100, 1019, 913, 859, 823, 734, 694.

HRMS (APCI) for $C_{16}H_{17}IO^+$ $[M]^+$: calculated 352.0319, found 352.0314.

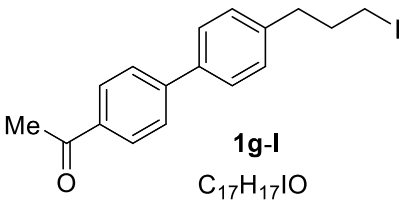

1g-I
$C_{17}H_{17}IO$
M = 364.03 g/mol

1-(4'-(3-Iodopropyl)-[1,1'-biphenyl]-4-yl) ethan-1-one (**1g-I**): Synthetized according to the method mentioned in Section 3 with a scale of 10 mmol. Purification by flash column chromatography on silica gel using *n*-pentane:ethyl acetate = 10:1 and obtained as a white solid.

R_f = 0.60 (*n*-pentane: ethyl acetate = 90:10).

M.p.: 121–123 °C.

^{1}H NMR (400 MHz, $CDCl_3$) δ 8.03 (d, *J* = 8.5 Hz, 2H), 7.67 (d, *J* = 8.5 Hz, 2H), 7.57 (d, *J* = 8.4 Hz, 2H), 7.31 (d, *J* = 7.8 Hz, 2H), 3.25 – 3.16 (m, 2H), 2.79 (t, *J* = 7.3 Hz, 2H), 2.64 (s,

3H), 2.21 – 2.14 (m, 2H).

^{13}C NMR (101 MHz, $CDCl_3$) δ 197.7, 145.5, 140.7, 137.8, 135.7, 129.2, 128.9, 127.4, 127.0, 35.9, 34.7, 26.6, 6.1.

IR (ATR): ṽ/cm^{-1} = 3344, 3030, 2931, 2329, 2117, 1998, 1906, 1805, 1678, 1598, 1576, 1524, 1421, 1394, 1357, 1264, 1166, 1137, 1002, 958, 817, 783, 728, 663.

HRMS (APCI) for $C_{17}H_{18}IO^+$ [M+H]$^+$: calculated 365.0397, found 365.0395.

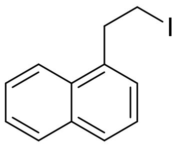

1j-I
$C_{12}H_{11}I$
M = 281.99 g/mol

4-(3-Iodopropyl)-1,1'-biphenyl (**1j-I**): Synthetized according to the method mentioned in Section 3 with a scale of 10 mmol. Purification by flash column chromatography on silica gel using *n*-pentane:ethyl acetate = 10:1 and obtained as a colorless oil.

R$_f$ = 0.85 (*n*-pentane: ethyl acetate = 90:10).

^{1}H NMR (400 MHz, $CDCl_3$) δ 7.99 (d, *J* = 8.8 Hz, 1H), 7.89 (d, *J* = 1.5 Hz, 1H), 7.81 (d, *J* = 8.2 Hz, 1H), 7.58 – 7.49 (m, 2H), 7.46 – 7.42 (m, 1H), 7.37 (d, *J* = 7.0 Hz, 1H), 3.71 – 3.62 (m, 2H), 3.48 (t, *J* = 8.3 Hz, 2H) ppm.

^{13}C NMR (101 MHz, $CDCl_3$) δ 136.8, 133.9, 131.2, 128.9, 127.7, 126.5, 126.3, 125.7, 125.5, 123.1, 37.9, 4.4 ppm.

IR (**ATR**): ṽ/cm^{-1} = 3041, 2958, 2650, 2293, 2103, 1991, 1920, 1803, 1595, 1508, 1423, 1393, 1303, 1237, 1166, 1077, 1013, 966, 918, 872, 771.

HRMS (APCI) for $C_{12}H_{11}^+$ [M-I]$^+$: calculated 155.0855, found 155.0855.

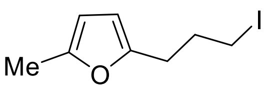

1k-I
$C_8H_{11}IO$
M = 249.99 g/mol

2-(3-Iodopropyl)-5-methylfuran (**1k-I**): Synthetized according to the method mentioned in Section 3 with a scale of 10 mmol. Purification by flash column chromatography on silica gel using *n*-pentane:ethyl acetate = 10:1 and obtained as a colorless oil.

R$_f$ = 0.70 (*n*-pentane: ethyl acetate = 90:10).

^{1}H NMR (500 MHz, $CDCl_3$) δ 5.91 (d, *J* = 3.1 Hz, 1H), 5.84 (d, *J* = 3.4 Hz, 1H), 3.20 (t, *J* = 6.9 Hz, 2H), 2.70 (t, *J* = 7.1 Hz, 2H), 2.25 (s, 3H), 2.15 – 2.10 (m, 2H) ppm.

^{13}C NMR (126 MHz, $CDCl_3$) δ 152.1, 150.7, 106.4, 105.9, 31.9, 28.7, 13.5, 5.9 ppm.

IR (ATR): ṽ/cm^{-1} = 3102, 2919, 2842, 2111, 1616, 1566, 1427, 1382, 1280, 1216, 1163, 1114, 1018, 931, 853, 777.

HRMS (APCI) for $C_8H_{12}IO^+$ $[M+H]^+$: calculated 250.9927, found 250.9924.

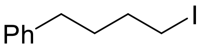

1l-I
$C_{10}H_{13}I$
M = 260.01 g/mol

(**4-Iodobutyl**) **benzene** (**1l-I**): Synthetized according to the method mentioned in Section 3 with a scale of 10 mmol. Purification by flash column chromatography on silica gel using *n*-pentane:ethyl acetate = 10:1 and obtained as a colorless oil.

R$_f$ = 0.90 (*n*-pentane: ethyl acetate = 90:10).

^{1}H NMR (400 MHz, CDCl3) δ 7.30 (t, J = 7.2 Hz, 2H), 7.22 – 7.18 (m, 3H), 3.21 (t, J = 6.9 Hz, 2H), 2.65 (t, J = 7.6 Hz, 2H), 1.90 – 1.83 (m, 2H), 1.78 – 1.71 (m, 2H) ppm.

^{13}C NMR (101 MHz, $CDCl_3$) δ 141.8, 128.4, 125.9, 34.7, 32.9, 32.2, 6.7 ppm.

IR (ATR): ṽ/cm^{-1} = 3023, 2929, 2853, 2080, 1943, 1601, 1494, 1452, 1350, 1204, 1164, 1070, 906, 802, 742, 696.

HRMS (APCI) for $C_{10}H_{12}I^+$ [M-H]$^+$: calculated 258.9978, found 258.9976.

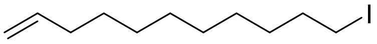

1m-I
$C_{11}H_{21}I$
M = 280.07 g/mol

11-Iodoundec-1-ene (**1m-I**): Synthetized according to the method mentioned in Section 3 with a scale of 10 mmol. Purification by flash column chromatography on silica gel using *n*-pentane:ethyl acetate = 10:1 and obtained as a colorless oil.

R$_f$ = 0.90 (*n*-pentane: ethyl acetate = 90:10).

^{1}H NMR (400 MHz, $CDCl_3$) δ 5.86 – 5.76 (m, 1H), 5.01 – 4.92 (m, 2H), 3.18 (t, *J* = 7.0 Hz, 2H), 2.07 – 2.01 (m, 2H), 1.85 – 1.78 (m, 2H), 1.40– 1.35 (m, 4H), 1.30 – 1.28 (m, 8H) ppm.

^{13}C NMR (101 MHz, $CDCl_3$) δ 139.2, 114.1, 33.8, 33.5, 30.5, 29.3, 29.0, 28.9, 28.5, 7.3 ppm.

IR (ATR): ṽ/cm^{-1} = 3074, 2922, 2851, 2683, 1930, 1820, 1639, 1460, 1367, 991, 907, 720.

HRMS (APCI) for $C_{11}H_{22}I^+$ [M+H]$^+$: calculated 281.0761, found 281.0753.

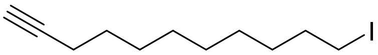

1n-I
$C_{11}H_{19}I$
M = 278.05 g/mol

11-Iodoundec-1-yne (**1n-I**): Synthetized according to the method mentioned in Section 3 with a scale of 10 mmol. Purification by flash column chromatography on silica gel using *n*-pentane:ethyl acetate = 10:1 and obtained as a colorless oil.

R$_f$ = 0.90 (*n*-pentane: ethyl acetate = 90:10).

^{1}H NMR (400 MHz, $CDCl_3$) δ 3.18 (t, *J* = 7.0 Hz, 2H), 2.20 – 2.16 (m, 2H), 1.93 (s, 1H), 1.85 –

1.78 (m, 2H), 1.55 – 1.50 (m, 2H), 1.40 – 1.36 (m, 4H), 1.32 – 1.29 (m, 6H) ppm.

^{13}C NMR (101 MHz, $CDCl_3$) δ 84.7, 68.1, 33.5, 30.4, 29.2, 28.9, 28.6, 28.4, 28.4, 18.4, 7.3 ppm.

IR (ATR): ṽ/cm^{-1} = 3299, 2924, 2852, 2681, 2115, 1460, 1349, 1218, 1178, 1090, 720.

HRMS (APCI) for $C_{11}H_{20}I^+$ $[M+H]^+$: calculated 279.0604, found 279.0596.

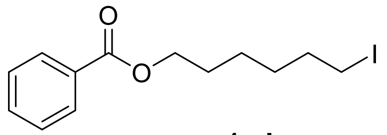

1o-I
$C_{13}H_{17}IO_2$
M = 332.03 g/mol

7-Iodoheptyl benzoate (**1o-I**): Synthetized according to the method mentioned in Section 3 with a scale of 10 mmol. Purification by flash column chromatography on silica gel using *n*-pentane:ethyl acetate = 10:1 and obtained as a colorless oil.

R_f = 0.50 (*n*-pentane: ethyl acetate = 90:10).

^{1}H NMR (400 MHz, $CDCl_3$) δ 8.04 (d, *J* = 6.9 Hz, 2H), 7.59 – 7.53 (m, 1H), 7.46 – 7.42 (m, 2H), 4.32 (t, *J* = 6.6 Hz, 2H), 3.20 (t, *J* = 7.0 Hz, 2H), 1.87 – 1.77 (m, 4H), 1.50 – 1.46 (m, 4H) ppm.

^{13}C NMR (101 MHz, $CDCl_3$) δ 166.6, 132.8, 130.4, 129.5, 128.3, 64.8, 33.3, 30.1, 28.5, 25.0, 6.8 ppm.

IR (ATR): ṽ/cm^{-1} = 3421, 3062, 2931, 2339, 2107, 1713, 1600, 1450, 1386, 1267, 1173, 1110, 1025, 954, 805, 707.

HRMS (APCI) for $C_{13}H_{18}IO_2^+$ $[M+H]^+$: calculated 333.0346, found 333.0342.

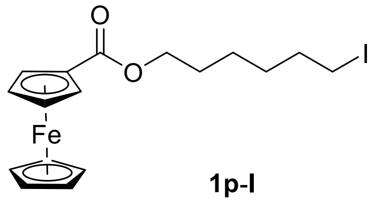

1p-I

$C_{17}H_{21}FeIO_2$
M = 439.99 g/mol

6-Iodohexyl ferrocene carboxylate (**1p-I**): Synthetized according to the method mentioned in Section 3 with a scale of 10 mmol. Purification by flash column chromatography on silica gel using *n*-pentane:ethyl acetate = 10:1 and obtained as a yellow oil.

R_f = 0.40 (*n*-pentane: ethyl acetate = 90:10).

^{1}H NMR (500 MHz, $CDCl_3$) δ 4.81 – 4.80 (m, 2H), 4.39 (t, *J* = 2.0 Hz, 2H), 4.22 (d, *J* = 6.6 Hz, 2H), 4.19 (d, *J* = 0.7 Hz, 5H), 3.21 (t, *J* = 6.9 Hz, 2H), 1.89 – 1.84 (m, 2H), 1.74 (t, *J* = 7.0 Hz, 2H), 1.50 – 1.47 (m, 4H) ppm.

^{13}C NMR (126 MHz, $CDCl_3$) δ 171.7, 71.2, 70.1, 69.7, 64.0, 33.4, 30.2, 28.8, 25.0, 6.7 ppm.

IR (ATR): $\tilde{v}/cm^{-1}$ = 3929, 3407, 3094, 2929, 2855, 2401, 2237, 2086, 1877, 1704, 1457, 1372, 1270, 1206, 1130, 1024, 959, 819, 772, 723.

HRMS (APCI) for $C_{17}H_{22}FeIO_2^+$ $[M+H]^+$: calculated 441.0003, found 441.0000.

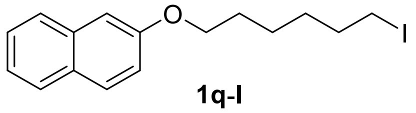

1q-I

$C_{16}H_{19}IO$
M = 354.05 g/mol

2-((6-Iodohexyl) oxy) naphthalene (**1q-I**): Synthetized according to the method mentioned in Section 3 with a scale of 10 mmol. Purification by flash column chromatography on silica gel using *n*-pentane:ethyl acetate = 10:1 and obtained as a colorless oil.

R_f = 0.70 (*n*-pentane: ethyl acetate = 90:10).

^{1}H NMR (400 MHz, $CDCl_3$) δ 7.77 – 7.71 (m, 3H), 7.43 (t, *J* = 7.5 Hz, 1H), 7.33 (t, *J* = 7.4 Hz,

1H), 7.16– 7.13 (m, 2H), 4.08 (t, *J* = 6.4 Hz, 2H), 3.22 (t, *J* = 7.0 Hz, 2H), 1.92 – 1.84 (m, 4H), 1.59– 1.48 (m, 4H) ppm.

^{13}C NMR (101 MHz, $CDCl_3$) δ 157.0, 134.6, 129.3, 128.9, 127.6, 126.7, 126.3, 123.5, 118.9, 106.5, 67.7, 33.4, 30.3, 29.0, 25.1, 7.0 ppm.

IR (ATR): ṽ/cm^{-1} = 3053, 2930, 2854, 2647, 2341, 2096, 1895, 1626, 1598, 1509, 1461, 1388, 1255, 1214, 1178, 1142, 1031, 834, 744.

HRMS (APCI) for $C_{16}H_{19}IO^+$ [M]$^+$: calculated 354.0475, found 354.0469.

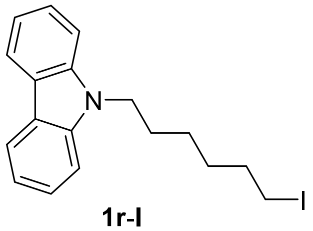

1r-I

$C_{18}H_{20}IN$
M = 377.06 g/mol

9-(6-Iodohexyl)-9H-carbazole (**1r-I**): Synthetized according to the method mentioned in Section 3 with a scale of 10 mmol. Purification by flash column chromatography on silica gel using *n*-pentane:ethyl acetate = 10:1 and obtained as a white solid.

R$_f$ = 0.70 (*n*-pentane: ethyl acetate = 90:10).

M.p.: 67–69 °C.

^{1}H NMR (400 MHz, $CDCl_3$) δ 8.15 (d, *J* = 7.8 Hz, 2H), 7.51 (t, *J* = 7.7 Hz, 2H), 7.45 (d, *J* = 8.2 Hz, 2H), 7.30 – 7.28 (m, 2H), 4.36 (t, *J* = 7.1 Hz, 2H), 3.19 (t, *J* = 6.9 Hz, 2H), 1.98 – 1.91 (m, 2H), 1.86 – 1.79 (m, 2H), 1.50 – 1.43 (m, 4H) ppm.

^{13}C NMR (101 MHz, $CDCl_3$) δ 140.4, 125.6, 122.8, 120.4, 118.8, 108.6, 42.8, 33.2, 30.2, 28.8, 26.2, 6.9 ppm.

IR (ATR): ṽ/cm^{-1} = 3198, 3048, 2928, 2853, 2325, 1885, 1595, 1460, 1378, 1325, 1229, 1166, 1121, 1066, 1020, 925, 843, 749.

HRMS (APCI) for $C_{18}H_{21}IN^+$ $[M+H]^+$: calculated 378.0713, found 378.0709.

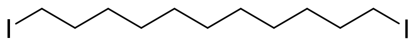

1s-I
$C_{11}H_{22}I_2$
M = 407.98 g/mol

1,11-Diiodoundecane (**1s-I**): Synthetized according to the method mentioned in Section 3 with a scale of 10 mmol. Purification by flash column chromatography on silica gel using *n*-pentane:ethyl acetate = 10:1 and obtained as a colorless oil.

$\mathbf{R_f}$ = 0.85 (*n*-pentane: ethyl acetate = 90:10).

^{1}H NMR (400 MHz, $CDCl_3$) δ 3.19 (t, *J* = 7.0 Hz, 4H), 1.85 – 1.78 (m, 4H), 1.40 – 1.36 (m, 4H), 1.31 – 1.28 (m, 10H) ppm.

^{13}C NMR (101 MHz, $CDCl_3$) δ 33.5, 30.5, 29.4, 29.3, 28.5, 7.3 ppm.

IR (**ATR**): ṽ/cm^{-1} = 2998, 2916, 2845, 2642, 2373, 2303, 2096, 1993, 1845, 1461, 1438, 1322, 1268, 1202, 1156, 1010, 942, 800, 718.

HRMS (**APCI**) for $C_{11}H_{21}I_2^+$ $[M-H]^+$: calculated 406.9727, found 406.9720.

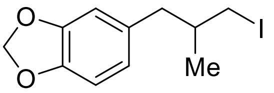

1t-I
$C_{11}H_{13}IO_2$
M = 304.00 g/mol

5-(3-Iodo-2-methylpropyl) benzo [d][1,3]dioxole (**1t-I**): Synthetized according to the method mentioned in Section 3 with a scale of 10 mmol. Purification by flash column chromatography on silica gel using *n*-pentane:ethyl acetate = 10:1 and obtained as a colorless oil.

$\mathbf{R_f}$ = 0.65 (*n*-pentane: ethyl acetate = 90:10).

^{1}H NMR (400 MHz, $CDCl_3$) δ 6.73 (d, *J* = 7.8 Hz, 1H), 6.68 – 6.63 (m, 2H), 5.93 (s, 2H), 3.23 –

3.08 (m, 2H), 2.61 – 2.43 (m, 2H), 1.71 – 1.63 (m, 1H), 1.00 (d, *J* = 6.6 Hz, 3H) ppm.

^{13}C NMR (101 MHz, $CDCl_3$) δ 147.6, 145.9, 133.6, 121.9, 109.3, 108.1, 100.8, 42.1, 36.8, 20.6, 17.1 ppm.

IR (ATR): ṽ/cm^{-1} = 2957, 2880, 2773, 2607, 2294, 2114, 1906, 1846, 1606, 1486, 1439, 1362, 1317, 1244, 1187, 1095, 1036, 922, 863, 803.

HRMS (APCI) for $C_{11}H_{14}IO_2^+$ [M+H]$^+$: calculated 305.0033, found 305.0030.

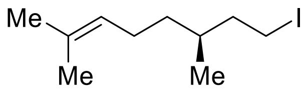

5a-I

$C_{10}H_{19}I$
M = 266.05 g/mol

(*S*)-8-iodo-2,6-dimethyloct-2-ene (**5a-I**): Synthetized according to the method mentioned in Section 3 with a scale of 10 mmol. Purification by flash column chromatography on silica gel using *n*-pentane:ethyl acetate = 10:1 and obtained as a colorless oil.

R$_f$ = 0.90 (*n*-pentane: ethyl acetate = 90:10).

^{1}H NMR (400 MHz, $CDCl_3$) δ 5.09 (t, *J* = 7.1 Hz, 1H), 3.28 – 3.22 (m, 1H), 3.19 – 3.13 (m, 1H), 2.02 – 1.84 (m, 3H), 1.71 – 1.62 (m, 4H), 1.61 (s, 3H), 1.57 – 1.51 (m, 1H), 1.38 – 1.30 (m, 1H), 1.22 – 1.14 (m, 1H), 0.89 (d, *J* = 6.6 Hz, 3H) ppm.

^{13}C NMR (101 MHz, $CDCl_3$) δ 131.5, 124.5, 40.9, 36.3, 33.6, 25.7, 25.3, 18.7, 17.7, 5.2 ppm.

IR (ATR): ṽ/cm^{-1} = 2960, 2913, 2852, 2726, 2665, 2245, 2101, 1886, 1671, 1449, 1376, 1259, 1178, 1080, 983, 884, 810, 734.

HRMS (APCI) for $C_{10}H_{20}I^+$ [M+H]$^+$: calculated 267.0604, found 267.0601.

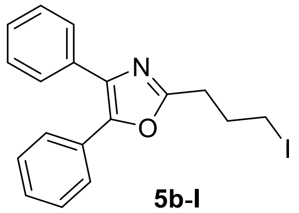

5b-I

$C_{18}H_{16}INO$
M = 389.03 g/mol

2-(3-Iodopropyl)-4,5-diphenyloxazole (**5b-I**): Synthetized according to the method mentioned in Section 3 with a scale of 10 mmol. Purification by flash column chromatography on silica gel using *n*-pentane:ethyl acetate = 10:1 and obtained as a colorless oil.

R_f = 0.45 (*n*-pentane: ethyl acetate = 90:10).

^{1}H NMR (400 MHz, $CDCl_3$) δ 7.64 (d, *J* = 8.4 Hz, 2H), 7.59 (d, *J* = 8.3 Hz, 2H), 7.40 – 7.30 (m, 6H), 3.36 (t, *J* = 6.7 Hz, 2H), 2.99 (t, *J* = 7.2 Hz, 2H), 2.42 – 2.35 (m, 2H) ppm.

^{13}C NMR (101 MHz, $CDCl_3$) δ 161.8, 145.4, 135.1, 132.4, 128.9, 128.6, 128.5, 128.4, 128.1, 127.9, 126.4, 30.5, 29.0, 5.1 ppm.

IR (ATR): ṽ/cm^{-1} = 3054, 2628, 2341, 2102, 1947, 1889, 1805, 1685, 1568, 1500, 1442, 1351, 1206, 1167, 1056, 960, 914, 857, 759, 690.

HRMS (APCI) for $C_{18}H_{17}IO^+$ $[M+H]^+$: calculated 390.0349, found 390.0345.

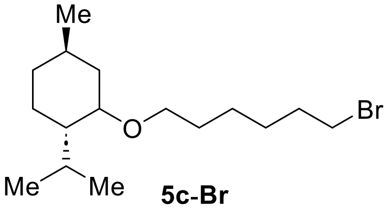

5c-Br

$C_{16}H_{31}BrO$
M = 318.16 g/mol

(1*S*,4*R*)-2-((6-Bromohexyl) oxy)-1-isopropyl-4-methylcyclohexane (**5c-Br**): Synthetized according to the method mentioned in Section 3 with a scale of 10 mmol. Purification by flash column chromatography on silica gel using *n*-pentane:ethyl acetate = 10:1 and obtained as a colorless oil.

R_f = 0.80 (*n*-pentane: ethyl acetate = 90:10).

^{1}H NMR (500 MHz, $CDCl_3$) δ 3.63 – 3.58 (m, 1H), 3.40 (t, *J* = 6.8 Hz, 2H), 3.27 – 3.23 (m, 1H), 3.01 – 2.96 (m, 1H), 2.23 – 2.17 (m, 1H), 2.10 – 2.05 (m, 1H), 1.88 – 1.83 (m, 1H), 1.68 – 1.49(m, 4H), 1.48 – 1.29 (m, 6H), 1.25 – 1.17 (m, 2H), 0.91 – 0.81 (m, 9H), 0.76 (d, *J* = 7.0 Hz, 3H) ppm.

^{13}C NMR (126 MHz, $CDCl_3$) δ 79.2, 68.2, 48.3, 40.5, 34.6, 33.8, 32.8, 31.6, 30.1, 28.0, 25.6, 25.5, 23.4, 22.3, 20.9, 16.2 ppm.

IR (**ATR**): ṽ/cm^{-1} = 2920, 2851, 1459, 1376, 1302, 1247, 1031, 895, 724.

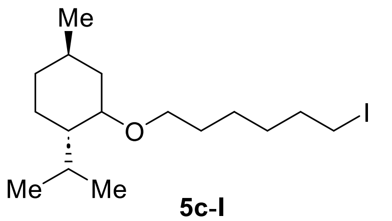

5c-I

$C_{16}H_{31}IO$

M = 366.14 g/mol

(1*S*,4*R*)-2-((6-Iodohexyl) oxy)-1-isopropyl-4-methylcyclohexane (**5c-I**): Synthetized according to the method mentioned in Section 3 with a scale of 10 mmol. Purification by flash column chromatography on silica gel using *n*-pentane:ethyl acetate = 10:1 and obtained as a colorless oil.

R_f = 0.80 (*n*-pentane: ethyl acetate = 90:10).

^{1}H NMR (500 MHz, $CDCl_3$) δ 3.63 – 3.59 (m, 1H), 3.27 – 3.23 (m, 1H), 3.19 (t, *J* = 7.0 Hz, 2H), 3.01 – 2.96 (m, 1H), 2.23 – 2.17 (m, 1H), 2.10 – 2.06 (m, 1H), 1.84 – 1.82 (m, 2H), 1.66 – 1.52 (m, 4H), 1.43 – 1.33 (m, 5H), 1.23 – 1.18 (m, 1H), 0.97 – 0.76 (m, 12H) ppm.

^{13}C NMR (126 MHz, $CDCl_3$) δ 79.2, 68.3, 48.3, 40.5, 34.6, 33.5, 31.6, 30.3, 30.1, 25.6, 25.3, 23.4, 22.4, 20.9, 16.3, 7.0 ppm.

IR (**ATR**): ṽ/cm^{-1} = 2918, 2862, 2331, 2128, 1725, 1453, 1368, 1204, 1168, 1108, 971, 919, 843, 722.

HRMS (APCI) for $C_{16}H_{32}IO^+$ $[M+H]^+$: calculated 367.1492, found 367.1489.

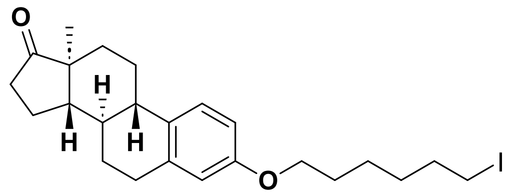

5d-I
$C_{24}H_{33}IO_2$
M = 480.15 g/mol

(8*R*,9*S*,13*S*,14*S*)-3-((6-Iodohexyl) oxy)-13-methyl-6,7,8,9,11,12,13,14,15,16-decahydro-17*H*-cyclopenta[a]phenanthren-17-one (**5d-I**): Synthetized according to the method mentioned in Section 3 with a scale of 10 mmol. Purification by flash column chromatography on silica gel using *n*-pentane:ethyl acetate = 10:1 and obtained as a white solid.

R_f = 0.40 (*n*-pentane: ethyl acetate = 90:10).

M.p.: 84–86 °C.

^{1}H NMR (500 MHz, $CDCl_3$) δ 7.19 (d, *J* = 8.7 Hz, 1H), 6.71 (dd, *J* = 8.6, 2.8 Hz, 1H), 6.64 (s, 1H), 3.93 (t, *J* = 6.4 Hz, 2H), 3.20 (t, *J* = 7.0 Hz, 2H), 2.91 – 2.88 (m, 2H), 2.53 – 2.47 (m, 1H), 2.41 – 2.38 (m, 1H), 2.28 – 2.23 (m, 1H), 2.18 – 2.10 (m, 1H), 2.08 – 2.04 (m, 1H), 2.03 – 1.98 (m, 1H), 1.97 – 1.94 (m, 1H), 1.89 – 1.83 (m, 2H), 1.80 – 1.75 (m, 2H), 1.62 – 1.44 (m, 10H), 0.91 (s, 3H) ppm.

^{13}C NMR (126 MHz, $CDCl_3$) δ 220.8, 157.1, 137.7, 132.0, 126.3, 114.6, 112.1, 67.7, 50.4, 48.0, 44.0, 38.4, 35.8, 33.4, 31.6, 30.2, 29.6, 29.1, 26.6, 25.9, 25.1, 21.6, 13.8, 6.8 ppm.

IR (ATR): ṽ/cm^{-1} = 3457, 2926, 2856, 2341, 1870, 1735, 1607, 1573, 1497, 1471, 1371, 1251, 1160, 1053, 1004, 919, 868, 815, 781, 721.

HRMS (APCI) for $C_{24}H_{33}IO_2^+$ $[M]^+$: calculated 480.1520, found 480.1508.

4.2 Characterization data of nitroalkenes and nitroalkanes

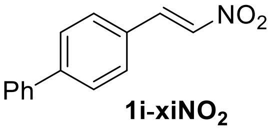

1i-xiNO$_2$
$C_{14}H_{11}NO_2$
M = 225.08 g/mol

(*E*)-4-(2-Nitrovinyl)-1,1'-biphenyl (**1i-xiNO$_2$**): Synthetized according to the method mentioned in Section 3 with a scale of 10 mmol. Purification by flash column chromatography on silica gel using *n*-pentane:ethyl acetate = 10:1 and obtained as a yellow solid.

R$_f$ = 0.40 (*n*-pentane: ethyl acetate = 90:10)

M.p.: 125–127 °C.

^{1}H NMR (400 MHz, $CDCl_3$) δ 8.06 (d, *J* = 13.7 Hz, 1H), 7.69 (d, *J* = 8.4 Hz, 2H), 7.65 – 7.61 (m, 5H), 7.48 (t, *J* = 7.4 Hz, 2H), 7.41 (t, *J* = 7.3 Hz, 1H) ppm.

^{13}C NMR (101 MHz, $CDCl_3$) δ 145.0, 139.5, 138.7, 136.8, 129.7, 129.0, 128.9, 128.4, 128.0, 127.1 ppm.

IR (**ATR**): ṽ/cm^{-1} = 3417, 3109, 3045, 2817, 2290, 2113, 1917, 1708, 1629, 1557, 1493, 1410, 1338, 1262, 1221, 1190, 1123, 1091, 969, 829, 767, 733, 695.

HRMS (APCI) for $C_{14}H_{12}NO_2^+$ $[M+H]^+$: calculated 226.0863, found 226.0862.

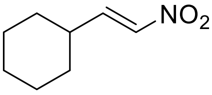

1u-xiNO$_2$
$C_8H_{13}NO_2$
M = 155.09 g/mol

(*E*)-(2-Nitrovinyl) cyclohexane (**1u-xiNO$_2$**): Synthetized according to the method mentioned in Section 3 with a scale of 10 mmol. Purification by flash column chromatography on silica gel using *n*-penane:ethyl acetate = 10:1 and obtained as a yellow oil.

R$_f$ = 0.80 (*n*-pentane: ethyl acetate = 90:10)

^{1}H NMR (500 MHz, $CDCl_3$) δ 7.21 (dd, *J* = 13.5, 7.2 Hz, 1H), 6.92 (d, *J* = 13.5 Hz, 1H), 2.28 – 2.21 (m, 1H), 1.81 – 1.76 (m, 4H), 1.72 – 1.67 (m, 1H), 1.37 – 1.26 (m, 2H), 1.25 – 1.13 (m, 3H) ppm.

^{13}C NMR (126 MHz, $CDCl_3$) δ 147.3, 138.2, 37.5, 31.3, 25.5, 25.4 ppm.

IR (**ATR**): ṽ/cm^{-1} = 3106, 2925, 2853, 2666, 2230, 2021, 1644, 1517, 1447, 1346, 1143, 1101, 959, 915, 882, 835, 755.

HRMS (APCI) for $C_8H_{14}O_2^+$ [M+H]$^+$: calculated 156.1019, found 156.1021.

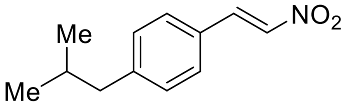

7a-xiNO$_2$
$C_{12}H_{15}NO_2$
M = 205.11 g/mol

(*E*)-1-Isobutyl-4-(2-nitrovinyl) benzene (**7a-xiNO$_2$**): Synthetized according to the method mentioned in Section 3 with a scale of 30 mmol. Purification by flash column chromatography on silica gel using *n*-penane:ethyl acetate = 10:1 and obtained as an orange to brown solid.

R_f = 0.65 (*n*-pentane: ethyl acetate = 90:10)

M.p.: 64–66 °C.

^{1}H NMR (500 MHz, $CDCl_3$) δ 8.00 (d, *J* = 13.6 Hz, 1H), 7.58 (d, *J* = 13.7 Hz, 1H), 7.46 (d, *J* = 8.1 Hz, 2H), 7.23 (d, *J* = 8.1 Hz, 2H), 2.52 (d, *J* = 7.2 Hz, 2H), 1.94 – 1.84 (m, 1H), 0.91 (d, *J* = 6.6 Hz, 6H) ppm.

^{13}C NMR (126 MHz, $CDCl_3$) δ146.9, 139.2, 136.3, 130.2, 129.1, 127.5, 45.4, 30.1, 22.3 ppm.

IR (**ATR**): ṽ/cm^{-1} = 3107, 3038, 2956, 2294, 2114, 2075, 1913, 1697, 1629, 1603, 1557, 1494, 1463, 1334, 1261, 1167, 1119, 964, 829, 723.

HRMS (APCI) for $C_{12}H_{16}O_2^+$ [M+H]$^+$: calculated 206.1176, found 206.1178.

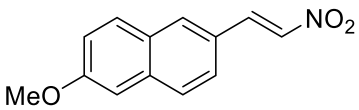

7b-xiNO$_2$

$C_{13}H_{11}NO_3$
M = 229.07 g/mol

(*E*)-2-Methoxy-6-(2-nitrovinyl) naphthalene (**7b-xiNO$_2$**): Synthetized according to the method mentioned in Section 3 with a scale of 30 mmol. Purification by flash column chromatography on silica gel using *n*-penane:ethyl acetate = 10:1 and obtained as an orange to brown solid.

R$_f$ = 0.55 (*n*-pentane: ethyl acetate = 90:10)

M.p.: 164–166 °C.

^{1}H NMR (500 MHz, $CDCl_3$) δ 8.13 (d, *J* = 13.5 Hz, 1H), 7.93 (s, 1H), 7.77 (t, *J* = 9.2 Hz, 2H), 7.67 (d, *J* = 13.6 Hz, 1H), 7.56 (d, *J* = 8.5 Hz, 1H), 7.21 (d, *J* = 11.4 Hz, 1H), 7.15 (s, 1H), 3.95 (s, 3H) ppm.

^{13}C NMR (126 MHz, $CDCl_3$) δ 159.7, 139.6, 136.6, 136.1, 132.2, 130.4, 128.5, 128.1, 125.3, 124.0, 120.0, 106.1, 55.5 ppm.

IR (**ATR**): ṽ/cm^{-1} = 3239, 3113, 2954, 2838, 2644, 2559, 2294, 2225, 2113, 1682, 1618, 1495, 1420, 1392, 1325, 1280, 1177, 1031, 961, 859, 826.

HRMS (APCI) for $C_{13}H_{12}NO_3^+$ $[M+H]^+$: calculated 230.0812, found 230.0817.

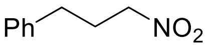

1a

$C_9H_{11}NO_2$
M = 165.08 g/mol

(3-Nitropropyl) benzene (**1a**): Synthetized according to the method mentioned in Section 3 with a scale of 5.0 mmol. Purification by flash column chromatography on silica gel using *n*-pentane:ethyl acetate = 10:1 and obtained as a yellow oil, yield: 73%.

R_f = 0.40 (*n*-pentane: ethyl acetate = 90:10)

^{1}H NMR (500 MHz, $CDCl_3$) δ 7.36 (t, *J* = 7.5 Hz, 2H), 7.27 (t, *J* = 7.2 Hz, 1H), 7.22 (d, *J* = 7.4 Hz, 2H), 4.40 (t, *J* = 6.9 Hz, 2H), 2.77 (t, *J* = 7.5 Hz, 2H), 2.40 – 2.34 (m, 2H) ppm.

^{13}C NMR (126 MHz, $CDCl_3$) δ 139.5, 128.7, 128.4, 126.6, 74.6, 32.2, 28.8 ppm.

IR (ATR): $\tilde{v}$/cm^{-1} = 3027, 2927, 1734, 1545, 1495, 1453, 1380, 1219, 1172, 1044, 984, 911, 876, 822, 745, 698.

HRMS (APCI) for $C_9H_{11}NO_2^+$ [M]$^+$: calculated 165.0784, found 165.0784.

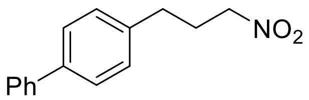

1b
$C_{15}H_{15}NO_2$
M = 241.11 g/mol

4-(3-Nitropropyl)-1,1'-biphenyl (**1b**): Synthetized according to the method mentioned in Section 3 with a scale of 5.0 mmol. Purification by flash column chromatography on silica gel using *n*-pentane:ethyl acetate = 10:1 and obtained as a yellow solid, yield: 66%.

R_f = 0.35 (*n*-pentane: ethyl acetate = 90:10)

M.p.: 52–54 °C.

^{1}H NMR (400 MHz, $CDCl_3$) δ 7.53 – 7.45 (m, 4H), 7.36 (t, *J* = 7.6 Hz, 2H), 7.27 (t, *J* = 7.3 Hz, 1H), 7.20 – 7.16 (m, 2H), 4.33 (t, *J* = 6.8 Hz, 2H), 2.69 (t, *J* = 7.5 Hz, 2H), 2.35 – 2.25 (m, 2H) ppm.

^{13}C NMR (101 MHz, $CDCl_3$) δ 140.7, 139.6, 138.5, 128.9, 128.8, 127.4, 127.2, 127.0, 74.6, 31.8, 28.8 ppm.

IR (ATR): $\tilde{v}$/cm^{-1} = 3029, 2920, 2320, 2110, 1908, 1795, 1711, 1544, 1378, 1287, 1219, 1166, 1066, 1005, 839, 755, 686.

HRMS (APCI) for $C_{15}H_{15}NO_2^+$ [M]$^+$: calculated 241.1097, found 241.1098.

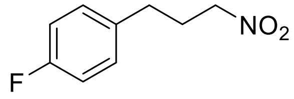

1c

$C_9H_{10}FNO_2$
M = 183.07g/mol

1-Fluoro-4-(3-nitropropyl) benzene (**1c**): Synthetized according to the method mentioned in Section 3 with a scale of 5.0 mmol. Purification by flash column chromatography on silica gel using *n*-pentane:ethyl acetate = 10:1 and obtained as a yellow oil, yield: 77%.

R_f = 0.40 (*n*-pentane: ethyl acetate = 90:10)

^{1}H NMR (400 MHz, $CDCl_3$) δ 7.16 – 7.13 (m, 2H), 7.00 (t, *J* = 8.7 Hz, 2H), 4.36 (t, *J* = 6.9 Hz, 2H), 2.70 (t, *J* = 7.5 Hz, 2H), 2.33 – 2.26 (m, 2H) ppm.

^{13}C NMR (101 MHz, $CDCl_3$) δ 161.55 (d, $J_{C,F}$ = 244.7 Hz), 135.10 (d, $J_{C,F}$ = 3.3 Hz), 129.82 (d, $J_{C,F}$ = 8.0 Hz), 115.43 (d, $J_{C,F}$ = 21.4 Hz), 74.5, 31.4, 28.9 ppm.

^{19}F NMR (471 MHz, $CDCl_3$) δ -116.4 ppm.

IR (**ATR**): ṽ/cm^{-1} = 2929, 2082, 1891, 1601, 1546, 1507, 1433, 1380, 1218, 1158, 1099, 1053, 1015, 821, 767, 702.

HRMS (**APCI**) for $C_9H_{10}FNO_2^+$ $[M]^+$: calculated 183.0690, found 183.0690.

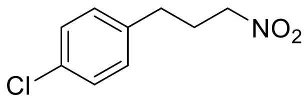

1d

$C_9H_{10}ClNO_2$
M = 199.04 g/mol

1-Chloro-4-(3-nitropropyl) benzene (**1d**): Synthetized according to the method mentioned in Section 3 with a scale of 5.0 mmol. Purification by flash column chromatography on silica gel using *n*-pentane:ethyl acetate = 10:1 and obtained as a yellow oil, yield: 79%.

R$_f$ = 0.40 (*n*-pentane: ethyl acetate = 90:10)

^{1}H NMR (400 MHz, $CDCl_3$) δ 7.30 (d, *J* = 8.2 Hz, 2H), 7.14 (d, *J* = 8.1 Hz, 2H), 4.38 (t, *J* = 6.9 Hz, 2H), 2.71 (t, *J* = 7.6 Hz, 2H), 2.33 – 2.30 (m, 2H) ppm.

^{13}C NMR (101 MHz, $CDCl_3$) δ 137.9, 132.2, 129.7, 129.7, 128.7, 74.4, 31.4, 28.5 ppm.

IR (**ATR**): ṽ/cm^{-1} = 2924, 2854, 1544, 1433, 1380, 1197, 1148, 1074, 875, 722.

HRMS (**APCI**) for $C_9H_{11}ClNO_2^+$ [M+H]$^+$: calculated 200.0473, found 200.0471.

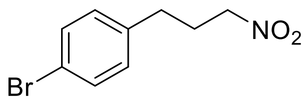

1e

$C_9H_{10}BrNO_2$
M = 242.99 g/mol

1-Bromo-4-(3-nitropropyl) benzene (**1e**): Synthetized according to the method mentioned in Section 3 with a scale of 5.0 mmol. Purification by flash column chromatography on silica gel using *n*-pentane:ethyl acetate = 10:1 and obtained as a yellow oil, yield: 81%.

R$_f$ = 0.40 (*n*-pentane: ethyl acetate = 90:10)

^{1}H NMR (400 MHz, $CDCl_3$) δ 7.43 (d, *J* = 8.3 Hz, 2H), 7.06 (d, *J* = 8.3 Hz, 2H), 4.36 (t, *J* = 6.8 Hz, 2H), 2.68 (t, *J* = 7.5 Hz, 2H), 2.34 – 2.26 (m, 2H) ppm.

^{13}C NMR (101 MHz, $CDCl_3$) δ 138.4, 131.8, 130.2, 120.4, 74.4, 31.6, 28.6 ppm.

IR (**ATR**): ṽ/cm^{-1} = 3024, 2926, 2289, 2199, 2084, 1989, 1898, 1545, 1486, 1431, 1379, 1267, 1170, 1105, 1071, 1010, 834, 797, 709.

HRMS (**APCI**) for $C_9H_{10}BrNO_2^+$ [M]$^+$: calculated 242.9884, found 242.9887.

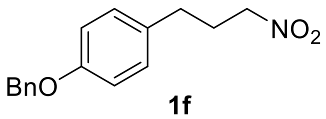

1f

$C_{16}H_{17}NO_3$
M = 271.12 g/mol

1-(Benzyloxy)-4-(3-nitropropyl) benzene (**1f**): Synthetized according to the method mentioned in Section 3 with a scale of 5.0 mmol. Purification by flash column chromatography on silica gel using *n*-pentane:ethyl acetate = 10:1 and obtained as a yellow solid, yield: 72%.

$\mathbf{R_f}$ = 0.30 (*n*-pentane: ethyl acetate = 90:10)

M.p.: 74–76 °C.

^{1}H NMR (400 MHz, $CDCl_3$) δ 7.44 – 7.37 (m, 4H), 7.33 (t, *J* = 7.1 Hz, 1H), 7.10 (d, *J* = 8.6 Hz, 2H), 6.93 (d, *J* = 8.6 Hz, 2H), 5.05 (s, 2H), 4.36 (t, *J* = 6.9 Hz, 2H), 2.67 (t, *J* = 7.4 Hz, 2H), 2.33 – 2.26 (m, 2H) ppm.

^{13}C NMR (101 MHz, $CDCl_3$) δ 157.5, 137.0, 131.7, 129.4, 128.6, 127.9, 127.4, 115.0, 74.6, 70.0, 31.3, 29.0 ppm.

IR (**ATR**): ṽ/cm^{-1} = 3033, 2929, 2858, 2468, 2318, 1891, 1717, 1541, 1454, 1382, 1313, 1239, 1174, 1040, 908, 833, 782, 735, 712.

HRMS (**APCI**) for $C_{16}H_{17}NO_3^+$ $[M]^+$: calculated 271.1203, found 271.1203.

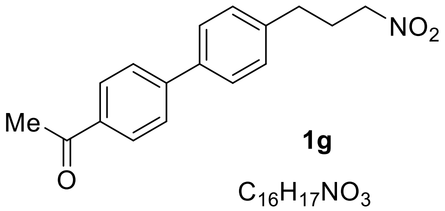

1g

$C_{16}H_{17}NO_3$
M = 271.12 g/mol

1-(4'-(3-Nitropropyl)-[1,1'-biphenyl]-4-yl) ethan-1-one (**1g**): Synthetized according to the method mentioned in Section 3 with a scale of 5.0 mmol. Purification by flash column chromatography on silica gel using *n*-pentane:ethyl acetate = 5:1 and obtained as a white solid, yield: 47%.

$\mathbf{R_f}$ = 0.45 (*n*-pentane: ethyl acetate = 40:10)

M.p.: 99–101 °C.

^{1}H NMR (500 MHz, $CDCl_3$) δ 8.03 (d, *J* = 8.3 Hz, 2H), 7.67 (d, *J* = 8.3 Hz, 2H), 7.59 (d, *J* =

8.1 Hz, 2H), 7.29 (d, *J* = 7.9 Hz, 2H), 4.41 (t, *J* = 6.9 Hz, 2H), 2.79 (t, *J* = 7.5 Hz, 2H), 2.64 (s, 3H), 2.40 – 2.35 (m, 2H).

^{13}C NMR (126 MHz, $CDCl_3$) δ 197.7, 145.3, 139.7, 138.2, 135.8, 129.1, 128.9, 127.6, 127.0, 74.6, 31.9, 28.7, 26.6.

IR (**ATR**): ṽ/cm^{-1} = 3349, 3018, 2918, 2321, 2108, 1909, 1800, 1679, 1599, 1550, 1433, 1360, 1263, 1167, 1002, 959, 878, 806, 719.

HRMS (APCI) for $C_{17}H_{18}NO_3^+$ $[M+H]^+$: calculated 284.1281, found 284.1279.

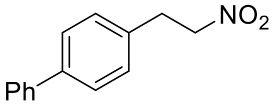

1i
$C_{14}H_{13}NO_2$
M = 227.09 g/mol

(4-Nitropropyl) benzene (**1i**): Synthetized according to the method mentioned in Section 3 with a scale of 1.0 mmol. Purification by flash column chromatography on silica gel using *n*-pentane:ethyl acetate = 10:1 and obtained as a yellow solid, yield: 97%.

R$_f$ = 0.35 (*n*-pentane: ethyl acetate = 90:10)

M.p.: 55–57 °C.

^{1}H NMR (500 MHz, $CDCl_3$) δ 7.58 – 7.55 (m, 4H), 7.44 (t, *J* = 7.5 Hz, 2H), 7.36 (t, *J* = 7.3 Hz, 1H), 7.29 (d, *J* = 7.8 Hz, 2H), 4.65 (t, *J* = 7.4 Hz, 2H), 3.37 (t, *J* = 7.4 Hz, 2H) ppm.

^{13}C NMR (126 MHz, $CDCl_3$) δ 140.5, 140.5, 134.6, 129.0, 128.8, 127.7, 127.4, 127.0, 76.2, 33.1 ppm.

IR (**ATR**): ṽ/cm^{-1} = 3419, 3029, 2924, 2852, 2236, 2114, 1905, 1710, 1544, 1486, 1432, 1375, 1219, 1176, 1118, 1076, 1040, 1006, 968, 913, 867, 830, 762, 695.

HRMS (APCI) for $C_{14}H_{12}NO_2^+$ $[M-H]^+$: calculated 226.0863, found 226.0863.

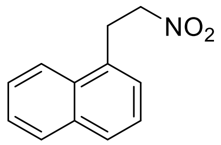

1j

$C_{12}H_{11}NO_2$
M = 201.08 g/ mol

1-(2-Nitroethyl) naphthalene (**1j**): Synthetized according to the method mentioned in Section 3 with a scale of 5.0 mmol. Purification by flash column chromatography on silica gel using *n*-pentane:ethyl acetate = 10:1 and obtained as a yellow oil, yield: 71%.

R_f = 0.35 (*n*-pentane: ethyl acetate = 90:10)

^{1}H NMR (400 MHz, $CDCl_3$) δ 7.99 (d, *J* = 8.8 Hz, 1H), 7.92 (d, *J* = 7.0 Hz, 1H), 7.82 (d, *J* = 8.2 Hz, 1H), 7.62 – 7.53 (m, 2H), 7.45 – 7.42 (m, 1H), 7.37 (d, *J* = 7.0 Hz, 1H), 4.74 – 4.70 (m, 2H), 3.80 (t, *J* = 7.7 Hz, 2H) ppm.

^{13}C NMR (101 MHz, $CDCl_3$) δ 133.8, 131.3, 131.2, 129.1, 128.2, 127.0, 126.7, 125.9, 125.5, 122.5, 75.3, 30.6 ppm.

IR (**ATR**): ṽ/cm^{-1} = 3047, 2651, 2284, 2113, 1930, 1810, 1596, 1544, 1430, 1375, 1259, 1185, 1079, 1024, 969, 907, 871, 773, 689.

HRMS (APCI) for $C_{12}H_{11}^+$ $[M-NO_2]^+$: calculated 155.0855, found 155.0854.

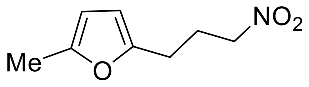

1k

$C_8H_{11}NO_3$
M = 169.07 g/mol

2-Methyl-5-(3-nitropropyl) furan (**1k**): Synthetized according to the method mentioned in Section 3 with a scale of 5.0 mmol. Purification by flash column chromatography on silica gel using *n*-pentane:ethyl acetate = 10:1 and obtained as a yellow oil, yield: 65%.

R_f = 0.30 (*n*-pentane: ethyl acetate = 90:10)

^{1}**H NMR** (400 MHz, $CDCl_3$) δ 5.92 (d, *J* = 3.0 Hz, 1H), 5.86 (d, *J* = 1.3 Hz, 1H), 4.41 (t, *J* = 6.9 Hz, 2H), 2.71 (t, *J* = 7.1 Hz, 2H), 2.36 – 2.29 (m, 2H), 2.25 (s, 3H) ppm.

^{13}**C NMR** (101 MHz, $CDCl_3$) δ 151.1, 151.1, 106.9, 105.9, 74.5, 25.9, 24.7, 13.5 ppm.

IR (**ATR**): ṽ/cm^{-1} = 3104, 2923, 2104, 1546, 1432, 1380, 1277, 1216, 1019, 938, 882, 781.

HRMS (**APCI**) for $C_8H_{10}NO_3^+$ [M-H]$^+$: calculated 168.0655, found 168.0654.

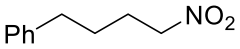

1l
$C_{10}H_{13}NO_2$
M = 179.09 g/mol

(4-Nitrobutyl) benzene (**1l**): Synthetized according to the method mentioned in Section 3 with a scale of 5.0 mmol. Purification by flash column chromatography on silica gel using *n*-pentane:ethyl acetate = 10:1 and obtained as a yellow oil, yield: 79%.

R_f = 0.40 (*n*-pentane: ethyl acetate = 90:10)

^{1}**H NMR** (400 MHz, $CDCl_3$) δ 7.30 (t, *J* = 7.3 Hz, 2H), 7.23 – 7.16 (m, 3H), 4.38 (t, *J* = 7.0 Hz, 2H), 2.68 (t, *J* = 7.6 Hz, 2H), 2.08 – 2.00 (m, 2H), 1.77 – 1.69 (m, 2H) ppm.

^{13}**C NMR** (101 MHz, $CDCl_3$) δ 141.0, 128.5, 128.3, 126.1, 75.4, 34.9, 27.8, 26.8 ppm.

IR (ATR): ṽ/cm^{-1} = 3026, 2926, 2860, 1949, 1545, 1494, 1453, 1380, 1210, 1163, 1091, 1029, 908, 838, 804, 746, 698.

HRMS (APCI) for $C_{10}H_{12}NO_2^+$ [M-H]$^+$: calculated 178.0863, found 178.0863.

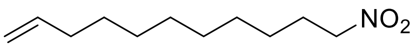

1m
$C_{11}H_{21}NO_2$
M = 199.16 g/mol

11-Nitroundec-1-ene (**1m**): Synthetized according to the method mentioned in Section 3 with a scale of 5.0 mmol. Purification by flash column chromatography on silica gel using *n*-pentane:ethyl acetate = 10:1 and obtained as a yellow oil, yield: 74%.

R$_f$ = 0.40 (*n*-pentane: ethyl acetate = 90:10)

^{1}H NMR (400 MHz, $CDCl_3$) δ 5.85 – 5.73 (m, 1H), 5.00 – 4.90 (m, 2H), 4.37 (t, *J* = 7.0 Hz, 2H), 2.05 – 1.96 (m, 4H), 1.38 – 1.27 (m, 12H) ppm.

^{13}C NMR (101 MHz, $CDCl_3$) δ 139.1, 114.1, 75.7, 33.7, 29.2, 29.1, 29.0, 28.8, 28.8, 27.3, 26.1 ppm.

IR (ATR): ṽ/cm^{-1} = 3075, 2924, 2854, 1639, 1549, 1434, 1381, 1207, 993, 908, 722.

HRMS (APCI) for $C_{11}H_{22}NO_2^+$ [M+H]$^+$: calculated 200.1645, found 200.1645.

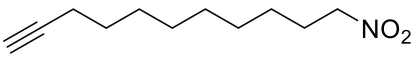

1n
$C_{11}H_{19}NO_2$
M = 197.14 g/mol

11-Nitroundec-1-yne (**1n**): Synthetized according to the method mentioned in Section 3 with a scale of 5.0 mmol. Purification by flash column chromatography on silica gel using *n*-pentane:ethyl acetate = 10:1 and obtained as a yellow oil, yield: 76%.

R$_f$ = 0.40 (*n*-pentane: ethyl acetate = 90:10)

^{1}H NMR (400 MHz, $CDCl_3$) δ 4.36 (t, *J* = 7.0 Hz, 2H), 2.18 – 2.14 (m, 2H), 2.03 – 1.97 (m, 2H), 1.92 (s, 1H), 1.54 – 1.47 (m, 2H), 1.39 – 1.27 (m, 10H) ppm.

^{13}C NMR (101 MHz, $CDCl_3$) δ 84.6, 75.6, 68.1, 29.0, 28.8, 28.7, 28.5, 28.3, 27.3, 26.1, 18.3 ppm.

IR (**ATR**): ṽ/cm^{-1} = 3297, 2927, 2855, 2115, 1724, 1548, 1433, 1381, 1257, 1053, 872, 723.

HRMS (**APCI**) for $C_{11}H_{20}NO_2^+$ [M+H]$^+$: calculated 198.1489, found 198.1488.

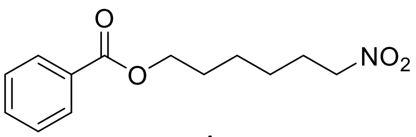

1o

$C_{13}H_{17}NO_4$
M = 251.12 g/mol

6-Nitrohexyl benzoate (**1o**): Synthetized according to the method mentioned in Section 3 with a scale of 5.0 mmol. Purification by flash column chromatography on silica gel using *n*-pentane:ethyl acetate = 5:1 and obtained as a yellow oil, yield: 60%.

R_f = 0.60 (*n*-pentane: ethyl acetate = 40:10)

^{1}H NMR (400 MHz, $CDCl_3$) δ 8.03 (d, *J* = 6.9 Hz, 2H), 7.55 (t, *J* = 7.4 Hz, 1H), 7.44 (t, *J* = 7.6 Hz, 2H), 4.38 (t, *J* = 7.0 Hz, 2H), 4.31 (t, *J* = 6.5 Hz, 2H), 2.07 – 2.00 (m, 2H), 1.82 – 1.75 (m, 2H), 1.55 – 1.42 (m, 4H) ppm.

^{13}C NMR (101 MHz, $CDCl_3$) δ 166.5, 132.9, 130.3, 129.4, 128.3, 75.5, 64.5, 28.4, 27.2, 25.9, 25.4 ppm.

IR (**ATR**): ṽ/cm^{-1} = 3421, 3065, 2937, 2861, 2338, 2093, 1912, 1713, 1546, 1451, 1381, 1269, 1175, 1109, 1025, 961, 902, 806, 709.

HRMS (**APCI**) for $C_{13}H_{18}NO_4^+$ [M+H]$^+$: calculated 252.1230, found 252.1230.

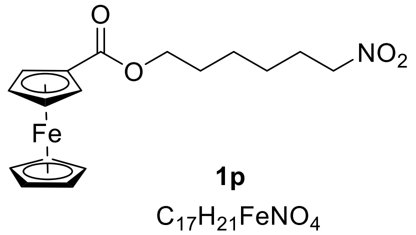

1p

$C_{17}H_{21}FeNO_4$
M = 359.08 g/mol

6-Nitrohexyl ferrocene carboxylate (**1p**): Synthetized according to the method mentioned in Section 3 with a scale of 5.0 mmol. Purification by flash column chromatography on silica gel using *n*-pentane:ethyl acetate = 5:1 and obtained as a yellow oil, yield: 61%.

R_f = 0.40 (*n*-pentane: ethyl acetate = 40:10)

^{1}H NMR (400 MHz, $CDCl_3$) δ 4.80 (t, *J* = 2.0 Hz, 2H), 4.42 – 4.39 (m, 4H), 4.23 – 4.18 (m, 6H), 2.09 – 2.02 (m, 3H), 1.76 – 1.71 (m, 2H), 1.50 – 1.47 (m, 4H) ppm.

^{13}C NMR (101 MHz, $CDCl_3$) δ 171.7, 75.5, 71.2, 70.1, 69.7, 63.8, 28.6, 27.3, 26.0, 25.4 ppm.

IR (**ATR**): ṽ/cm^{-1} = 3418, 3095, 2934, 2860, 2737, 2341, 2109, 2091, 1887, 1702, 1546, 1457, 1374, 1271, 1133, 1024, 962, 923, 821, 773, 731.

HRMS (**APCI**) for $C_{17}H_{22}FeNO_4^+$ $[M+H]^+$: calculated 360.0887, found 360.0890.

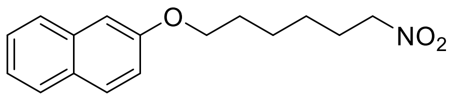

1q
$C_{16}H_{19}NO_3$
M = 273.14 g/mol

2-((6-Nitrohexyl) oxy) naphthalene (**1q**): Synthetized according to the method mentioned in Section 3 with a scale of 5.0 mmol. Purification by flash column chromatography on silica gel using *n*-pentane:ethyl acetate = 10:1 and obtained as a yellow oil, yield: 61%.

R_f = 0.30 (*n*-pentane: ethyl acetate = 90:10)

^{1}H NMR (400 MHz, $CDCl_3$) δ 7.79 – 7.74 (m, 3H), 7.46 (t, *J* = 6.9 Hz, 1H), 7.37 – 7.33 (m, 1H), 7.18 – 7.14 (m, 2H), 4.37 (t, *J* = 7.0 Hz, 2H), 4.07 (t, *J* = 6.3 Hz, 2H), 2.07 – 2.02 (m, 2H), 1.89 – 1.82 (m, 2H), 1.60 – 1.53 (m, 2H), 1.50 – 1.42 (m, 2H) ppm.

^{13}C NMR (101 MHz, $CDCl_3$) δ 156.8, 134.5, 129.3, 128.8, 127.5, 126.6, 126.3, 123.5, 118.8, 106.4, 75.5, 67.4, 28.8, 27.2, 25.9, 25.4 ppm.

IR (**ATR**): ṽ/cm^{-1} = 3056, 2929, 2860, 2299, 2110, 1907, 1627, 1598, 1545, 1509, 1462, 1433, 1385, 1256, 1214, 1178, 1118, 1015, 978, 836, 746.

HRMS (**APCI**) for $C_{16}H_{19}NO_3^+$ $[M]^+$: calculated 273.1359, found 273.1360.

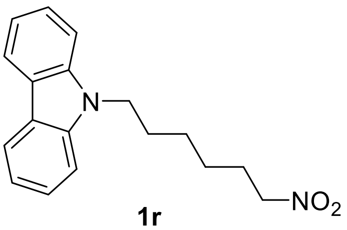

1r

$C_{18}H_{20}N_2O_2$
M = 296.15 g/mol

9-(6-Nitrohexyl)-9H-carbazole (**1r**): Synthetized according to the method mentioned in Section 3 with a scale of 5.0 mmol. Purification by flash column chromatography on silica gel using *n*-pentane:ethyl acetate = 5:1 and obtained as a yellow oil, yield: 73%.

R_f = 0.30 (*n*-pentane: ethyl acetate = 90:10)

^{1}H NMR (400 MHz, $CDCl_3$) δ 7.97 (d, *J* = 7.7 Hz, 2H), 7.33 (t, *J* = 7.8 Hz, 2H), 7.22 (d, *J* = 8.2 Hz, 1H), 7.11 (t, *J* = 7.4 Hz, 1H), 4.11 – 4.06 (m, 2H), 1.75 – 1.65 (m, 2H), 1.20 – 1.14 (m, 2H) ppm.

^{13}C NMR (101 MHz, $CDCl_3$) δ 140.2, 125.6, 122.7, 120.2, 118.7, 108.5, 75.3, 42.5, 28.5, 26.9, 26.4, 25.9 ppm.

IR (**ATR**): ṽ/cm^{-1} = 3049, 2927, 2857, 2644, 2282, 2097, 1891, 1774, 1594, 1545, 1451, 1378, 1323, 1221, 1152, 1120, 1063, 1020, 907, 846, 721.

HRMS (**APCI**) for $C_{18}H_{21}N_2O_2^+$ [M+H]$^+$: calculated 297.1598, found 297.1594.

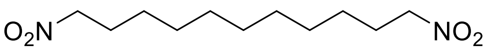

1s

$C_{11}H_{22}N_2O_4$
M = 246.16 g/mol

1,11-Dinitroundecane (**1s**): Synthetized according to the method mentioned in Section 3 with a scale of 5.0 mmol. Purification by flash column chromatography on silica gel using *n*-pentane:ethyl acetate = 4:1 and obtained as a yellow oil, yield: 45%.

R_f = 0.40 (*n*-pentane: ethyl acetate = 40:10)

^{1}H NMR (400 MHz, $CDCl_3$) δ 4.37 (t, *J* = 7.0 Hz, 4H), 2.04 – 1.96 (m, 4H), 1.41 – 1.28 (m, 14H) ppm.

^{13}C NMR (101 MHz, $CDCl_3$) δ 75.7, 29.2, 29.1, 28.7, 27.3, 26.1 ppm.

IR (**ATR**): ṽ/cm^{-1} = 2924, 2854, 1544, 1433, 1380, 1197, 1148, 1074, 875, 722.

HRMS (**APCI**) for $C_{11}H_{23}N_2O_4^+$ [M+H]$^+$: calculated 247.1652, found 247.1652.

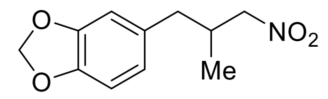

1t
$C_{11}H_{13}NO_4$
M = 223.08 g/mol

5-(2-Methyl-3-nitropropyl) benzo[d] [1,3] dioxole (**1t**): Synthetized according to the method mentioned in Section 3 with a scale of 5.0 mmol. Purification by flash column chromatography on silica gel using *n*-pentane:ethyl acetate = 5:1 and obtained as a yellow oil, yield: 58%.

R_f = 0.60 (*n*-pentane: ethyl acetate = 40:10)

^{1}H NMR (400 MHz, $CDCl_3$) δ 6.74 (d, *J* = 7.9 Hz, 1H), 6.66 (s, 1H), 6.61 (d, *J* = 7.9 Hz, 1H), 5.94 (s, 2H), 4.33 – 4.28 (m, 1H), 4.20 – 4.15 (m, 1H), 2.59 – 2.52 (m, 3H), 1.02 (d, *J* = 6.6 Hz, 3H) ppm.

^{13}C NMR (101 MHz, $CDCl_3$) δ 147.8, 146.3, 132.0, 122.0, 109.3, 108.3, 100.9, 80.6, 39.7, 34.7, 17.1 ppm.

IR (**ATR**): ṽ/cm^{-1} = 2915, 2777, 2612, 2441, 2306, 2119, 1851, 1747, 1544, 1486, 1439, 1380, 1245, 1188, 1099, 1034, 925, 865, 805, 771, 722.

HRMS (**APCI**) for $C_{11}H_{13}NO_4^+$ [M]$^+$: calculated 223.0839, found 223.0840.

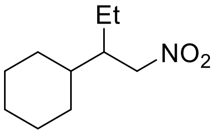

1u-rac

$C_{10}H_{19}NO_2$
M= 185.14 g/mol

(1-Nitrobutan-2-yl) cyclohexane (**1u-rac**): Synthetized according to the method mentioned in Section 3 with a scale of 5.0 mmol. Purification by flash column chromatography on silica gel using *n*-pentane:ethyl acetate = 10:1 and obtained as a yellow oli, yield: 64%.

$\mathbf{R_f}$ = 0.80 (*n*-pentane: ethyl acetate = 90:10)

^{1}H NMR (400 MHz, $CDCl_3$) δ 4.40 (dd, *J* = 12.0, 6.4 Hz, 1H), 4.26 (dd, *J* = 12.0, 7.6 Hz, 1H), 2.07 – 1.99 (m, 1H), 1.77 – 1.73 (m, 2H), 1.69 – 1.60 (m, 3H), 1.51 – 1.38 (m, 2H), 1.34 – 0.95 (m, 6H), 0.92 (t, *J* = 7.5 Hz, 3H) ppm.

^{13}C NMR (101 MHz, $CDCl_3$) δ 44.4, 38.4, 29.6, 29.3, 26.5, 26.4, 21.4, 11.3 ppm.

IR (**ATR**): ṽ/cm^{-1} = 2923, 2852, 1546, 1447, 1381, 1215, 1139, 1031, 983, 891, 780, 732.

HRMS (**APCI**) $C_{10}H_{20}NO_2^+$ [M+H]$^+$: calculated 186.1489, found 186.1489.

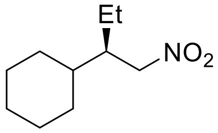

1u

$C_{10}H_{19}NO_2$
M= 185.14 g/mol

(*R*)- (1-Nitrobutan-2-yl) cyclohexane (**1u**): Synthetized according to the method mentioned in Section 3 with a scale of 5.0 mmol. Purification by flash column chromatography on silica gel using *n*-pentane:ethyl acetate = 10:1 and obtained as a yellow oli, yield: 41%.

$\mathbf{R_f}$ = 0.80 (*n*-pentane: ethyl acetate = 90:10)

^{1}H NMR (400 MHz, $CDCl_3$) δ 4.40 (dd, *J* = 12.0, 6.4 Hz, 1H), 4.26 (dd, *J* = 12.0, 7.6 Hz, 1H),

2.07 – 1.99 (m, 1H), 1.77 – 1.73 (m, 2H), 1.69 – 1.60 (m, 3H), 1.51 – 1.38 (m, 2H), 1.34 – 0.95 (m, 6H), 0.92 (t, *J* = 7.5 Hz, 3H) ppm.

^{13}C NMR (101 MHz, $CDCl_3$) δ 44.4, 38.4, 29.6, 29.3, 26.5, 26.4, 21.4, 11.3 ppm.

IR (**ATR**): ṽ/cm^{-1} = 2924, 2852, 2177, 1783, 1724, 1546, 1447, 1381, 1217, 1157, 1032, 983, 891, 731.

HRMS (**APCI**) $C_{10}H_{20}NO_2^+$ [M]$^+$: calculated 186.1489, found 186.1490.

The enantiomeric ratio of **9a** was determined by chiral GLC analysis (Sigma-Aldrich Astec Chiraldex B-DM column (30 m × 0.25 mm, 0.12 μm film thickness), column temperature 60 °C (30 min), ramp of 0.2 °C/min to 175 °C (20 min), flow rate isobaric 14 psi): t_R = 213.4 min (major), t_R = 217.1 min (minor).

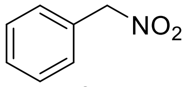

3a

$C_7H_7NO_2$
M = 137.14 g/mol

(Nitromethyl)benzene (**3a**): Synthetized according to the method mentioned in Section 3 with a scale of 10 mmol. Purification by flash column chromatography on silica gel using *n*-pentane:ethyl acetate = 10:1 and obtained as a yellow oil, yield: 97%.

R_f = 0.50 (*n*-pentane: ethyl acetate = 90:10)

^{1}H NMR (400 MHz, $CDCl_3$) δ7.48 – 7.42 (m, 5H), 5.44 (s, 2H) ppm.

^{13}C NMR (101 MHz, $CDCl_3$) δ 130.0, 129.9, 129.7, 129.1, 80.0 ppm.

IR (**ATR**): ṽ/cm^{-1} = 2913, 1545, 1454, 1431, 1371, 1311, 1189, 1078, 1029, 925, 891, 848, 768.

HRMS (**APCI**) for $C_{17}H_8NO_2^+$ [M+H]$^+$: calculated 138.0550, found 138.0546.

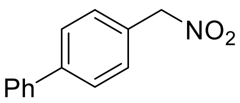

3b

$C_{13}H_{11}NO_2$
M = 213.24 g/mol

4-(Nitromethyl)-1,1'-biphenyl (**3b**): Synthetized according to the method mentioned in Section 3 with a scale of 10 mmol. Purification by flash column chromatography on silica gel using *n*-pentane:ethyl acetate = 10:1 and obtained as a white solid, yield: 83%.

R_f = 0.50 (*n*-pentane: ethyl acetate = 90:10)

M.p.: 98–100 °C.

^{1}H NMR (400 MHz, $CDCl_3$) δ 7.66 (d, *J* = 8.0 Hz, 2H), 7.60 (d, *J* = 7.0 Hz, 2H), 7.54 (d, *J* = 7.9 Hz, 2H), 7.47 (t, *J* = 7.6 Hz, 2H), 7.39 (t, *J* = 7.3 Hz, 1H), 5.49 (s, 2H) ppm.

^{13}C NMR (101 MHz, $CDCl_3$) δ143.0, 140.0, 130.4, 128.9, 128.5, 127.9, 127.8, 127.2, 79.7 ppm.

IR (**ATR**): ṽ/cm^{-1} = 3030, 2908, 2340, 2110, 1916, 1699, 1599, 1540, 1485, 1408, 1370, 1309, 1182, 1124, 1076, 1006, 966, 898, 825, 750, 695.

HRMS (**APCI**) for $C_{13}H_{12}NO_2^+$ $[M+H]^+$: calculated 214.0863, found 214.0858.

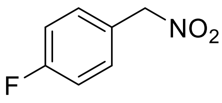

3c

$C_7H_6FNO_2$
M = 155.13 g/mol

1-Fluoro-4-(nitromethyl) benzene (**3c**): Synthetized according to the method mentioned in Section 3 with a scale of 10 mmol. Purification by flash column chromatography on silica gel using *n*-pentane:ethyl acetate = 10:1 and obtained as a yellow oil, yield: 86%.

R_f = 0.50 (*n*-pentane: ethyl acetate = 90:10)

^{1}H NMR (400 MHz, $CDCl_3$) δ 7.47 – 7.44 (m, 2H), 7.12 (t, *J* = 8.6 Hz, 2H), 5.41 (s, 2H) ppm.

^{13}**C NMR** (101 MHz, $CDCl_3$) δ 163.65 (d, $J_{C,F}$ = 250.1 Hz), 132.07 (d, $J_{C,F}$ = 8.7 Hz), 125.62 (d, $J_{C,F}$ = 3.2 Hz), 116.18 (d, $J_{C,F}$ = 21.9 Hz), 79.1 ppm.

^{19}**F NMR** (471 MHz, $CDCl_3$) δ -110.5 ppm.

IR (**ATR**): ṽ/cm^{-1} = 3081, 2915, 2311, 1899, 1731, 1548, 1509, 1432, 1370, 1313, 1224, 1189, 1099, 1045, 963, 865, 834, 792, 727, 678.

HRMS (**APCI**) for $C_7H_7FNO_2^+$ $[M+H]^+$: calculated 156.0455, found 156.0452.

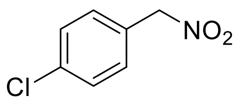

3d

$C_7H_6ClNO_2$
M = 171.58 g/mol

1-Chloro-4-(nitromethyl) benzene (**3d**): Synthetized according to the method mentioned in Section 3 with a scale of 10 mmol. Purification by flash column chromatography on silica gel using *n*-pentane:ethyl acetate = 10:1 and obtained as a white solid, yield: 92%.

R$_f$ = 0.50 (*n*-pentane: ethyl acetate = 90:10)

M.p.: 34–36 °C.

^{1}**H NMR** (400 MHz, $CDCl_3$) δ 7.42 – 7.38 (m, 4H), 5.40 (s, 2H) ppm.

^{13}**C NMR** (101 MHz, $CDCl_3$) δ 164.6, 162.7, 132.1, 132.0, 125.6, 125.6, 116.3, 116.1, 79.1 ppm.

IR (**ATR**): ṽ/cm^{-1} = 3589, 2915, 2732, 2293, 2111, 2074, 1992, 1906, 1788, 1547, 1491, 1369, 1308, 1185, 1089, 1016, 965, 895, 860, 810, 757.

HRMS (**APCI**) for $C_7H_7ClNO_2^+$ $[M+H]^+$: calculated 172.0160, found 172.0156.

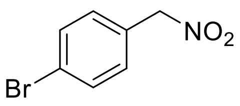

3e

$C_7H_6BrNO_2$
M = 214.96 g/mol

1-Bromo-4-(nitromethyl) benzene (**3e**): Synthetized according to the method mentioned in Section 3 with a scale of 10 mmol. Purification by flash column chromatography on silica gel using *n*-pentane:ethyl acetate = 10:1 and obtained as a white solid, yield: 84%.

R_f = 0.50 (*n*-pentane: ethyl acetate = 90:10)

M.p.: 56–59 °C.

^{1}H NMR (400 MHz, $CDCl_3$) δ 7.57 (d, *J* = 8.2 Hz, 2H), 7.33 (d, *J* = 8.2 Hz, 2H), 5.39 (s, 2H) ppm.
^{13}C NMR (101 MHz, $CDCl_3$) δ 132.3, 131.6, 128.5, 124.5, 79.2 ppm.

IR (**ATR**): ṽ/cm^{-1} = 3328, 3060, 2913, 2360, 2162, 1978, 1551, 1488, 1431, 1370, 1311, 1185, 1070, 1014, 896, 859, 808, 753, 679.

HRMS (**APCI**) for $C_7H_7BrNO_2^+$ $[M+H]^+$: calculated 215.9655, found 215.9651.

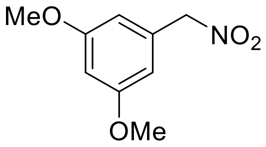

3f

$C_9H_{11}NO_4$
M = 197.19 g/mol

1,3-Dimethoxy-5-(nitromethyl) benzene (**3f**): Synthetized according to the method mentioned in Section 3 with a scale of 10 mmol. Purification by flash column chromatography on silica gel using *n*-pentane:ethyl acetate = 10:1 and obtained as a white solid, yield: 61%.

R_f = 0.50 (*n*-pentane: ethyl acetate = 90:10)

M.p.: 59–61 °C.

^{1}H NMR (400 MHz, $CDCl_3$) δ 6.58 (s, 2H), 6.51 (s, 1H), 5.36 (s, 2H), 3.80 (s, 7H) ppm.

^{13}C NMR (101 MHz, $CDCl_3$) δ 161.1, 131.4, 107.9, 107.7, 101.9, 101.7, 80.1, 55.5, 55.4 ppm.

IR (**ATR**): ṽ/cm^{-1} = 2944, 2842, 2643, 2375, 2209, 2120, 1738, 1597, 1538, 1457, 1349, 1293, 1205, 1150, 1053, 958, 916, 834, 751.

HRMS (**APCI**) for $C_9H_{12}NO_4^+$ [M+H]$^+$: calculated 198.0761, found 198.0757.

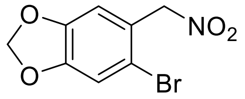

3g

$C_8H_6BrNO_4$
M = 260.04 g/mol

5-Bromo-6-(nitromethyl) benzo [*d*] [1,3] dioxole (**3g**): Synthetized according to the method mentioned in Section 3 with a scale of 10 mmol. Purification by flash column chromatography on silica gel using *n*-pentane:ethyl acetate = 10:1 and obtained as a white solid, yield: 74%.

R_f = 0.40 (*n*-pentane: ethyl acetate = 90:10)

M.p.: 89–91 °C.

^{1}H NMR (400 MHz, $CDCl_3$) δ 7.08 (s, 1H), 6.90 (s, 1H), 6.04 (s, 2H), 5.49 (s, 2H) ppm.

^{13}C NMR (101 MHz, $CDCl_3$) δ149.9, 147.8, 122.2, 117.4, 113.1, 111.9, 102.4, 78.9 ppm.

IR (**ATR**): ṽ/cm^{-1} = 3098, 2970, 2907, 2780, 2726, 2599, 2436, 2319, 2098, 1924, 1841, 1726, 1617, 1539, 1477, 1412, 1362, 1307, 1238, 1118, 1034, 920, 892, 798, 737,675.

HRMS (**APCI**) for $C_8H_7BrNO_4^+$ [M+H]$^+$: calculated 259.9553, found 259.9549.

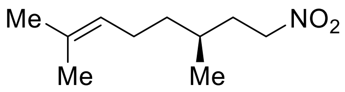

5a

$C_{10}H_{19}NO_2$
M = 185.14 g/mol

(*S*)-2,6-dimethyl-8-nitrooct-2-ene (**5a**): Synthetized according to the method mentioned in Section 3 with a scale of 5.0 mmol. Purification by flash column chromatography on silica gel using *n*-pentane:ethyl acetate = 10:1 and obtained as a yellow oil, yield: 77%.

R_f = 0.50 (*n*-pentane: ethyl acetate = 90:10)

^{1}H NMR (400 MHz, $CDCl_3$) δ 5.07 – 5.04 (m, 1H), 4.45 – 4.34 (m, 2H), 2.10 – 1.92 (m, 3H), 1.85 – 1.76 (m, 1H), 1.68 (d, J = 9.1 Hz, 3H), 1.59 (s, 3H), 1.56 – 1.49 (m, 1H), 1.39 – 1.30 (m, 1H), 1.26 – 1.16 (m, 1H), 0.93 (d, J = 6.7 Hz, 3H) ppm.

^{13}C NMR (101 MHz, $CDCl_3$) δ 131.8, 123.9, 74.1, 36.5, 34.2, 29.9, 25.6, 25.1, 18.9, 17.6 ppm.

IR (**ATR**): ṽ/cm^{-1} = 2915, 1549, 1434, 1378, 1206, 1116, 984, 829, 712.

HRMS (**APCI**) for $C_{10}H_{19}NO_2^+$ [M]$^+$: calculated 185.1410, found 185.1410.

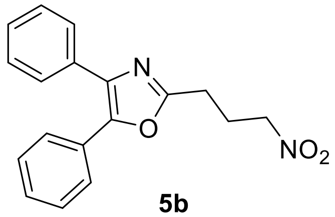

5b

$C_{18}H_{16}N_2O_3$
M = 308.12 g/mol

2-(3-Nitropropyl)-4,5-diphenyloxazole (**5b**): Synthetized according to the method mentioned in Section 3 with a scale of 5.0 mmol. Purification by flash column chromatography on silica gel using *n*-pentane:ethyl acetate = 5:1 and obtained as a yellow solid, yield: 69%.

R_f = 0.35 (*n*-pentane: ethyl acetate = 40:10)

M.p.: 83–85 °C.

^{1}H NMR (400 MHz, $CDCl_3$) δ 7.64 (d, *J* = 7.0 Hz, 2H), 7.58 (d, *J* = 6.7 Hz, 2H), 7.40 – 7.31 (m, 6H), 4.63 (t, *J* = 6.8 Hz, 2H), 3.01 (t, *J* = 7.1 Hz, 2H), 2.62 – 2.55 (m, 2H) ppm.

^{13}C NMR (101 MHz, $CDCl_3$) δ 161.0, 145.6, 135.1, 132.2, 128.7, 128.6, 128.2, 127.8, 126.5, 74.2, 24.8, 24.2 ppm.

IR (**ATR**): ṽ/cm^{-1} = 2922, 2342, 2097, 1896, 1588, 1543, 1441, 1384, 1354, 1296, 1208, 1057, 1024, 972, 919, 873, 805, 759, 693.

HRMS (APCI) for $C_{18}H_{17}N_2O_3^+$ [M+H]$^+$: calculated 309.1234, found 309.1231.

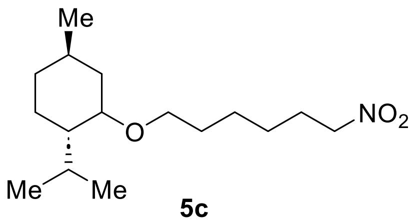

5c

$C_{16}H_{31}NO_3$

M = 285.23 g/mol

(1*S*,4*R*)-1-isopropyl-4-methyl-2-((6-nitrohexyl) oxy) cyclohexane (**5c**): Synthetized according to the method mentioned in Section 3 with a scale of 5.0 mmol. Purification by flash column chromatography on silica gel using *n*-pentane:ethyl acetate = 10:1 and obtained as a yellow oil, yield: 69%.

R_f = 0.30 (*n*-pentane: ethyl acetate = 90:10)

^{1}H NMR (500 MHz, $CDCl_3$) δ 4.38 (t, *J* = 7.1 Hz, 2H), 3.62 – 3.59 (m, 1H), 3.27 – 3.23 (m, 1H), 3.01 – 2.96 (m, 1H), 2.21 – 2.17 (m, 1H), 2.08 – 2.00 (m, 3H), 1.66 – 1.54 (m, 4H), 1.44 – 1.31 (m, 5H), 1.22 – 1.18 (m, 1H), 0.97 – 0.82 (m, 9H), 0.76 (d, *J* = 6.9 Hz, 3H) ppm.

^{13}C NMR (101 MHz, $CDCl_3$) δ 79.2, 75.6, 68.1, 48.3, 40.4, 34.6, 31.5, 29.9, 27.3, 26.1, 25.6, 25.6, 23.3, 22.3, 20.9, 16.2 ppm.

IR (**ATR**): ṽ/cm^{-1} = 3100, 2920, 2863, 2310, 2127, 1729, 1551, 1454, 1380, 1179, 1106, 972, 912, 844, 731.

HRMS (APCI) for $C_{16}H_{32}NO_3^+$ [M+H]$^+$: calculated 286.2377, found 286.2375.

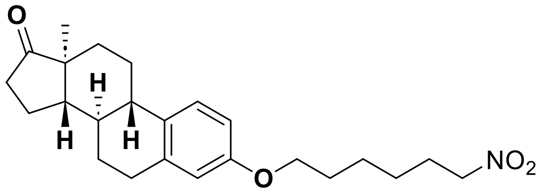

5d
$C_{24}H_{33}NO_4$
M = 399.24 g/mol

(8*R*,9*S*,13*S*,14*S*)-13-methyl-3-((6-nitrohexyl) oxy)-6,7,8,9,11,12,13,14,15,16-decahydro-17H-cyclopenta[*a*]phenanthren-17-one (**5d**): Synthetized according to the method mentioned in Section 3 with a scale of 5.0 mmol. Purification by flash column chromatography on silica gel using *n*-pentane:ethyl acetate = 5:1 and obtained as a white solid, yield: 65%.

R_f = 0.30 (*n*-pentane: ethyl acetate = 40:10)

M.p.: 110–112 °C.

^{1}H NMR (400 MHz, $CDCl_3$) δ 7.19 (d, *J* = 8.6 Hz, 1H), 6.70 (d, *J* = 8.5 Hz, 1H), 6.64 (s, 1H), 4.39 (t, *J* = 7.0 Hz, 2H), 3.93 (t, *J* = 6.3 Hz, 2H), 2.90 – 2.87 (m, 2H), 2.54 – 2.47 (m, 1H), 2.39 (d, *J* = 9.9 Hz, 1H), 2.25 (s, 1H), 2.19 – 1.94 (m, 7H), 1.78 (d, *J* = 6.5 Hz, 2H), 1.65 – 1.41 (m, 11H), 0.91 (s, 3H) ppm.

^{13}C NMR (101 MHz, $CDCl_3$) δ 220.9, 156.9, 137.7, 132.0, 126.3, 114.5, 112.0, 75.6, 67.4, 50.4, 48.0, 44.0, 38.4, 35.8, 31.6, 29.6, 28.9, 27.3, 26.5, 26.0, 25.9, 25.4, 21.6, 13.8 ppm.

IR (**ATR**): ṽ/cm^{-1} = 3475, 2927, 2860, 2264, 1735, 1608, 1548, 1499, 1471, 1454, 1379, 1253, 1161, 1055, 1006, 871, 817, 784, 738.

HRMS (**APCI**) for $C_{24}H_{34}NO_4^+$ [M+H]$^+$: calculated 400.2482, found 400.2479.

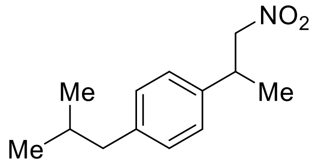

7a

$C_{13}H_{19}NO_2$
M = 221.14 g/mol

1-Isobutyl-4-(1-nitropropan-2-yl) benzene (**7a**): Synthetized according to the method mentioned in Section 3 with a scale of 1.7 mmol. Purification by flash column chromatography on silica gel using *n*-pentane:ethyl acetate = 10:1 and obtained as a yellow oli, yield: 88%.

$\mathbf{R_f}$ = 0.45 (*n*-pentane: ethyl acetate = 90:10)

^{1}H NMR (400 MHz, $CDCl_3$) δ 7.13 (d, *J* = 1.6 Hz, 4H), 4.57 – 4.44 (m, 2H), 3.66 – 3.57 (m, 1H), 2.45 (d, *J* = 7.1 Hz, 2H), 1.90 – 1.80 (m, 1H), 1.37 (d, *J* = 7.0 Hz, 3H), 0.90 (d, *J* = 6.6 Hz, 7H) ppm.

^{13}C NMR (101 MHz, $CDCl_3$) δ 141.0, 138.0, 129.6, 126.6, 82.0, 44.9, 38.3, 30.1, 22.3, 18.7 ppm.

IR (**ATR**): ṽ/cm^{-1} = 3019, 2954, 2924, 2224, 1976, 1905, 1736, 1547, 1460, 1429, 1377, 1199, 1125, 1029, 899, 845, 799, 700.

HRMS (**APCI**) $C_{13}H_{20}NO_2^+$ [M+H]$^+$: calculated 222.1489, found 222.1492.

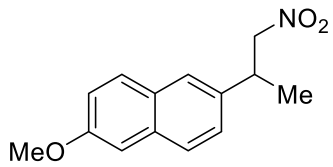

7b

$C_{14}H_{15}NO_3$
M = 245.11 g/mol

2-Methoxy-6-(1-nitropropan-2-yl) naphthalene (**7b**): Synthetized according to the method mentioned in Section 3 with a scale of 1.7 mmol. Purification by flash column chromatography on silica gel using *n*-pentane:ethyl acetate = 10:1 and obtained as a yellow oil, yield: 81%.

$\mathbf{R_f}$ = 0.30 (*n*-pentane: ethyl acetate = 90:10)

^{1}H NMR (400 MHz, $CDCl_3$) δ 7.72 (dd, *J* = 11.4, 8.7 Hz, 2H), 7.60 (s, 1H), 7.31 (dd, *J* = 8.4, 2.0 Hz, 1H), 7.16 (dd, *J* = 8.9, 2.5 Hz, 1H), 7.12 (d, *J* = 2.6 Hz, 1H), 4.64 (dd, *J* = 12.0, 7.2 Hz, 1H), 4.55 (dd, *J* = 12.0, 8.3 Hz, 1H), 3.92 (s, 3H), 3.82 – 3.73 (m, 1H), 1.46 (d, *J* = 7.1 Hz, 3H) ppm.

^{13}C NMR (101 MHz, $CDCl_3$) δ 157.8, 135.8, 133.9, 129.2, 128.9, 127.6, 125.6, 125.3, 119.3, 105.6, 81.9, 55.3, 38.6, 18.7 ppm.

IR (**ATR**): ṽ/cm^{-1} = 2965, 2934, 2839, 2750, 2653, 2442, 2293, 2221, 2117, 1912, 1716, 1605, 1545, 1455, 1377, 1334, 1265, 1211, 1161, 1125, 1028, 961, 890, 853, 811, 731, 674.

HRMS (**APCI**) $C_{14}H_{15}NO_3^+$ $[M]^+$: calculated 245.1046, found 245.1046.

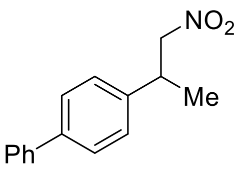

7c

$C_{15}H_{15}NO_2$

M = 241.29 g/mol

4-(1-Nitropropan-2-yl)-1,1'-biphenyl (**7c**): Synthetized according to the method mentioned in Section 3 with a scale of 1.7 mmol. Purification by flash column chromatography on silica gel using *n*-pentane:ethyl acetate = 10:1 and obtained as a yellow solid, yield: 96%.

R_f = 0.30 (*n*-pentane: ethyl acetate = 90:10)

M.p.: 82–84 °C.

^{1}H NMR (400 MHz, $CDCl_3$) δ 7.58 – 7.56 (m, 4H), 7.44 (t, *J* = 7.8 Hz, 2H), 7.37 – 7.34 (m, 1H), 7.31 (d, *J* = 8.4 Hz, 2H), 4.62 – 4.50 (m, 2H), 3.73 – 3.66 (m, 1H), 1.42 (d, *J* = 7.0 Hz, 3H) ppm.

^{13}C NMR (101 MHz, $CDCl_3$) δ ^{13}C NMR (126 MHz, $CDCl_3$) δ 140.5, 140.5, 139.8, 128.8, 127.6, 127.4, 127.3, 127.0, 81.8, 38.3, 18.7 ppm.

IR (**ATR**): ṽ/cm^{-1} = 2970, 2743, 2450, 2322, 2118, 1911, 1799, 1699, 1543, 1485, 1376, 1227, 1113, 1018, 908, 838, 766, 693.

HRMS (**APCI**) $C_{15}H_{15}NO_2^+$ $[M]^+$: calculated 241.1097, found 241.1097.

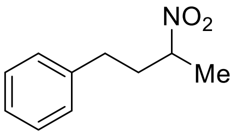

9

$C_{10}H_{13}NO_2$
M = 179.22 g/mol

(3-Nitrobutyl) benzene (**9**): Synthetized according to the method mentioned in Section 3 with a scale of 5.0 mmol. Purification by flash column chromatography on silica gel using *n*-pentane:ethyl acetate = 10:1 and obtained as a yellow oil, yield: 62%.

R_f = 0.60 (*n*-pentane: ethyl acetate = 90:10)

^{1}H NMR (400 MHz, $CDCl_3$) δ 7.38 – 7.34(m, 2H), 7.30 – 7.28 (m, 1H), 7.24 – 7.22 (m, 2H), 4.66 – 4.57 (m, 1H), 2.77 – 2.63 (m, 2H), 2.46 – 2.37 (m, 1H), 2.10 – 2.00 (m, 1H), 1.60 (d, J = 6.7 Hz, 3H) ppm.

^{13}C NMR (101 MHz, $CDCl_3$) δ 139.8, 128.6, 128.4, 126.4, 82.6, 36.6, 31.8, 19.3 ppm.

IR (**ATR**): ṽ/cm^{-1} = 3027, 2933, 2703, 1952, 1736, 1543, 1495, 1452, 1388, 1355, 1312, 1188, 1123, 918, 861, 817, 747, 697.

HRMS (**APCI**) $C_{10}H_{14}NO_2^+$ $[M+H]^+$: calculated 180.1019, found 180.1019.

4.3 Characterization data of nitrile products

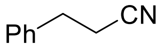

2a

C_9H_9N

M = 131.07 g/mol

3-Phenylpropanenitrile (**2a**): The reaction was performed on a 0.20 mmol scale. Purification by flash column chromatography on silica gel using *n*-pentane:ethyl acetate = 10:1 and obtained as a light-yellow oil, yield: 93%.

R_f = 0.30 (*n*-pentane: ethyl acetate = 90:10).

^{1}H NMR (500 MHz, $CDCl_3$) δ 7.37 (t, *J* = 7.6 Hz, 2H), 7.32 – 7.26 (m, 3H), 2.99 (t, *J* = 7.5 Hz, 2H), 2.65 (t, *J* = 7.4 Hz, 2H) ppm.

^{13}C NMR (126 MHz, $CDCl_3$) δ 138.0, 128.8, 128.2, 127.2, 119.0, 31.5, 19.3 ppm.

IR (**ATR**): ṽ/cm^{-1} = 3029, 2932, 2245, 2132, 1957, 1881, 1603, 1495, 1453, 1338, 1220, 1180, 1078, 1029, 976, 929, 746, 697.

HRMS (**APCI**) for $C_9H_{10}N^+$ [M+H]$^+$: calculated 132.0808, found 132.0804.

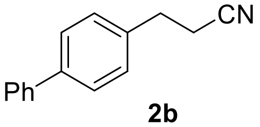

2b

$C_{15}H_{13}N$

M = 207.10 g/mol

3-([1,1'-Biphenyl]-4-yl) propane nitrile (**2b**): The reaction was performed on a 0.20 mmol scale. Purification by flash column chromatography on silica gel using *n*-pentane:ethyl acetate = 10:1 and obtained as a white solid, yield: 97%.

R_f = 0.30 (*n*-pentane: ethyl acetate = 90:10).

M.p.: 104–106 °C.

^{1}H NMR (500 MHz, $CDCl_3$) δ 7.60 – 7.57 (m, 4H), 7.45 (t, *J* = 7.6 Hz, 2H), 7.36 (t, *J* = 7.4 Hz, 1H), 7.32 (d, *J* = 7.9 Hz, 2H), 3.01 (t, *J* = 7.4 Hz, 2H), 2.66 (t, *J* = 7.4 Hz, 2H) ppm.

^{13}C NMR (126 MHz, $CDCl_3$) δ 140.6, 140.2, 137.0, 128.8, 128.7, 127.6, 127.3, 127.0, 119.1, 31.2, 19.3 ppm.

IR (**ATR**): ṽ/cm^{-1} = 3027, 2925, 2315, 2239, 2113, 1879, 1484, 1453, 1405, 1339, 1263, 1210, 1165, 1120, 910, 841, 760, 685.

HRMS (**APCI**) for $C_{15}H_{14}N^+$ $[M+H]^+$: calculated 208.1121, found 208.1117.

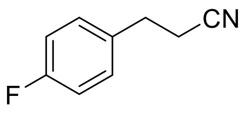

2c

C_9H_8FN

M = 149.06 g/mol

3-(4-Fluorophenyl) propane nitrile (**2c**): The reaction was performed on a 0.20 mmol scale. Purification by flash column chromatography on silica gel using *n*-pentane:ethyl acetate = 10:1 and obtained as light-yellow oil, yield: 75%.

R_f = 0.30 (*n*-pentane: ethyl acetate = 90:10).

^{1}H NMR (500 MHz, $CDCl_3$) δ 7.22 – 7.19 (m, 2H), 7.03 (t, *J* = 8.6 Hz, 2H), 2.93 (t, *J* = 7.3 Hz, 2H), 2.60 (t, *J* = 7.3 Hz, 2H) ppm.

^{13}C NMR (126 MHz, $CDCl_3$) δ 162.01 (d, $J_{C,F}$ = 245.5 Hz), 133.70 (d, $J_{C,F}$ = 3.2 Hz), 129.82 (d, $J_{C,F}$ = 8.0 Hz), 118.8, 115.71 (d, $J_{C,F}$ = 21.6 Hz), 30.7, 19.5 ppm.

^{19}F NMR (471 MHz, $CDCl_3$) δ -115.4 ppm.

IR (**ATR**): ṽ/cm^{-1} = 2934, 2246, 1893, 1600, 1508, 1423, 1342, 1220, 1158, 1096, 1016, 978, 920, 831, 764, 702.

HRMS (**APCI**) for $C_9H_9FN^+$ $[M+H]^+$: calculated 150.0714, found 150.0711.

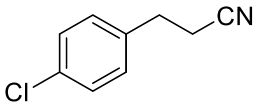

2d
C_9H_8ClN
M = 165.03 g/mol

3-(4-Chlorophenyl) propane nitrile (**2d**): The reaction was performed on a 0.20 mmol scale. Purification by flash column chromatography on silica gel using *n*-pentane:ethyl acetate = 10:1 and obtained as light-yellow oil, yield: 82%.

$\mathbf{R_f}$ = 0.30 (*n*-pentane: ethyl acetate = 90:10).

^{1}H NMR (500 MHz, $CDCl_3$) δ 7.31 (d, *J* = 8.3 Hz, 2H), 7.17 (d, *J* = 8.1 Hz, 2H), 2.92 (t, *J* = 7.3 Hz, 2H), 2.60 (t, *J* = 7.3 Hz, 2H) ppm.

^{13}C NMR (126 MHz, $CDCl_3$) δ 136.4, 133.1, 129.6, 129.0, 118.7, 30.9, 30.8, 19.2 ppm.

IR (**ATR**): ṽ/cm^{-1} = 3040, 2933, 2869, 2245, 2109, 2084, 1901, 1719, 1653, 1597, 1491, 1423 1341, 1181, 1090, 1014, 920, 806, 716, 666.

HRMS (**APCI**) for $C_9H_9ClN^+$ $[M+H]^+$: calculated 166.0418, found 166.0419.

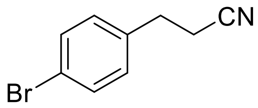

2e
C_9H_8BrN
M = 208.98 g/mol

3-(4-Bromophenyl) propane nitrile (**2e**): The reaction was performed on a 0.20 mmol scale. Purification by flash column chromatography on silica gel using *n*-pentane:ethyl acetate = 10:1 and obtained as light-yellow oil, yield: 94%.

$\mathbf{R_f}$ = 0.30 (*n*-pentane: ethyl acetate = 90:10).

^{1}H NMR (400 MHz, $CDCl_3$) δ 7.46 (d, *J* = 8.4 Hz, 1H), 7.11 (d, *J* = 8.4 Hz, 1H), 2.90 (t, *J* = 7.3 Hz, 1H), 2.60 (t, *J* = 7.3 Hz, 1H) ppm.

^{13}C NMR (101 MHz, $CDCl_3$) δ 136.9, 131.9, 130.0, 121.1, 118.7, 30.9, 19.1 ppm.

IR (**ATR**): ṽ/cm^{-1} = 3027, 2930, 2868, 2245, 2191, 2109, 2080, 1898, 1775, 1591, 1487, 1423, 1342, 1201, 1102, 1071, 1010, 920, 802, 710.

HRMS (**APCI**) for $C_9H_9BrN^+$ [M+H]$^+$: calculated 209.9913, found 209.9910.

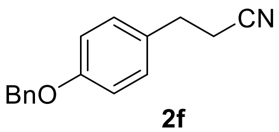

2f

$C_{16}H_{15}NO$

M = 237.12 g/mol

3-(4-(Benzyloxy) phenyl) propane nitrile (**2f**): The reaction was performed on a 0.20 mmol scale. Purification by flash column chromatography on silica gel using *n*-pentane:ethyl acetate = 10:1 and obtained as a white solid, yield: 94%.

R_f = 0.20 (*n*-pentane: ethyl acetate = 90:10).

M.p.: 94–96 °C.

^{1}H NMR (400 MHz, $CDCl_3$) δ 7.46 – 7.38 (m, 4H), 7.34 (t, *J* = 7.0 Hz, 1H), 7.16 (d, *J* = 8.6 Hz, 2H), 6.96 (d, *J* = 8.6 Hz, 2H), 5.06 (s, 2H), 2.90 (t, *J* = 7.4 Hz, 2H), 2.58 (t, *J* = 7.4 Hz, 2H) ppm.

^{13}C NMR (101 MHz, $CDCl_3$) δ 157.9, 136.8, 130.4, 129.3, 128.5, 127.9, 127.4, 119.2, 115.1, 115.1, 70.0, 69.9, 30.7, 19.5 ppm.

IR (**ATR**): ṽ/cm^{-1} = 3034, 2930, 2870, 2340, 2241, 2107, 1995, 1887, 1608, 1509, 1453, 1382, 1297, 1232, 1174, 1115, 1016, 915, 843, 805, 756, 723, 694.

HRMS (**APCI**) for $C_{16}H_{16}NO^+$ [M+H]$^+$: calculated 238.1226, found 238.1223.

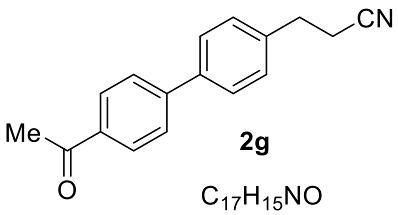

2g

$C_{17}H_{15}NO$
M = 249.12 g/mol

3-(4'-Acetyl-[1,1'-biphenyl]-4-yl) propane nitrile (**2g**): The reaction was performed on a 0.20 mmol scale. Purification by flash column chromatography on silica gel using *n*-pentane:ethyl acetate = 5:1 and obtained as a white solid, yield: 90%.

R$_f$ = 0.40 (*n*-pentane: ethyl acetate = 40:10).

M.p.: 142–144 °C.

^{1}H NMR (500 MHz, $CDCl_3$) δ 8.03 (d, *J* = 8.2 Hz, 2H), 7.67 (d, *J* = 8.1 Hz, 2H), 7.60 (d, *J* = 7.9 Hz, 2H), 7.34 (d, *J* = 7.9 Hz, 2H), 3.01 (t, *J* = 7.3 Hz, 2H), 2.68 – 2.63 (m, 5H).

^{13}C NMR (126 MHz, $CDCl_3$) δ 197.7, 145.1, 138.8, 138.1, 135.8, 128.9, 128.9, 127.7, 127.0, 119.0, 31.1, 26.6, 19.2.

IR (**ATR**): ṽ/cm^{-1} = 3340, 2917, 2853, 2342, 2245, 2119, 1995, 1915, 1800, 1673, 1596, 1491, 1396, 1361, 1316, 1261, 1215, 1178, 1137, 957, 803, 731.

HRMS (**APCI**) for $C_{17}H_{16}NO^+$ $[M+H]^+$: calculated 250.1226, found 250.1224.

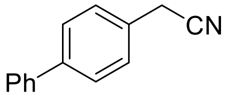

2i

$C_{14}H_{11}N$
M = 193.09 g/mol

2-([1,1'-Biphenyl]-4-yl) acetonitrile (**2i**): The reaction was performed on a 0.20 mmol scale. Purification by flash column chromatography on silica gel using *n*-pentane:ethyl acetate = 10:1 and obtained as a white solid, yield: 73%.

R$_f$ = 0.30 (*n*-pentane: ethyl acetate = 90:10).

M.p.: 95–97 °C.

^{1}H NMR (500 MHz, $CDCl_3$) δ 7.62 – 7.58 (m, 4H), 7.46 (t, J = 7.6 Hz, 2H), 7.42 – 7.36 (m, 3H), 3.80 (s, 2H) ppm.

^{13}C NMR (126 MHz, $CDCl_3$) δ 141.1, 140.2, 128.9, 128.8, 128.3, 127.8, 127.6, 127.0, 117.8, 23.3 ppm.

IR (**ATR**): ṽ/cm^{-1} = 3417, 3033, 2249, 2115, 1918, 1711, 1598, 1563, 1521, 1485, 1407, 1360, 1221, 1125, 1091, 1006, 907, 814, 751, 685.

HRMS (**APCI**) for $C_{14}H_{12}N^+$ [M+H]$^+$: calculated 194.0964, found 194.0965.

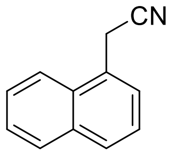

2j
$C_{12}H_9N$
M = 167.07 g/mol

2-(Naphthalen-1-yl) acetonitrile (**2j**): The reaction was performed on a 0.20 mmol scale. Purification by flash column chromatography on silica gel using *n*-pentane:ethyl acetate = 10:1 and obtained as a light-yellow oil, yield: 84%.

R_f = 0.30 (*n*-pentane: ethyl acetate = 90:10).

^{1}H NMR (500 MHz, $CDCl_3$) δ 7.92 (d, *J* = 8.1 Hz, 1H), 7.87 (d, *J* = 8.3 Hz, 2H), 7.63 – 7.55 (m, 3H), 7.49 – 7.46 (m, 1H), 4.13 (s, 2H) ppm.

^{13}C NMR (126 MHz, $CDCl_3$) δ 133.7, 130.7, 129.1, 129.0, 127.0, 126.4, 126.3, 125.8, 125.4, 122.4, 117.6, 21.7 ppm.

IR (**ATR**): ṽ/cm^{-1} = 3195, 3047, 2925, 2652, 2250, 2104, 1927, 1597, 1510, 1413, 1377, 1259, 1215, 1161, 1076, 1016, 970, 936, 851, 769.

HRMS (**APCI**) for $C_{12}H_{10}N^+$ [M+H]$^+$: calculated 168.0808, found 168.0806.

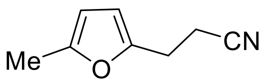

2k
C_8H_9NO
M = 135.07 g/mol

3-(5-Methylfuran-2-yl) propane nitrile (**2k**): The reaction was performed on a 0.20 mmol scale. Purification by flash column chromatography on silica gel using *n*-pentane:ethyl acetate = 10:1 and obtained as light-yellow oil, yield: 84%.

R$_f$ = 0.20 (*n*-pentane: ethyl acetate = 90:10).

^{1}H NMR (500 MHz, $CDCl_3$) δ 6.03 (d, *J* = 3.1 Hz, 1H), 5.88 (d, *J* = 1.7 Hz, 1H), 2.94 (t, *J* = 7.3 Hz, 2H), 2.64 (t, *J* = 7.3 Hz, 2H), 2.25 (s, 3H) ppm.

^{13}C NMR (101 MHz, $CDCl_3$) δ 151.6, 149.4, 118.9, 107.4, 106.2, 24.4, 16.7, 13.4 ppm.

IR (**ATR**): ṽ/cm^{-1} = 3417, 3106, 2924, 2248, 2119, 1769, 1698, 1613, 1568, 1423, 1390, 1215, 1185, 1102, 1023, 931, 785, 692.

HRMS (**APCI**) for $C_8H_{10}NO^+$ $[M+H]^+$: calculated 136.0757, found 136.0754.

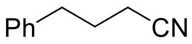

2l
$C_{10}H_{11}N$
M = 145.09 g/mol

4-Phenylbutanenitrile (**2l**): The reaction was performed on a 0.20 mmol scale. Purification by flash column chromatography on silica gel using *n*-pentane:ethyl acetate = 10:1 and obtained as a light-yellow oil, yield: 95%.

R$_f$ = 0.30 (*n*-pentane: ethyl acetate = 90:10).

^{1}H NMR (500 MHz, $CDCl_3$) δ 7.32 (t, *J* = 7.4 Hz, 2H), 7.23 (t, *J* = 7.4 Hz, 1H), 7.19 (d, *J* = 7.5 Hz, 2H), 2.79 (t, *J* = 7.4 Hz, 2H), 2.32 (t, *J* = 7.1 Hz, 2H), 2.02 – 1.96 (m, 2H) ppm.

^{13}C NMR (126 MHz, $CDCl_3$) δ 139.7, 128.6, 128.4, 126.5, 119.4, 34.3, 26.9, 16.3 ppm.

IR (ATR): ṽ/cm^{-1} = 3026, 2931, 2863, 2244, 2106, 1953, 1877, 1602, 1495, 1453, 1349, 1263, 1154, 1081, 1029, 911, 844, 801, 745, 698.

HRMS (**APCI**) for $C_{10}H_{12}N^+$ $[M+H]^+$: calculated 146.0964, found 146.0962.

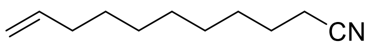

2m
$C_{11}H_{19}N$
M = 165.15 g/mol

Undec-10-ene nitrile (**2m**): The reaction was performed on a 0.20 mmol scale. Purification by flash column chromatography on silica gel using *n*-pentane:ethyl acetate = 10:1 and obtained as a light-yellow oil, yield: 73%.

R_f = 0.35 (*n*-pentane: ethyl acetate = 90:10).

^{1}H NMR (500 MHz, $CDCl_3$) δ 5.84 – 5.76 (m, 1H), 4.99 (dd, *J* = 17.1, 5.3 Hz, 1H), 4.93 (dd, *J* = 9.6, 1.7 Hz, 1H), 2.32 (t, *J* = 7.2 Hz, 2H), 2.06 – 2.02 (m, 2H), 1.68 – 1.62 (m, 2H), 1.47 – 1.42 (m, 2H), 1.39 – 1.28 (m, 8H) ppm.

^{13}C NMR (126 MHz, $CDCl_3$) δ 139.0, 119.7, 114.2, 33.7, 29.1, 28.9, 28.8, 28.7, 28.6, 25.3, 17.1 ppm.

IR (**ATR**): ṽ/cm^{-1} = 3075, 2925, 2854, 2683, 2245, 1823, 1733, 1639, 1461, 1438, 1352, 993, 908, 723.

HRMS (**APCI**) for $C_{11}H_{20}N^+$ $[M+H]^+$: calculated 166.1590, found 166.1586.

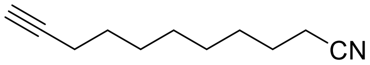

2n
$C_{11}H_{17}N$
M = 163.14 g/mol

Undec-10-ynenitrile (**2n**): The reaction was performed on a 0.20 mmol scale. Purification by flash column chromatography on silica gel using *n*-pentane:ethyl acetate = 10:1 and obtained as a light-yellow oil, yield: 77%.

R_f = 0.35 (*n*-pentane: ethyl acetate = 90:10).

^{1}H NMR (500 MHz, $CDCl_3$) δ 2.32 (t, *J* = 7.1 Hz, 2H), 2.19 – 2.16 (m, 2H), 1.93 (t, *J* = 2.7 Hz, 1H), 1.68 – 1.62 (m, 2H), 1.55 – 1.49 (m, 2H), 1.46 – 1.38 (m, 4H), 1.35 – 1.29 (m, 4H) ppm.

^{13}C NMR (126 MHz, $CDCl_3$) δ 119.7, 84.5, 68.1, 28.7, 28.6, 28.5, 28.5, 28.3, 25.3, 18.3, 17.1 ppm.

IR (**ATR**): ṽ/cm^{-1} = 3293, 2929, 2856, 2685, 2244, 2114, 1461, 1427, 1351, 1327, 1264, 1090, 1019, 920, 842.

HRMS (**APCI**) for $C_{11}H_{18}N^+$ [M+H]$^+$: calculated 164.1434, found 164.1429.

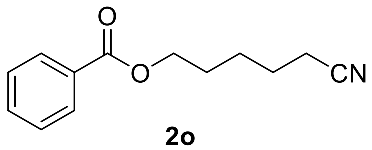

2o
$C_{13}H_{15}NO_2$
M = 217.11 g/mol

5-Cyanopentyl benzoate (**2o**): The reaction was performed on a 0.20 mmol scale. Purification by flash column chromatography on silica gel using *n*-pentane:ethyl acetate = 10:1 and obtained as light-yellow oil, yield: 72%.

R_f = 0.50 (*n*-pentane: ethyl acetate = 40:10).

^{1}H NMR (500 MHz, $CDCl_3$) δ 8.03 (d, *J* = 7.8 Hz, 2H), 7.55 (t, *J* = 7.6 Hz, 1H), 7.43 (t, *J* = 7.8 Hz, 2H), 4.33 (t, *J* = 6.7 Hz, 2H), 2.39 – 2.35 (m, 2H), 1.84 – 1.79 (m, 2H), 1.77 – 1.70 (m, 2H), 1.65 – 1.59 (m, 2H) ppm.

^{13}C NMR (126 MHz, $CDCl_3$) δ 166.5, 132.9, 130.2, 129.5, 128.3, 119.4, 64.3, 27.9, 25.2, 25.0, 17.0 ppm.

IR (**ATR**): ṽ/cm^{-1} = 3422, 3063, 2943, 2867, 2385, 2244, 2116, 1712, 1600, 1451, 1386, 1269, 1175, 1115, 1025, 953, 850, 807, 709.

HRMS (**APCI**) for $C_{13}H_{16}NO_2^+$ [M+H]$^+$: calculated 218.1176, found 218.1172.

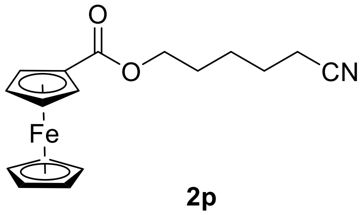

2p
$C_{17}H_{19}FeNO_2$
M = 325.08 g/mol

5-Cyanopentyl ferrocene carboxylate (**2p**): The reaction was performed on a 0.20 mmol scale. Purification by flash column chromatography on silica gel using *n*-pentane:ethyl acetate = 5:1 and obtained as yellow oil, yield: 57%.

R$_f$ = 0.30 (*n*-pentane: ethyl acetate = 40:10).

^{1}H NMR (400 MHz, $CDCl_3$) δ 4.80 (d, *J* = 3.9 Hz, 2H), 4.39 (d, *J* = 3.8 Hz, 2H), 4.21 (d, *J* = 12.2 Hz, 7H), 2.40 (t, *J* = 7.1 Hz, 2H), 1.81 – 1.72 (m, 4H), 1.65 – 1.58 (m, 2H) ppm.

^{13}C NMR (101 MHz, $CDCl_3$) δ 171.7, 119.5, 71.3, 71.1, 70.0, 69.7, 63.5, 28.1, 25.2, 25.0, 17.1 ppm.

IR (**ATR**): ṽ/cm^{-1} = 3348, 3098, 2937, 2245, 2110, 1877, 1703, 1603, 1511, 1458, 1371, 1272, 1135, 1026, 916, 822, 745.

HRMS (**APCI**) for $C_{17}H_{20}FeNO_2^+$ [M+H]$^+$: calculated 326.0832, found 326.0837.

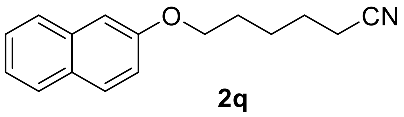

2q
$C_{16}H_{17}NO$
M = 239.13 g/mol

6-(Naphthalen-2-yloxy) hexane nitrile (**2q**): The reaction was performed on a 0.20 mmol scale. Purification by flash column chromatography on silica gel using *n*-pentane:ethyl acetate = 10:1 and obtained as a light-yellow oil, yield: 82%.

R$_f$ = 0.20 (*n*-pentane: ethyl acetate = 90:10).

^{1}H NMR (500 MHz, $CDCl_3$) δ 7.79 – 7.74 (m, 3H), 7.46 (t, *J* = 7.5 Hz, 1H), 7.35 (t, *J* = 7.5 Hz,

1H), 7.17 – 7.13 (m, 2H), 4.09 (t, *J* = 6.2 Hz, 2H), 2.37 (t, *J* = 6.9 Hz, 2H), 1.91 – 1.85 (m, 2H), 1.78 – 1.73 (m, 2H), 1.71 – 1.65 (m, 2H) ppm.

^{13}C NMR (126 MHz, $CDCl_3$) δ 156.8, 134.5, 129.3, 129.3, 128.9, 127.6, 126.6, 126.3, 123.5, 119.5, 118.8, 106.6, 67.3, 28.4, 25.4, 25.4, 25.1, 25.1, 17.0, 17.0 ppm.

IR (**ATR**): ṽ/cm^{-1} = 3196, 3055, 2934, 2867, 2243, 2076, 1908, 1626, 1598, 1509, 1462, 1389, 1353, 1256, 1214, 1179, 1118, 1045, 1013, 976, 915, 835, 746.

HRMS (**APCI**) for $C_{16}H_{18}NO^+$ [M+H]$^+$: calculated 240.1383, found 240.1377.

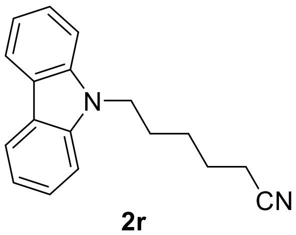

2r
$C_{18}H_{18}N_2$
M = 262.15 g/mol

6-(9*H*-carbazol-9-yl) hexane nitrile (**2r**): The reaction was performed on a 0.20 mmol scale. Purification by flash column chromatography on silica gel using *n*-pentane:ethyl acetate = 5:1 and obtained as light-yellow solid, yield: 82%.

R$_f$ = 0.20 (*n*-pentane: ethyl acetate = 90:10).

M.p.: 67–69 °C.

^{1}H NMR (400 MHz, $CDCl_3$) δ 8.11 (d, *J* = 7.9 Hz, 2H), 7.47 (t, *J* = 7.4 Hz, 2H), 7.38 (d, *J* = 8.1 Hz, 2H), 7.26 – 7.22 (m, 2H), 4.32 (t, *J* = 7.0 Hz, 2H), 2.26 (t, *J* = 7.0 Hz, 2H), 1.95 – 1.87 (m, 2H), 1.68 – 1.60 (m, 2H), 1.55 – 1.47 (m, 2H) ppm.

^{13}C NMR (101 MHz, $CDCl_3$) δ 140.2, 125.7, 122.8, 120.4, 119.4, 118.9, 108.4, 42.6, 28.3, 26.4, 25.2, 17.0 ppm.

IR (**ATR**): ṽ/cm^{-1} = 3047, 2930, 2860, 2645, 2242, 2106, 1891, 1774, 1594, 1451, 1377, 1324, 1229, 1152, 1122, 1067, 999, 928, 849, 748, 722.

HRMS (**APCI**) for $C_{18}H_{19}N_2^+$ [M+H]+: calculated 263.1543, found 263.1540.

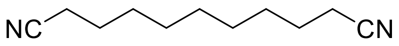

2s
$C_{11}H_{18}N_2$
M = 178.15 g/mol

Undecane dinitrile (**2s**): The reaction was performed on a 0.20 mmol scale. Purification by flash column chromatography on silica gel using *n*-pentane:ethyl acetate = 5:1 and obtained as a light-yellow oil, yield: 96%.

R_f = 0.30 (*n*-pentane: ethyl acetate = 90:10).

^{1}H NMR (500 MHz, $CDCl_3$) δ 2.34 (t, *J* = 7.1 Hz, 4H), 1.69 – 1.63 (m, 4H), 1.48 – 1.42 (m, 4H), 1.35 – 1.32 (m, 6H) ppm.

^{13}C NMR (126 MHz, $CDCl_3$) δ 119.7, 29.0, 28.6, 28.6, 25.3, 17.1 ppm.

IR (**ATR**): ṽ/cm^{-1} = 2927, 2855, 2694, 2244, 1721, 1582, 1461, 1425, 1374, 1274, 1110, 1041, 942, 848, 802, 723.

HRMS (**APCI**) for $C_{11}H_{19}N_2^+$ [M+H]+: calculated 179.1543, found 179.1540.

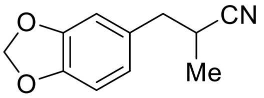

2t
$C_{11}H_{11}NO_2$
M = 189.08 g/mol

3-(Benzo[d] [1,3] dioxol-5-yl)-2-methylpropanenitrile (**2t**): The reaction was performed on a 0.20 mmol scale. Purification by flash column chromatography on silica gel using *n*-pentane:ethyl acetate = 5:1 and obtained as light-yellow oil, yield: 84%.

R_f = 0.50 (*n*-pentane: ethyl acetate = 40:10).

^{1}H NMR (500 MHz, $CDCl_3$) δ 6.76 (d, *J* = 7.9 Hz, 1H), 6.71 (s, 1H), 6.68 (d, *J* = 7.8 Hz, 1H),

5.94 (s, 2H), 2.86 – 2.72 (m, 4H), 1.31 (d, *J* = 6.5 Hz, 3H) ppm.

^{13}C NMR (101 MHz, $CDCl_3$) δ 147.8, 146.7, 130.5, 122.5, 122.2, 109.2, 108.4, 108.3, 101.0, 39.7, 27.7, 17.5 ppm.

IR (**ATR**): ṽ/cm^{-1} = 2981, 2895, 2778, 2238, 2133, 1916, 1847, 1748, 1608, 1487, 1441, 1362, 1326, 1244, 1190, 1100, 1034, 926, 861, 811, 766, 674.

HRMS (**APCI**) for $C_{11}H_{12}NO_2^+$ [M+H]$^+$: calculated 190.0863, found 190.0860.

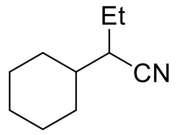

2u-rac
$C_{10}H_{17}N$
M= 151.14 g/mol

2-Cyclohexylbutanenitrile (**2u-rac**): The reaction was performed on a 0.20 mmol scale. Purification by flash column chromatography on silica gel using *n*-pentane:ethyl acetate = 10:1 and obtained as light-yellow oil, yield: 86%.

R_f = 0.70 (*n*-pentane: ethyl acetate = 90:10).

^{1}H NMR (400 MHz, $CDCl_3$) δ 2.34 – 2.29 (m, 1H), 1.90 – 1.85 (m, 1H), 1.81 – 1.58 (m, 6H), 1.54 – 1.44 (m, 1H), 1.31 – 1.20 (m, 4H), 1.18 – 1.13 (m, 2H), 1.07 (t, *J* = 7.4 Hz, 3H) ppm.

^{13}C NMR (101 MHz, $CDCl_3$) δ 40.2, 38.9, 31.3, 29.5, 26.0, 26.0, 25.9, 22.9, 12.0 ppm.

IR (**ATR**): ṽ/cm^{-1} = 3589, 2924, 2854, 2110, 1724, 1460, 1377, 1267, 1164, 1115, 1019, 821, 730.

HRMS (APCI) for $C_{10}H_{18}N^+$ [M+H]$^+$: calculated 152.1434, found 152.1436.

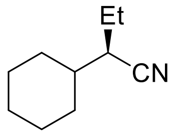

2u

$C_{10}H_{17}N$
M= 151.14 g/mol

(*R*)-2-Cyclohexylbutanenitrile (**2u**): The reaction was performed on a 0.20 mmol scale. Purification by flash column chromatography on silica gel using *n*-pentane:ethyl acetate = 10:1 and obtained as light-yellow oil, yield: 87%.

R_f = 0.70 (*n*-pentane: ethyl acetate = 90:10).

^{1}H NMR (400 MHz, $CDCl_3$) δ 2.34 – 2.29 (m, 1H), 1.90 – 1.85 (m, 1H), 1.81 – 1.58 (m, 6H), 1.54 – 1.44 (m, 1H), 1.31 – 1.20 (m, 4H), 1.18 – 1.13 (m, 2H), 1.07 (t, *J* = 7.4 Hz, 3H) ppm.

^{13}C NMR (101 MHz, $CDCl_3$) δ 40.2, 38.9, 31.3, 29.5, 26.0, 26.0, 25.9, 22.9, 12.0 ppm.

IR (**ATR**): ṽ/cm^{-1} = 3353, 2925, 2854, 2431, 2162, 2035, 1724, 1456, 1378, 1263, 1169, 1100, 1021, 804.

HRMS (APCI) for $C_{10}H_{18}N^+$ [M+H]$^+$: calculated 152.1434, found 152.1436.

The enantiomeric ratio of **10a** was determined by chiral GLC analysis (Sigma-Aldrich Astec Chiraldex B-DM column (30 m × 0.25 mm, 0.12 μm film thickness), column temperature 60 °C (30 min), ramp of 0.5 °C/min to 175 °C (20 min), flow rate isobaric 14 psi): t_R = 85.4 min (major), t_R = 88.4 min (minor).

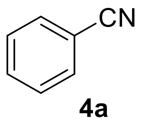

4a

C_7H_5N
M = 103.12 g/mol

Benzonitrile (**4a**): The reaction was performed on a 0.20 mmol scale. Purification by flash column chromatography on silica gel using *n*-pentane:ethyl acetate = 10:1 and obtained as

light-yellow oil, yield: 77%.

R$_f$ = 0.30 (*n*-pentane: ethyl acetate = 90:10).

^{1}H NMR (400 MHz, $CDCl_3$) δ 7.65 – 7.58 (m, 3H), 7.46 (t, *J* = 7.7 Hz, 2H) ppm.

^{13}C NMR (101 MHz, $CDCl_3$) δ 132.7, 132.0, 129.1, 129.0, 118.7, 112.3 ppm.

IR (**ATR**): ṽ/cm^{-1} = 3029, 2932, 2348, 2245, 2115, 1872, 1603, 1495, 1453, 1338, 1078, 1029, 929, 746, 697.

HRMS (**APCI**) for $C_7H_6N^+$ [M+H]$^+$: calculated 104.0495, found 104.0491.

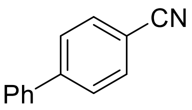

4b

$C_{13}H_9N$

M =179.22 g/mol

[1,1'-Biphenyl]-4-carbonitrile (**4b**): The reaction was performed on a 0.20 mmol scale. Purification by flash column chromatography on silica gel using *n*-pentane:ethyl acetate = 10:1 and obtained as light-yellow solid, yield: 84%.

R$_f$ = 0.30 (*n*-pentane: ethyl acetate = 90:10).

M.p.: 85–87 °C.

^{1}H NMR (400 MHz, $CDCl_3$) δ 7.73 (d, *J* = 8.3 Hz, 2H), 7.69 (d, *J* = 8.5 Hz, 2H), 7.59 (d, *J* = 7.3 Hz, 2H), 7.49 (t, *J* = 7.5 Hz, 2H), 7.43 (t, *J* = 7.3 Hz, 1H) ppm.

^{13}C NMR (101 MHz, $CDCl_3$) δ 145.6, 139.1, 132.6, 129.1, 128.6, 127.7, 127.2, 118.9, 110.9 ppm.

IR (**ATR**): ṽ/cm^{-1} = 2924, 2358, 2222, 2150, 1930, 1806, 1685, 1602, 1581, 1480, 1395, 1177, 1128, 1076, 1005, 921, 845, 767, 696.

HRMS (**APCI**) for $C_{13}H_{10}N^+$ [M+H]$^+$: calculated 180.0808, found 180.0804.

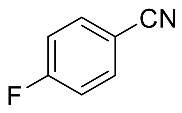

4c

C_7H_4FN
M = 121.11 g/mol

4-Fluorobenzonitrile (**4c**): The reaction was performed on a 0.20 mmol scale. Purification by flash column chromatography on silica gel using *n*-pentane:ethyl acetate = 10:1 and obtained as light-yellow oil, yield: 79%.

R_f = 0.30 (*n*-pentane: ethyl acetate = 90:10).

M.p.: 32–34 °C.

^{1}H NMR (400 MHz, $CDCl_3$) δ 7.69 – 7.66 (m, 2H), 7.17 (t, J = 8.6 Hz, 2H) ppm.

^{13}C NMR (101 MHz, $CDCl_3$) δ164.97 (d, $J_{C,F}$ = 256.5 Hz), 134.62 (d, $J_{C,F}$ = 9.4 Hz), 118.0, 116.79 (d, $J_{C,F}$ = 22.8 Hz), 108.51 (d, $J_{C,F}$ = 3.6 Hz) ppm.

^{19}F NMR (471 MHz, $CDCl_3$) δ -102.4 ppm.

IR (**ATR**): ṽ/cm^{-1} = 3072, 2999, 2232, 1911, 1655, 1601, 1505, 1407, 1236, 1161, 1096, 1020, 835, 682.

HRMS (**APCI**) for $C_7H_5FN^+$ $[M+H]^+$: calculated 122.0401, found 122.0399.

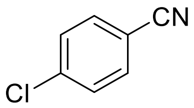

4d

C_7H_4ClN
M = 137.57 g/mol

4-Chlorobenzonitrile (**4d**): The reaction was performed on a 0.20 mmol scale. Purification by flash column chromatography on silica gel using *n*-pentane:ethyl acetate = 10:1 and obtained

as light-yellow solid, yield: 88%.

R$_f$ = 0.30 (*n*-pentane: ethyl acetate = 90:10).

M.p.: 90–93 °C.

^{1}H NMR (400 MHz, $CDCl_3$) δ 7.60 (d, *J* = 8.4 Hz, 2H), 7.46 (d, *J* = 8.6 Hz, 2H) ppm.

^{13}C NMR (101 MHz, $CDCl_3$) δ 139.5, 133.3, 129.6, 117.9, 110.7 ppm.

IR (**ATR**): ṽ/cm^{-1} = 3182, 3090, 2575, 2318, 2223, 2114, 1913, 1781, 1654, 1590, 1480, 1396, 1278, 1085, 1014, 902, 824, 778.

HRMS (**APCI**) for $C_7H_5ClN^+$ $[M+H]^+$: calculated 138.0105, found 138.0104.

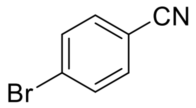

4e

C_7H_4BrN
M = 182.02 g/mol

4-Bromobenzonitrile (**4e**): The reaction was performed on a 0.20 mmol scale. Purification by flash column chromatography on silica gel using *n*-pentane:ethyl acetate = 10:1 and obtained as light-yellow solid, yield: 76%.

R$_f$ = 0.30 (*n*-pentane: ethyl acetate = 90:10).

M.p.: 112–114 °C.

^{1}H NMR (400 MHz, $CDCl_3$) δ 7.63 (d, *J* = 8.5 Hz, 2H), 7.52 (d, *J* = 8.5 Hz, 2H) ppm.

^{13}C NMR (101 MHz, $CDCl_3$) δ 133.4, 132.6, 128.0, 118.0, 111.2 ppm.

IR (**ATR**): ṽ/cm^{-1} = 3085, 2559, 2370, 2222, 2111, 1915, 1791, 1662, 1582, 1476, 1388, 1253, 1174, 1066, 1012, 821, 769.

HRMS (**APCI**) for $C_7H_5BrN^+$ $[M+H]^+$: calculated 181.9600, found 181.9600.

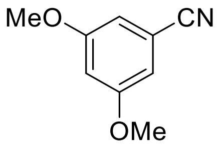

4f
$C_9H_9NO_2$
M = 163.18 g/mol

3,5-Dimethoxybenzonitrile (**4f**): The reaction was performed on a 0.20 mmol scale. Purification by flash column chromatography on silica gel using *n*-pentane:ethyl acetate = 10:1 and obtained as light-yellow solid, yield: 98%.

R$_f$ = 0.20 (*n*-pentane: ethyl acetate = 90:10).

M.p.: 87–89 °C.

^{1}H NMR (400 MHz, $CDCl_3$) δ 6.74 (s, 2H), 6.64 (s, 1H), 3.79 (s, 6H) ppm.

^{13}C NMR (101 MHz, $CDCl_3$) δ 160.9, 118.7, 113.3, 109.8, 105.5, 55.6 ppm.

IR (**ATR**): ṽ/cm^{-1} = 3194, 3095, 2941, 2842, 2228, 2092, 1899, 1594, 1457, 1349, 1328, 1256, 1208, 1160, 1049, 907, 834, 729, 670.

HRMS (**APCI**) for $C_9H_{10}NO_2^+$ [M+H]$^+$: calculated 164.0706, found 164.0702.

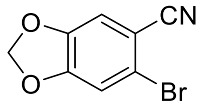

4g
$C_8H_4BrNO_2$
M = 226.03 g/mol

6-Bromobenzo[d] [1,3] dioxole-5-carbonitrile (**4g**): The reaction was performed on a 0.20 mmol scale. Purification by flash column chromatography on silica gel using *n*-pentane:ethyl acetate = 10:1 and obtained as light-yellow solid, yield: 69%.

R$_f$ = 0.20 (*n*-pentane: ethyl acetate = 90:10).

M.p.: 99–101 °C.

^{1}H NMR (400 MHz, $CDCl_3$) δ 7.08 (s, 1H), 7.02 (s, 1H), 6.10 (s, 2H) ppm.

^{13}C NMR (101 MHz, $CDCl_3$) δ 152.2, 147.4, 118.9, 117.3, 113.4, 112.5, 107.9, 103.0 ppm.

IR (**ATR**): ṽ/cm^{-1} = 3208, 3102, 3052, 2999, 2916, 2597, 2302, 2229, 2085, 1915, 1840, 1752, 1704, 1610, 1408, 1366, 1257, 1114, 1032, 922, 879, 836, 737.

HRMS (**APCI**) for $C_8H_5BrNO_2^+$ [M+H]$^+$: calculated 225.9498, found 225.9494.

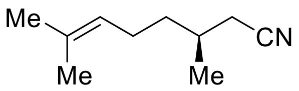

6a
$C_{10}H_{17}N$
M = 151.14 g/mol

(*S*)-3,7-dimethyloct-6-enenitrile (**6a**): The reaction was performed on a 0.20 mmol scale. Purification by flash column chromatography on silica gel using *n*-pentane:ethyl acetate = 10:1 and obtained as light-yellow oil, yield: 72%.

R_f = 0.40 (*n*-pentane: ethyl acetate = 90:10).

^{1}H NMR (500 MHz, $CDCl_3$) δ 5.08 – 5.05 (m, 1H), 2.34 – 2.29 (m, 1H), 2.25 – 2.21 (m, 1H), 2.05 – 1.97 (m, 2H), 1.89 – 1.83 (m, 1H), 1.68 (s, 3H), 1.59 (d, *J* = 12.1 Hz, 3H), 1.49 – 1.42 (m, 1H), 1.37 – 1.30 (m, 1H), 1.07 (d, *J* = 6.7 Hz, 3H) ppm.

^{13}C NMR (126 MHz, $CDCl_3$) δ 132.2, 123.4, 118.8, 35.9, 29.9, 25.6, 25.2, 24.4, 19.3, 17.6 ppm.

IR (**ATR**): ṽ/cm^{-1} = 2963, 2918, 2855, 2732, 2244, 1721, 1456, 1381, 1273, 1243, 1175, 1111, 983, 885, 824, 740.

HRMS (**APCI**) for $C_{10}H_{18}N^+$ [M+H]$^+$: calculated 152.1434, found 152.1431.

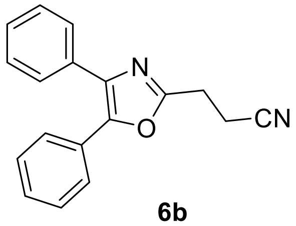

6b
$C_{18}H_{14}N_2O$
M =274.11 g/mol

3-(4,5-Diphenyloxazol-2-yl) propane nitrile (**6b**): The reaction was performed on a 0.20 mmol scale. Purification by flash column chromatography on silica gel using *n*-pentane:ethyl acetate = 10:1 and obtained as light-yellow solid, yield: 98%.

R$_f$ = 0.30 (*n*-pentane: ethyl acetate = 40:10).

M.p.: 118–120 °C.

^{1}H NMR (400 MHz, $CDCl_3$) δ 7.63 (d, *J* = 7.2 Hz, 2H), 7.58 (d, *J* = 5.7 Hz, 2H), 7.40 – 7.32 (m, 6H), 3.24 (t, *J* = 7.5 Hz, 2H), 2.95 (t, *J* = 7.5 Hz, 2H) ppm.

^{13}C NMR (101 MHz, $CDCl_3$) δ 159.1, 146.1, 135.3, 131.9, 128.7, 128.7, 128.6, 128.5, 128.3, 127.8, 126.5, 118.3, 24.5, 14.9 ppm.

IR (**ATR**): ṽ/cm^{-1} = 3058, 2923, 2253, 2097, 1582, 1500, 1436, 1357, 1300, 1274, 1213, 1177, 1056, 1022, 958, 918, 855, 763, 696.

HRMS (**APCI**) for $C_{18}H_{15}N_2O^+$ [M+H]$^+$: calculated 275.1179, found 275.1176.

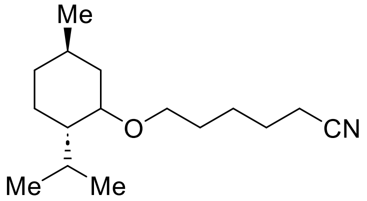

6c
$C_{16}H_{29}NO$
M = 251.22 g/mol

6-(((2*S*,5*R*)-2-isopropyl-5-methylcyclohexyl) oxy) hexane nitrile (**6c**): The reaction was performed on a 0.20 mmol scale. Purification by flash column chromatography on silica gel using *n*-pentane:ethyl acetate = 10:1 and obtained as light-yellow oil, yield: 79%.

$\mathbf{R_f}$ = 0.20 (*n*-pentane: ethyl acetate = 90:10).

^{1}**H NMR** (400 MHz, $CDCl_3$) δ 3.65 – 3.60 (m, 1H), 3.28 – 3.23 (m, 1H), 3.02 – 2.95 (m, 1H), 2.34 (t, *J* = 7.1 Hz, 2H), 2.22 – 2.15 (m, 1H), 2.07 (d, *J* = 11.4 Hz, 1H), 1.72 – 1.49 (m, 8H), 1.37 – 1.28 (m, 1H), 1.25 – 1.16 (m, 1H), 0.97 – 0.80 (m, 9H), 0.76 (d, *J* = 7.0 Hz, 3H) ppm.

^{13}**C NMR** (101 MHz, $CDCl_3$) δ 119.7, 79.2, 67.8, 48.2, 40.4, 34.5, 31.5, 29.4, 25.6, 25.5, 25.2, 23.3, 22.3, 20.9, 17.1, 16.2 ppm.

IR (**ATR**): ṽ/cm^{-1} = 2919, 2865, 2657, 2245, 2137, 1739, 1454, 1369, 1238, 1180, 1110, 972, 920, 844, 731.

HRMS (APCI) for $C_{16}H_{30}NO^+$ $[M+H]^+$: calculated 252.2322, found 252.2319.

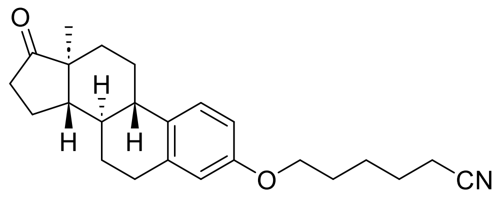

6d
$C_{24}H_{31}NO_2$
M = 365.24 g/mol

6-(((8*R*,9*S*,13*S*,14*S*)-13-methyl-17-oxo-7,8,9,11,12,13,14,15,16,17-decahydro-6H-cyclopenta[*a*]phenanthren-3-yl) oxy) hexane nitrile (**6d**): The reaction was performed on a 0.20 mmol scale. Purification by flash column chromatography on silica gel using *n*-pentane:ethyl acetate = 10:1 and obtained as white solid, yield: 96%.

$\mathbf{R_f}$ = 0.20 (*n*-pentane: ethyl acetate = 40:10).

M.p.: 116–118 °C.

^{1}**H NMR** (400 MHz, $CDCl_3$) δ 7.20 (d, *J* = 8.6 Hz, 1H), 6.70 (d, *J* = 7.4 Hz, 1H), 6.64 (s, 1H),

3.95 (t, J = 6.1 Hz, 2H), 2.90 – 2.88 (m, 2H), 2.54 – 2.49 (m, 1H), 2.38 (t, J = 7.0 Hz, 3H), 2.25 (s, 1H), 2.19 – 1.94 (m, 4H), 1.84 – 1.71 (m, 4H), 1.68 – 1.41 (m, 9H), 0.91 (s, 3H) ppm.

^{13}C NMR (101 MHz, $CDCl_3$) δ 221.0, 156.8, 137.8, 132.1, 126.3, 119.6, 114.5, 112.0, 67.2, 50.4, 48.0, 43.9, 38.3, 35.9, 31.5, 29.6, 28.5, 26.5, 25.9, 25.4, 25.2, 21.6, 17.1, 13.8 ppm.

IR (**ATR**): ṽ/cm^{-1} = 2936, 2872, 2243, 2094, 1729, 1609, 1496, 1379, 1250, 1157, 1100, 1056, 1026, 915, 872, 815, 738.

HRMS (**APCI**) for $C_{24}H_{32}NO_2^+$ [M+H]$^+$: calculated 366.2428, found 366.2422.

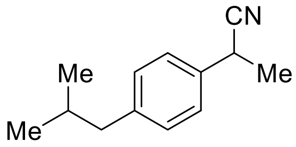

8a

$C_{13}H_{17}N$

M = 187.14 g/mol

2-(4-Isobutylphenyl) propane nitrile (**8a**): The reaction was performed on a 0.20 mmol scale. Purification by flash column chromatography on silica gel using *n*-pentane:ethyl acetate = 10:1 and obtained as light-yellow oil, yield: 92%.

R_f = 0.40 (*n*-pentane: ethyl acetate = 90:10).

^{1}H NMR (400 MHz, $CDCl_3$) δ 7.26 (d, J = 8.2 Hz, 2H), 7.16 (d, J = 8.2 Hz, 2H), 3.88 (q, J = 7.3 Hz, 1H), 2.48 (d, J = 7.2 Hz, 2H), 1.91 – 1.81 (m, 1H), 1.63 (d, J = 7.3 Hz, 3H), 0.91 (d, J = 6.6 Hz, 7H) ppm.

^{13}C NMR (101 MHz, $CDCl_3$) δ 141.6, 134.2, 129.7, 126.4, 121.8, 44.9, 30.8, 30.1, 22.2, 21.4 ppm.

IR (**ATR**): ṽ/cm^{-1} = 2954, 2315, 2240, 2093, 1905, 1681, 1606, 1511, 1454, 1382, 1266, 1167, 1084, 844, 795, 724.

HRMS (APCI) for $C_{13}H_{18}N^+$ [M+H]$^+$: calculated 188.1434, found 188.1435.

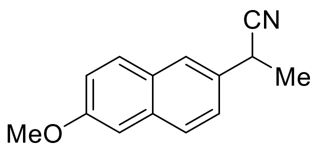

8b

$C_{14}H_{13}NO$
M = 211.10 g/mol

2-(6-Methoxynaphthalen-2-yl) propane nitrile (**8b**): The reaction was performed on a 0.20 mmol scale. Purification by flash column chromatography on silica gel using *n*-pentane:ethyl acetate = 10:1 and obtained as light-yellow solid, yield: 83%.

R$_f$ = 0.20 (*n*-pentane: ethyl acetate = 90:10).

M.p.: 67–69 °C.

^{1}H NMR (400 MHz, $CDCl_3$) δ 7.77 – 7.72 (m, 3H), 7.39 (dd, *J* = 8.4, 2.0 Hz, 1H), 7.19 (dd, *J* = 8.9, 2.5 Hz, 1H), 7.14 (d, *J* = 2.5 Hz, 1H), 4.03 (q, *J* = 7.3 Hz, 1H), 3.93 (s, 3H), 1.71 (d, *J* = 7.3 Hz, 3H) ppm.

^{13}C NMR (101 MHz, $CDCl_3$) δ 158.0, 133.9, 131.9, 129.3, 128.7, 127.8, 125.3, 124.9, 121.7, 119.5, 105.6, 55.3, 31.2, 21.4 ppm.

IR (**ATR**): ṽ/cm^{-1} = 3206, 2983, 2937, 2841, 2533, 2423, 2295, 2239, 2112, 1915, 1710, 1604, 1452, 1390, 1265, 1213, 1161, 1083, 1027, 960, 927, 890, 853, 812, 749, 671.

HRMS (APCI) for $C_{14}H_{14}NO^+$ $[M+H]^+$: calculated 212.1070, found 212.1069.

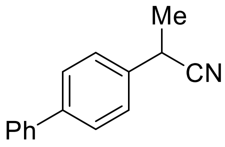

8c

$C_{15}H_{13}N$
M = 207.28 g/mol

2-([1,1'-Biphenyl]-4-yl) propane nitrile (**8c**): The reaction was performed on a 0.20 mmol scale. Purification by flash column chromatography on silica gel using *n*-pentane:ethyl acetate

= 10:1 and obtained as light-yellow solid, yield: 63%.

R_f = 0.20 (*n*-pentane: ethyl acetate = 90:10).

M.p.: 61–63 °C.

^{1}H NMR (400 MHz, $CDCl_3$) δ 7.63 – 7.58 (m, 4H), 7.48 – 7.43 (m, 4H), 7.40 – 7.36 (m, 1H), 3.96 (q, J = 7.3 Hz, 1H), 1.69 (d, J = 7.3 Hz, 3H) ppm.

^{13}C NMR (101 MHz, $CDCl_3$) δ 141.1, 140.2, 136.0, 128.8, 127.8, 127.6, 127.1, 127.0, 121.5, 30.9, 21.4ppm.

IR (**ATR**): ṽ/cm^{-1} = 3030, 2984, 2932, 2345, 2240, 2107, 1803, 1680, 1601, 1486, 1405, 1265, 1084, 991, 838, 764, 697.

HRMS (APCI) for $C_{15}H_{13}N^+$ $[M]^+$: calculated 207.1043, found 207.1042.

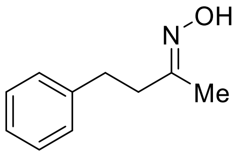

10

$C_{10}H_{13}NO$
M = 163.22 g/mol

(*E*)-4-Phenylbutan-2-one oxime (**10**): The reaction was performed on a 0.20 mmol scale. Purification by flash column chromatography on silica gel using *n*-pentane:ethyl acetate = 10:1 and obtained as white solid, yield: 77%.

R_f = 0.60 (*n*-pentane: ethyl acetate = 90:10).

^{1}H NMR (400 MHz, $CDCl_3$) δ 8.85 (s, 1H), 7.32 – 7.28 (m, 2H), 7.23 – 7.20 (m, 3H), 2.87 – 2.83 (m, 2H), 2.54 – 2.50 (m, 2H), 1.93 (s, 3H) ppm.

^{13}C NMR (101 MHz, $CDCl_3$) δ 157.9, 141.0, 128.4, 128.3, 126.1, 77.3, 77.0, 76.7,37.7, 32.6, 13.8 ppm.

5 GLC Traces of Starting Material 1u and Product 2u

5.1 (*R*)- (1-Nitrobutan-2-yl) cyclohexane (1u)

Figure S1. rac-1-Nitrobutan-2-yl) cyclohexane (**1u-rac**)

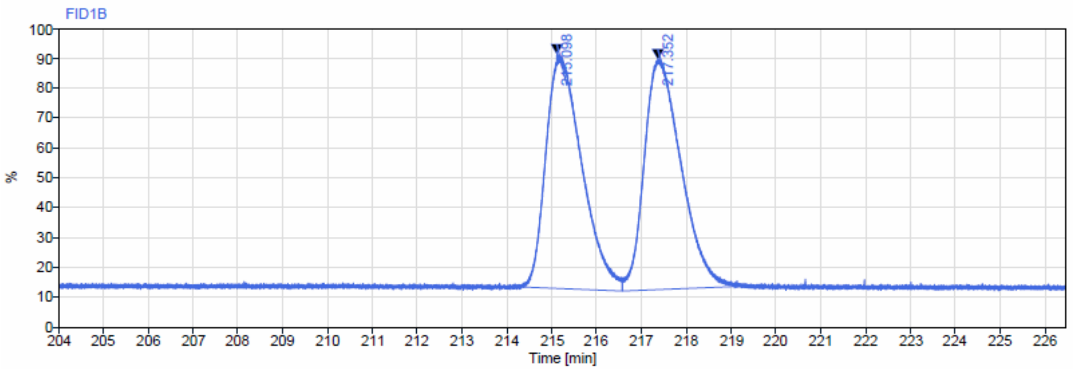

Signal:	FID1B					
RT [min]	Type	Width [min]	Area	Height	Area%	Name
215.098	MM m	2.21	64.10	1.15	49.70	
217.352	MM m	2.67	64.86	1.13	50.30	
		Sum	128.96			

Figure S2. (*R*)- (1-Nitrobutan-2-yl) cyclohexane (**1u**)

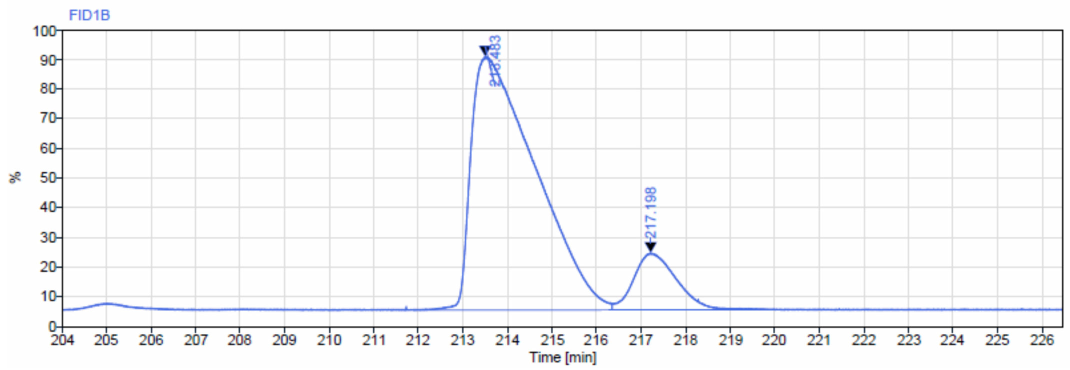

Signal:	FID1B					
RT [min]	Type	Width [min]	Area	Height	Area%	Name
213.483	MM m	4.46	527.90	5.25	86.99	
217.198	MM m	4.81	78.99	1.18	13.01	
		Sum	606.89			

5.2 (*R*)-2-Cyclohexylbutanenitrile (2u)

Figure S3. rac-2-Cyclohexylbutanenitrile (**2u-rac**)

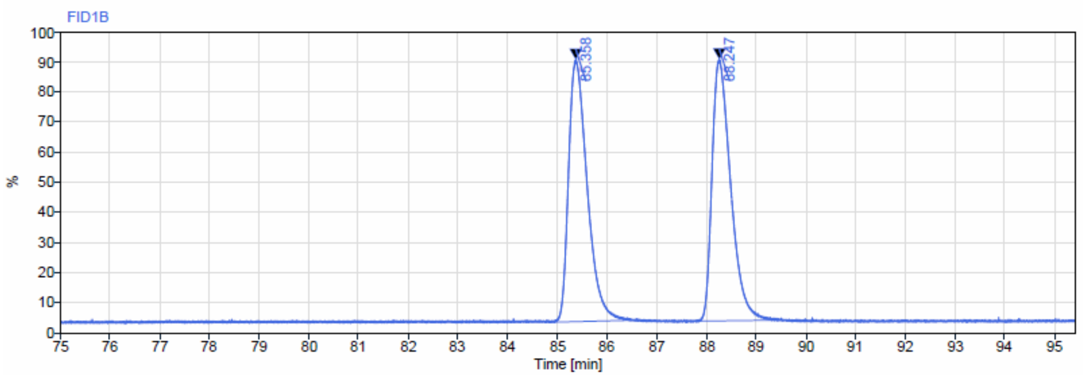

Signal:	FID1B					
**RT [min]**	**Type**	**Width [min]**	**Area**	**Height**	**Area%**	**Name**
85.358	MM m	1.80	73.04	2.80	50.07	
88.247	MM m	1.59	72.85	2.80	49.93	
		**Sum**	**145.89**			

Figure S4. (*R*)-2-Cyclohexylbutanenitrile (**2u**)

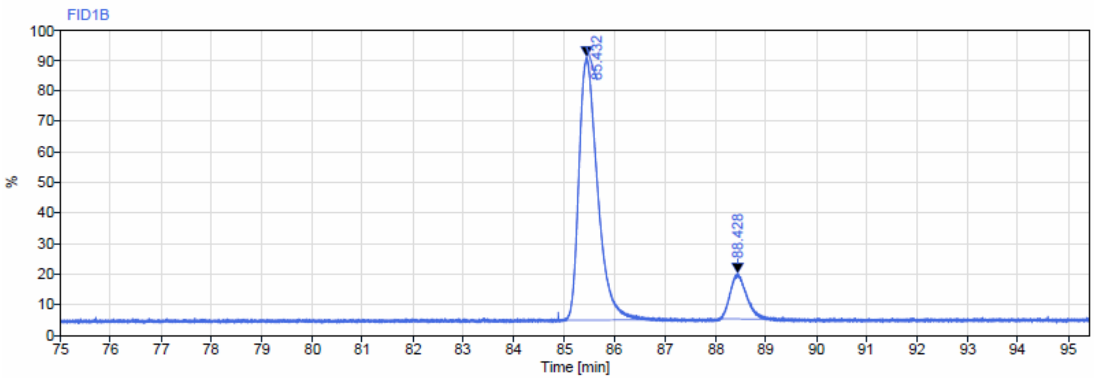

Signal:	FID1B					
**RT [min]**	**Type**	**Width [min]**	**Area**	**Height**	**Area%**	**Name**
85.432	MM m	1.85	48.70	1.90	87.01	
88.428	MM m	0.99	7.27	0.32	12.99	
		**Sum**	**55.97**			

6 NMR Spectra of Starting Materials and Products

Figure S5. ^{1}H NMR (500 MHz, $CDCl_3$) of **1a**.

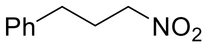

1a

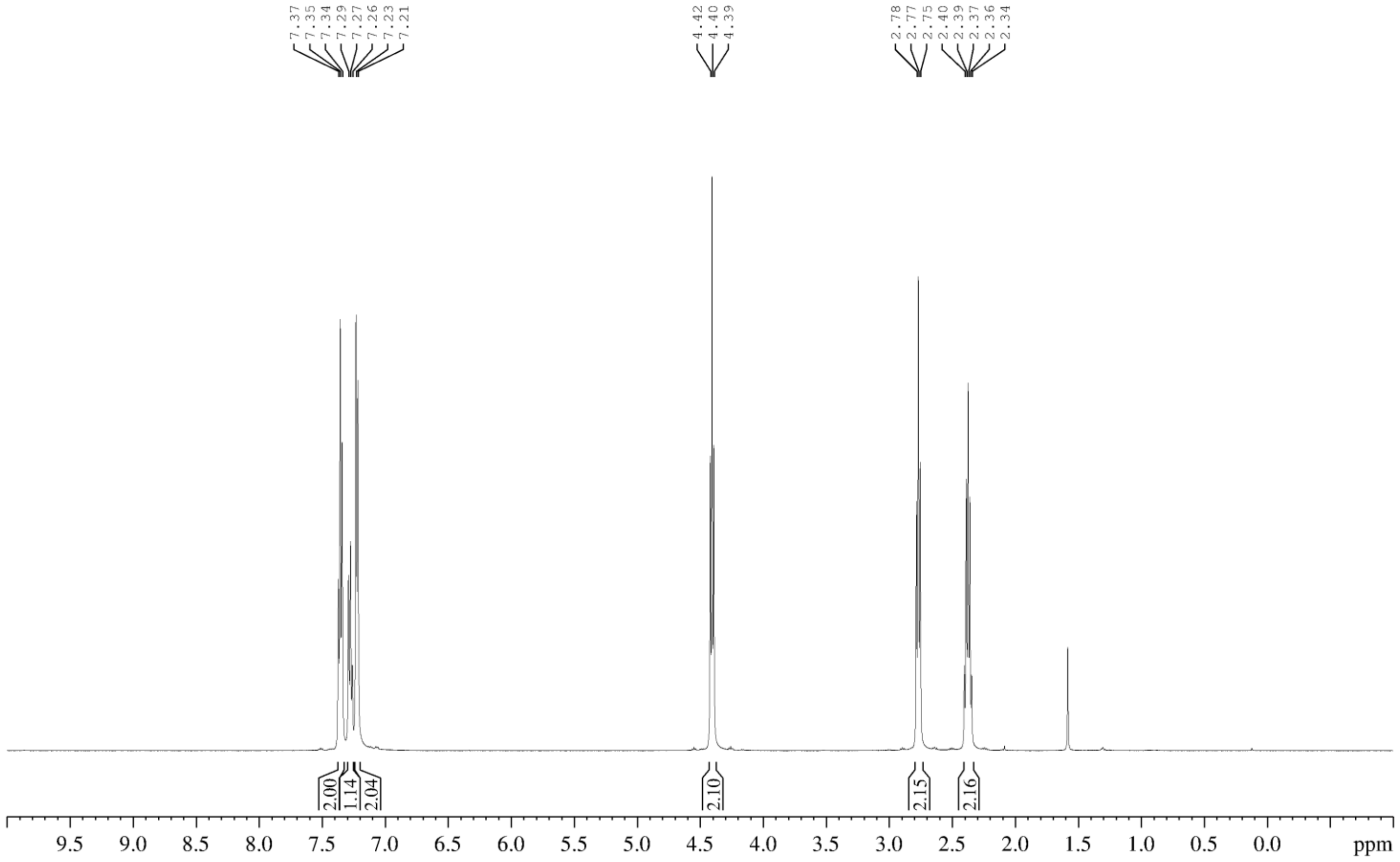

Figure S6. ^{13}**C NMR** (126 MHz, $CDCl_3$) of **1a**.

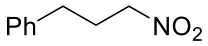

1a

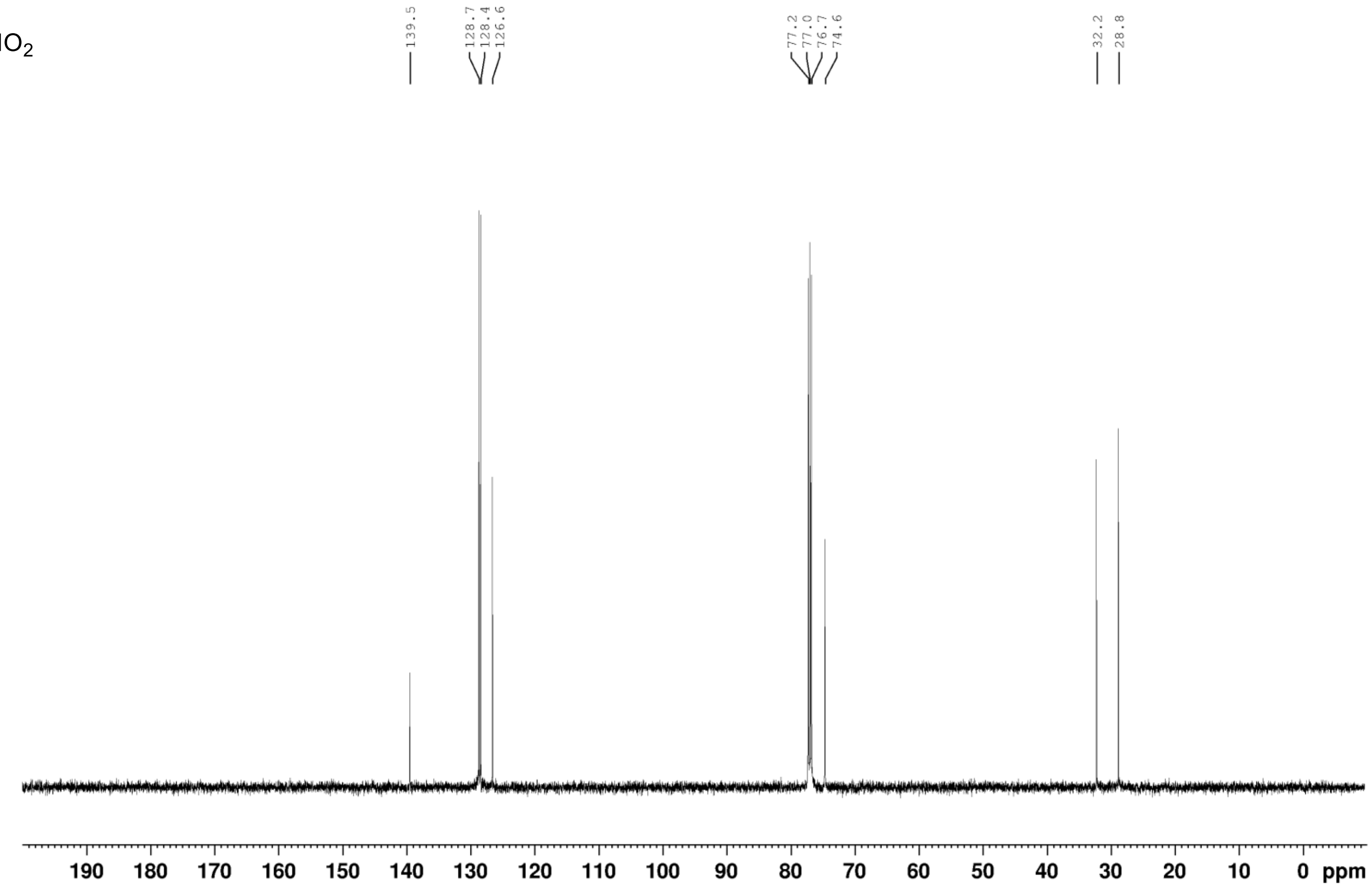

Figure S7. **^{1}H NMR** (400 MHz, $CDCl_3$) of **1b**.

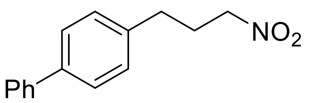

1b

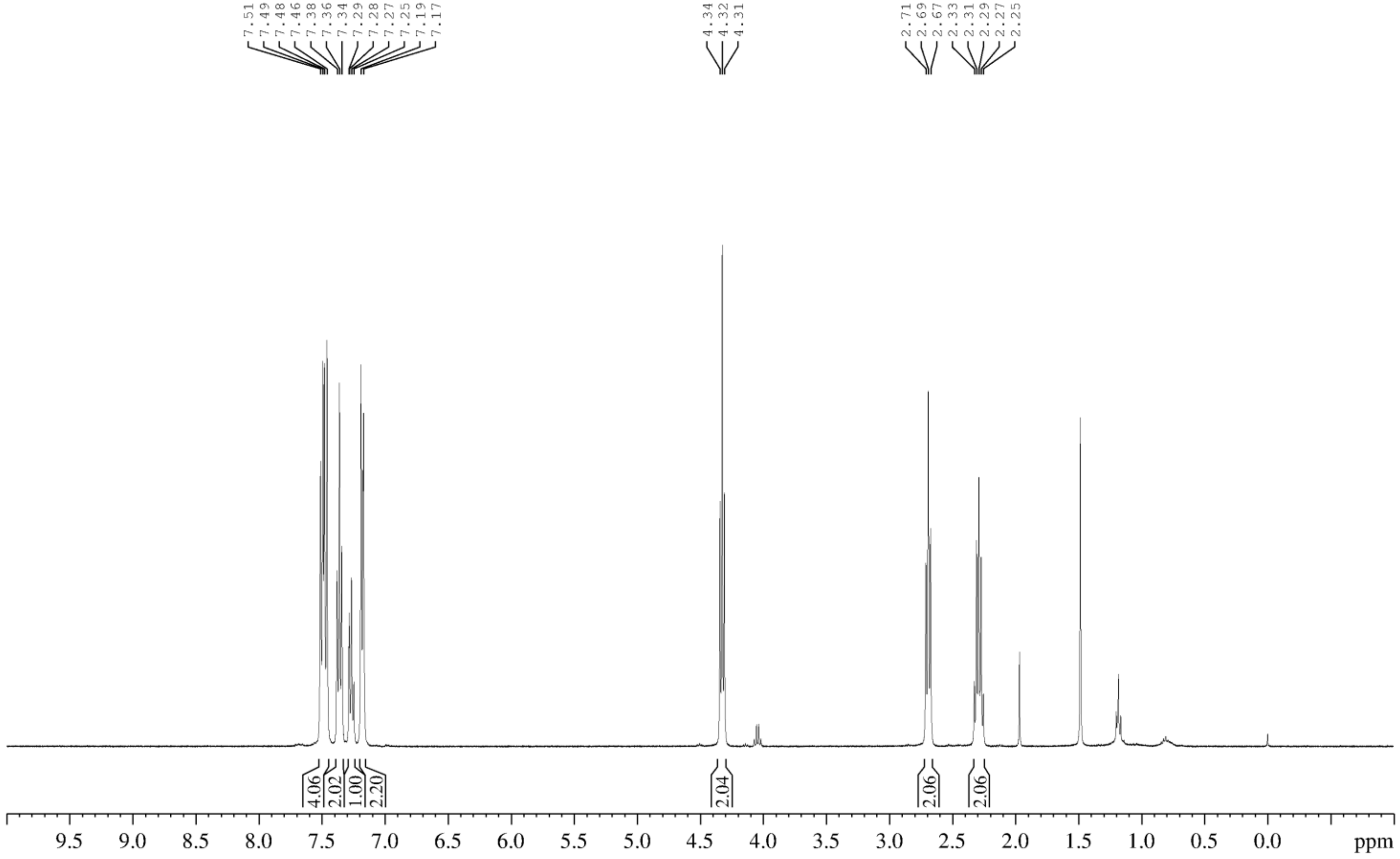

Figure S8. ¹³C NMR (101 MHz, $CDCl_3$) of **1b**.

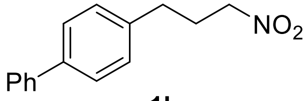

1b

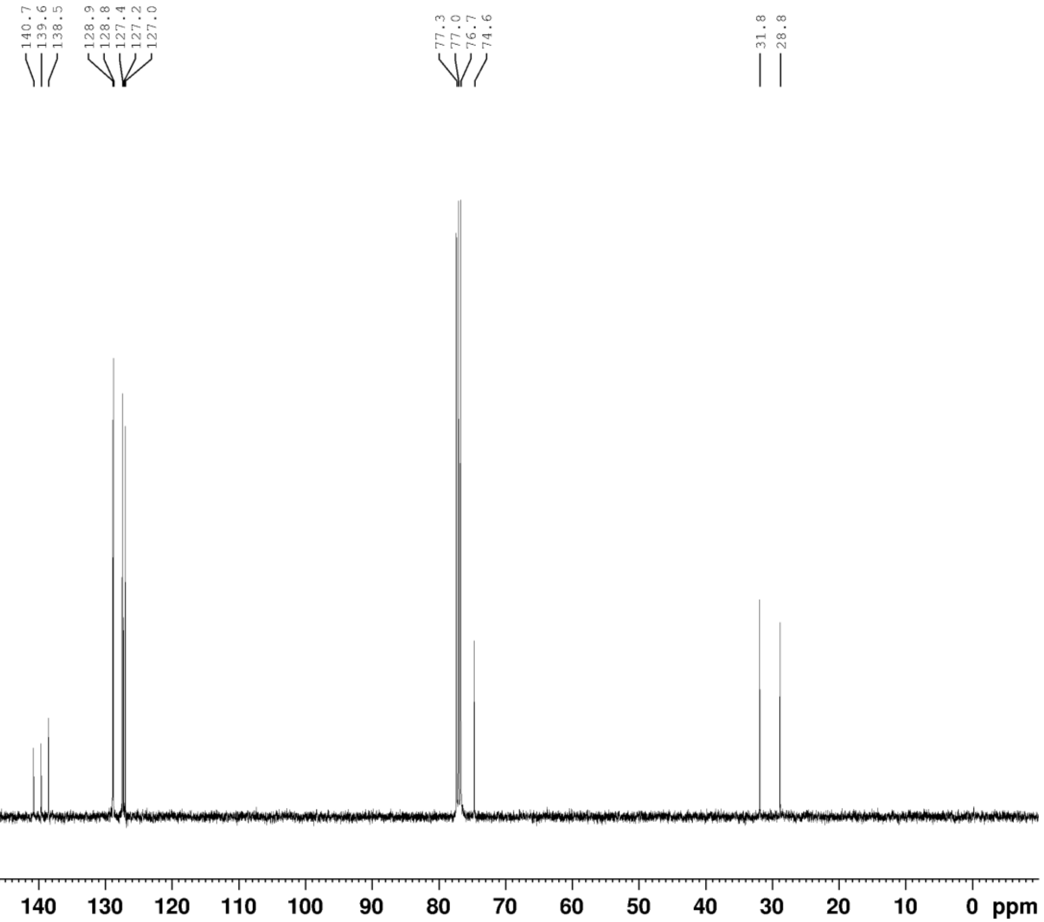

Figure S9. **^{1}H NMR** (500 MHz, $CDCl_3$) of **1c**.

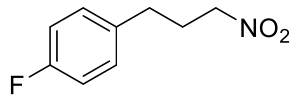

1c

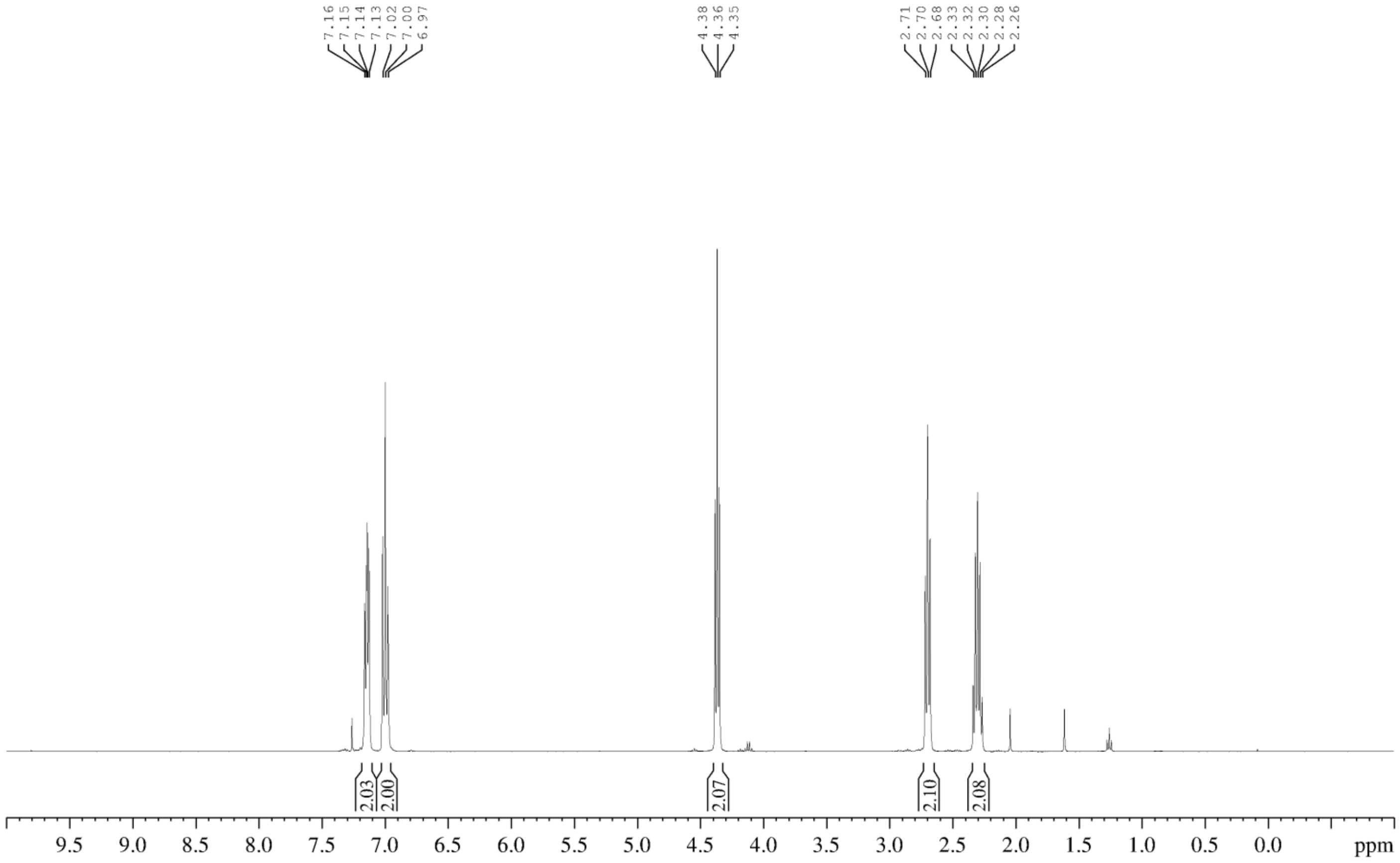

Figure S10. **^{13}C NMR** (126 MHz, $CDCl_3$) of **1c**.

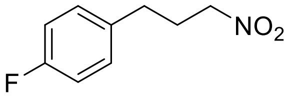

1c

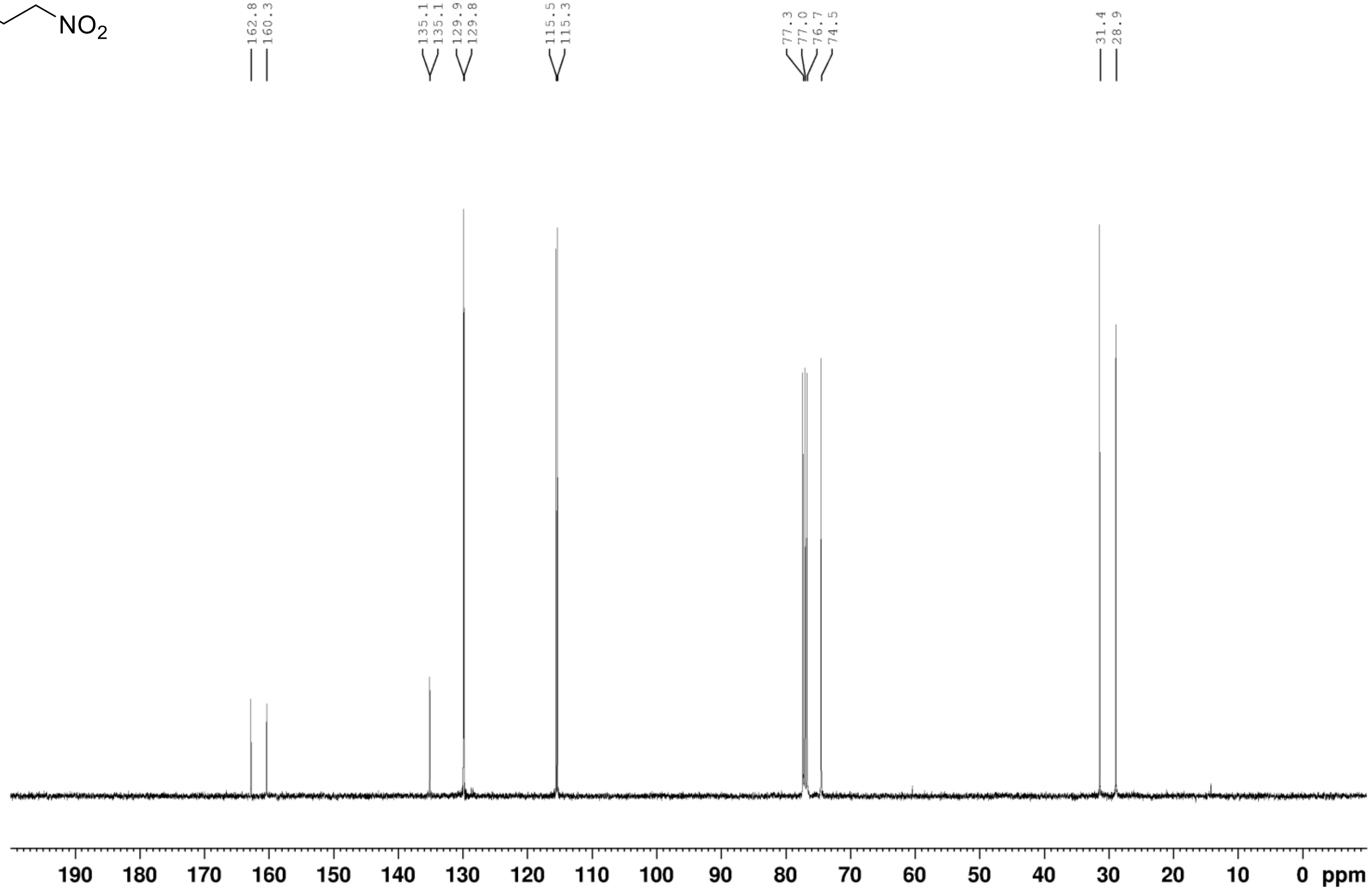

Figure S11. ^{19}**F NMR** (471 MHz, $CDCl_3$, 298 K) of **1c**.

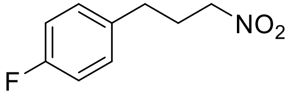

1c

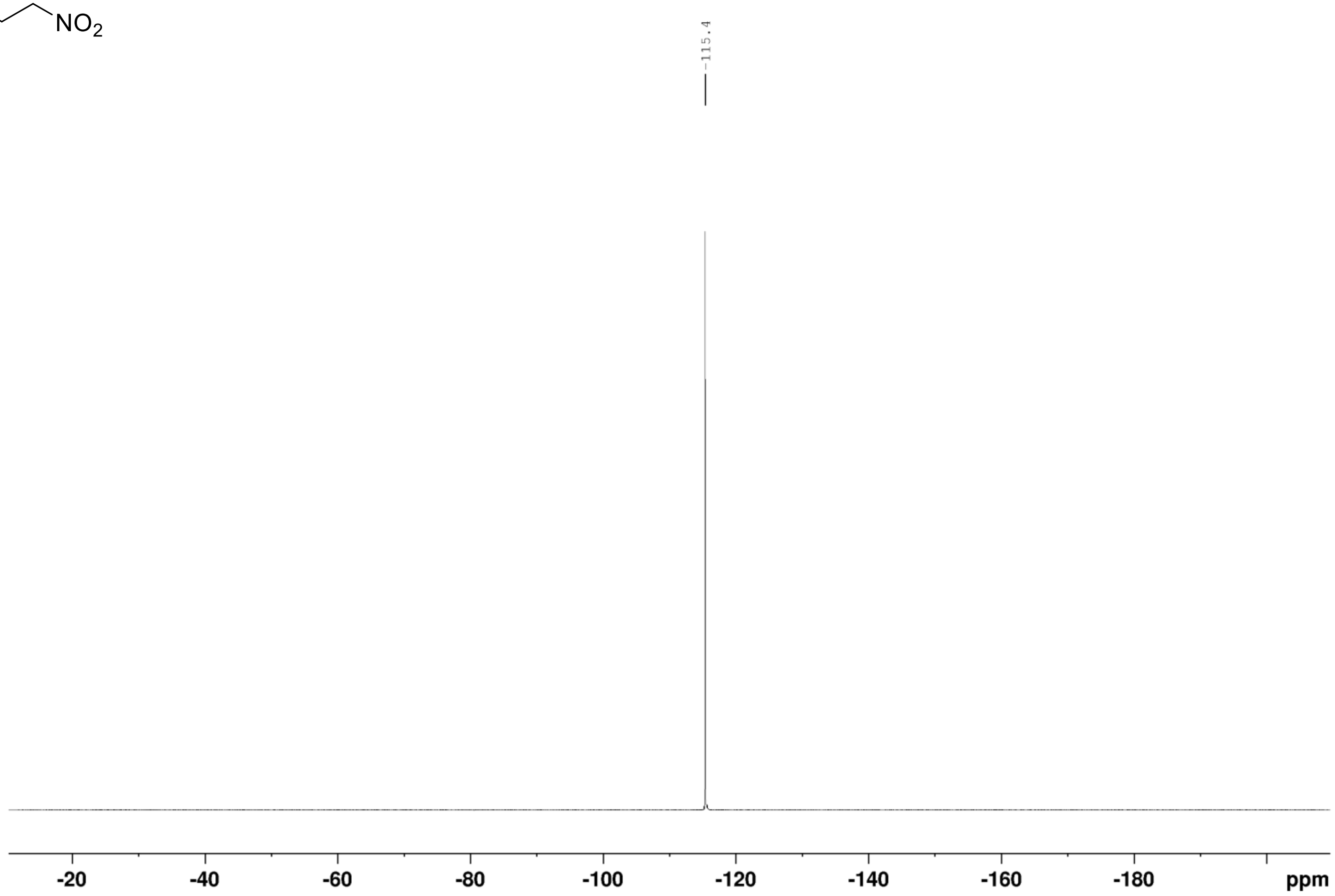

Figure S12. ^{1}**H NMR** (400 MHz, $CDCl_3$) of **1d**.

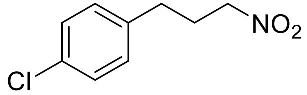

1d

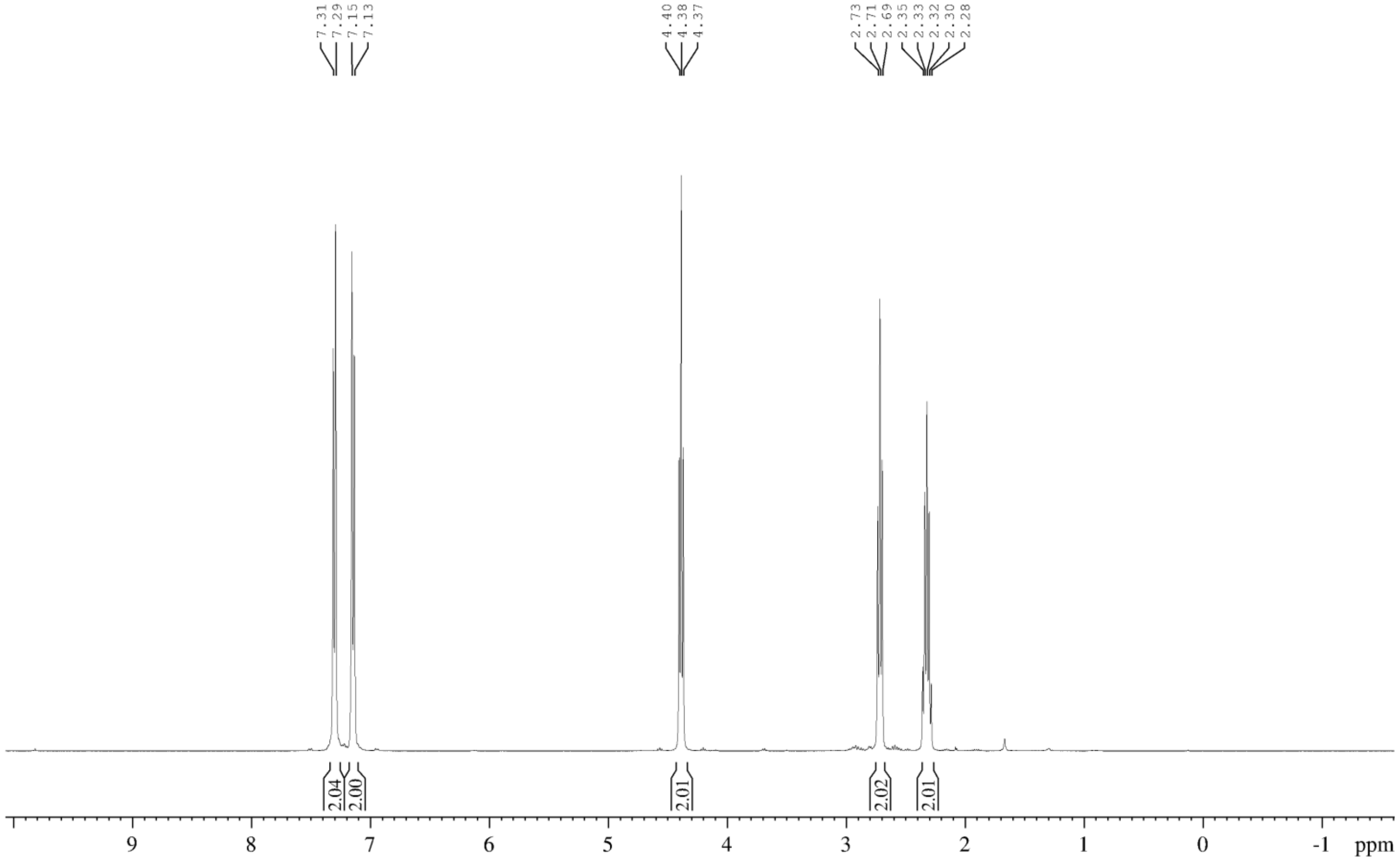

Figure S13. ^{13}**C NMR** (101 MHz, $CDCl_3$) of **1d**.

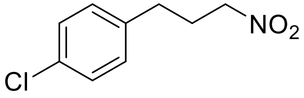

1d

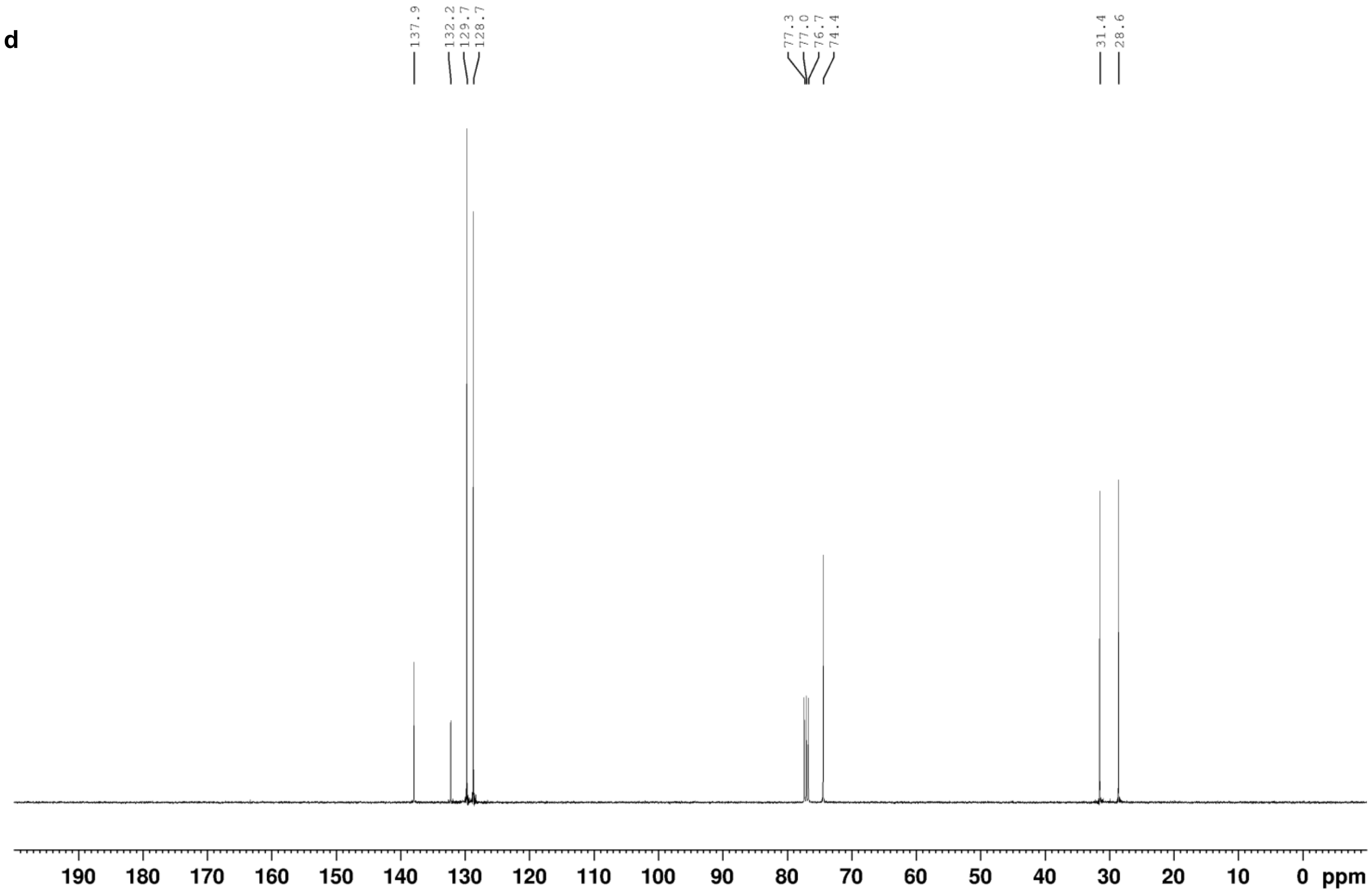

Figure S14. **^{1}H NMR** (400 MHz, $CDCl_3$) of **1e**.

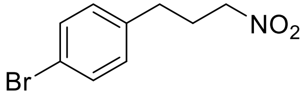

1e

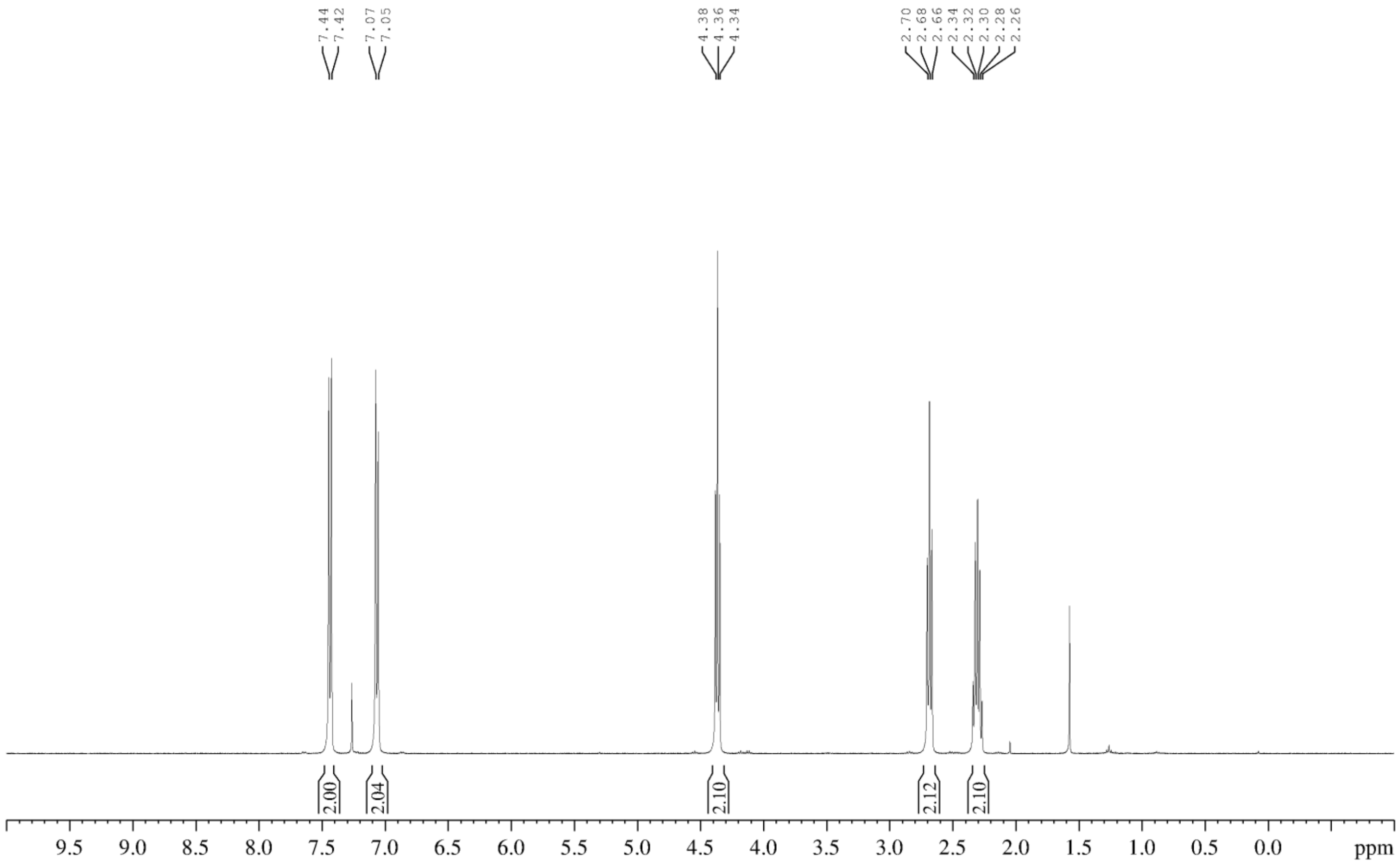

Figure S15. ^{13}**C NMR** (101 MHz, $CDCl_3$) of **1e**.

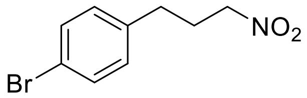

1e

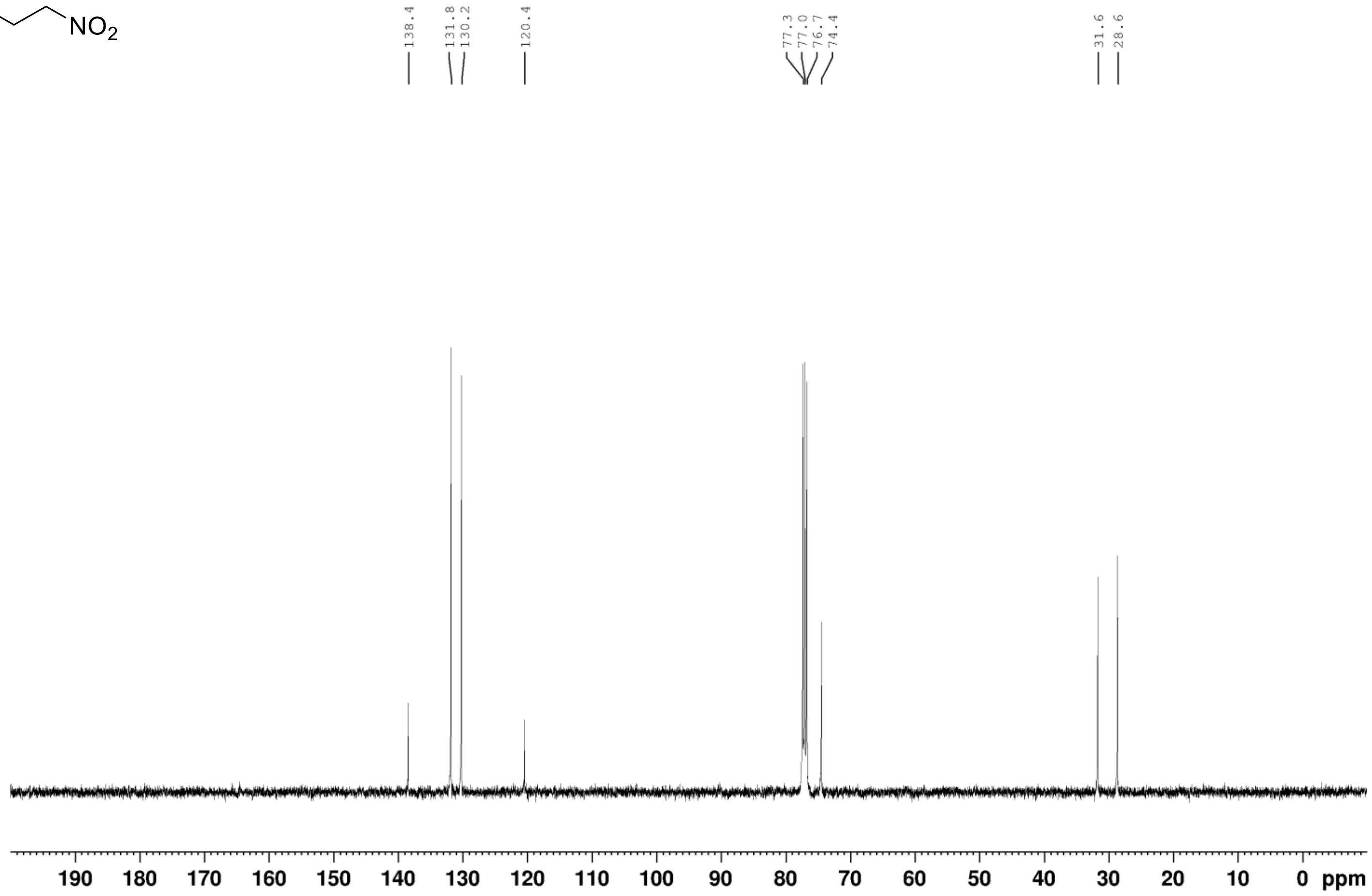

Figure S16. **^{1}H NMR** (400 MHz, $CDCl_3$) of **1f**.

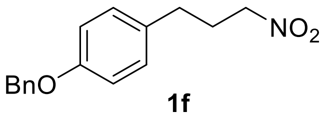

1f

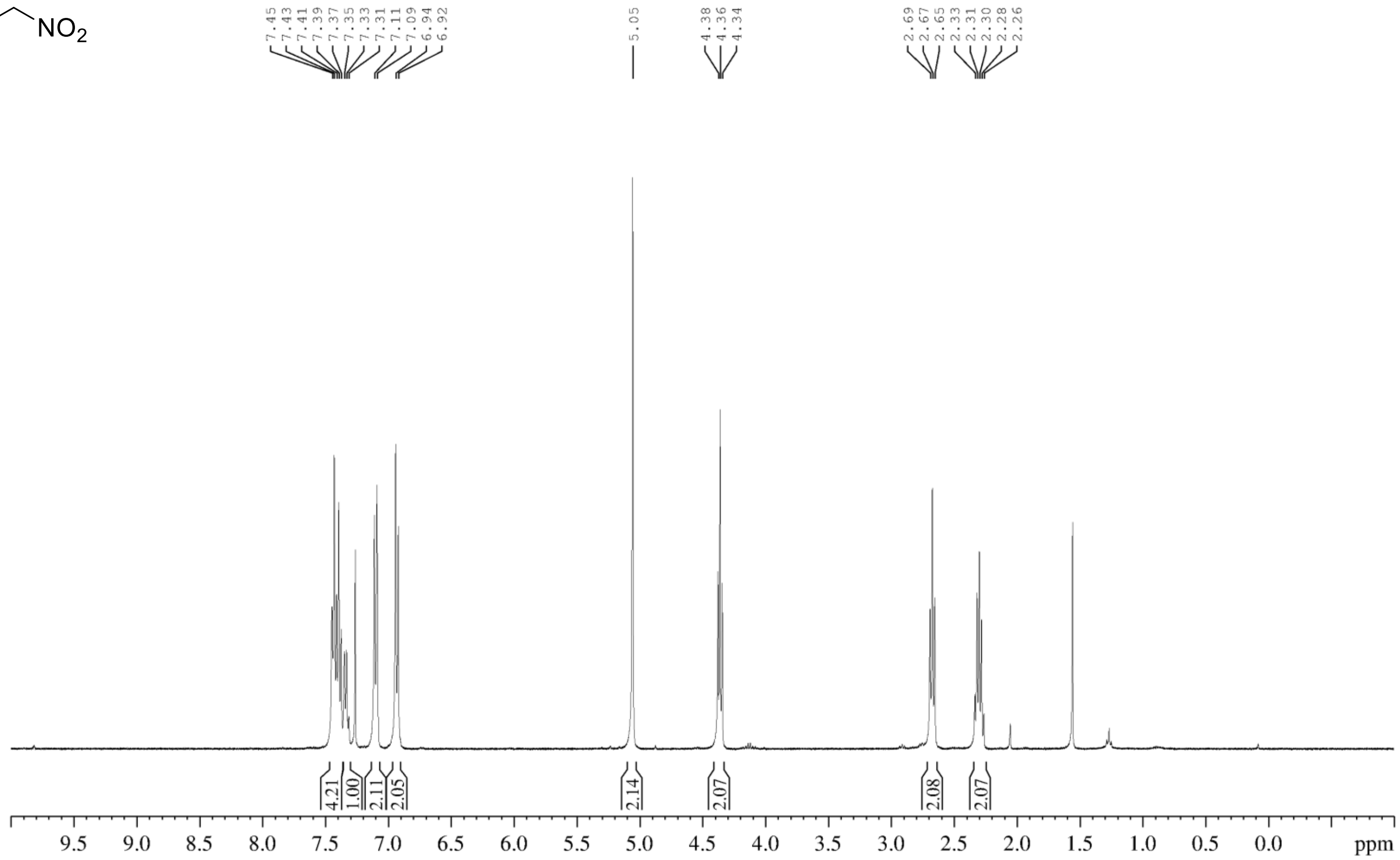

Figure S17. ^{13}**C NMR** (101 MHz, $CDCl_3$) of **1f**.

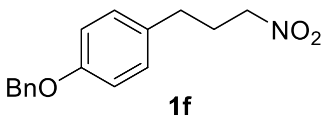

1f

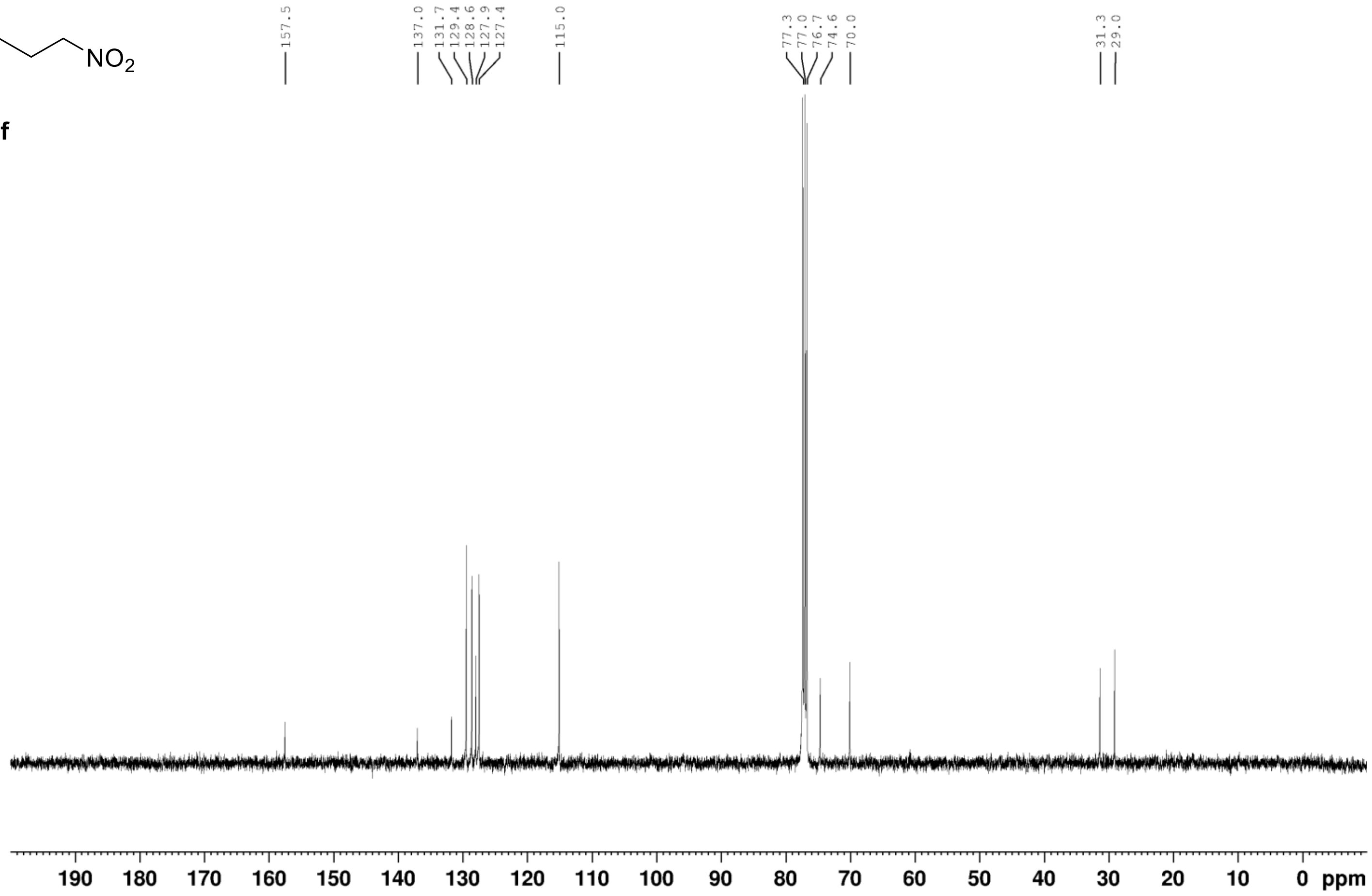

Figure S18. **^{1}H NMR** (400 MHz, $CDCl_3$) of **1g**.

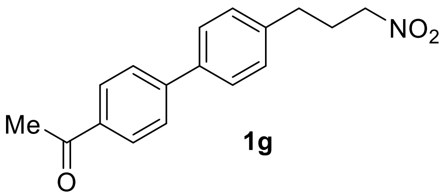

1g

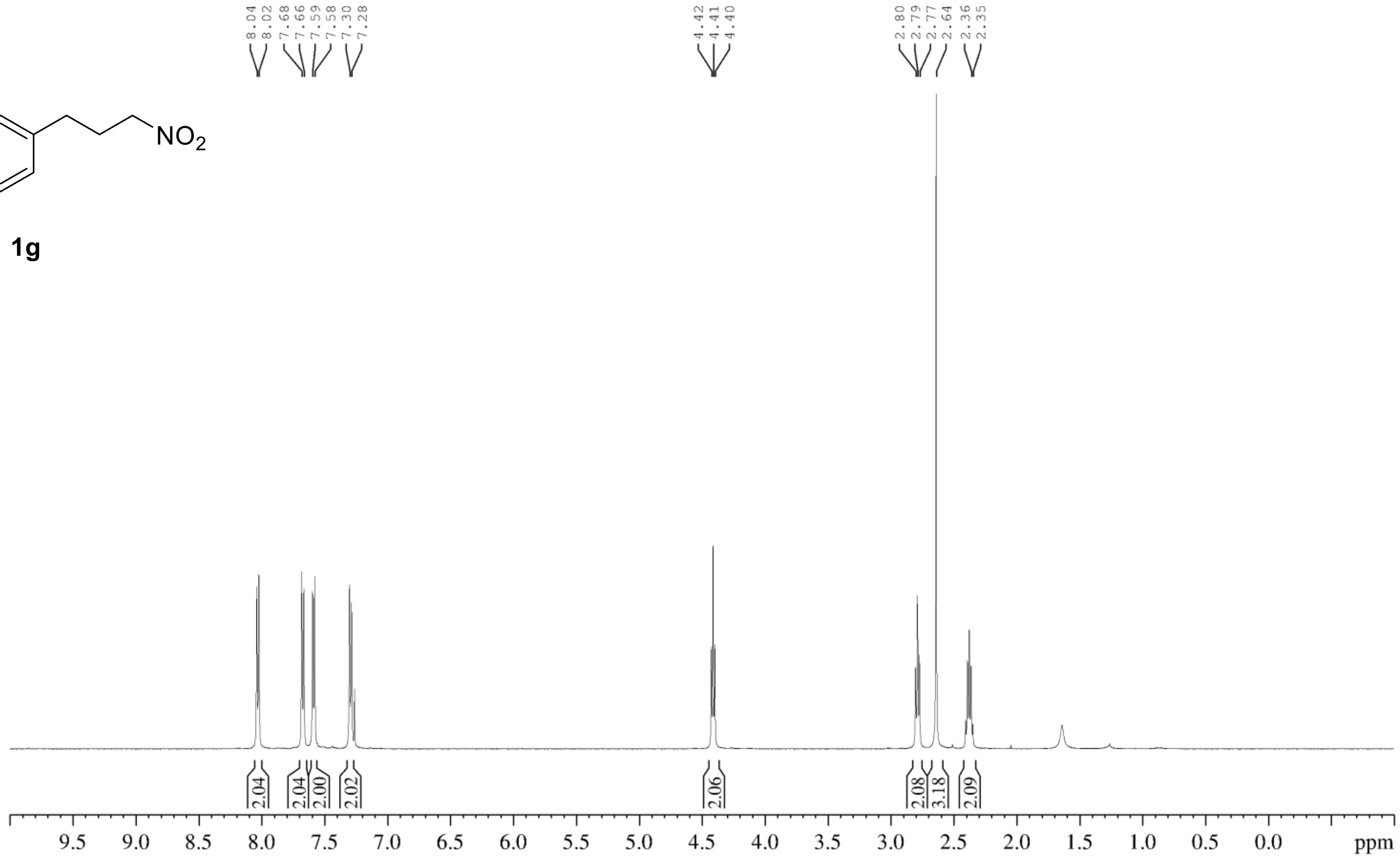

Figure S19. **^{13}C NMR** (101 MHz, $CDCl_3$) of **1g**.

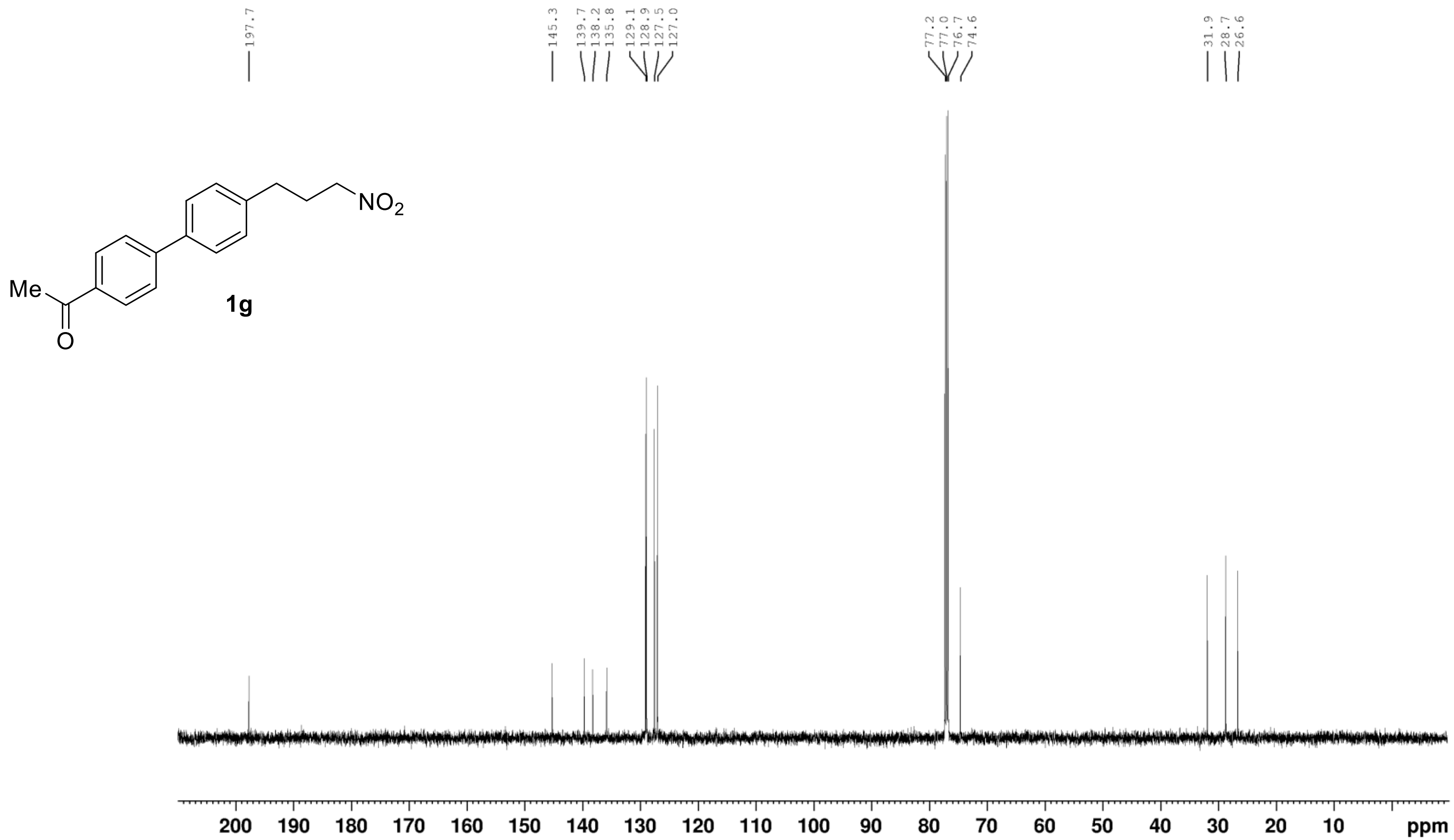

Figure S20. **^{1}H NMR** (500 MHz, $CDCl_3$) of **1i-xiNO2**.

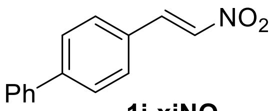

1i-xiNO$_2$

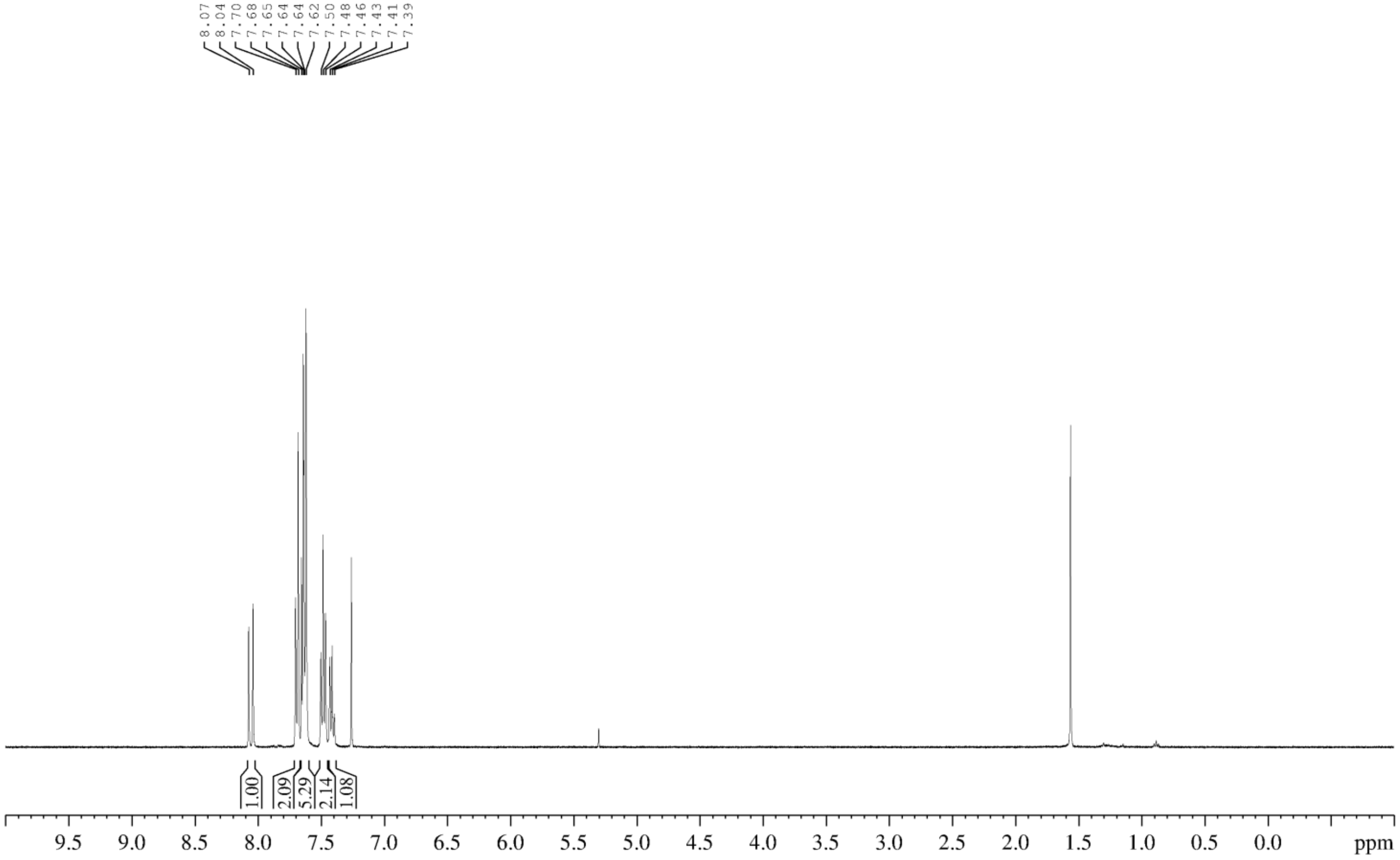

Figure S21. **^{13}C NMR** (126 MHz, $CDCl_3$) of **1i-xiNO2**.

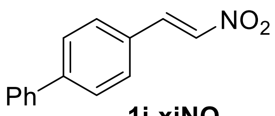

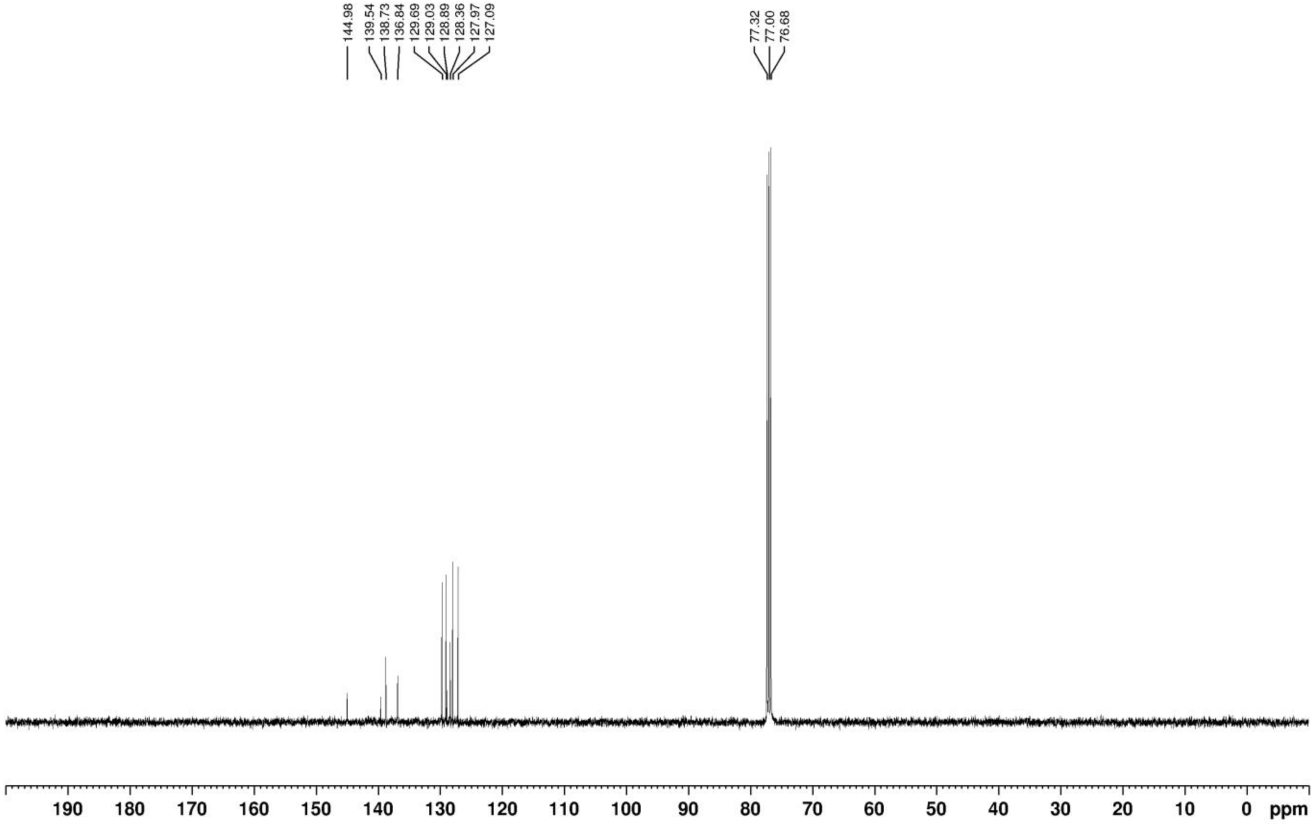

Figure S22. **^{1}H NMR** (500 MHz, $CDCl_3$) of **1i**.

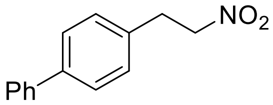

1i

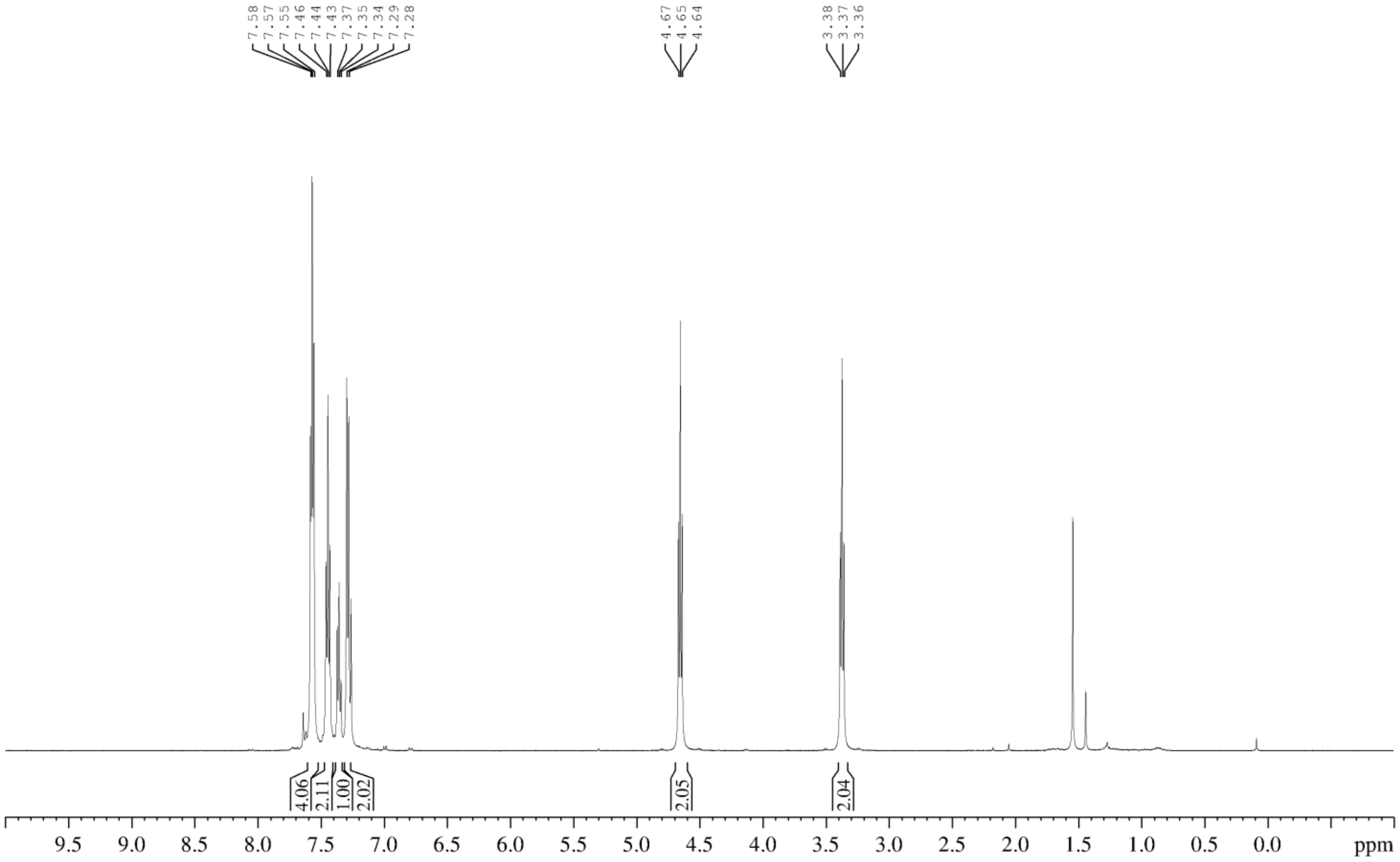

Figure S23. **^{13}C NMR** (126 MHz, $CDCl_3$) of **1i**.

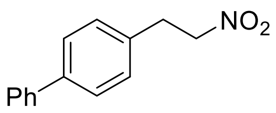

1i

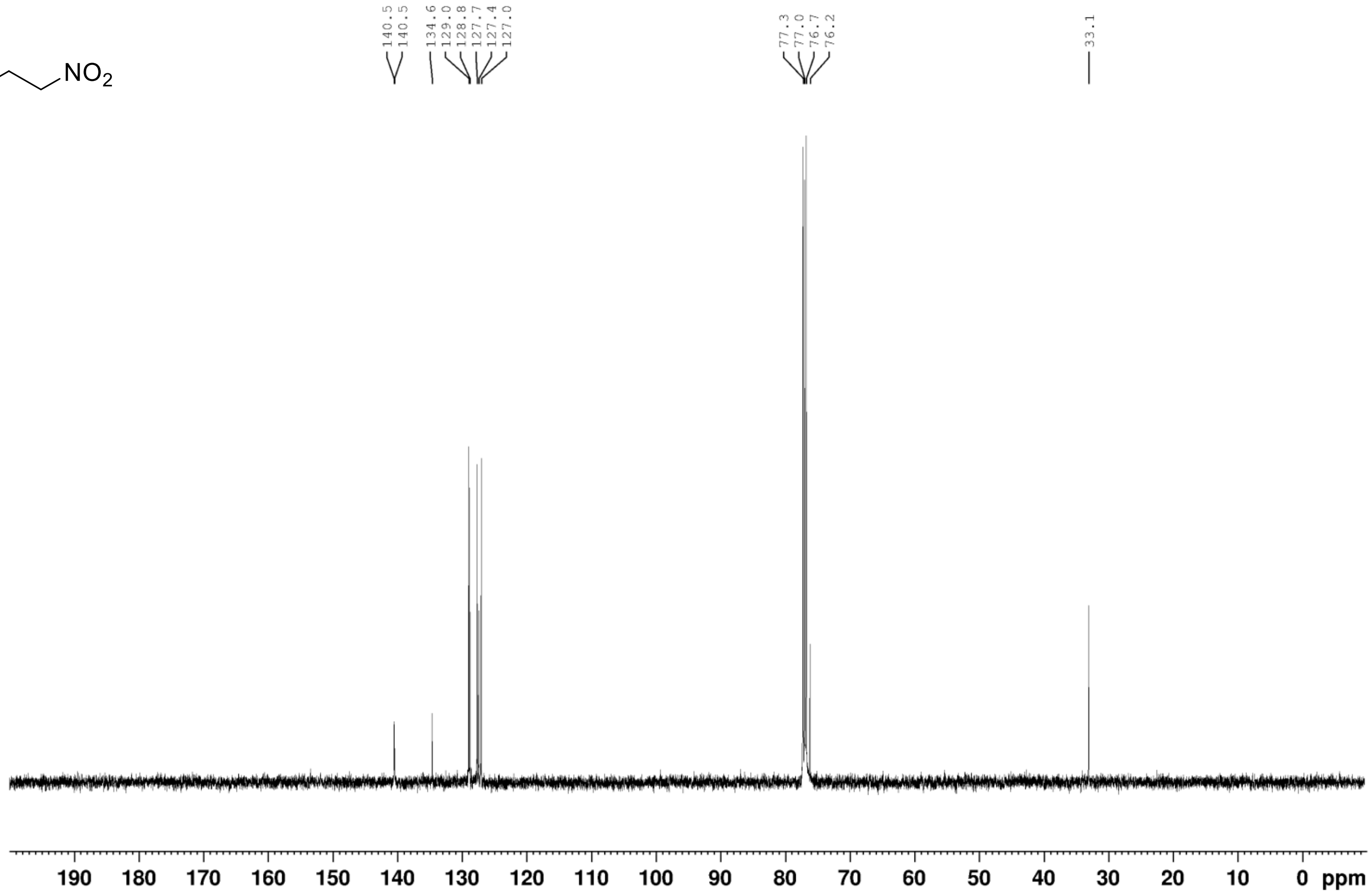

Figure S24. **^{1}H NMR** (400 MHz, $CDCl_3$) of **1j**.

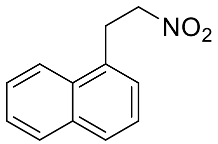

1j

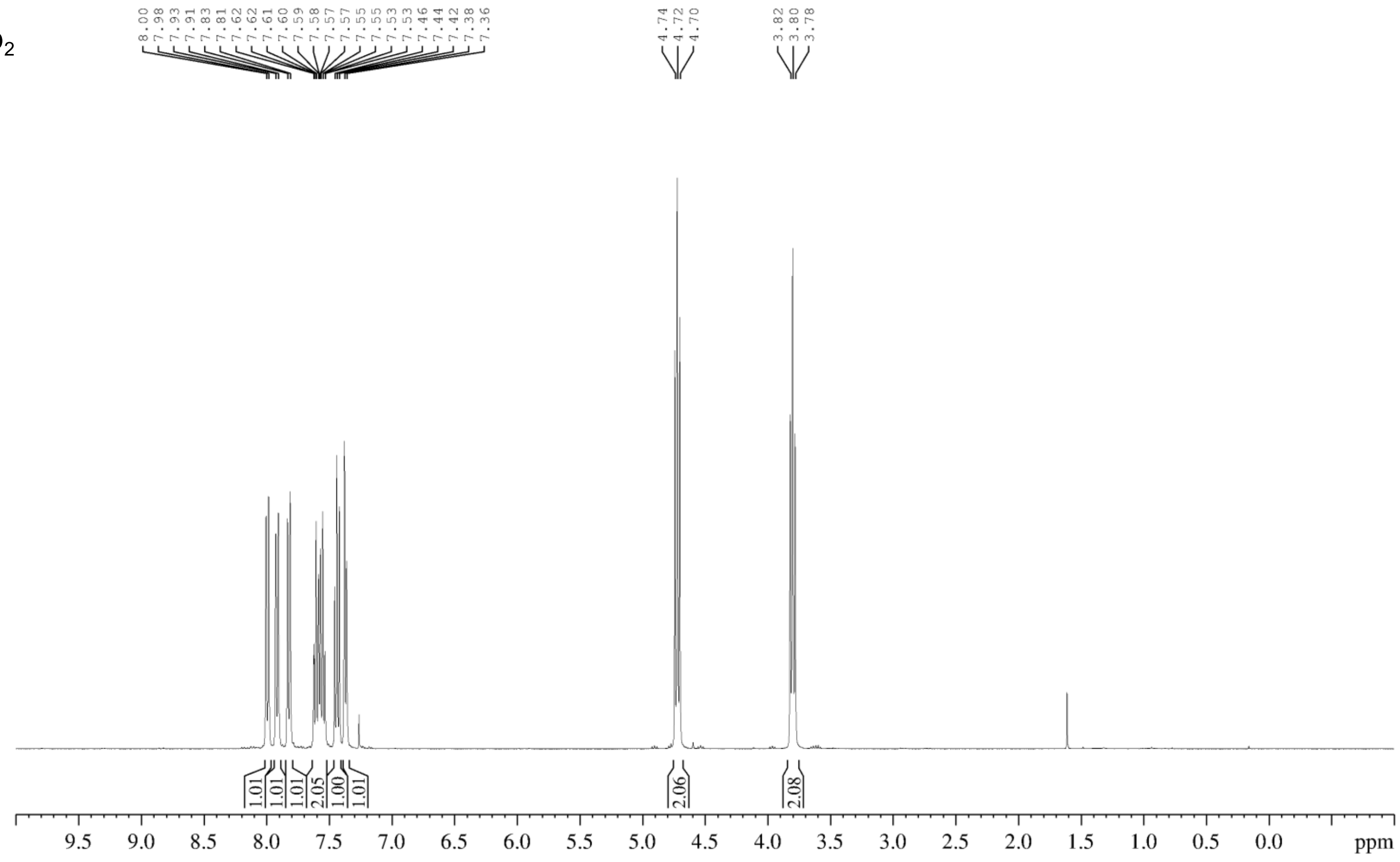

Figure S25. ^{13}C **NMR** (101 MHz, $CDCl_3$) of **1j**.

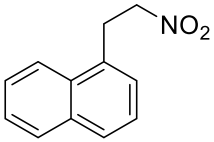

1j

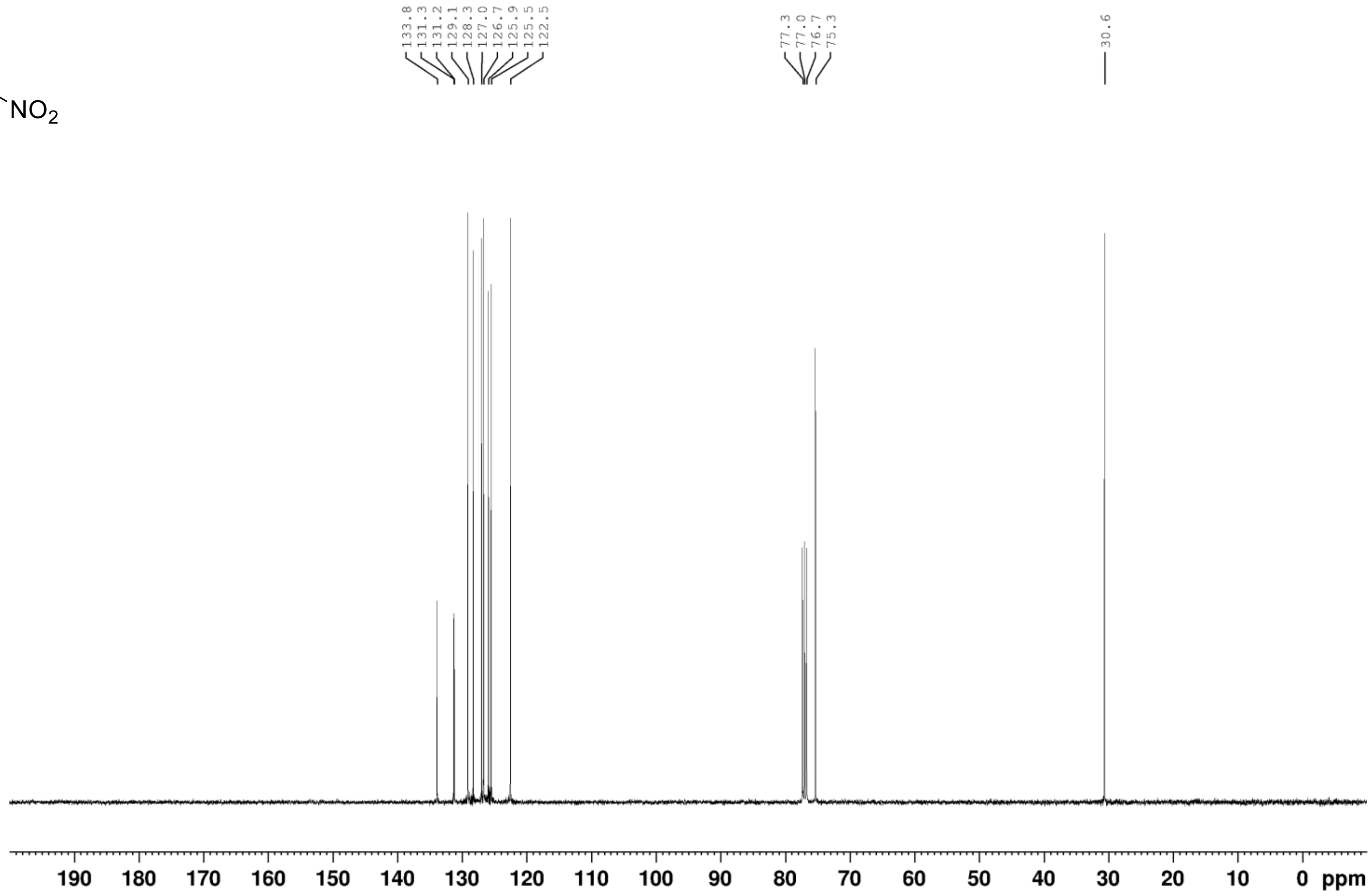

Figure S26. **^{1}H NMR** (400 MHz, $CDCl_3$) of **1k**.

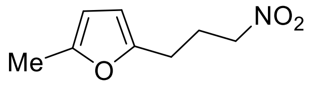

1k

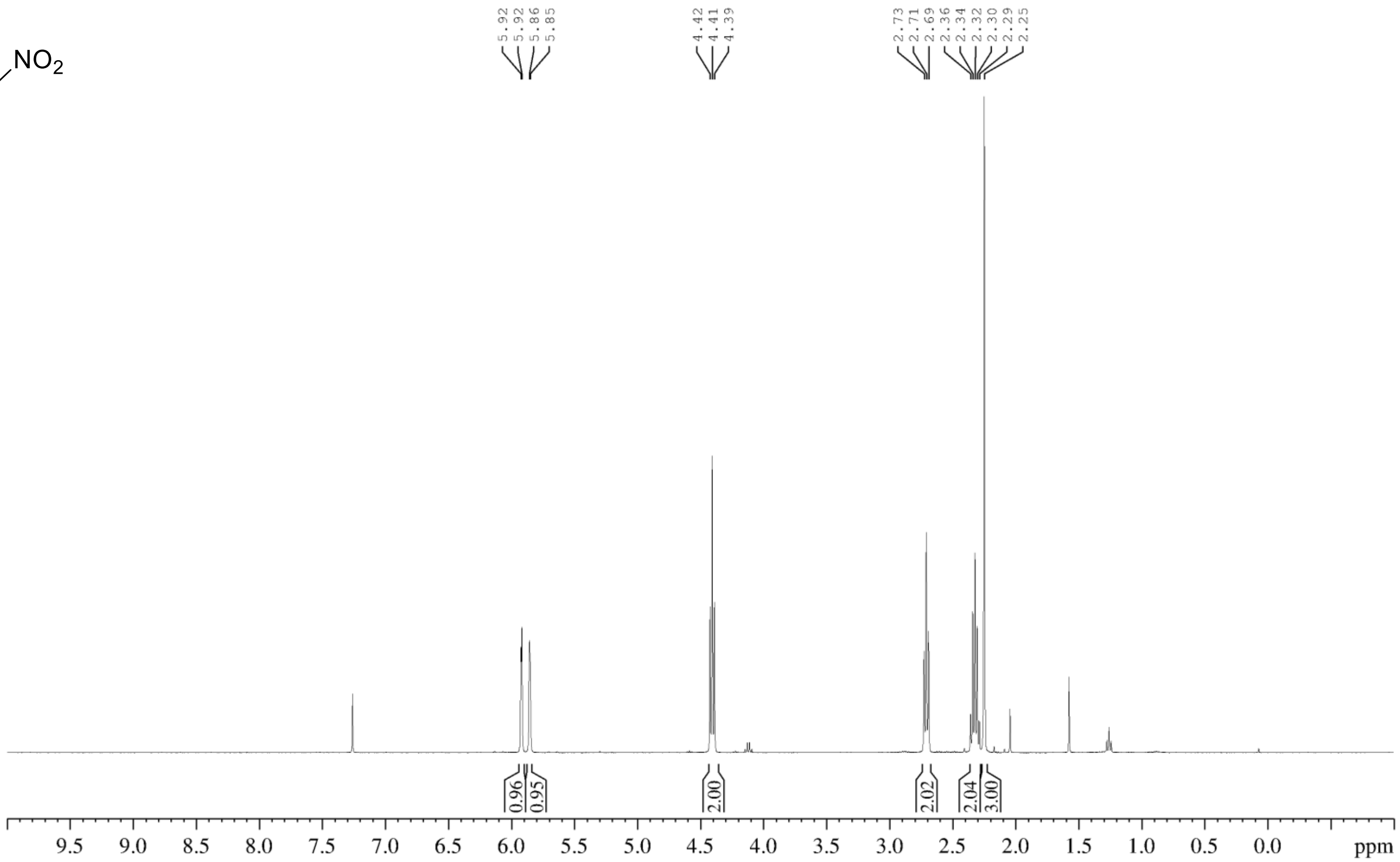

Figure S27. **^{13}C NMR** (101 MHz, $CDCl_3$) of **1k**.

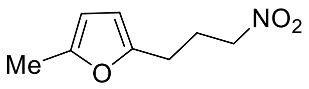

1k

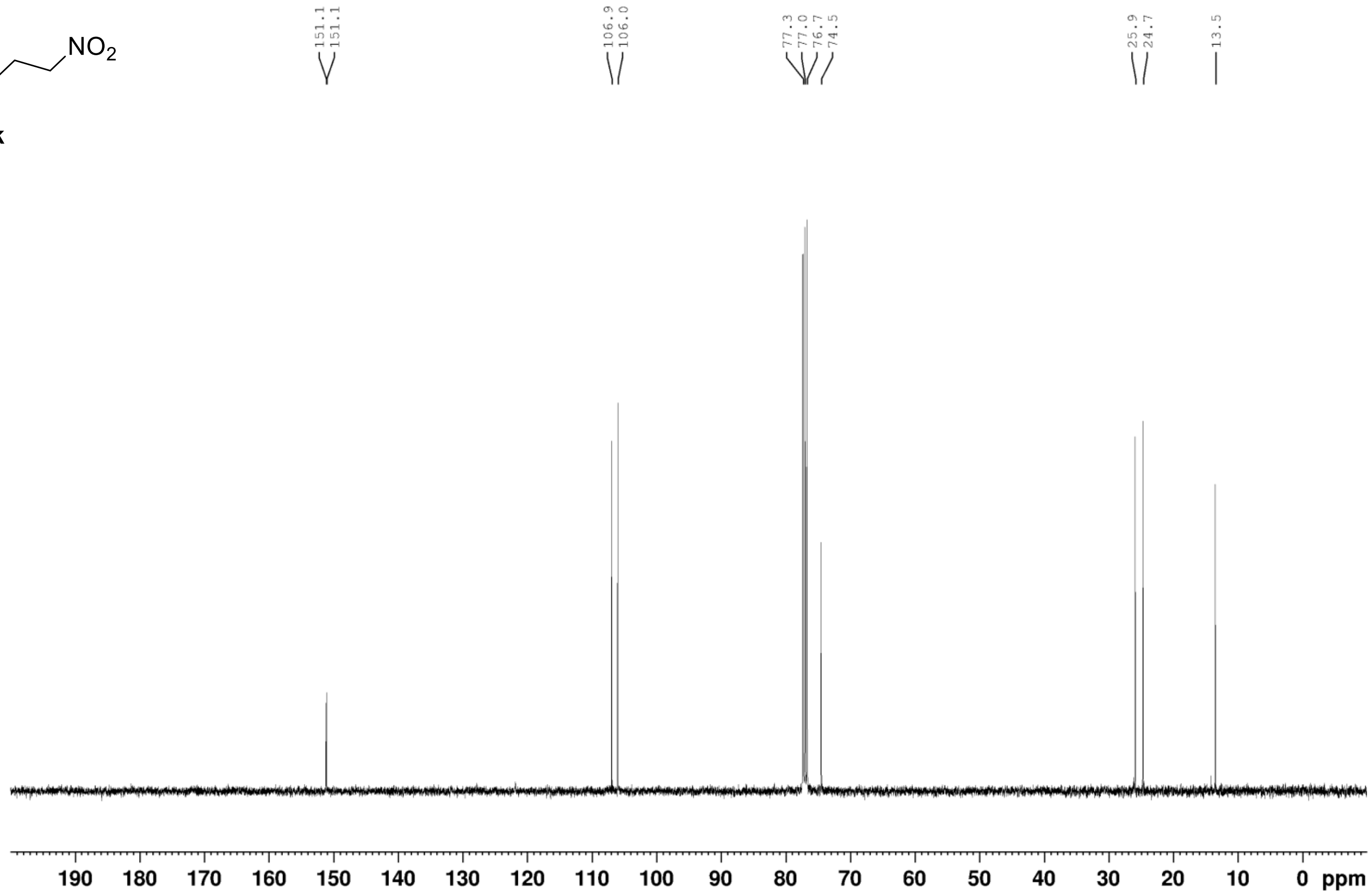

Figure S28. **^{1}H NMR** (400 MHz, $CDCl_3$) of **1l**.

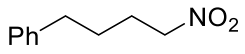

1l

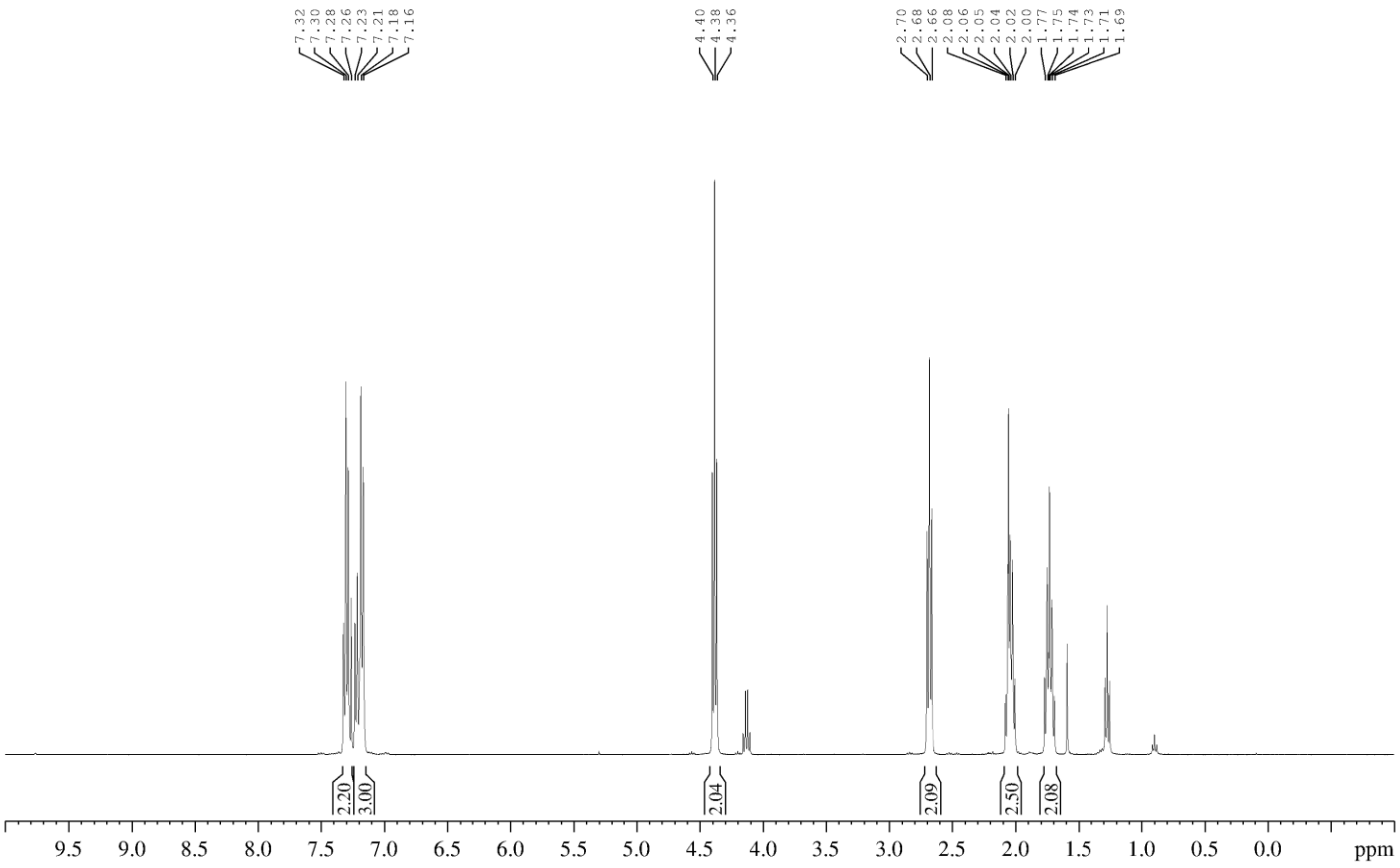

Figure S29. ^{13}C NMR (101 MHz, $CDCl_3$) of **1l**.

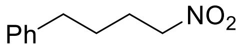

1l

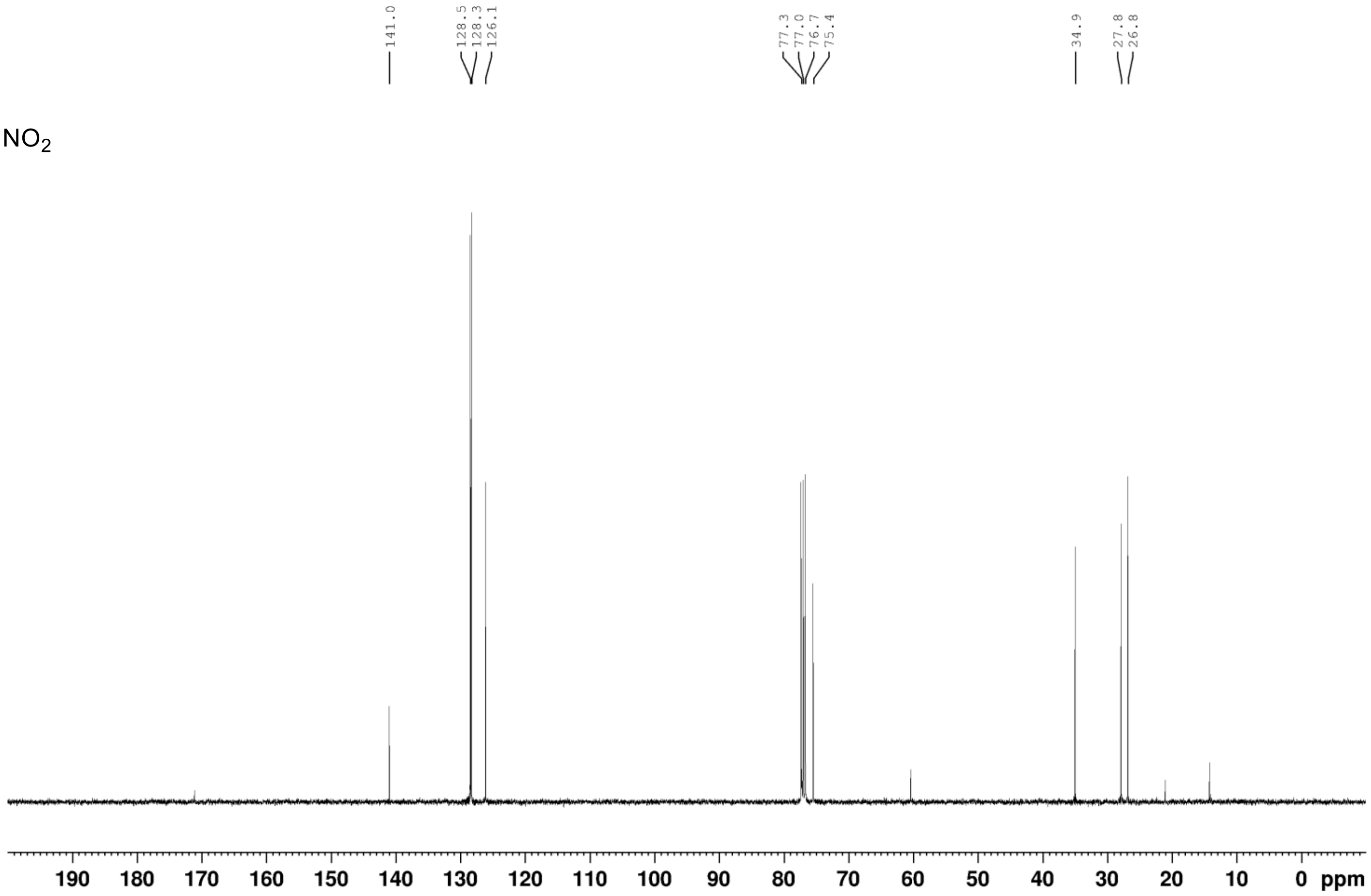

Figure S30. **^{1}H NMR** (400 MHz, $CDCl_3$) of **1m**.

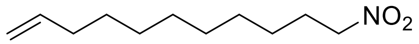

1m

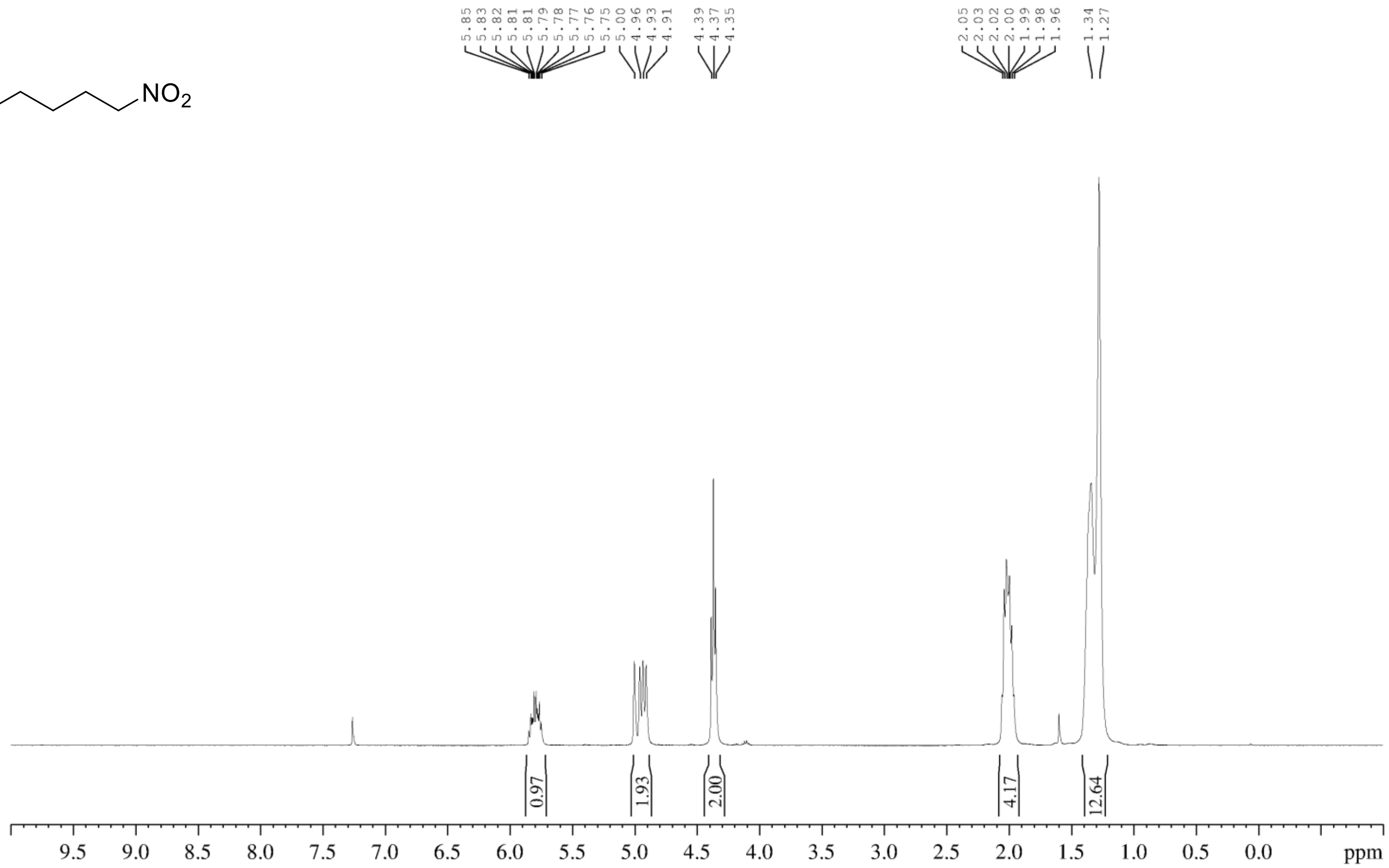

Figure S31. ^{13}**C NMR** (101 MHz, $CDCl_3$) of **1m**.

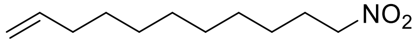

1m

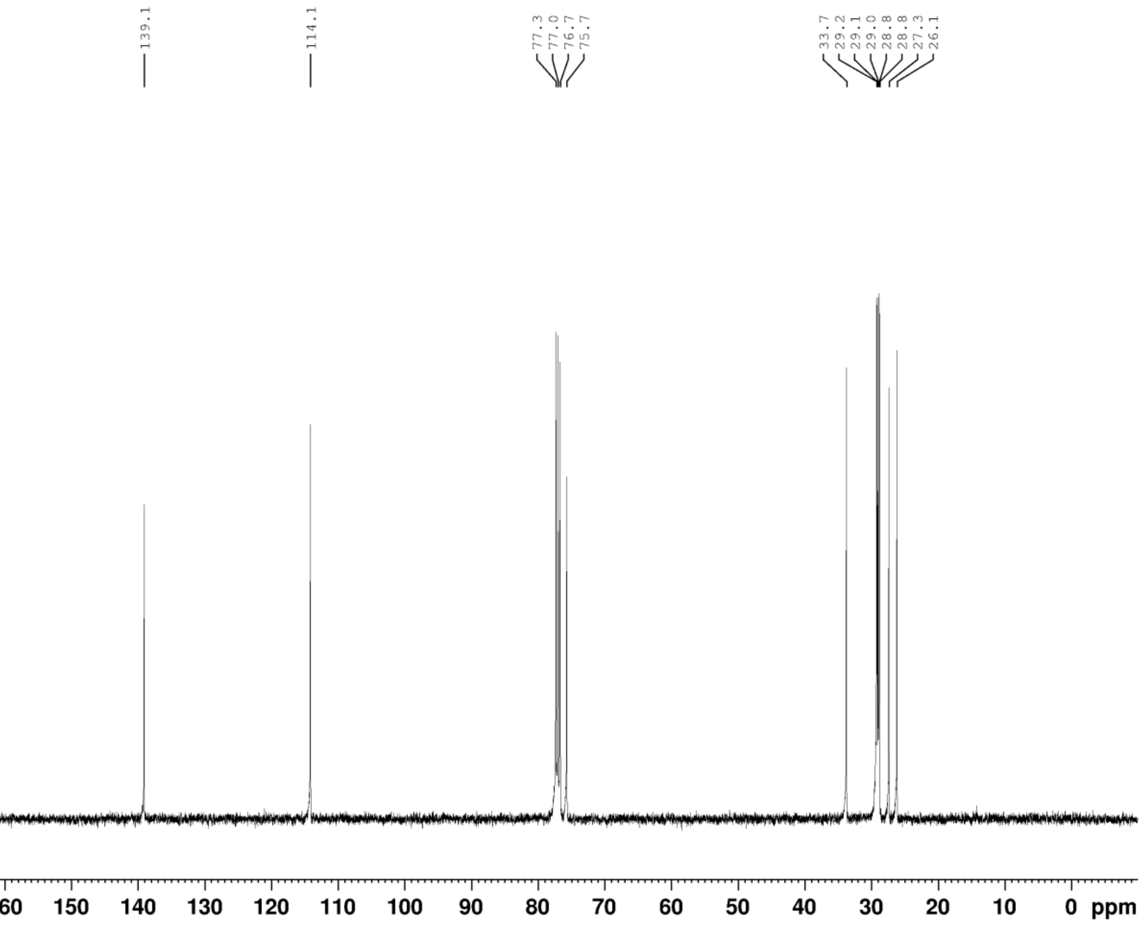

Figure S32. **¹H NMR** (400 MHz, $CDCl_3$) of **1n**.

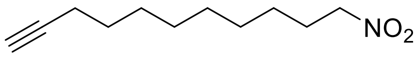

1n

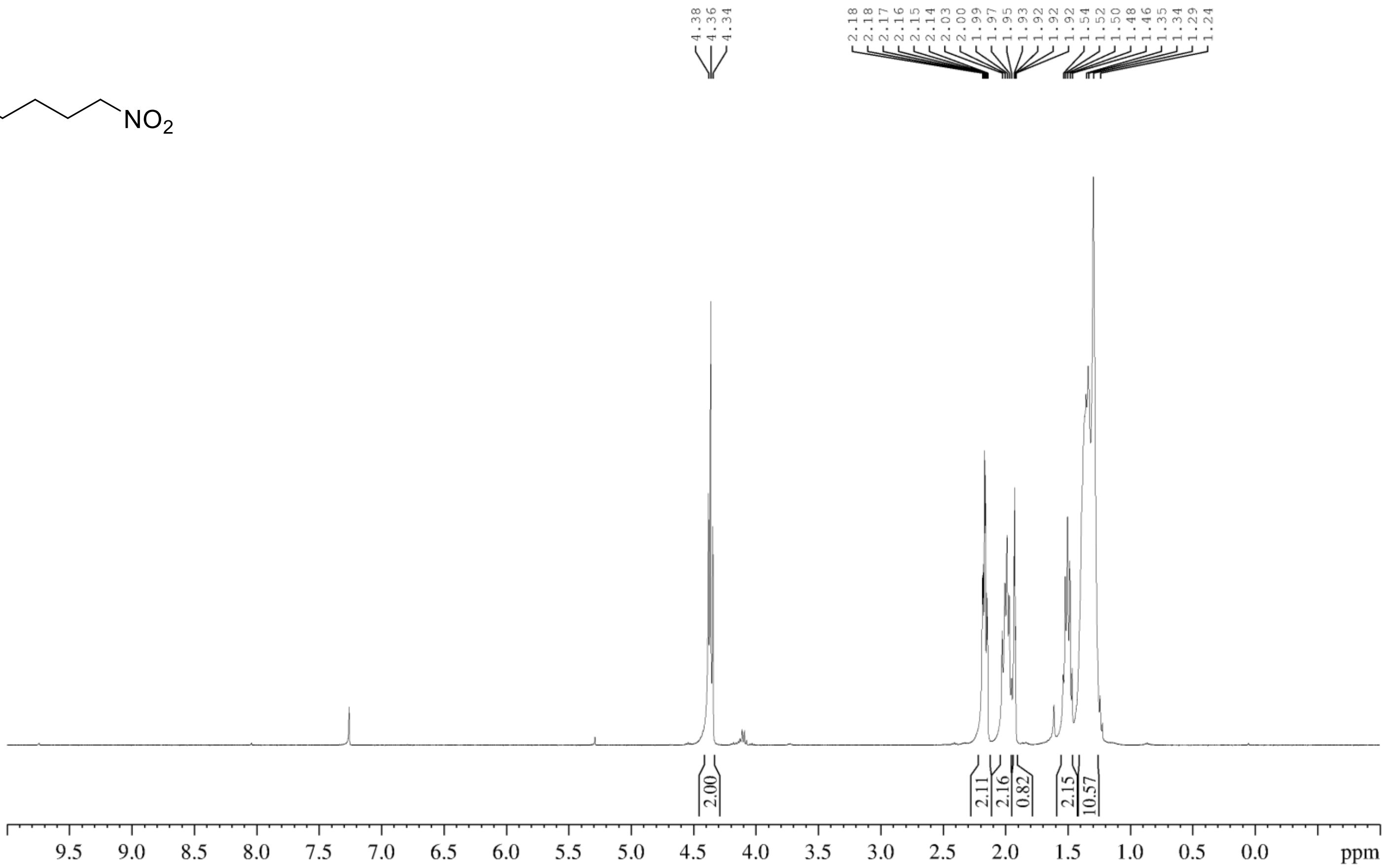

Figure S33. **^{13}C NMR** (101 MHz, $CDCl_3$) of **1n**.

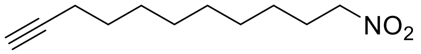

1n

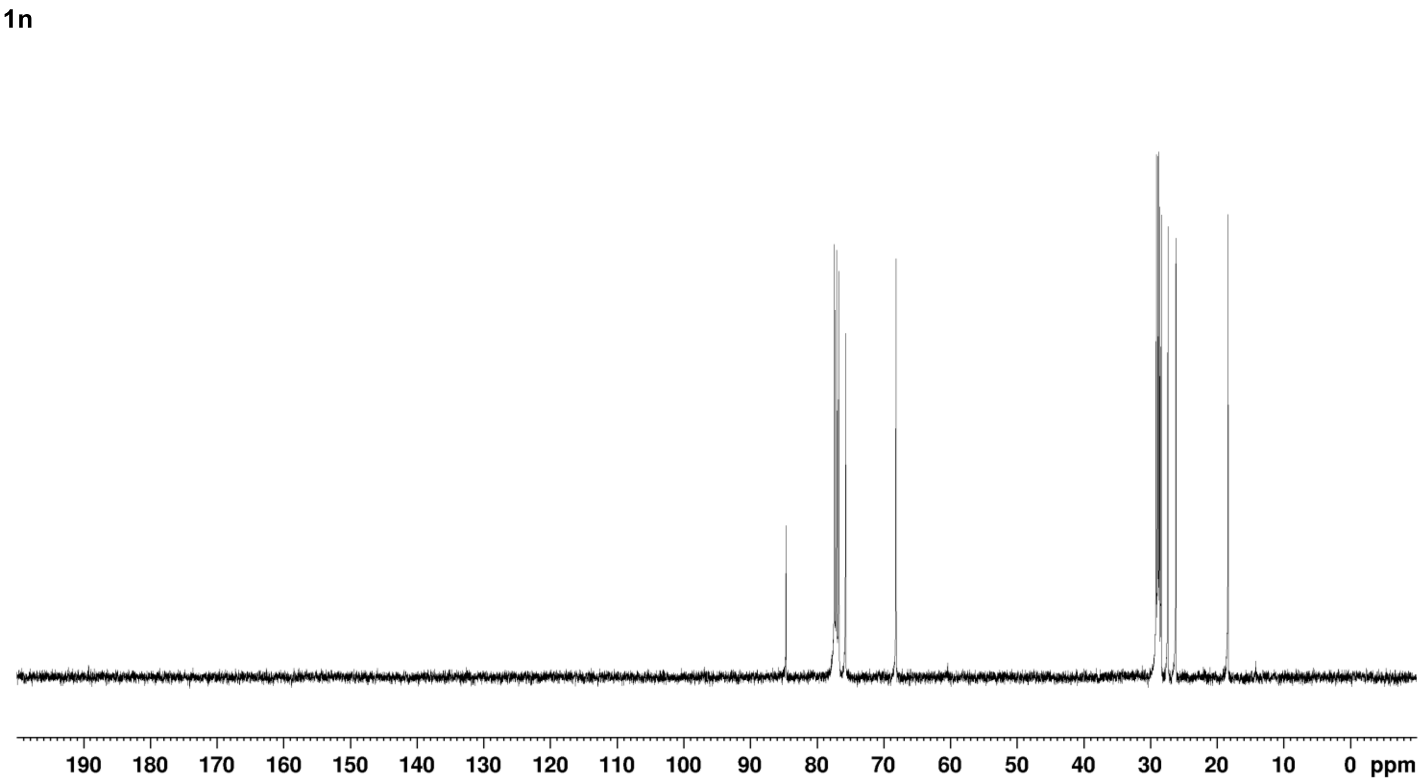

Figure S34. **^{1}H NMR** (400 MHz, $CDCl_3$) of **1o**.

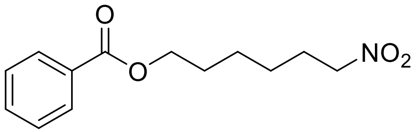

1o

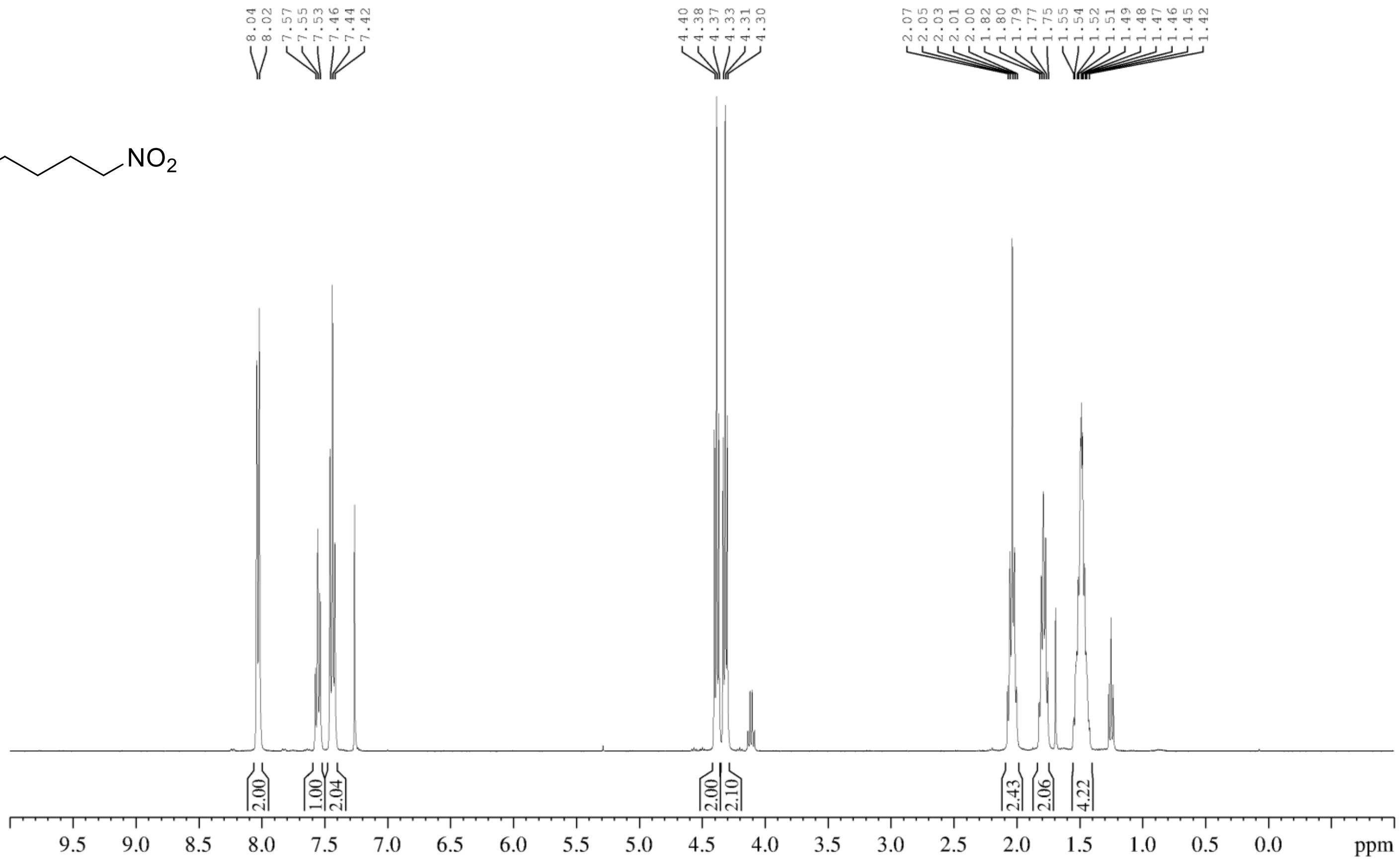

Figure S35. **^{13}C NMR** (101 MHz, $CDCl_3$) of **1o**.

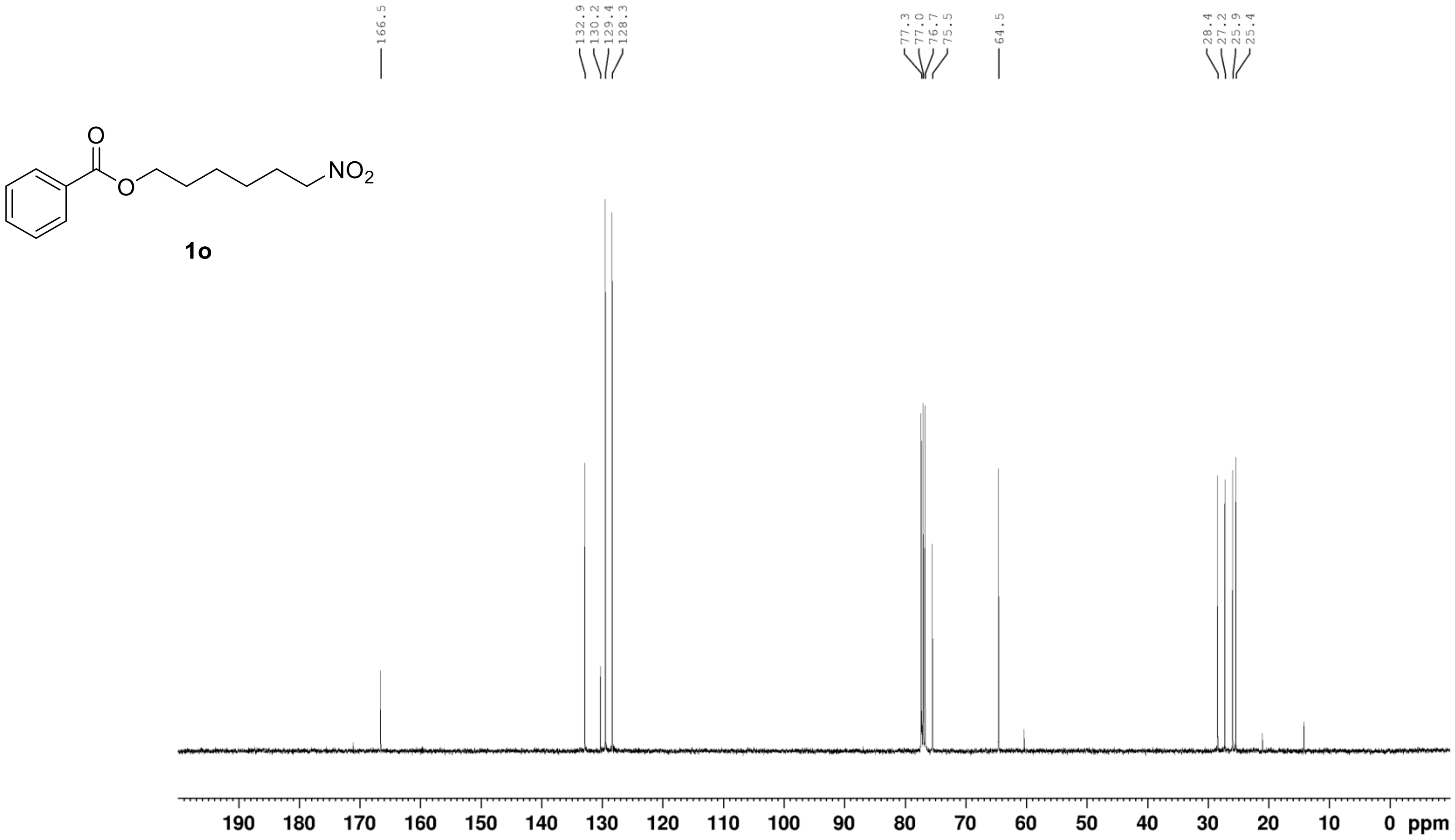

Figure S36. **^{1}H NMR** (400 MHz, $CDCl_3$) of **1p**.

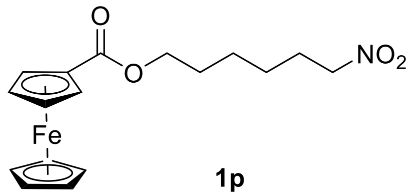

1p

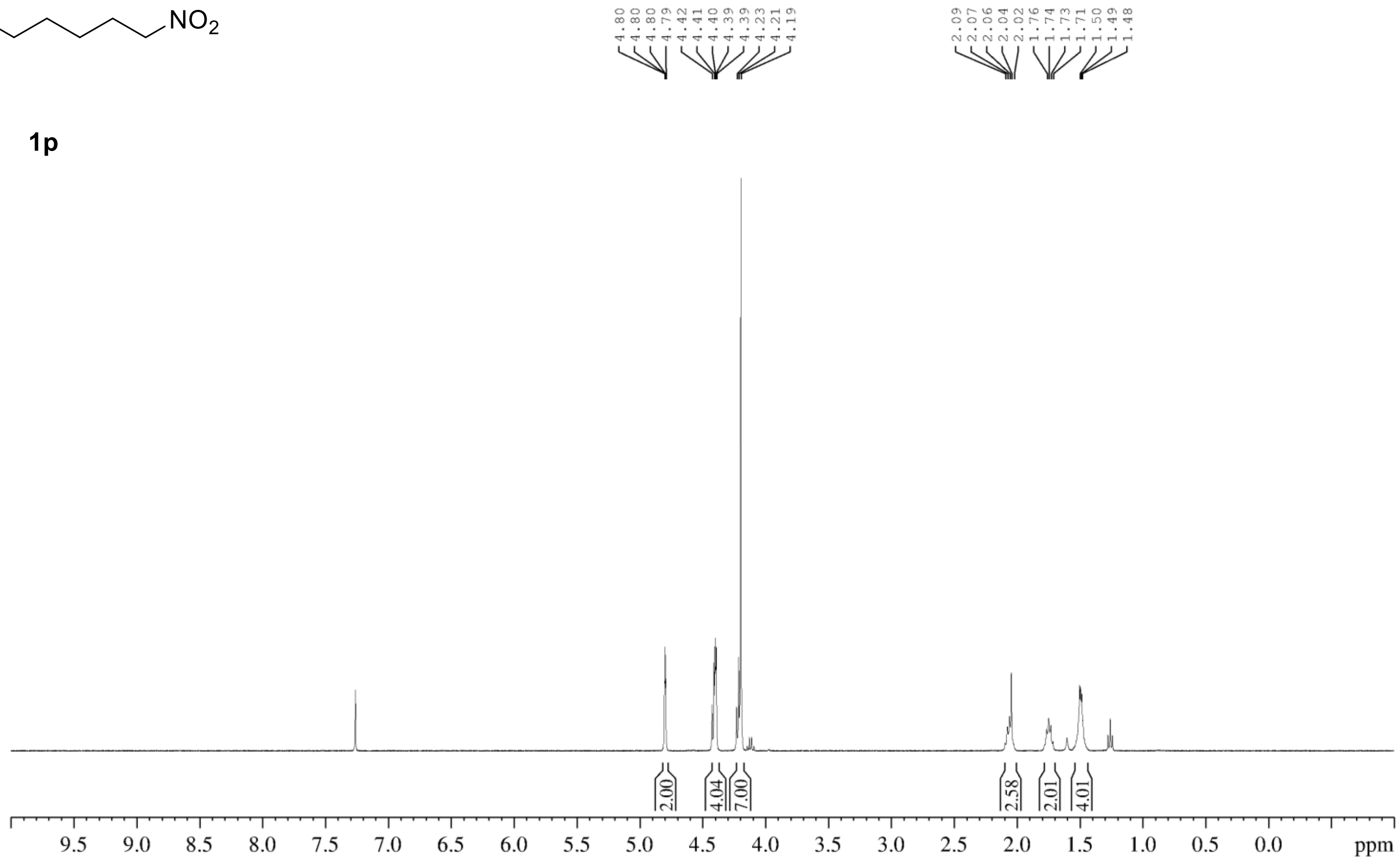

Figure S37. **^{13}C NMR** (101 MHz, $CDCl_3$) of **1p**.

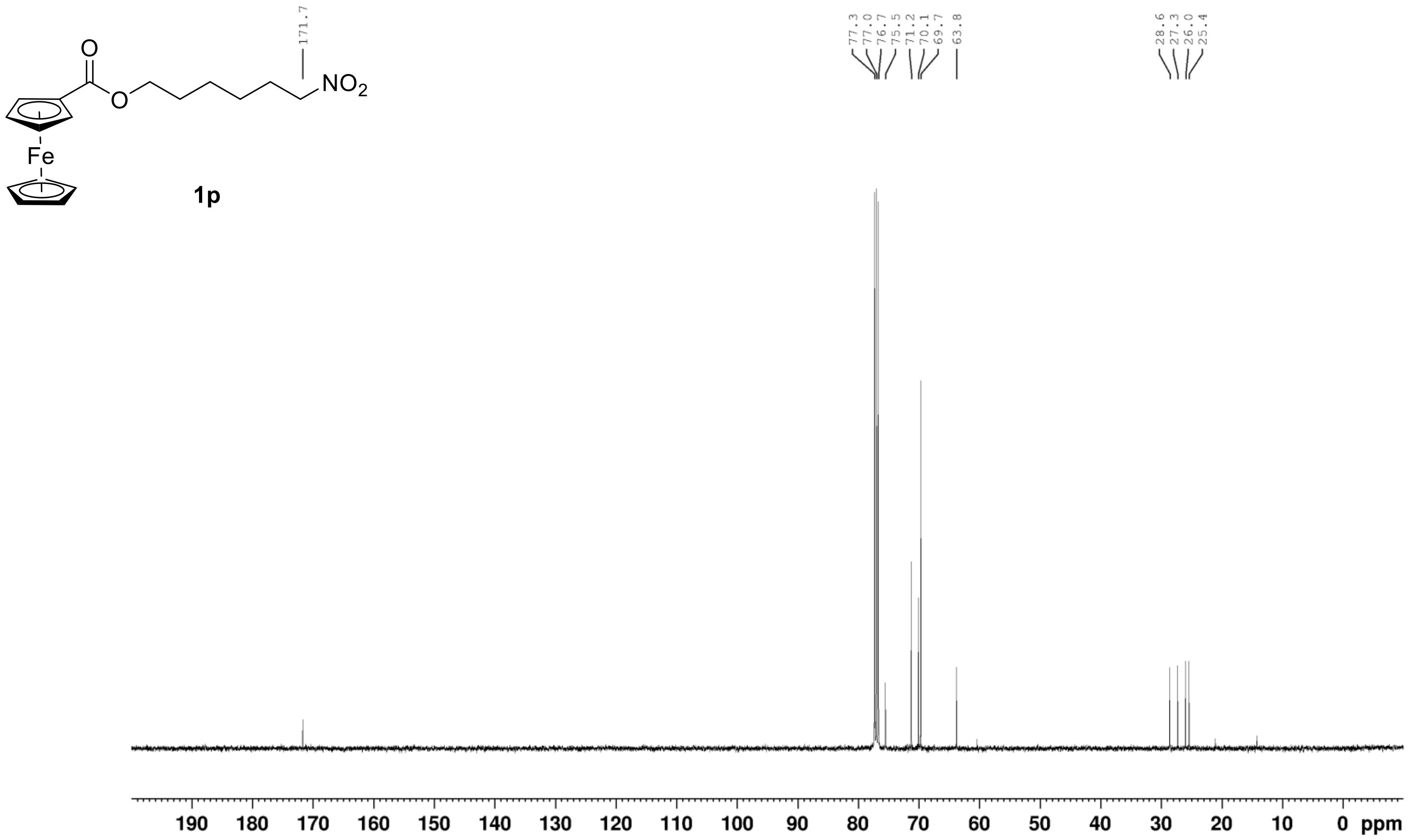

Figure S38. **^{1}H NMR** (400 MHz, $CDCl_3$) of **1q**.

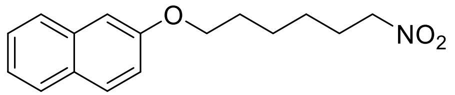

1q

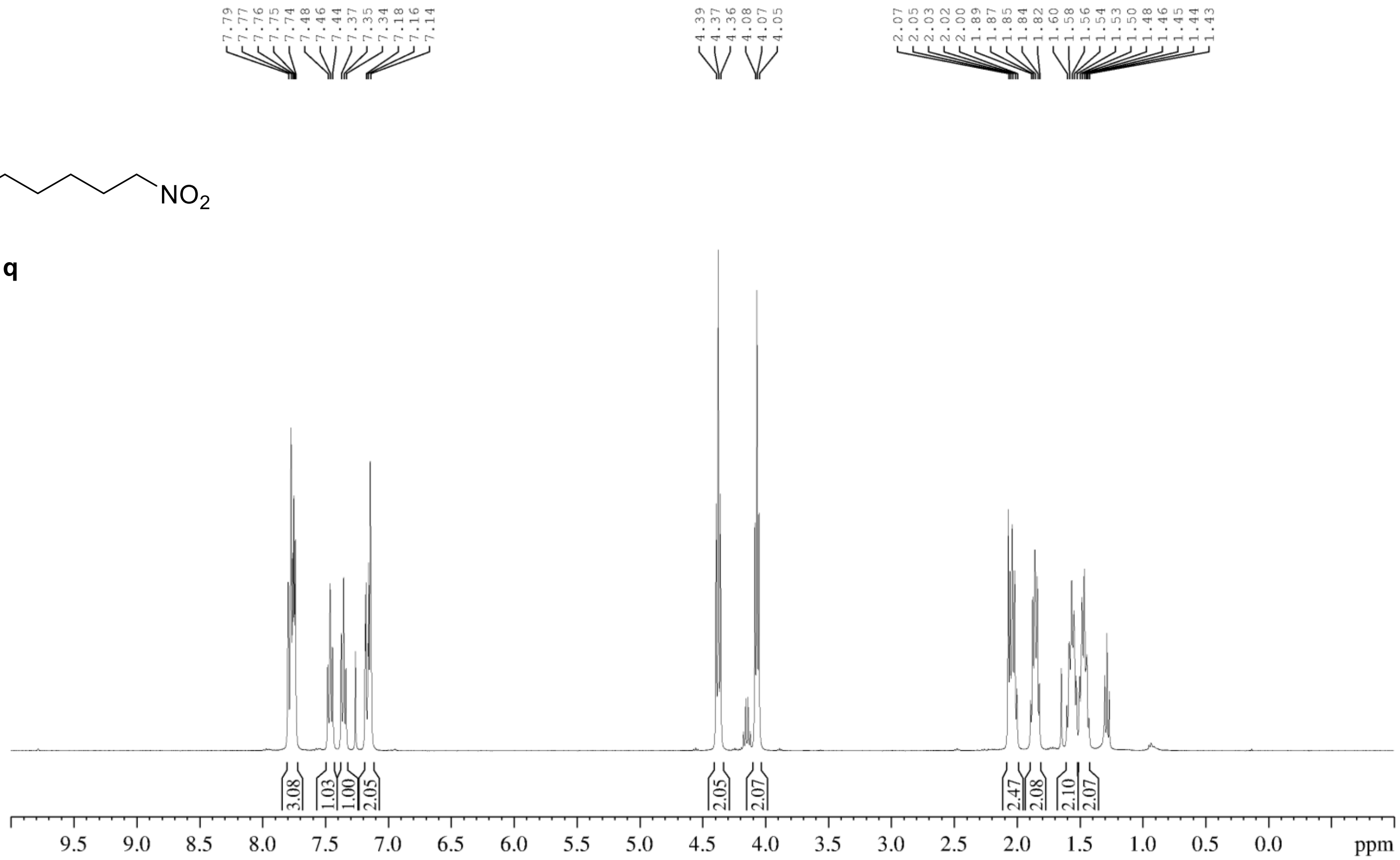

Figure S39. ^{13}**C NMR** (101 MHz, $CDCl_3$) of **1q**.

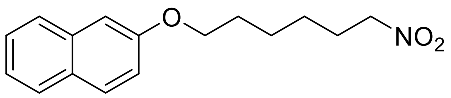

1q

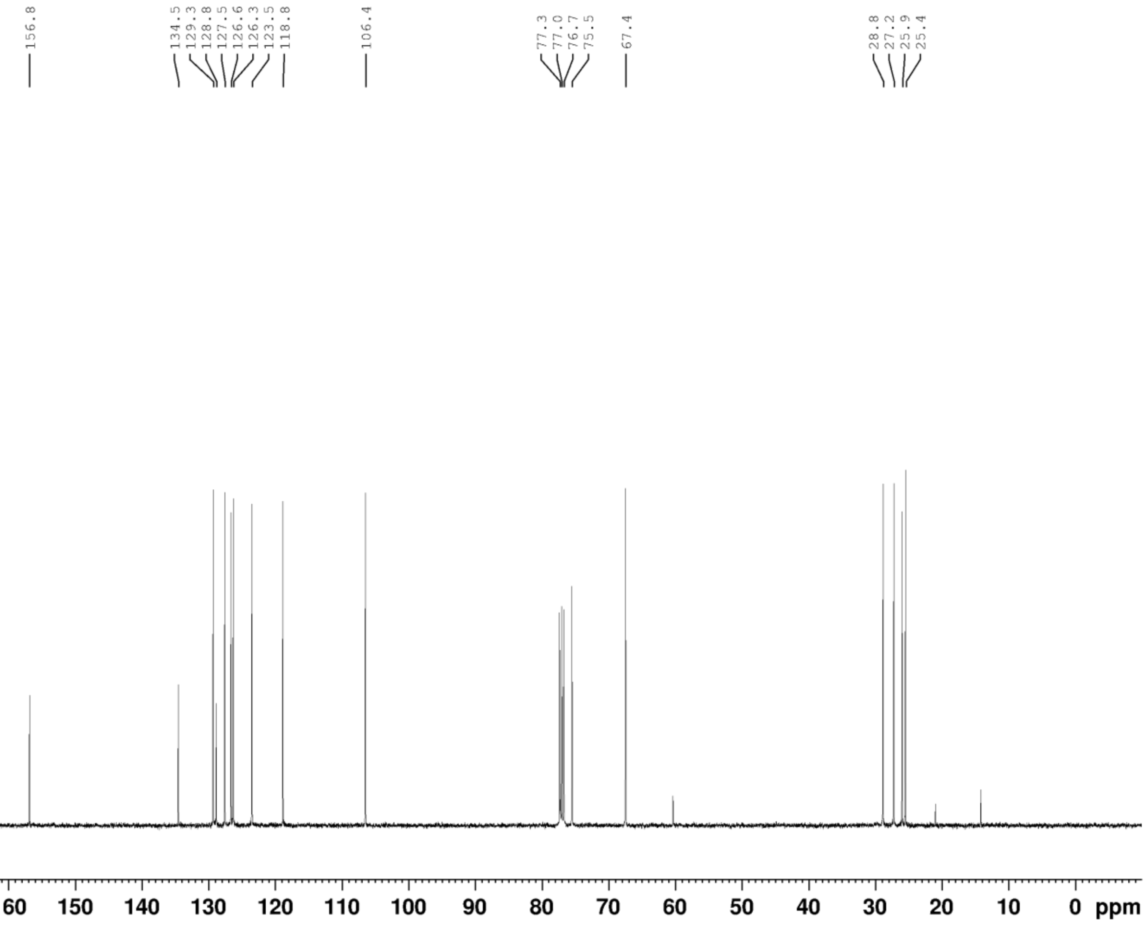

Figure S40. **^{1}H NMR** (400 MHz, $CDCl_3$) of **1r**.

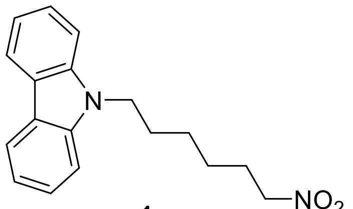

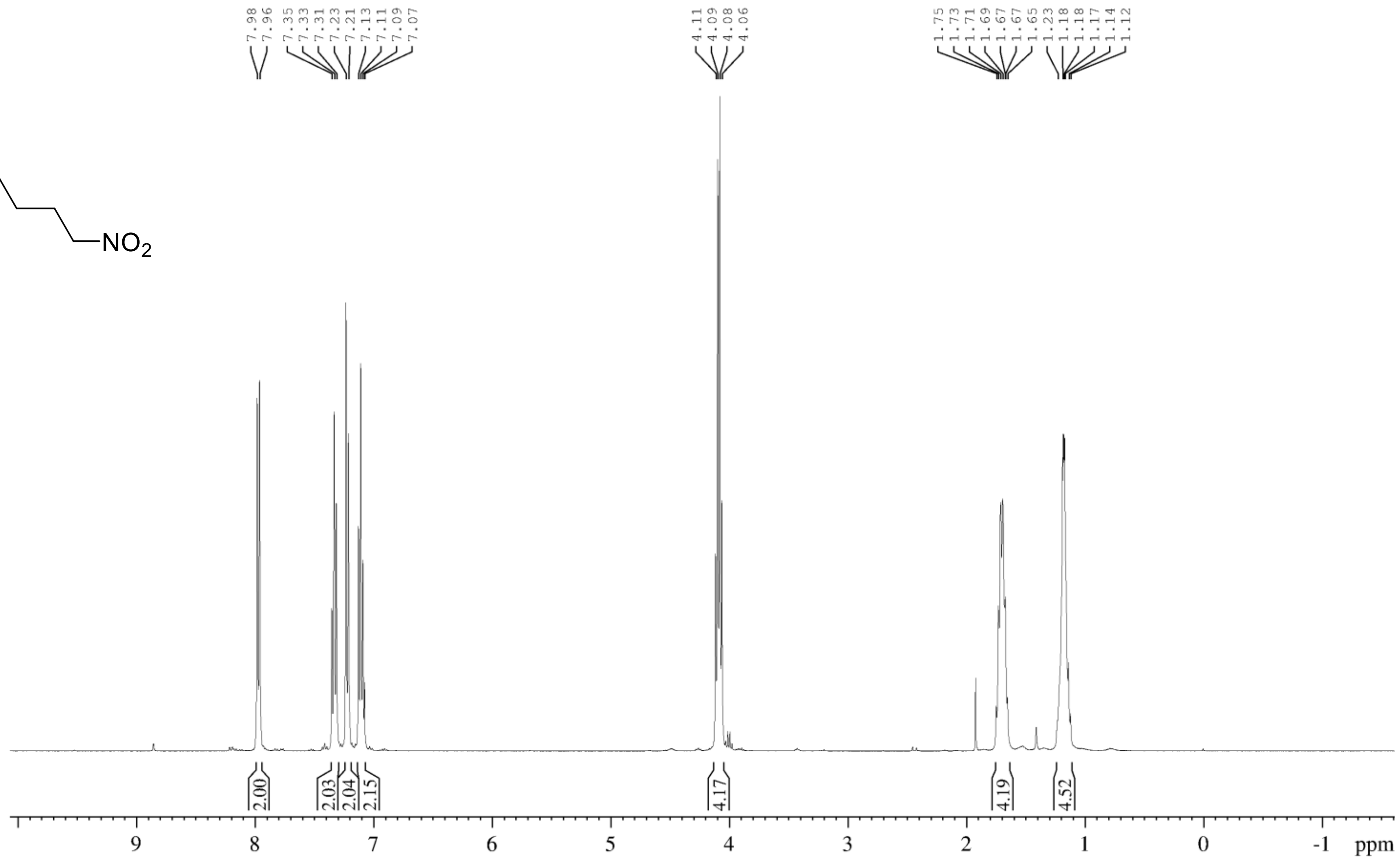

Figure S41. ^{13}**C NMR** (101 MHz, $CDCl_3$) of **1r**.

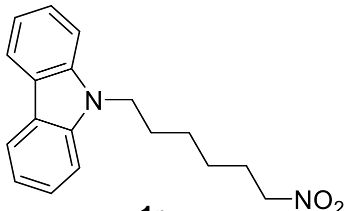

1r

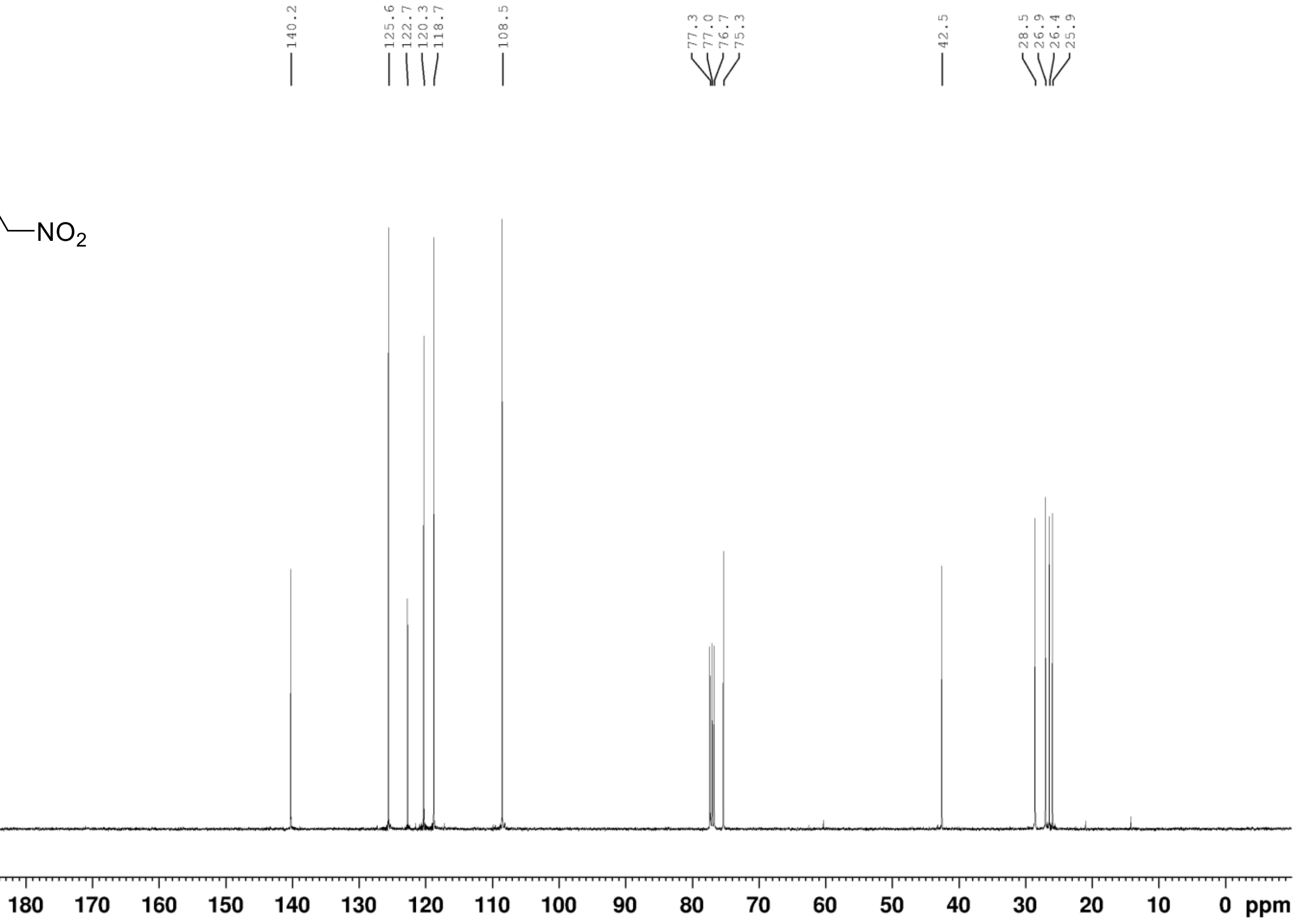

Figure S42. **^{1}H NMR** (400 MHz, $CDCl_3$) of **1s**.

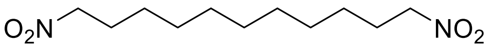

1s

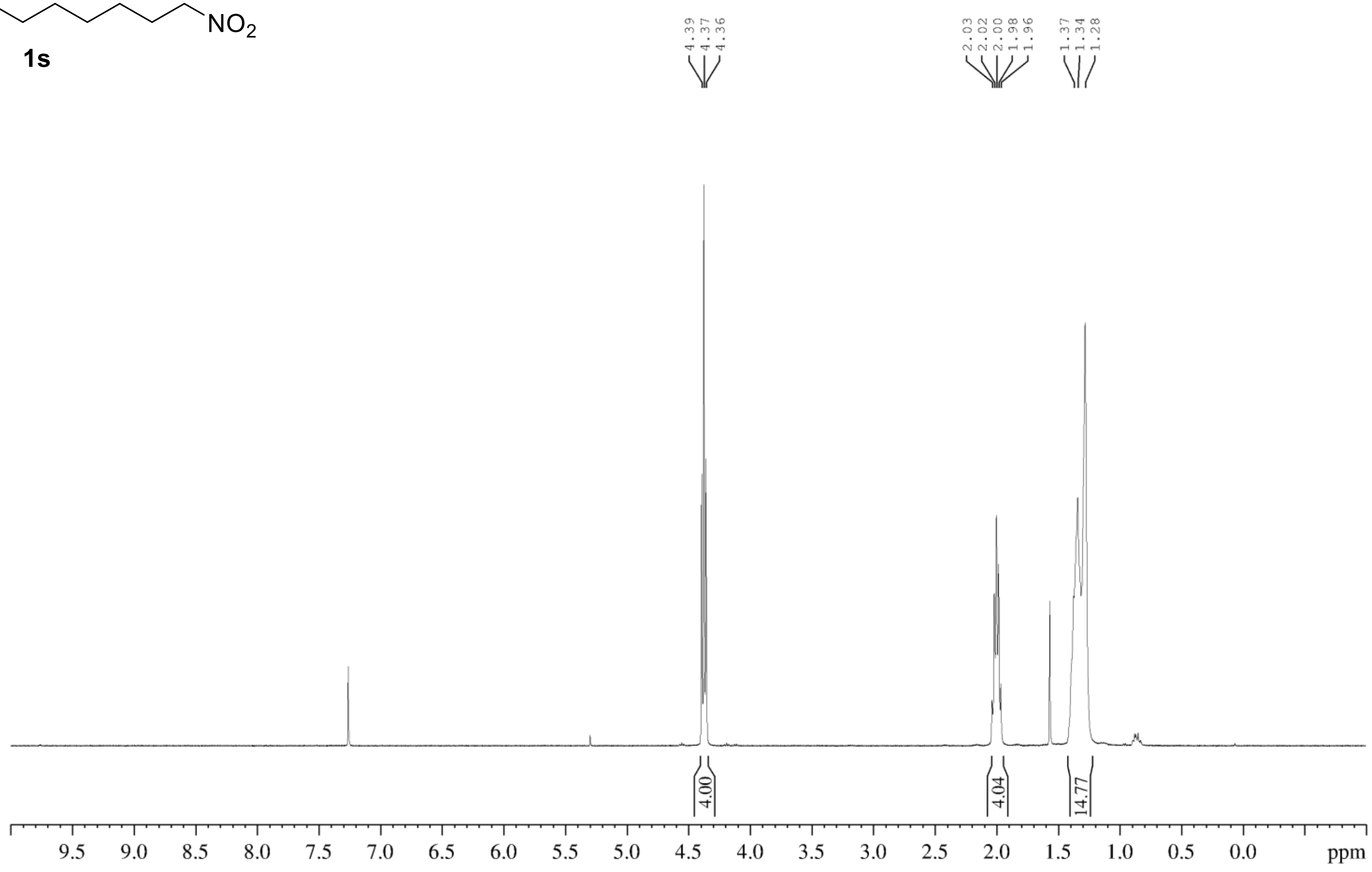

Figure S43. ^{13}**C NMR** (101 MHz, $CDCl_3$) of **1s**.

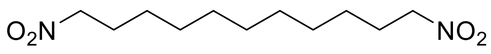

1s

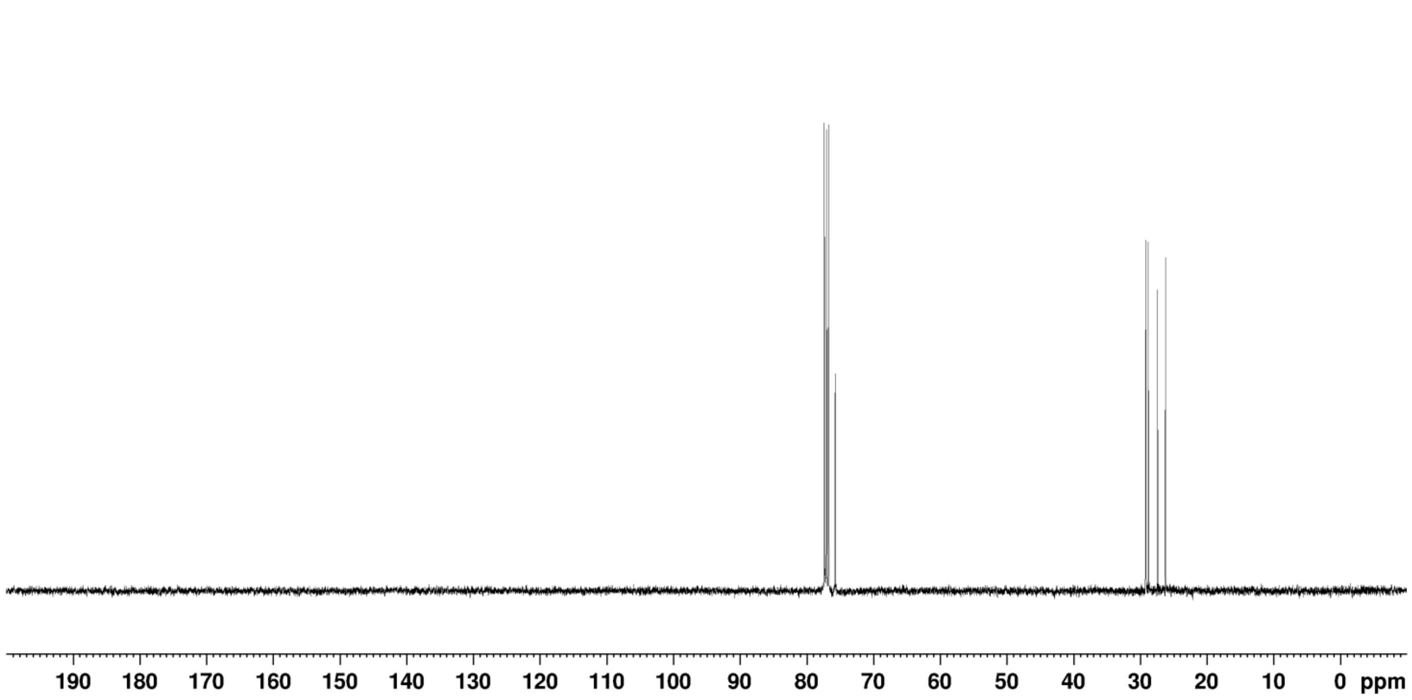

Figure S44. **^{1}H NMR** (400 MHz, $CDCl_3$) of **1t**.

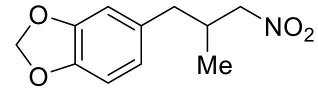

1t

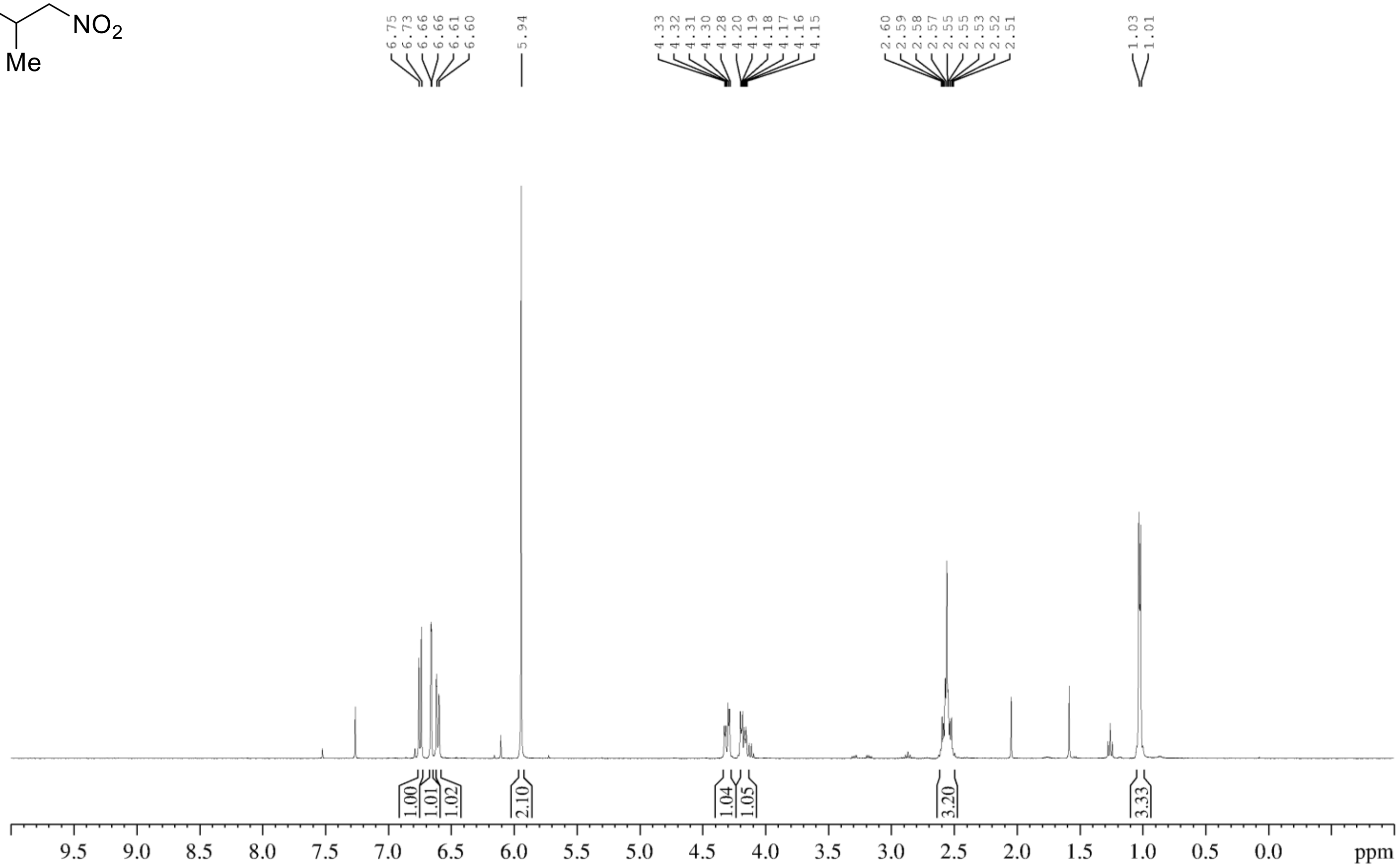

Figure S45. **^{13}C NMR** (101 MHz, $CDCl_3$) of **1t**.

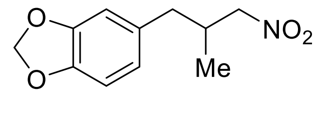

1t

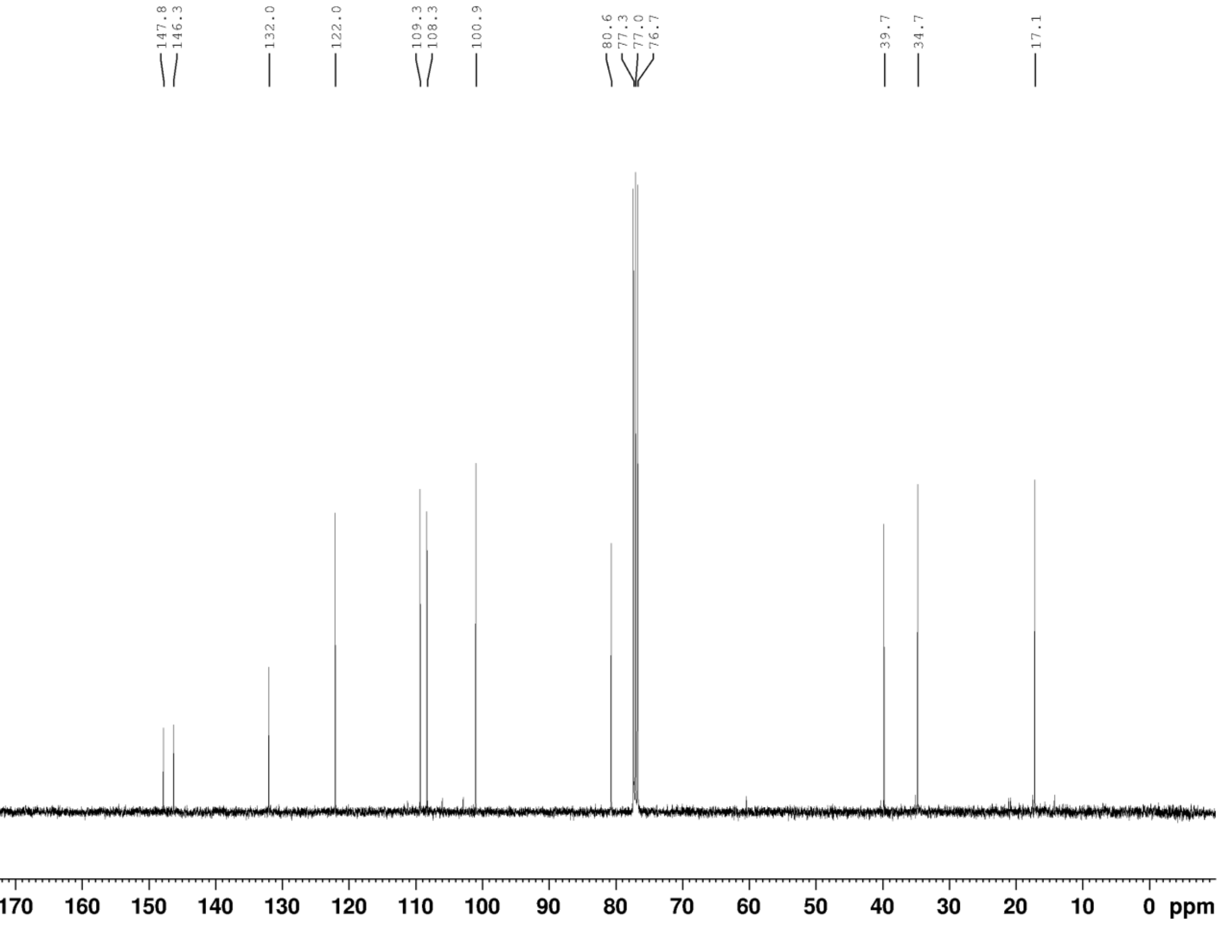

Figure S46. **^{1}H NMR** (500 MHz, $CDCl_3$) of **1u-xiNO$_2$**.

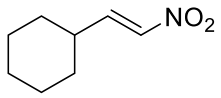

1u-xiNO$_2$

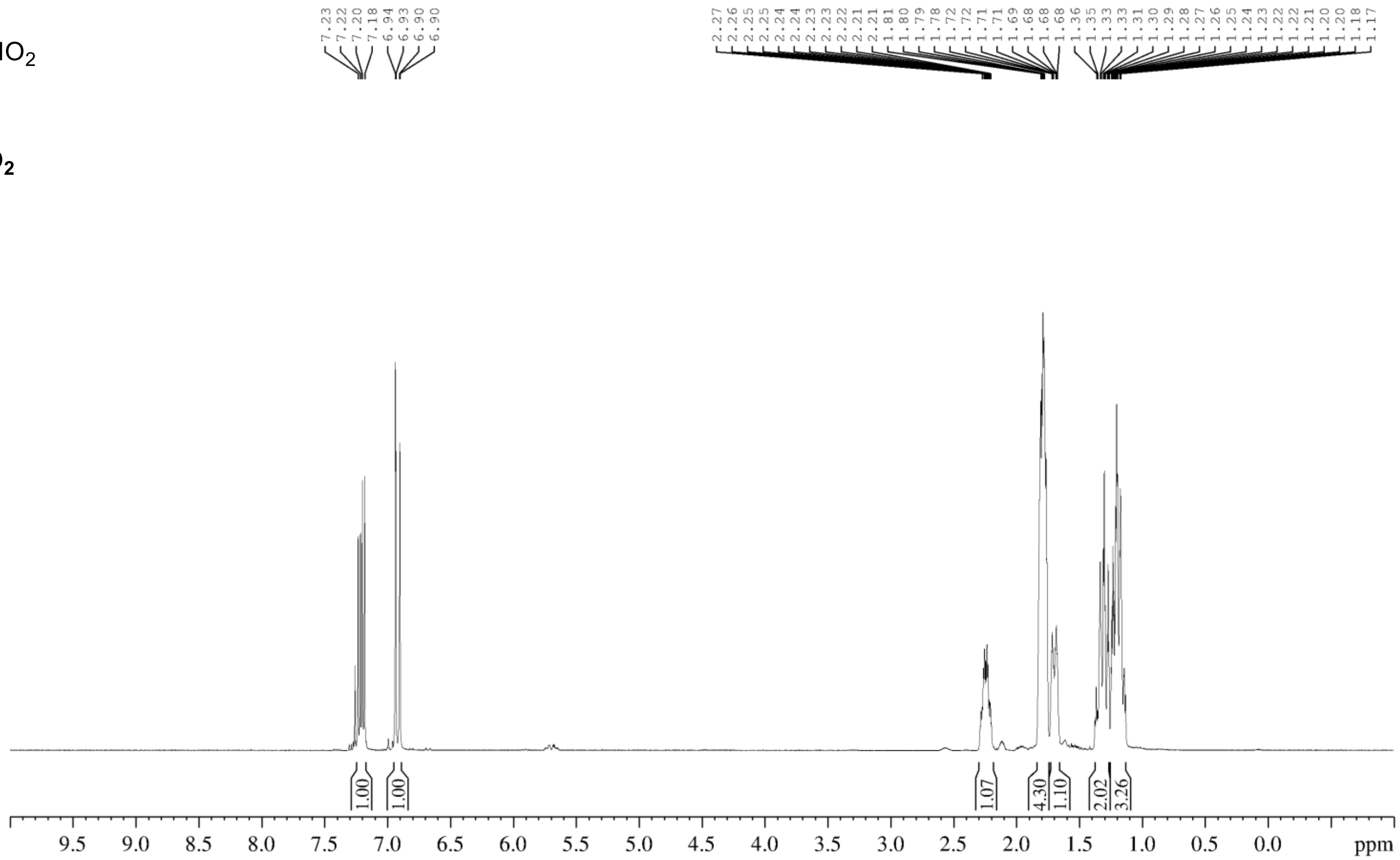

Figure S47. **^{13}C NMR** (126 MHz, $CDCl_3$) of **1u-xiNO$_2$**.

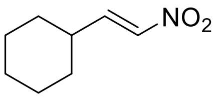

1u-xiNO$_2$

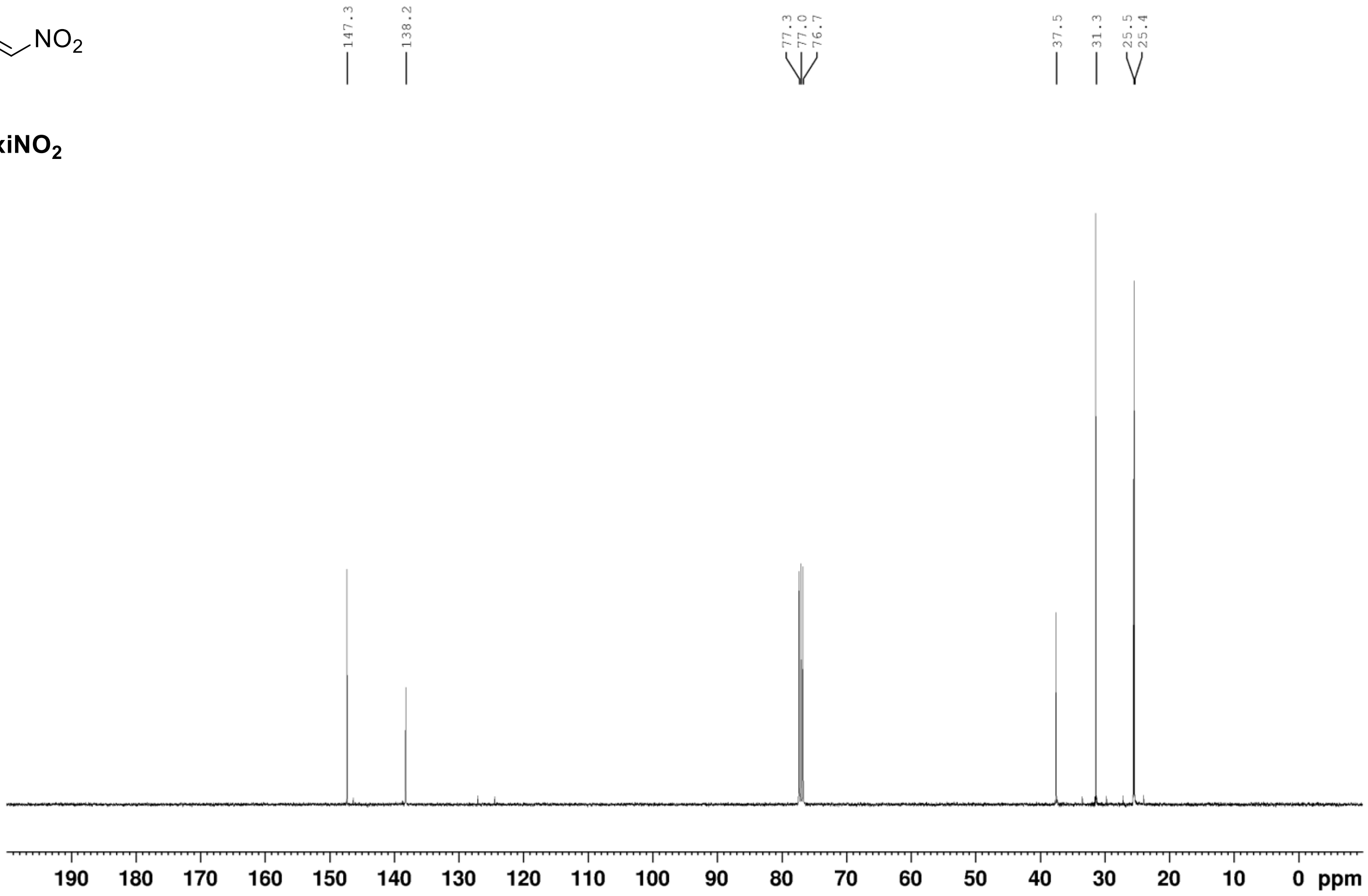

Figure S48. **^{1}H NMR** (500 MHz, $CDCl_3$) of **1u-rac**.

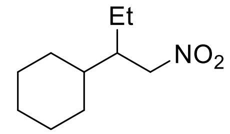

1u-rac

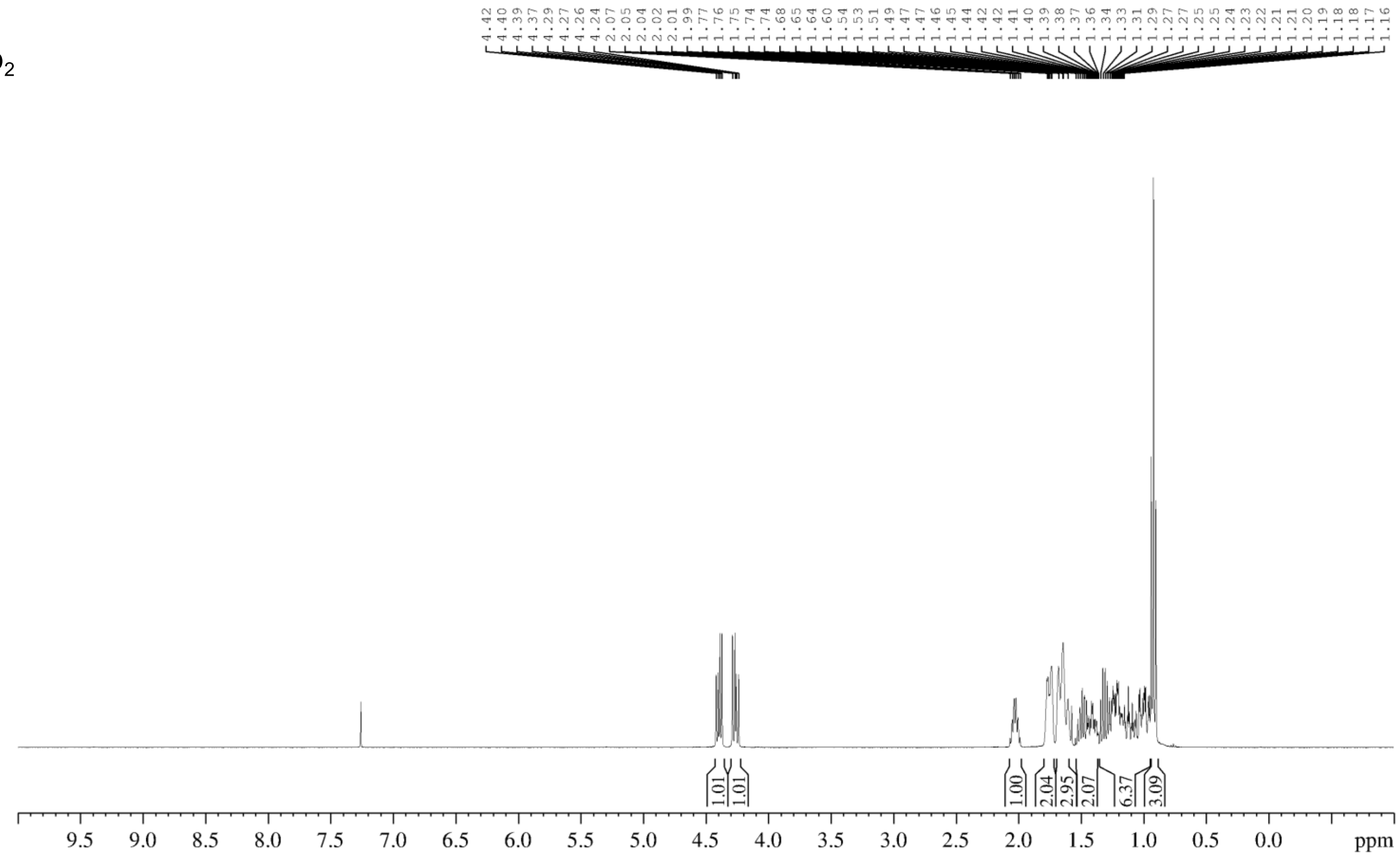

Figure S49. **^{13}C NMR** (126 MHz, $CDCl_3$) of **1u-rac**.

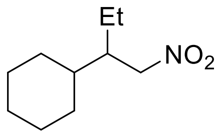

1u-rac

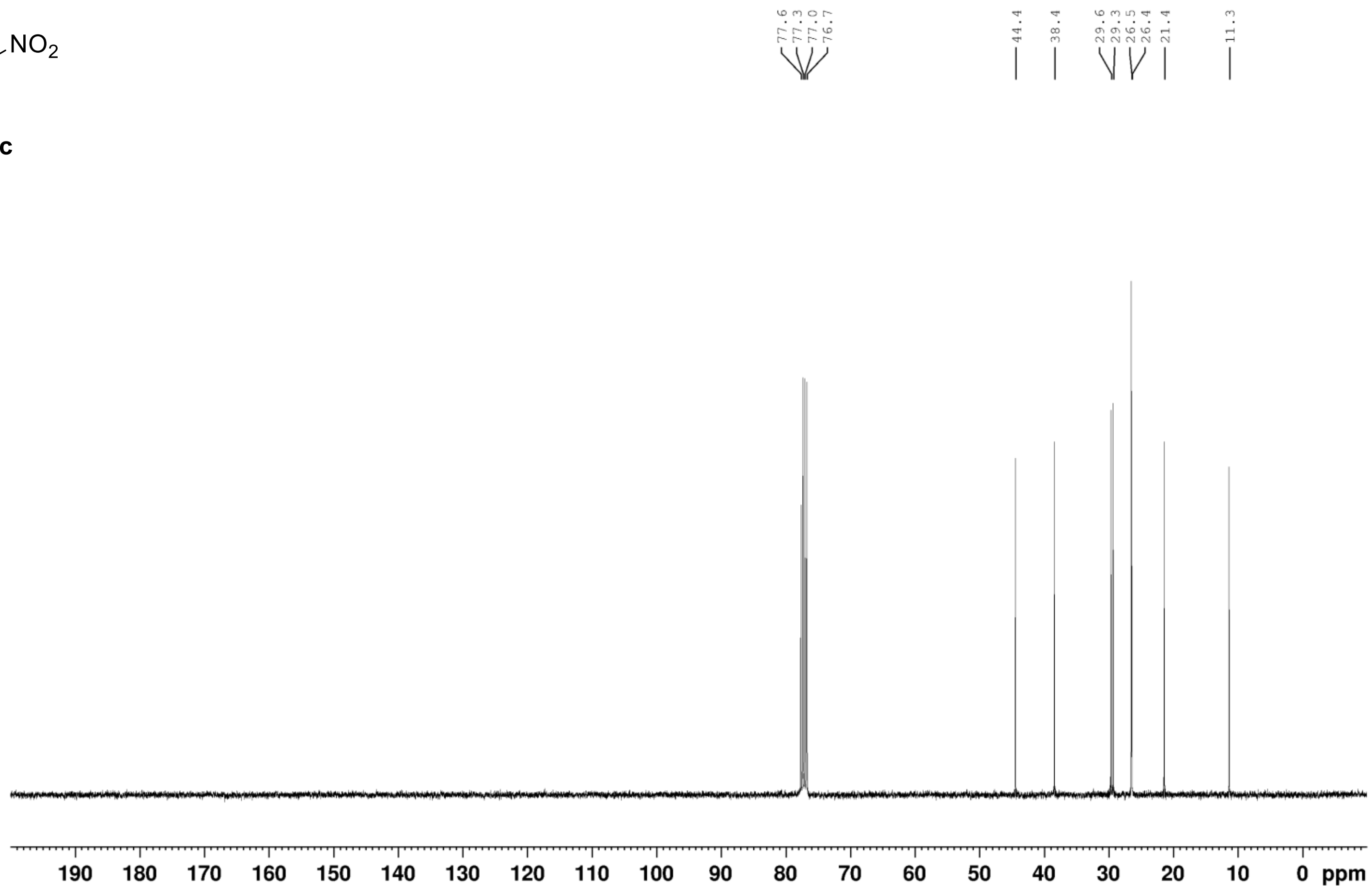

Figure S50. **^{1}H NMR** (500 MHz, $CDCl_3$) of **1u**.

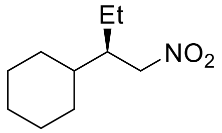

1u

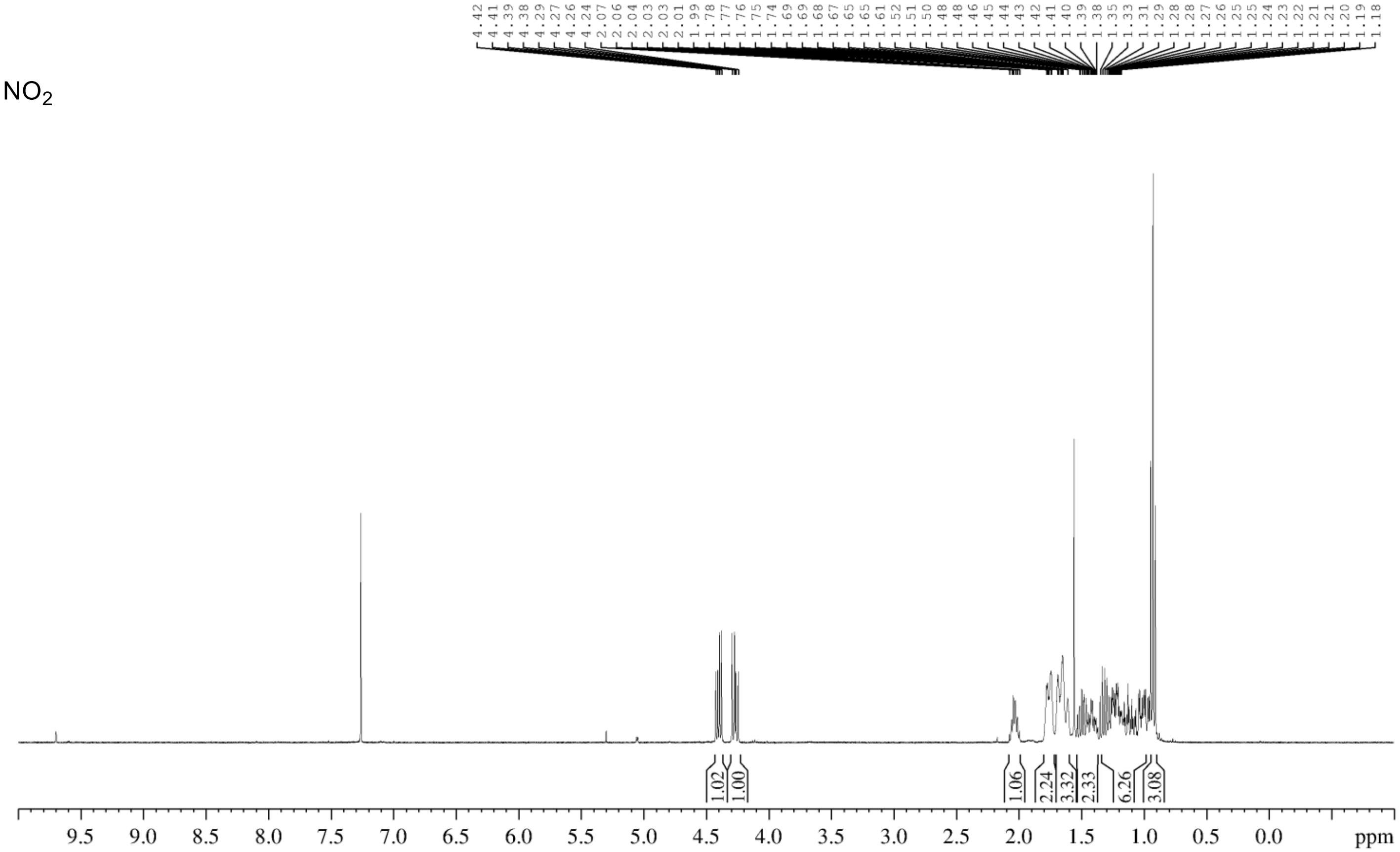

Figure S51. **^{13}C NMR** (126 MHz, $CDCl_3$) of **1u**.

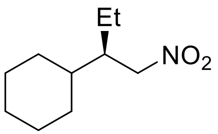

1u

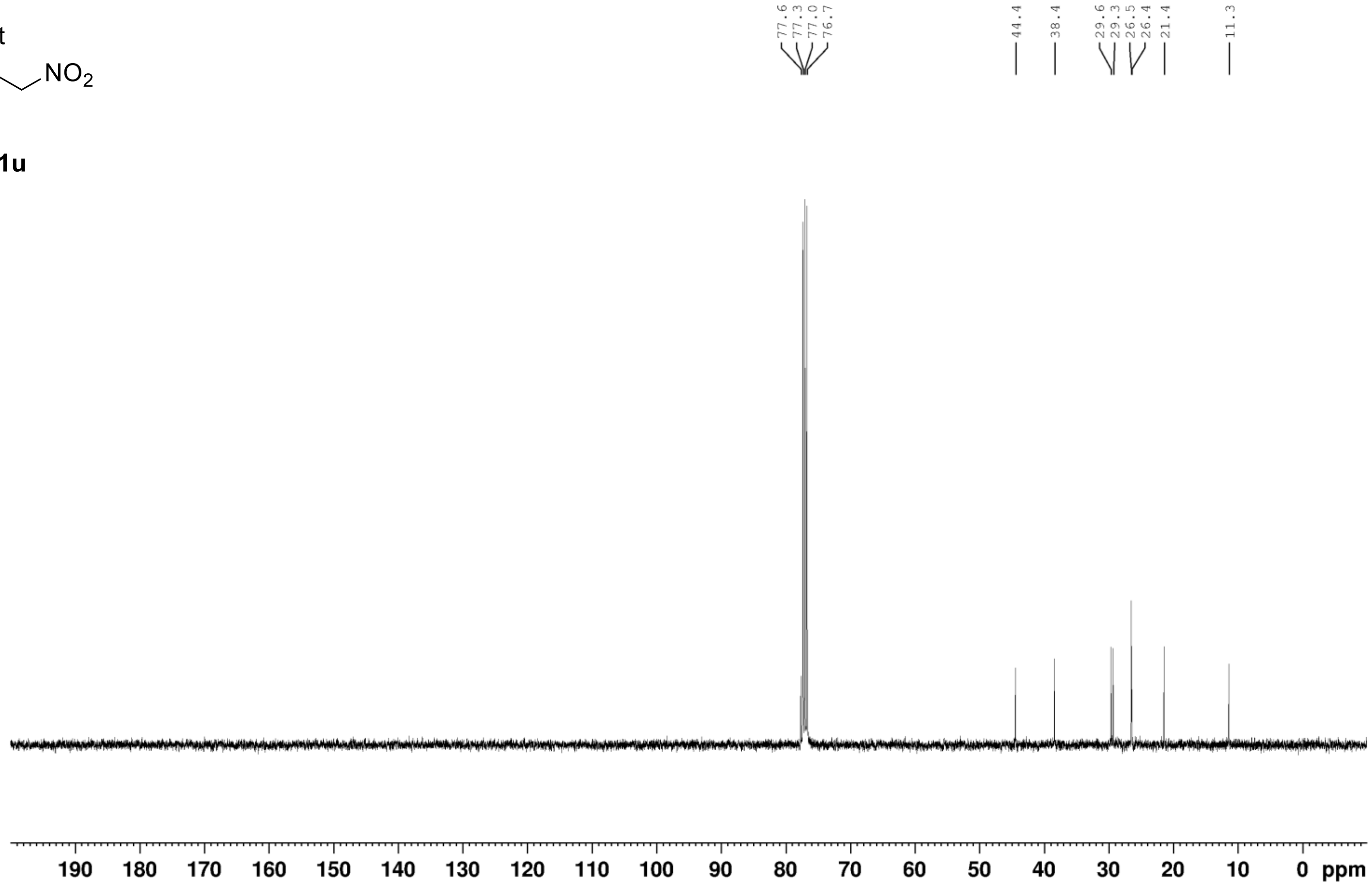

Figure S52. **^{1}H NMR** (400 MHz, $CDCl_3$) of **3a**.

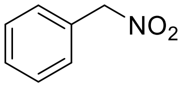

3a

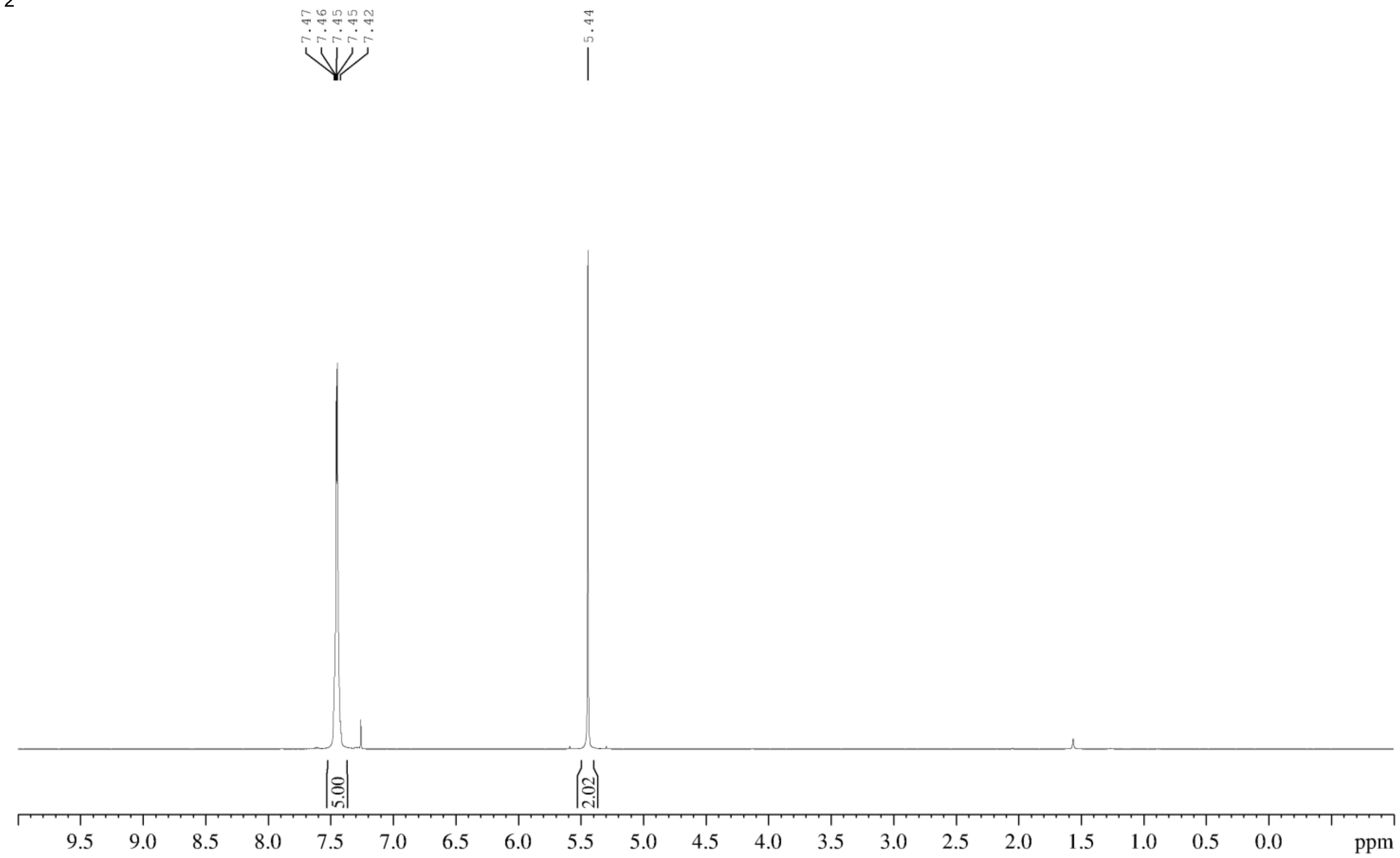

Figure S53. **^{13}C NMR** (101 MHz, $CDCl_3$) of **3a**.

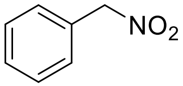

3a

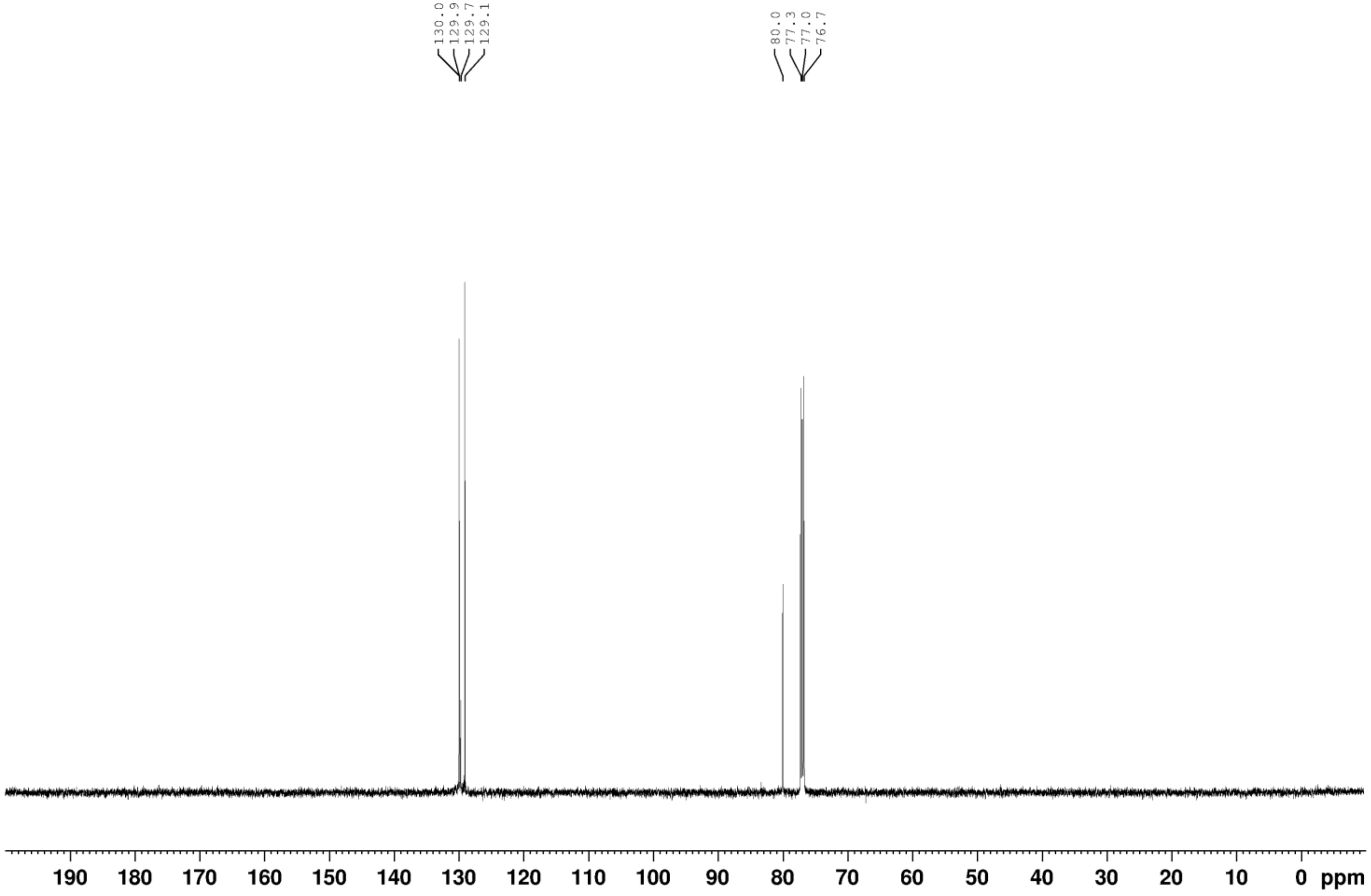

Figure S54. **^{1}H NMR** (400 MHz, $CDCl_3$) of **3b**.

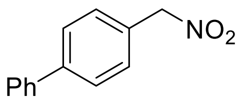

3b

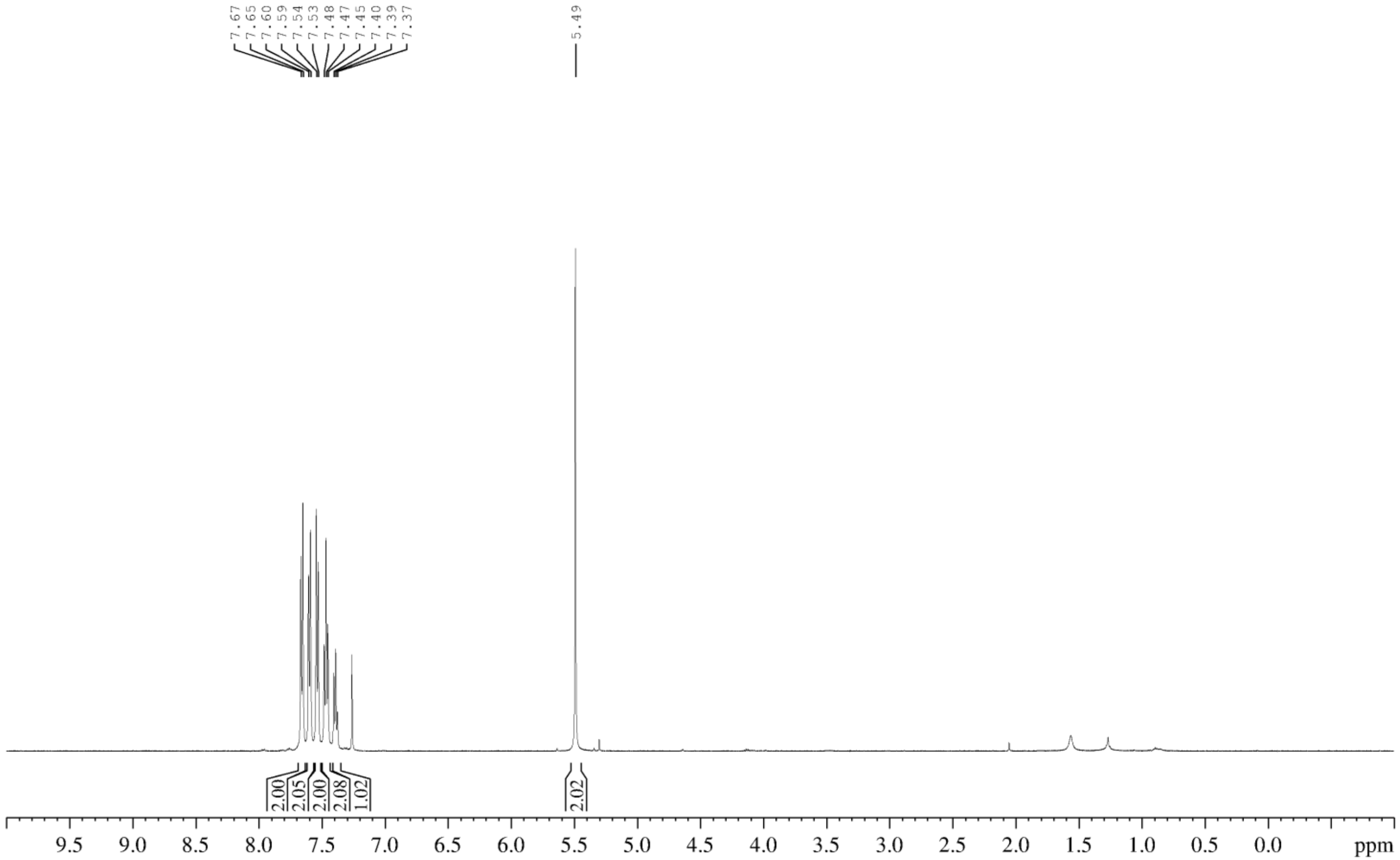

Figure S55. **^{13}C NMR** (101 MHz, $CDCl_3$) of **3b**.

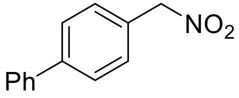

3b

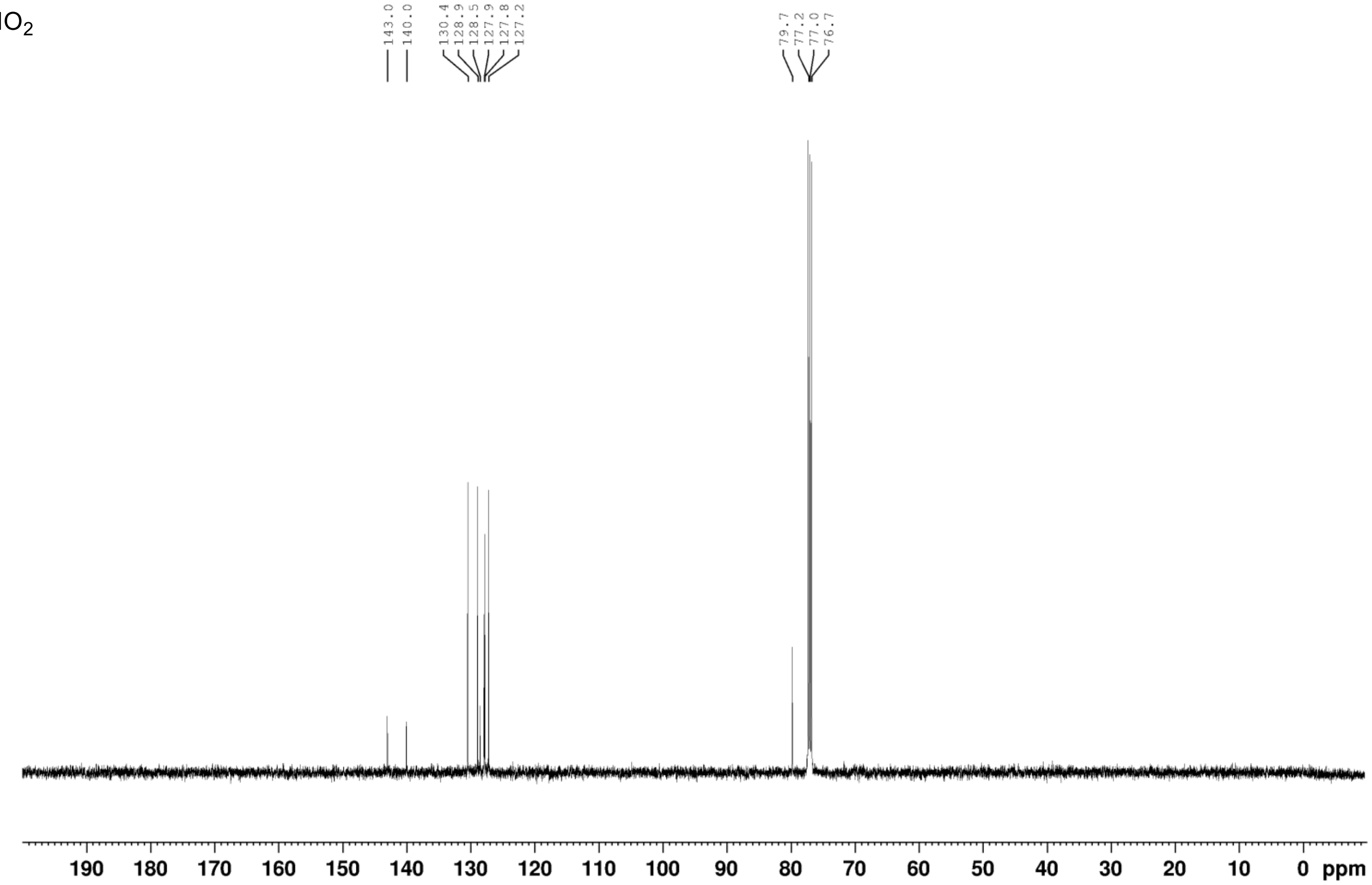

Figure S56. **^{1}H NMR** (400 MHz, $CDCl_3$) of **3c**.

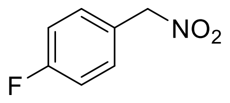

3c

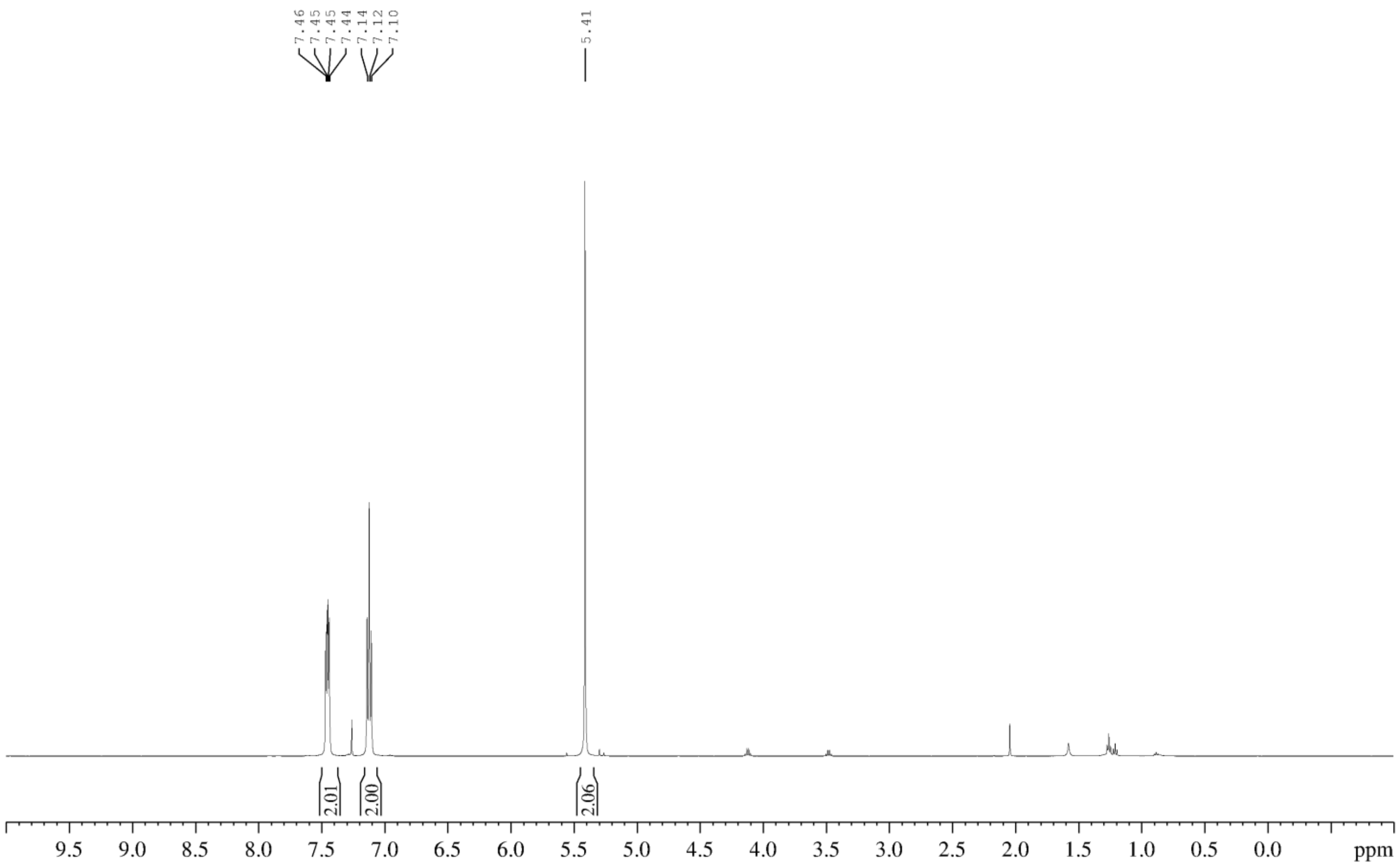

Figure S57. **^{13}C NMR** (101 MHz, $CDCl_3$) of **3c**.

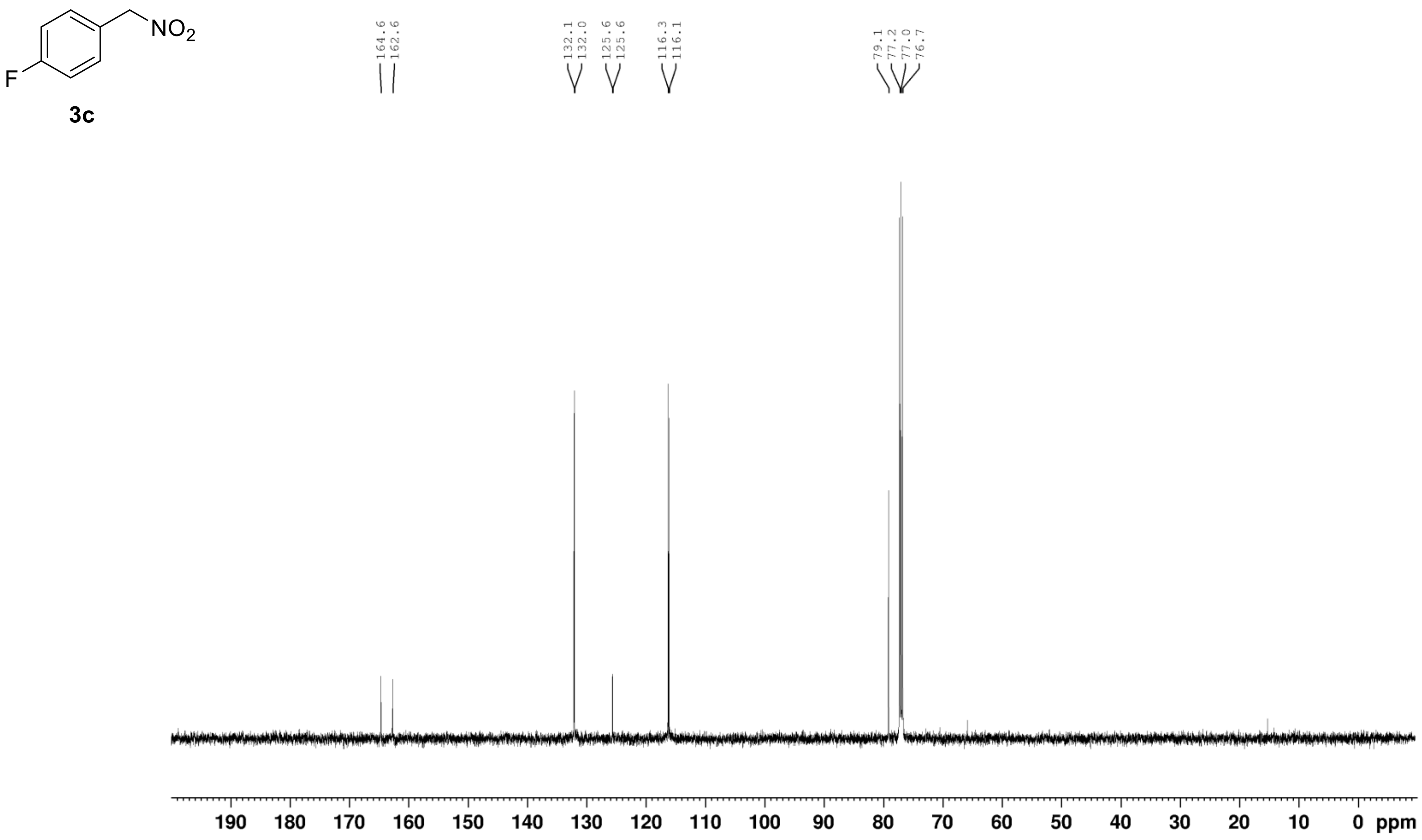

Figure S58. **^{19}F NMR** (471 MHz, $CDCl_3$, 298 K) of **3c**.

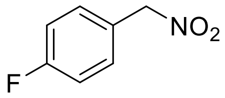

3c

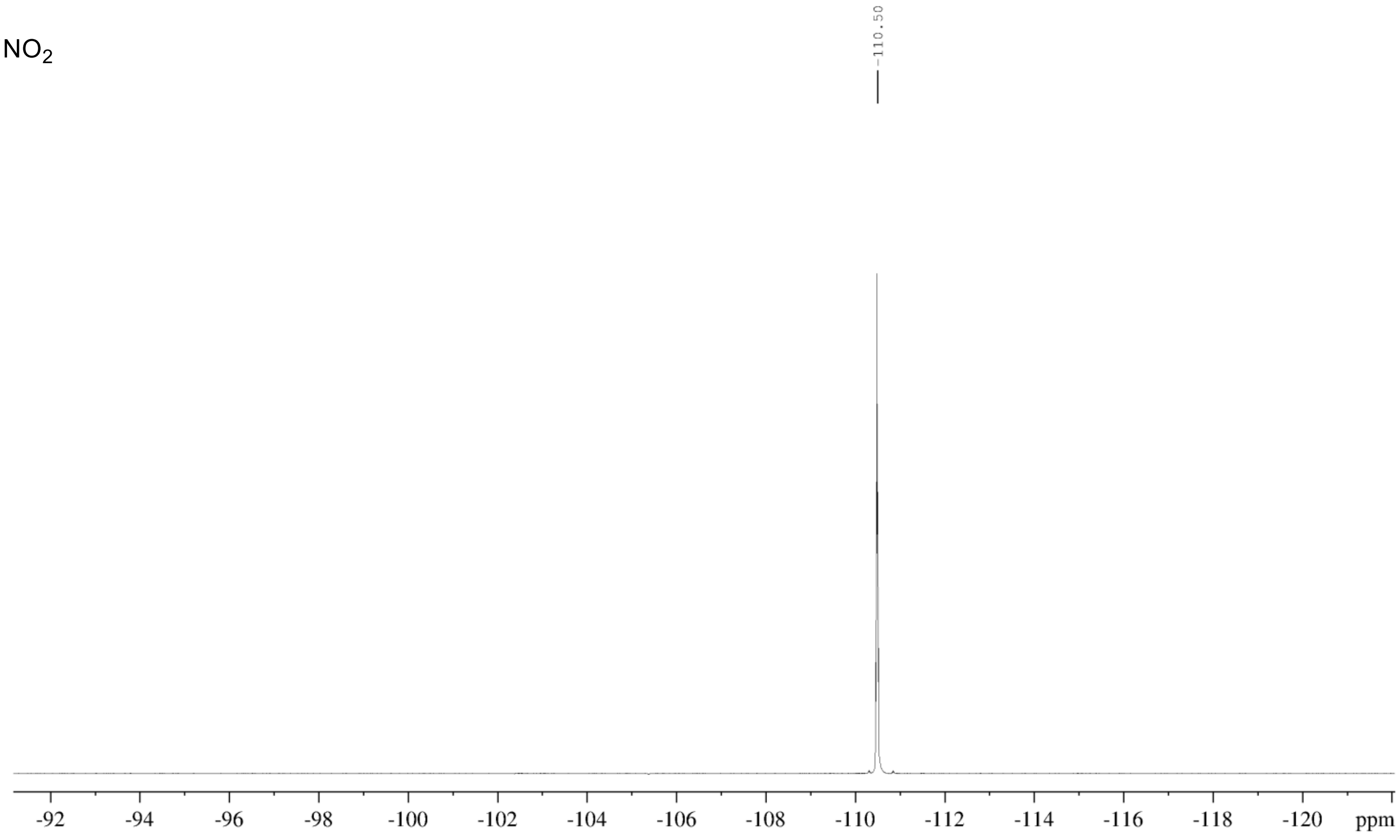

Figure S59. ^{1}**H NMR** (400 MHz, $CDCl_3$) of **3d**.

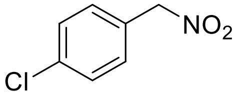

3d

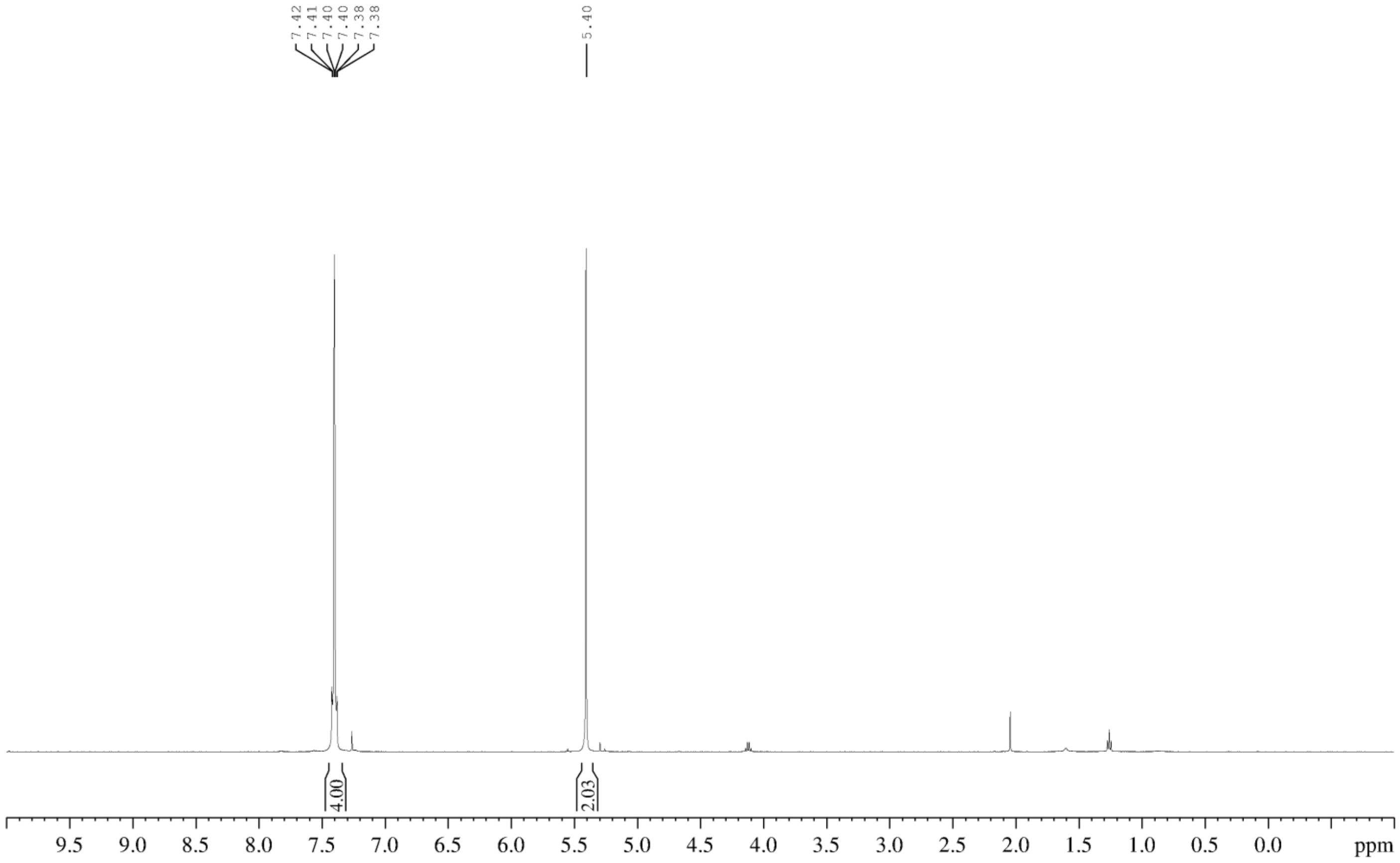

Figure S60. ^{13}**C NMR** (101 MHz, $CDCl_3$) of **3d**.

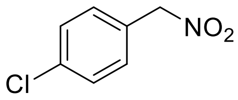

3d

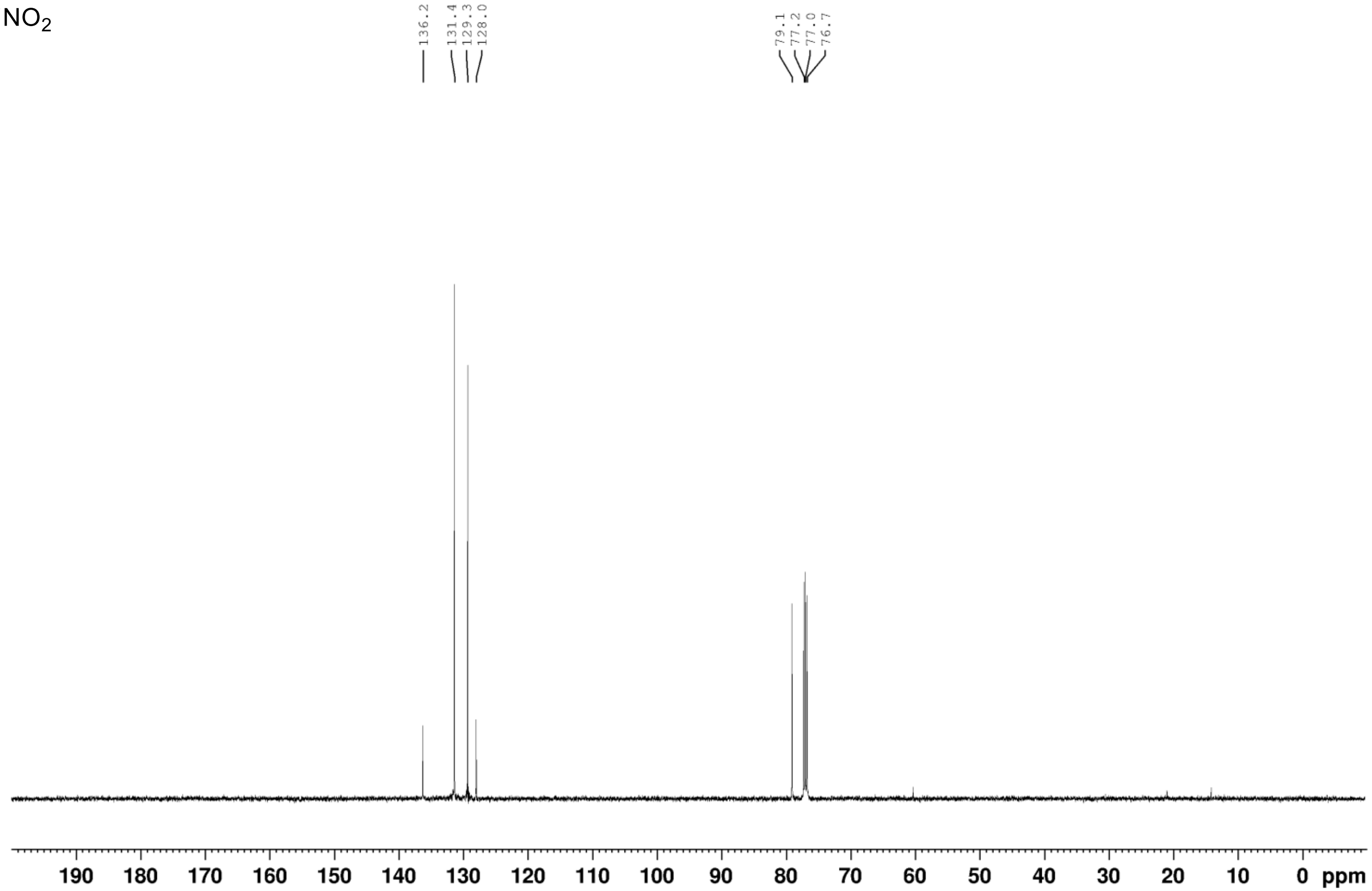

Figure S61. **^{1}H NMR** (400 MHz, $CDCl_3$) of **3e**.

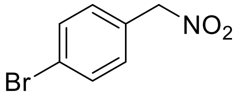

3e

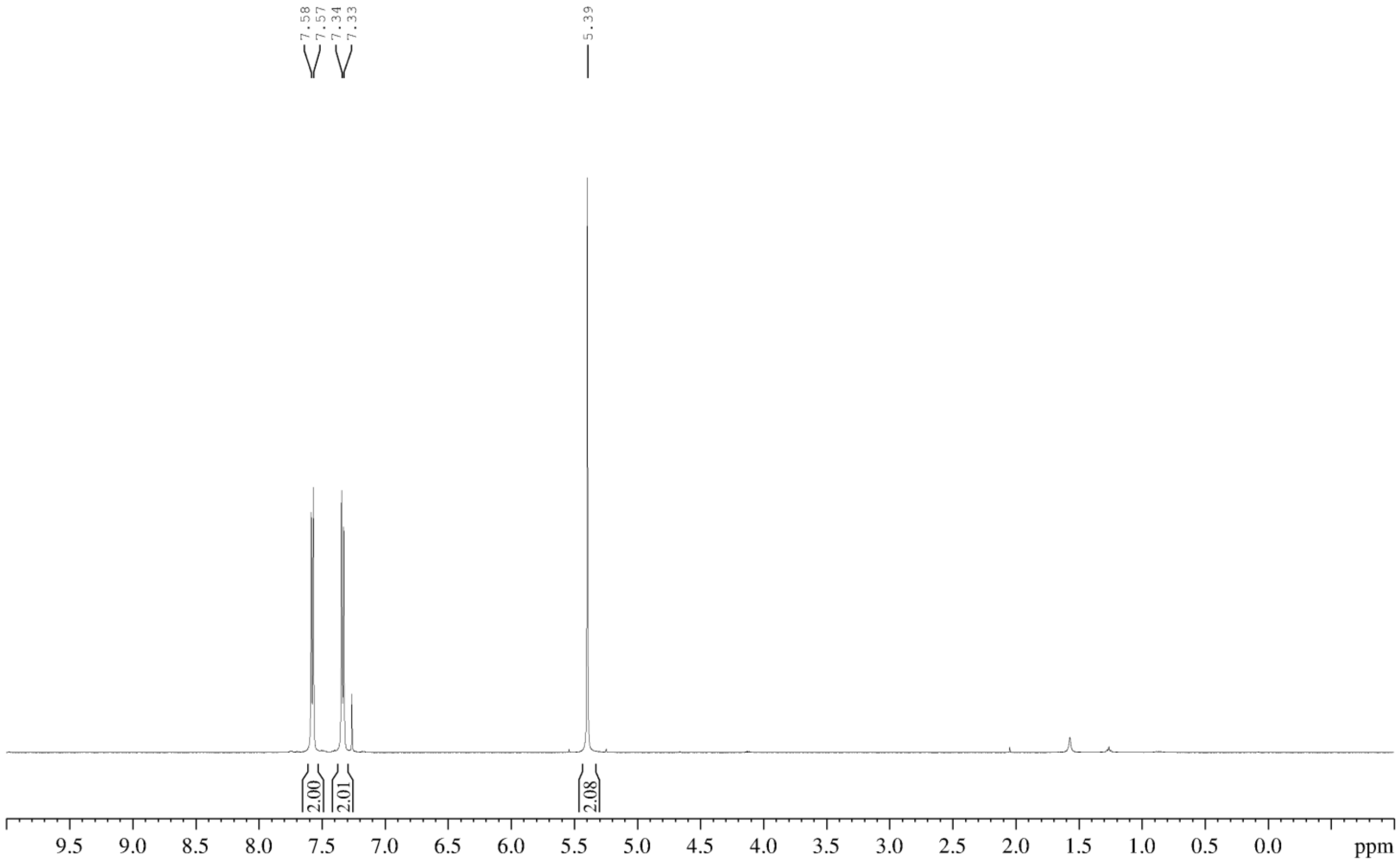

Figure S62. **^{13}C NMR** (101 MHz, $CDCl_3$) of **3e**.

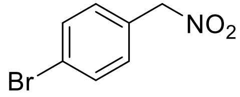

3e

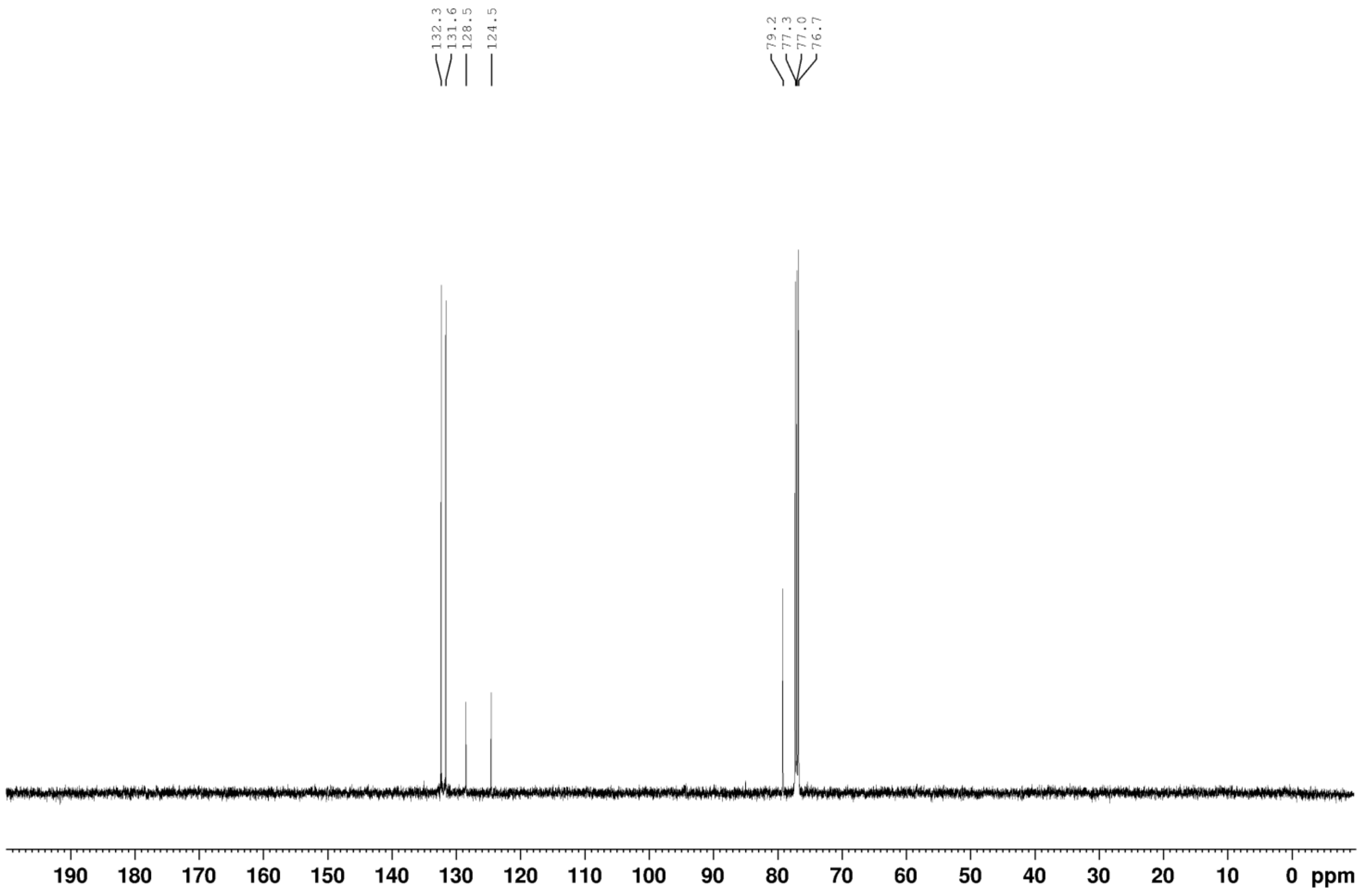

Figure S63. **^{1}H NMR** (400 MHz, $CDCl_3$) of **3f**.

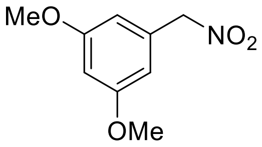

3f

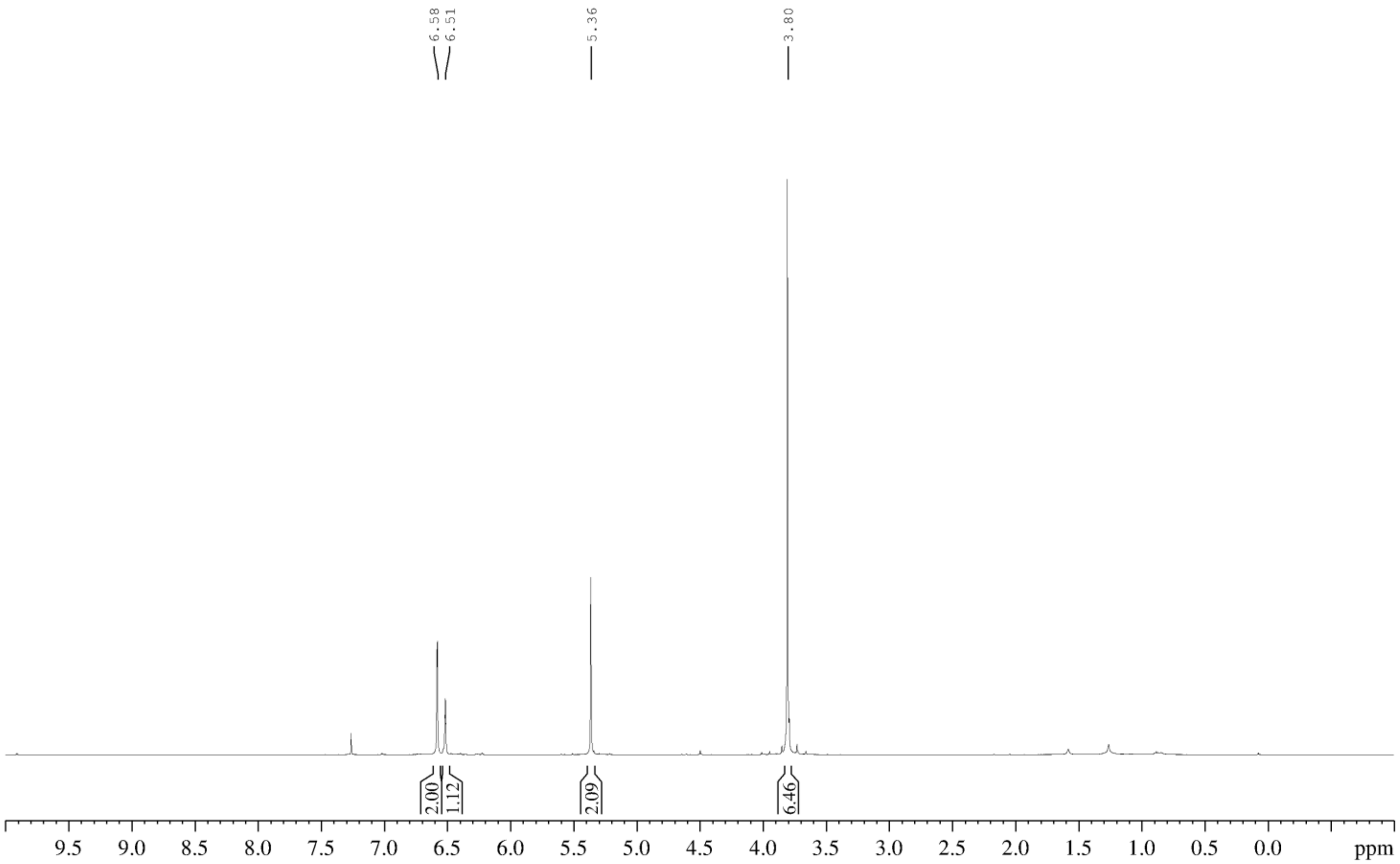

Figure S64. **^{13}C NMR** (101 MHz, $CDCl_3$) of **3f**.

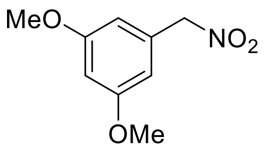

3f

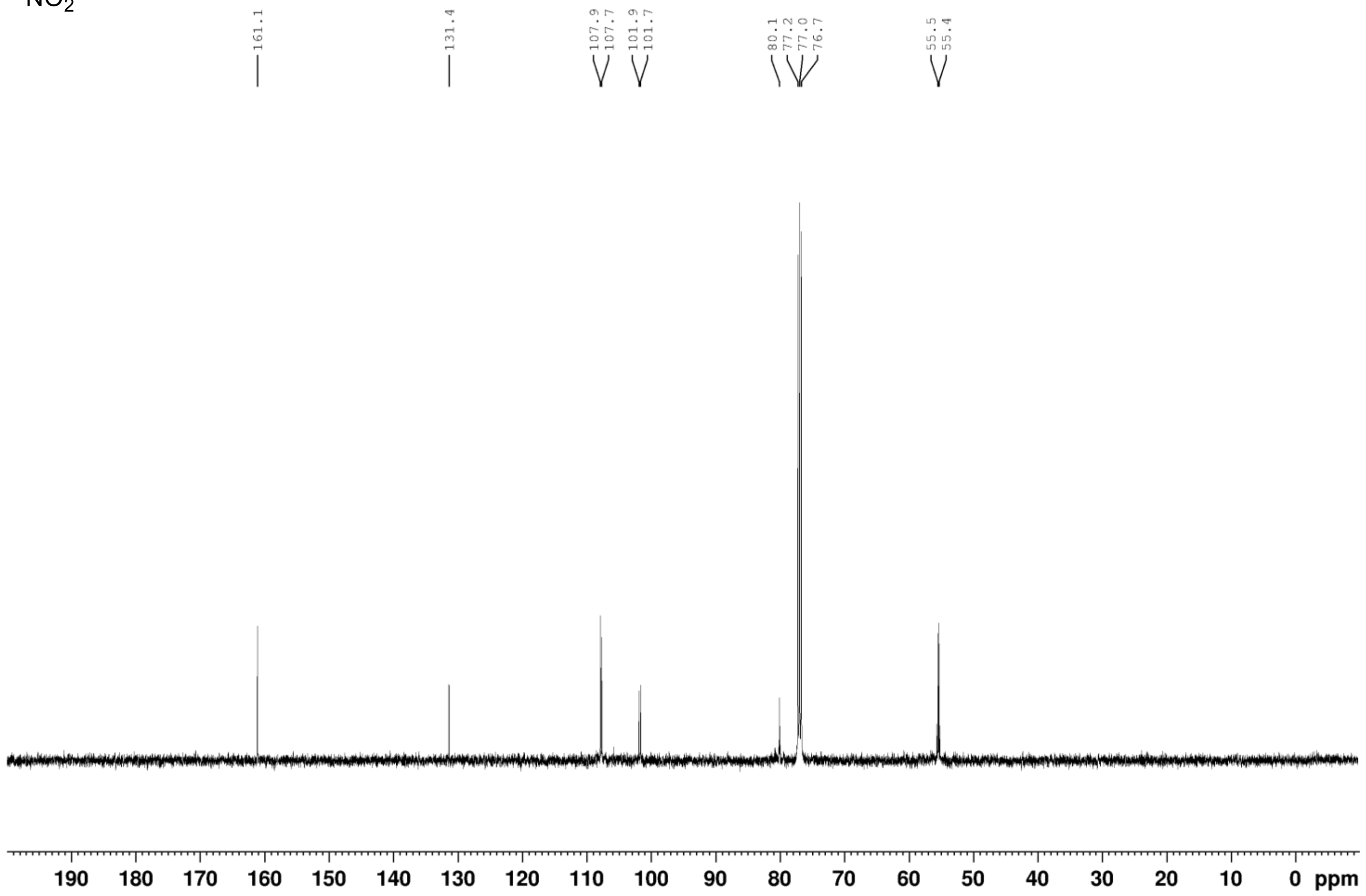

Figure S65. ¹H NMR (400 MHz, $CDCl_3$) of **3g**.

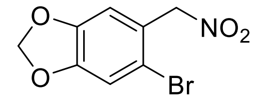

3g

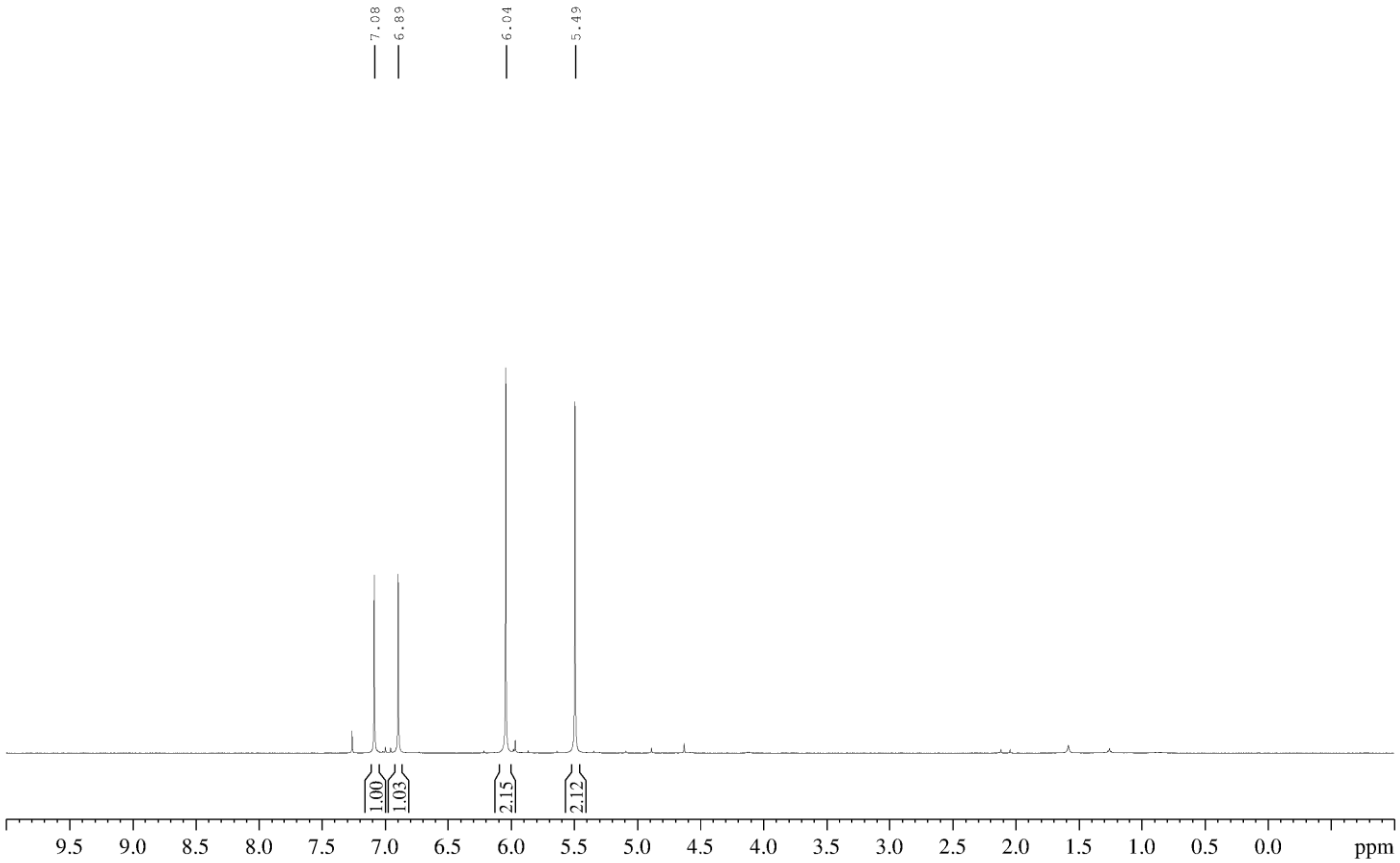

Figure S66. **^{13}C NMR** (101 MHz, $CDCl_3$) of **3g**.

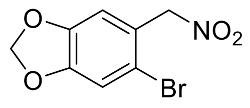

3g

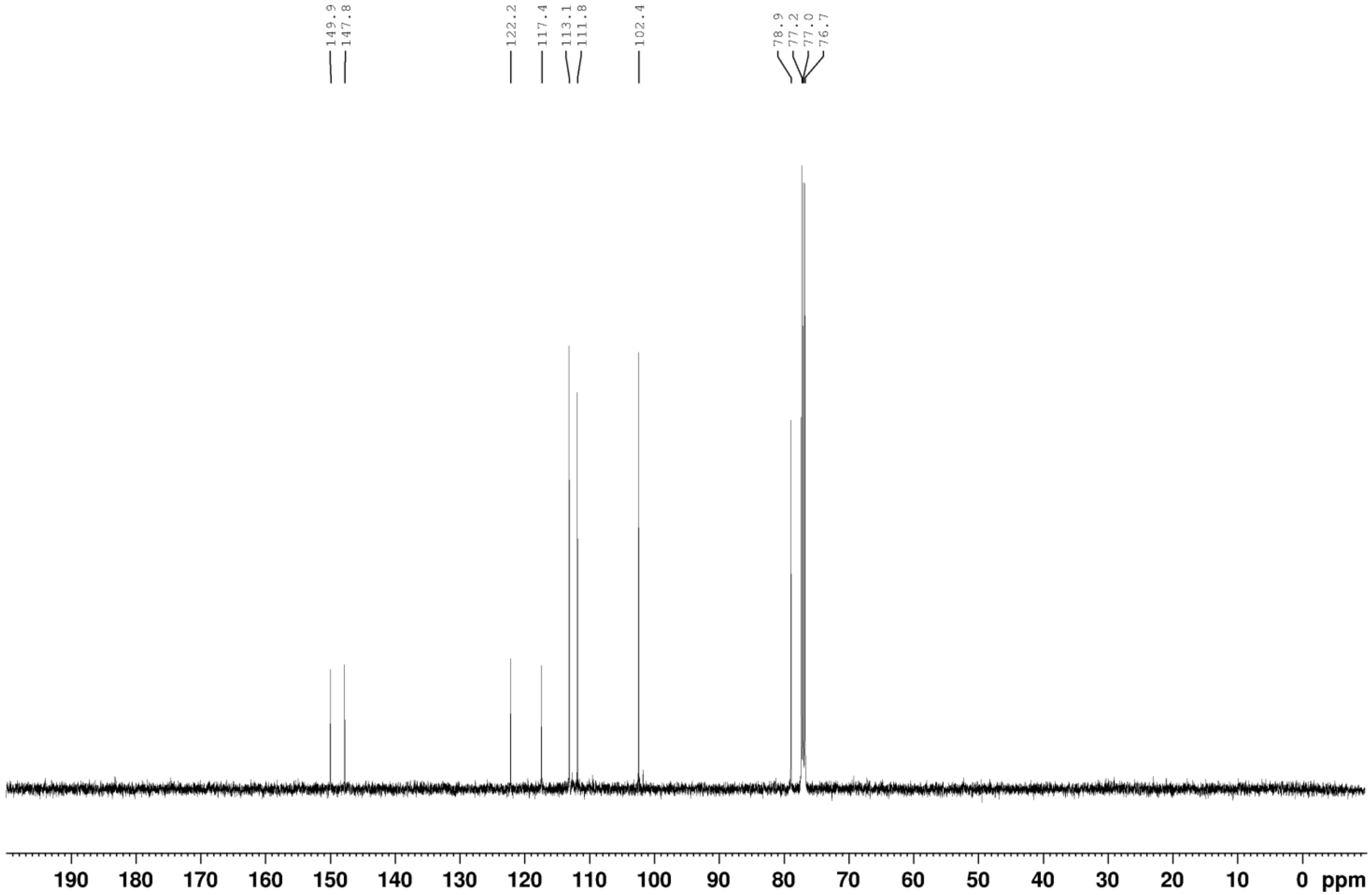

Figure S67. **¹H NMR** (400 MHz, $CDCl_3$) of **5a**.

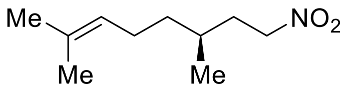

5a

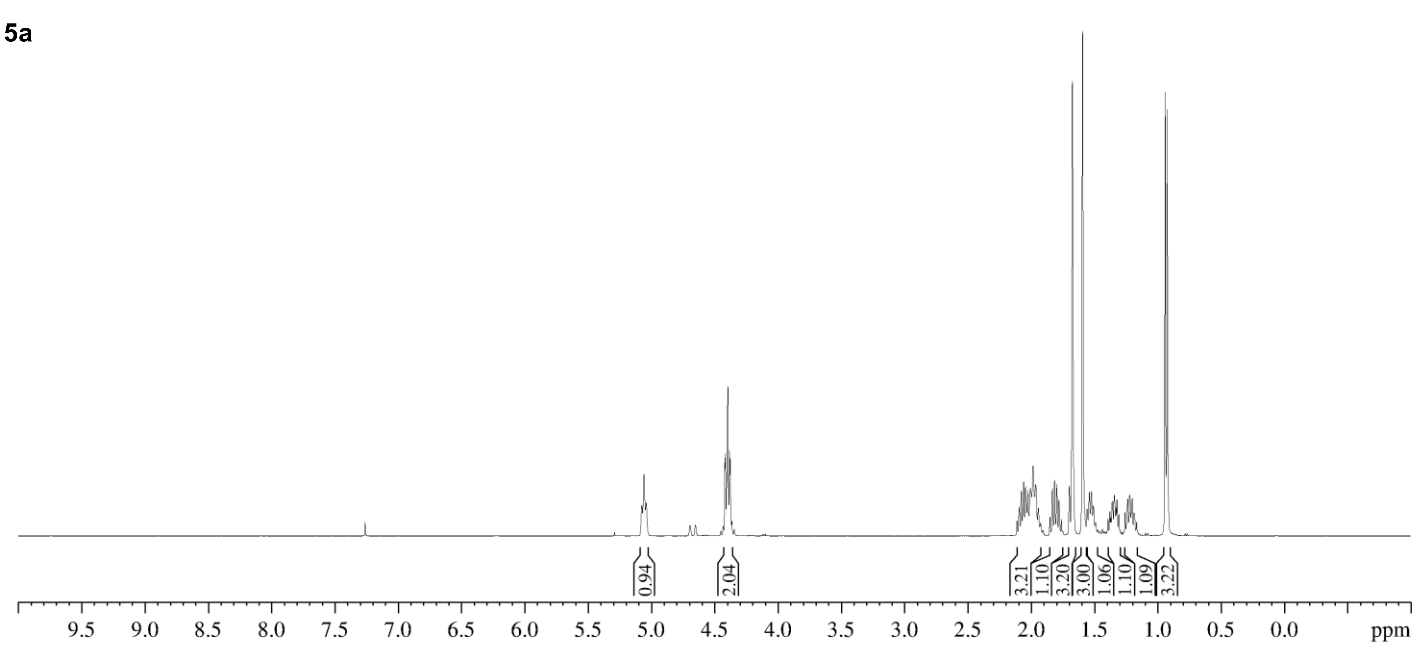

Figure S68. **^{13}C NMR** (101 MHz, $CDCl_3$) of **5a**.

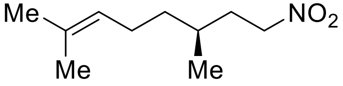

5a

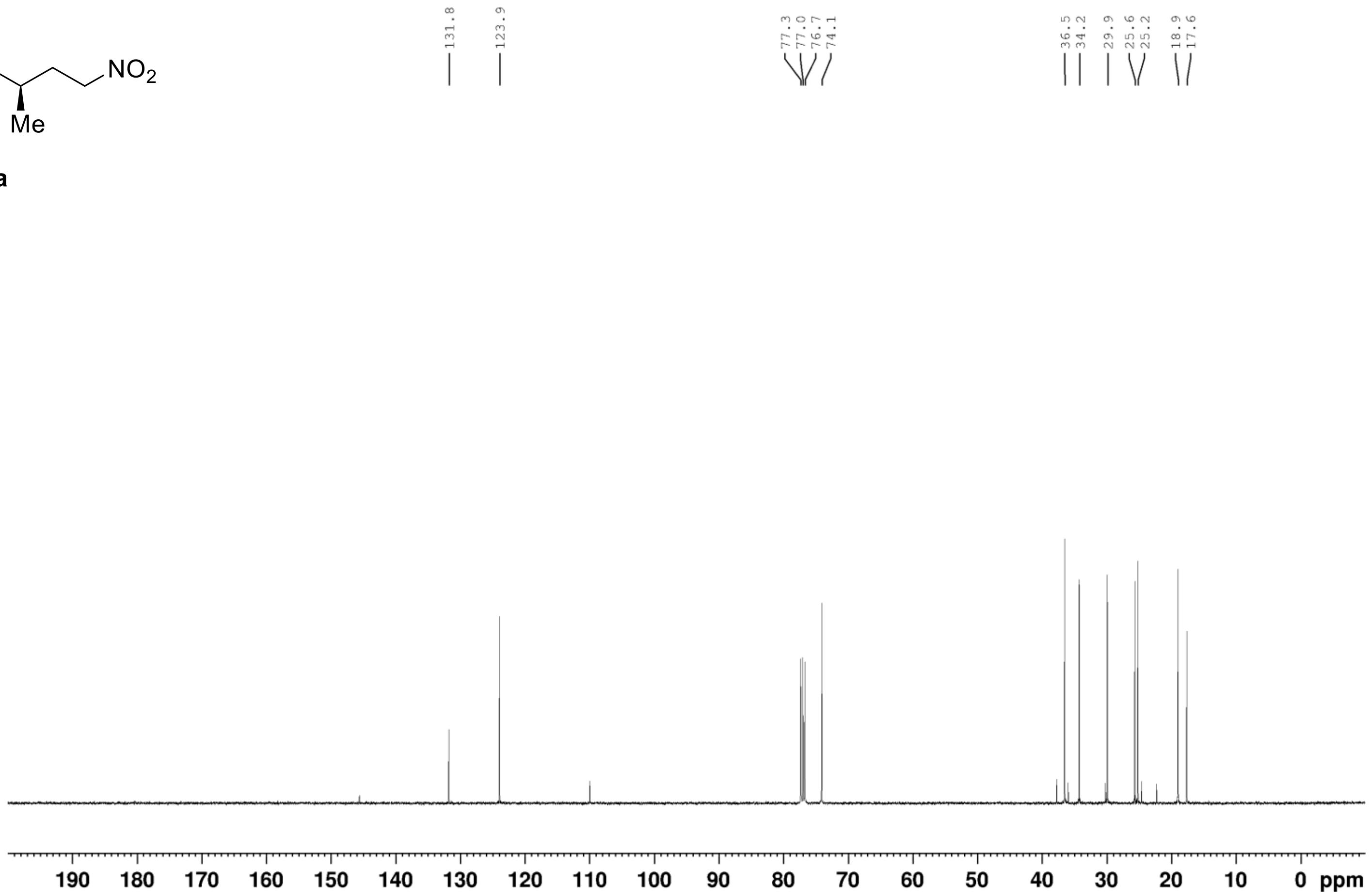

Figure S69. **^{1}H NMR** (400 MHz, $CDCl_3$) of **5b**.

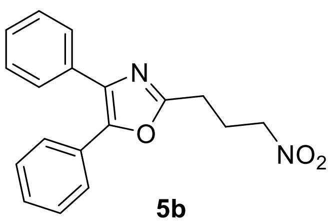

5b

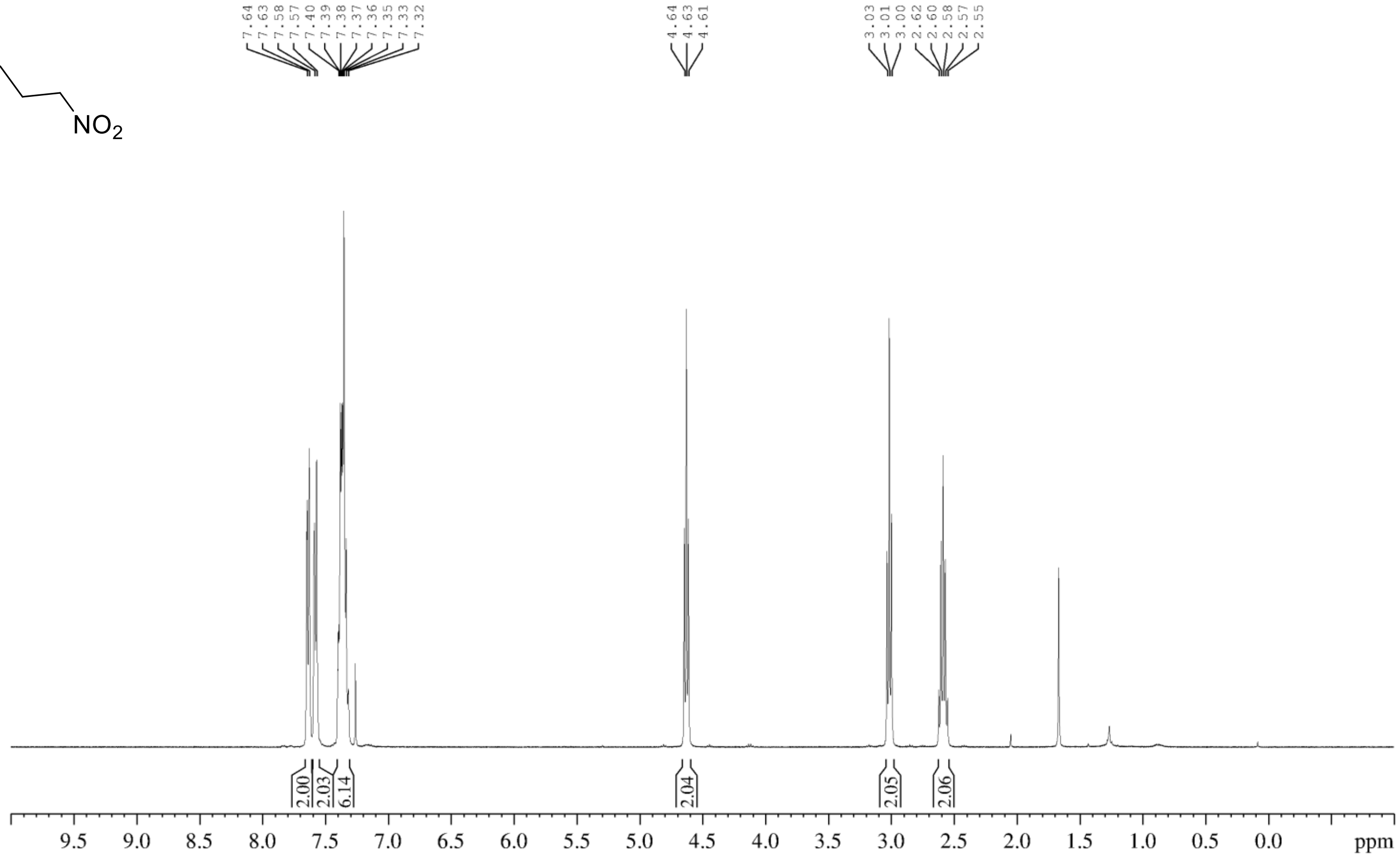

Figure S70. **^{13}C NMR** (101 MHz, $CDCl_3$) of **5b**.

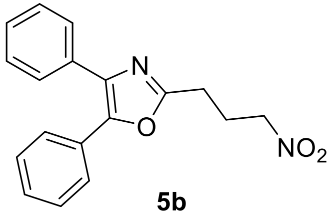

5b

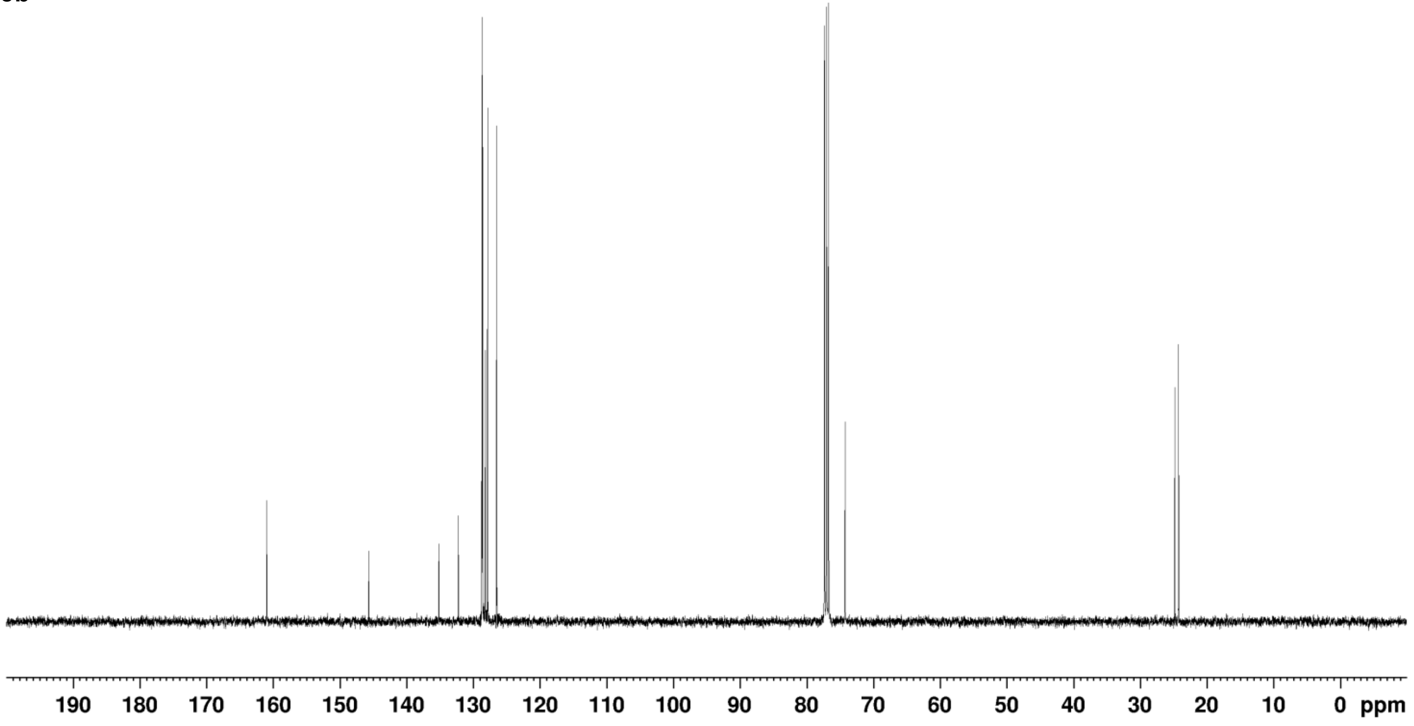

Figure S71. **^{1}H NMR** (500 MHz, $CDCl_3$) of **5c**.

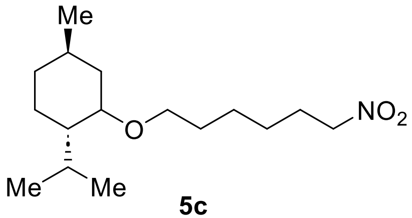

5c

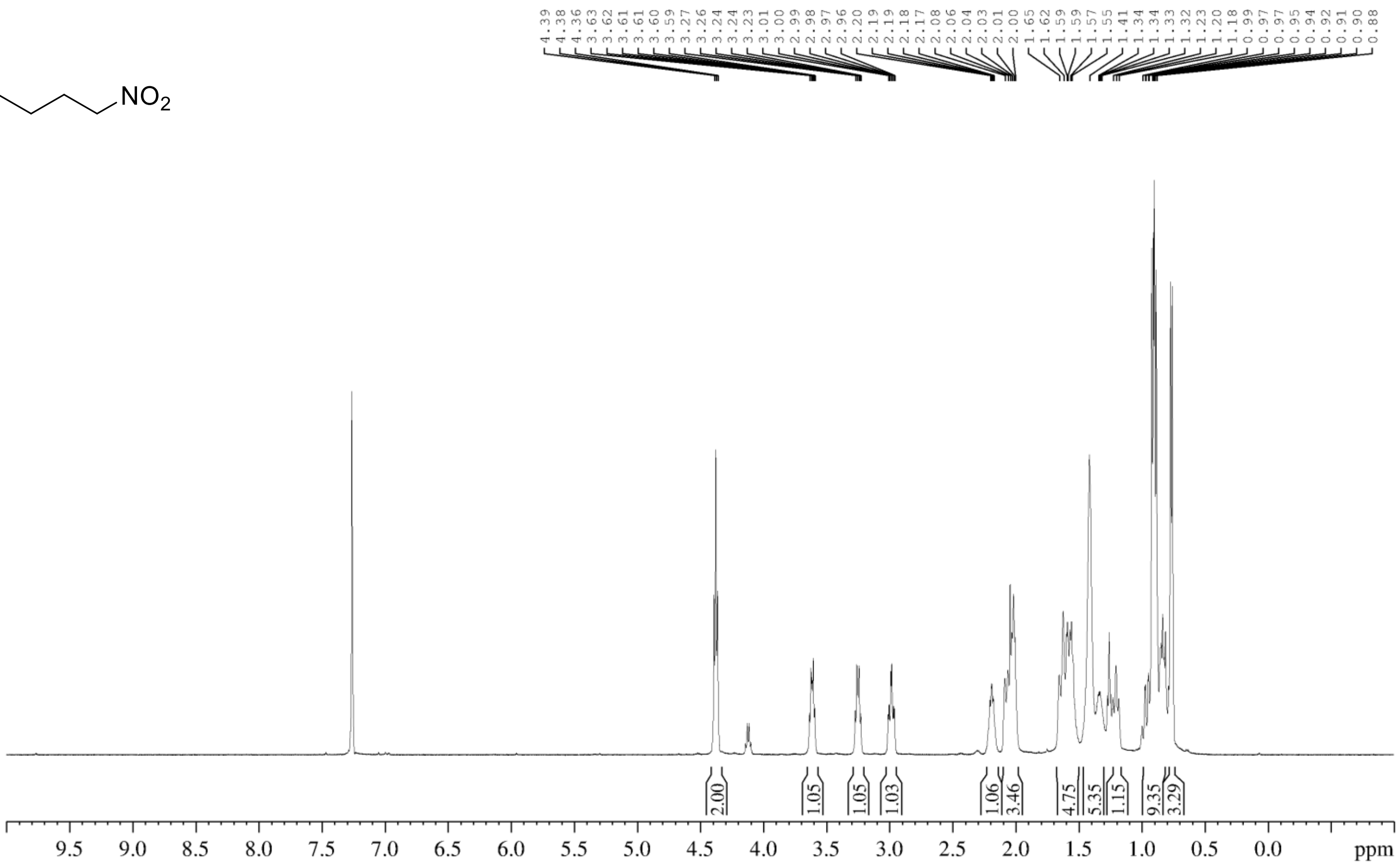

Figure S72. **^{13}C NMR** (126 MHz, $CDCl_3$) of **5c**

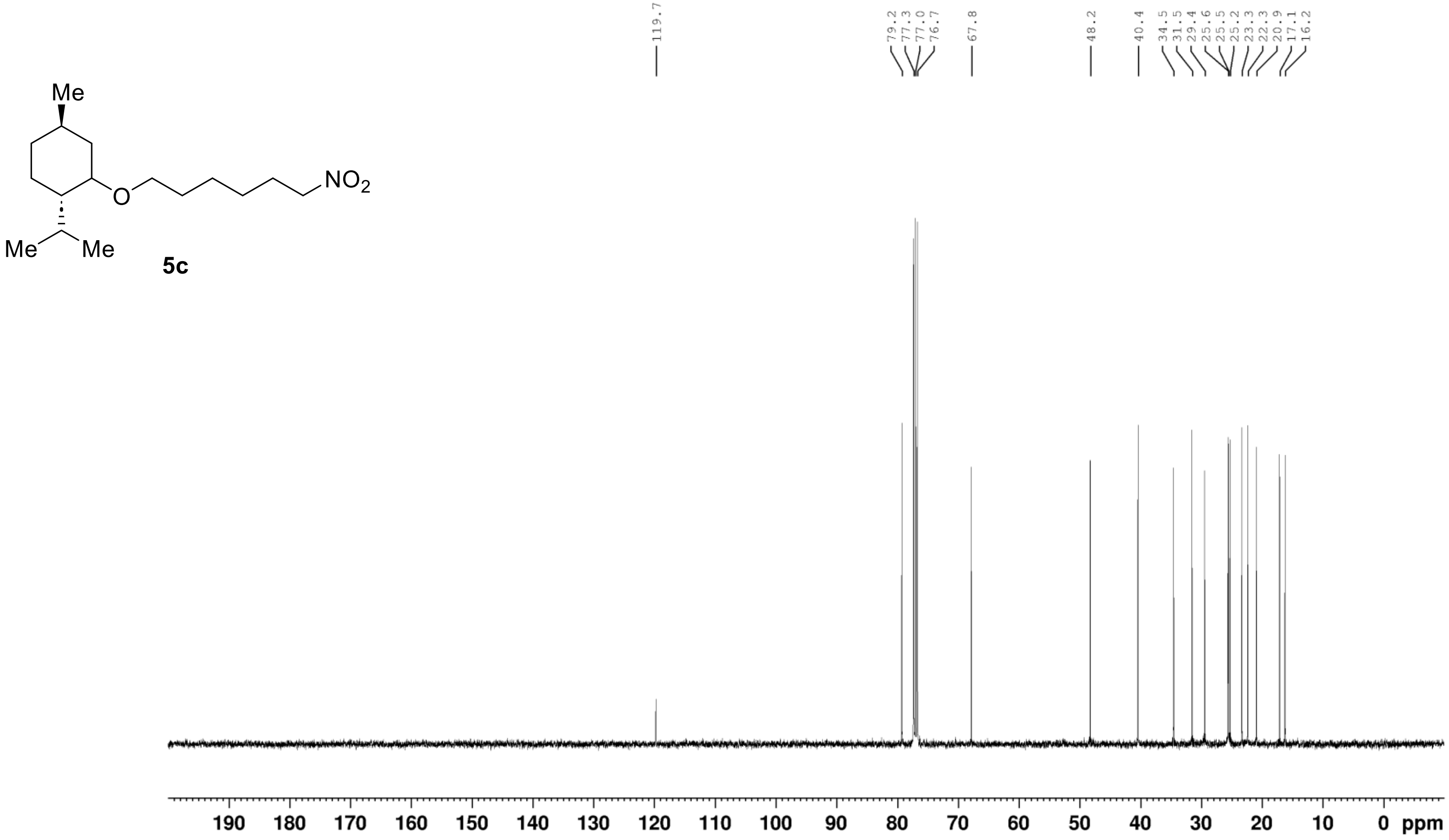

Figure S73. **¹H NMR** (500 MHz, $CDCl_3$) of **5d**.

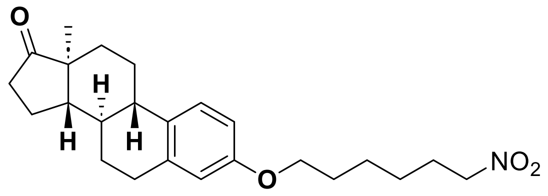

5d

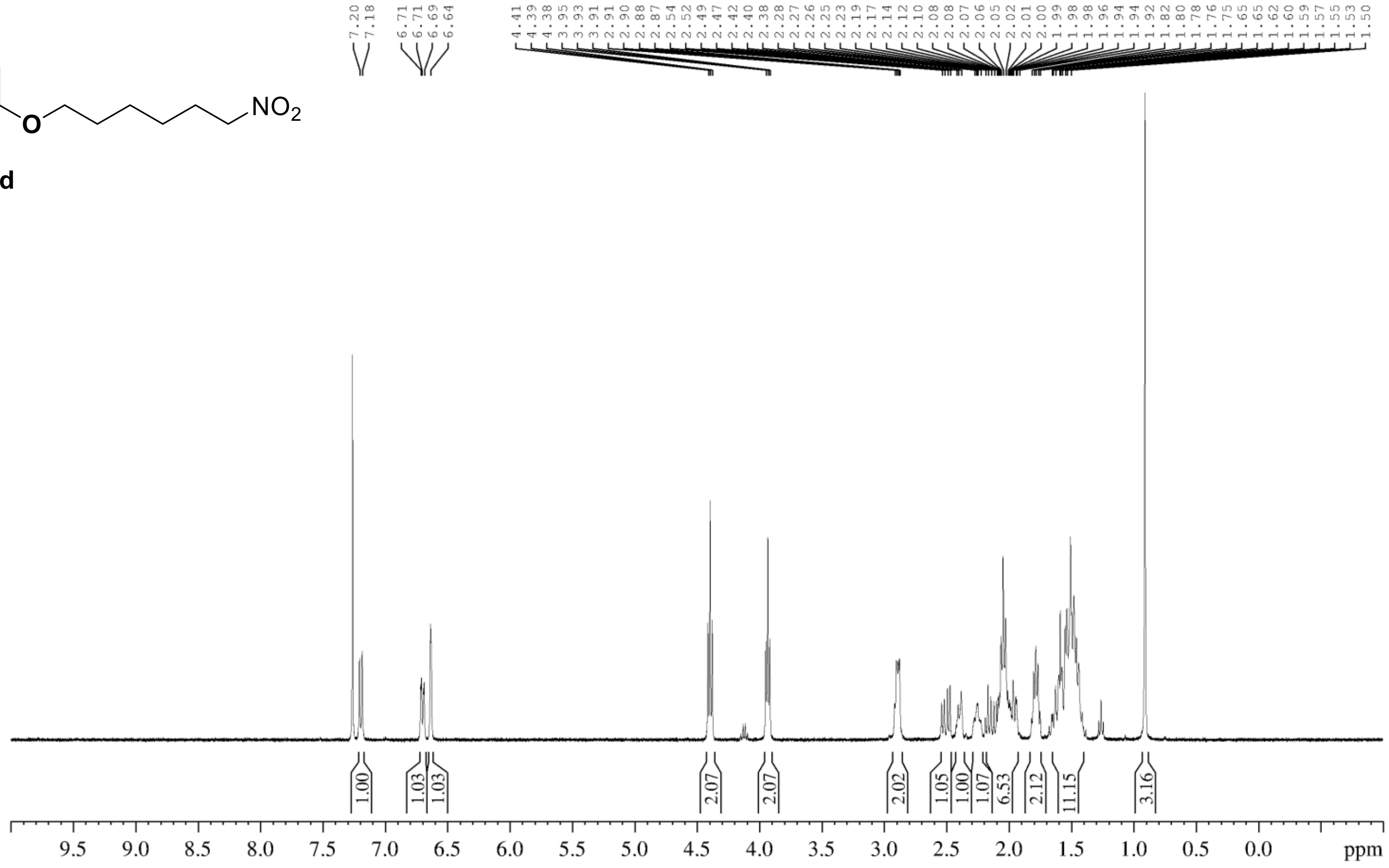

Figure S74. **^{13}C NMR** (126 MHz, $CDCl_3$) of **5d**.

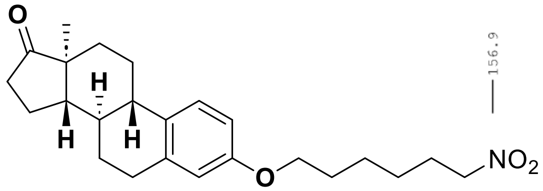

5d

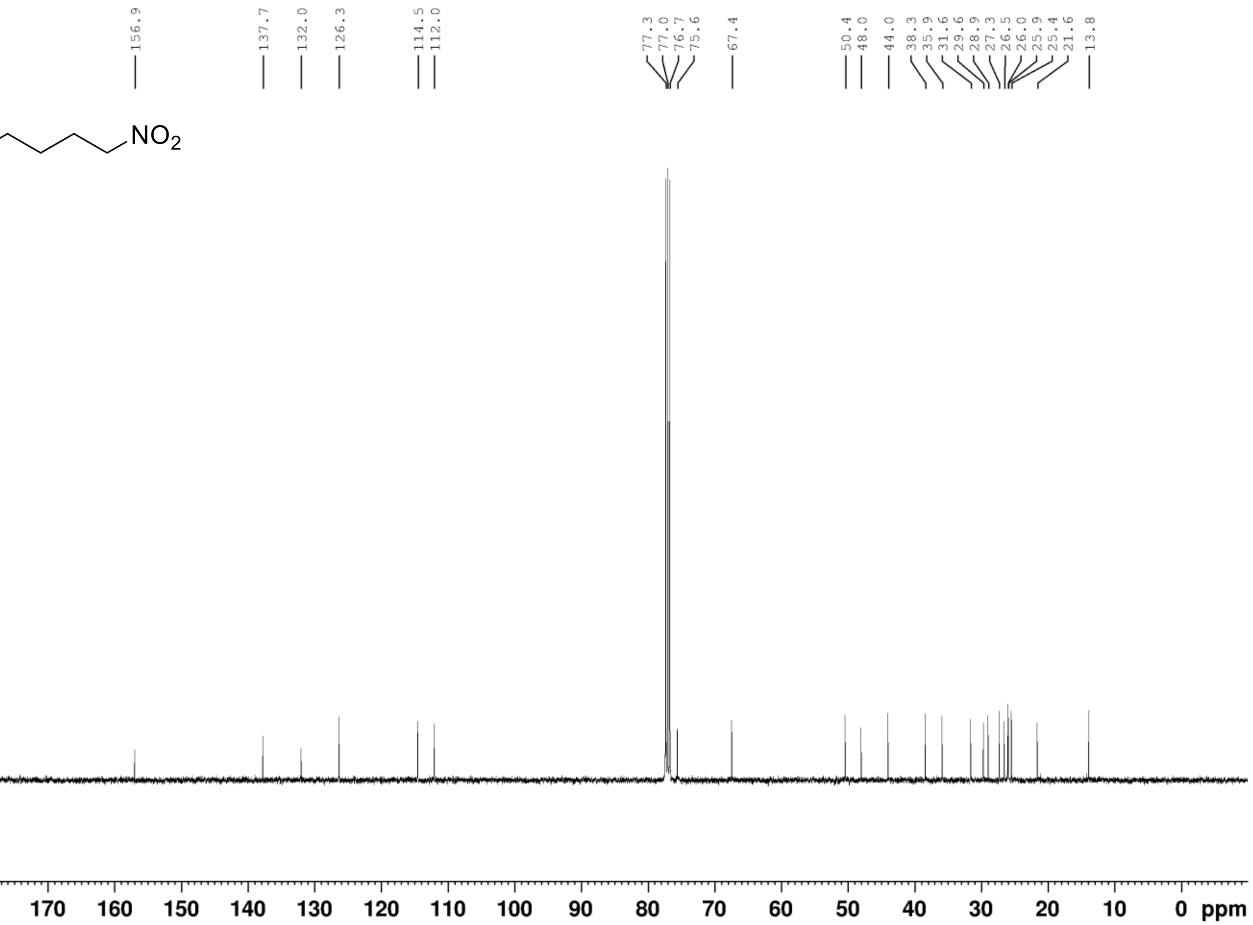

Figure S75. **^{1}H NMR** (500 MHz, $CDCl_3$) of **7a-xiNO$_2$**.

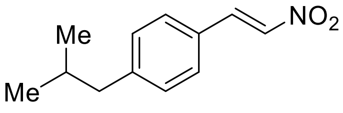

7a-xiNO$_2$

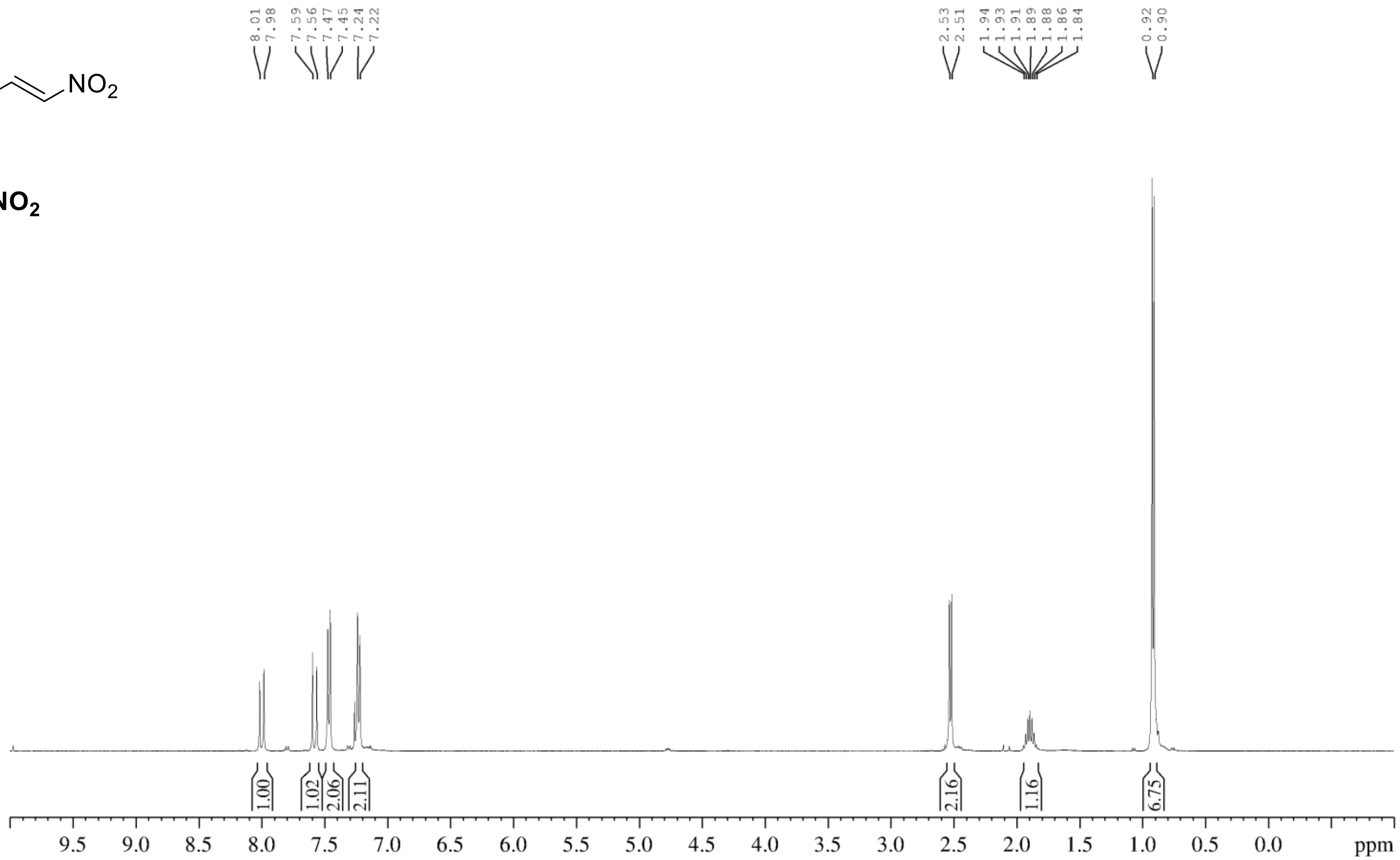

Figure S76. **^{13}C NMR** (126 MHz, $CDCl_3$) of **7a-xiNO$_2$**.

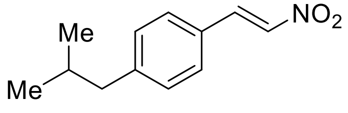

7a-xiNO$_2$

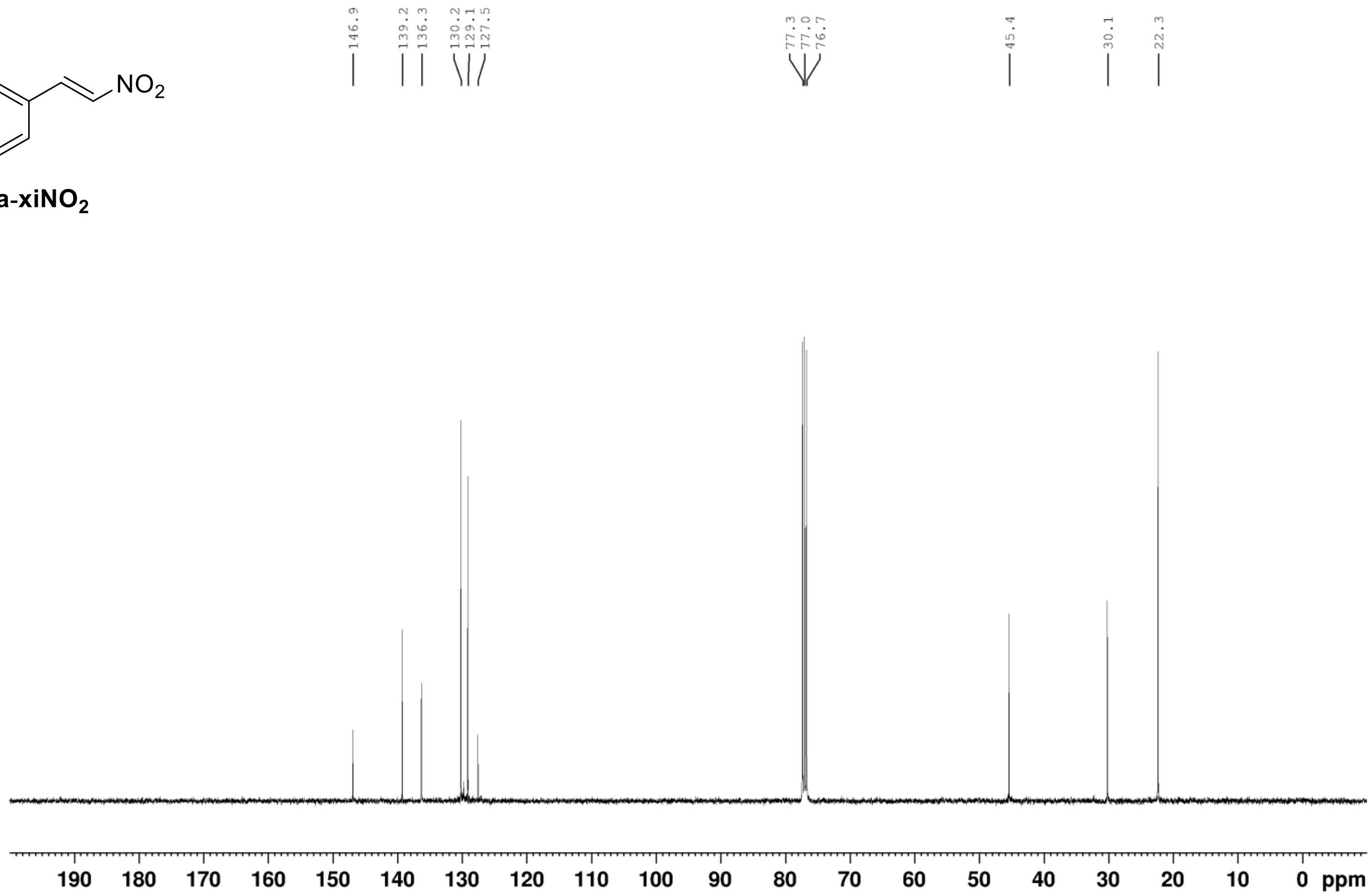

Figure S77. ^{1}**H NMR** (500 MHz, $CDCl_3$) of **7a**.

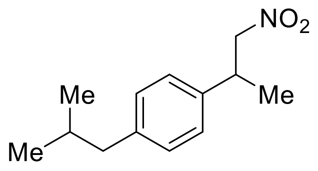

7a

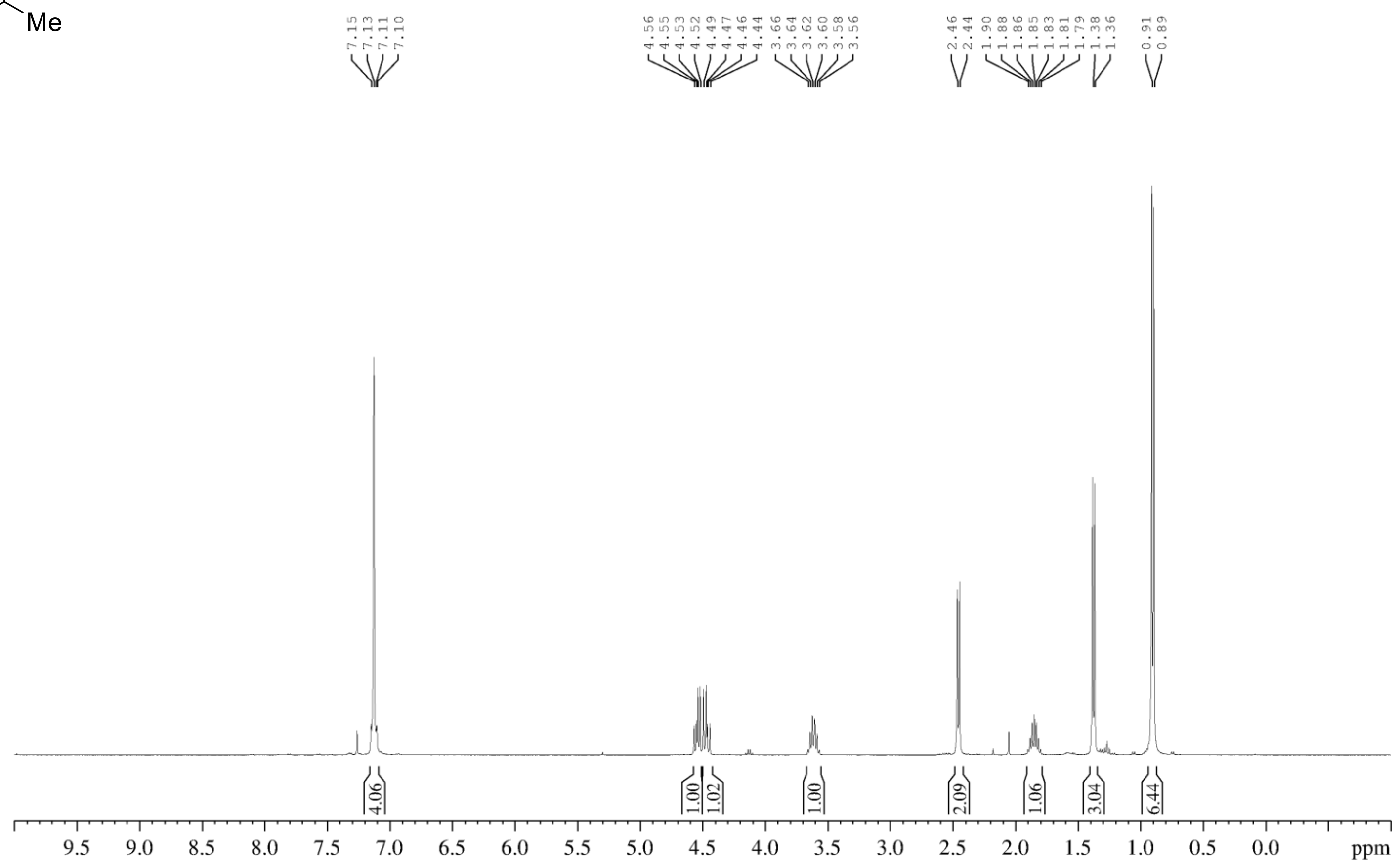

Figure S78. **^{13}C NMR** (126 MHz, $CDCl_3$) of **7a**.

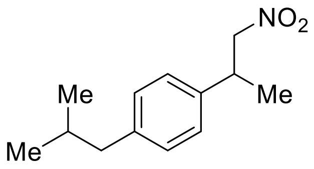

7a

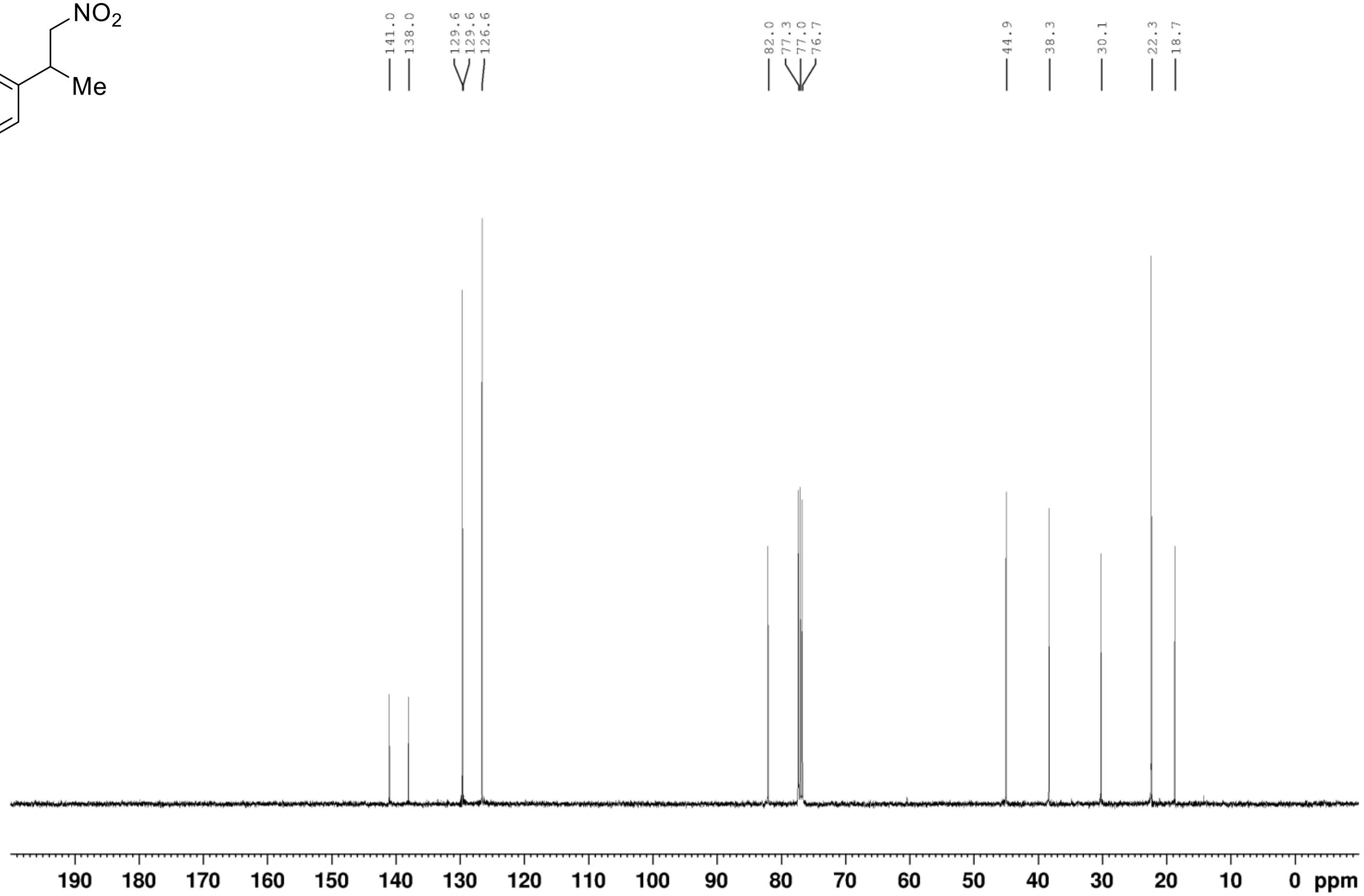

Figure S79. **¹H NMR** (500 MHz, $CDCl_3$) of **7b-xiNO₂**.

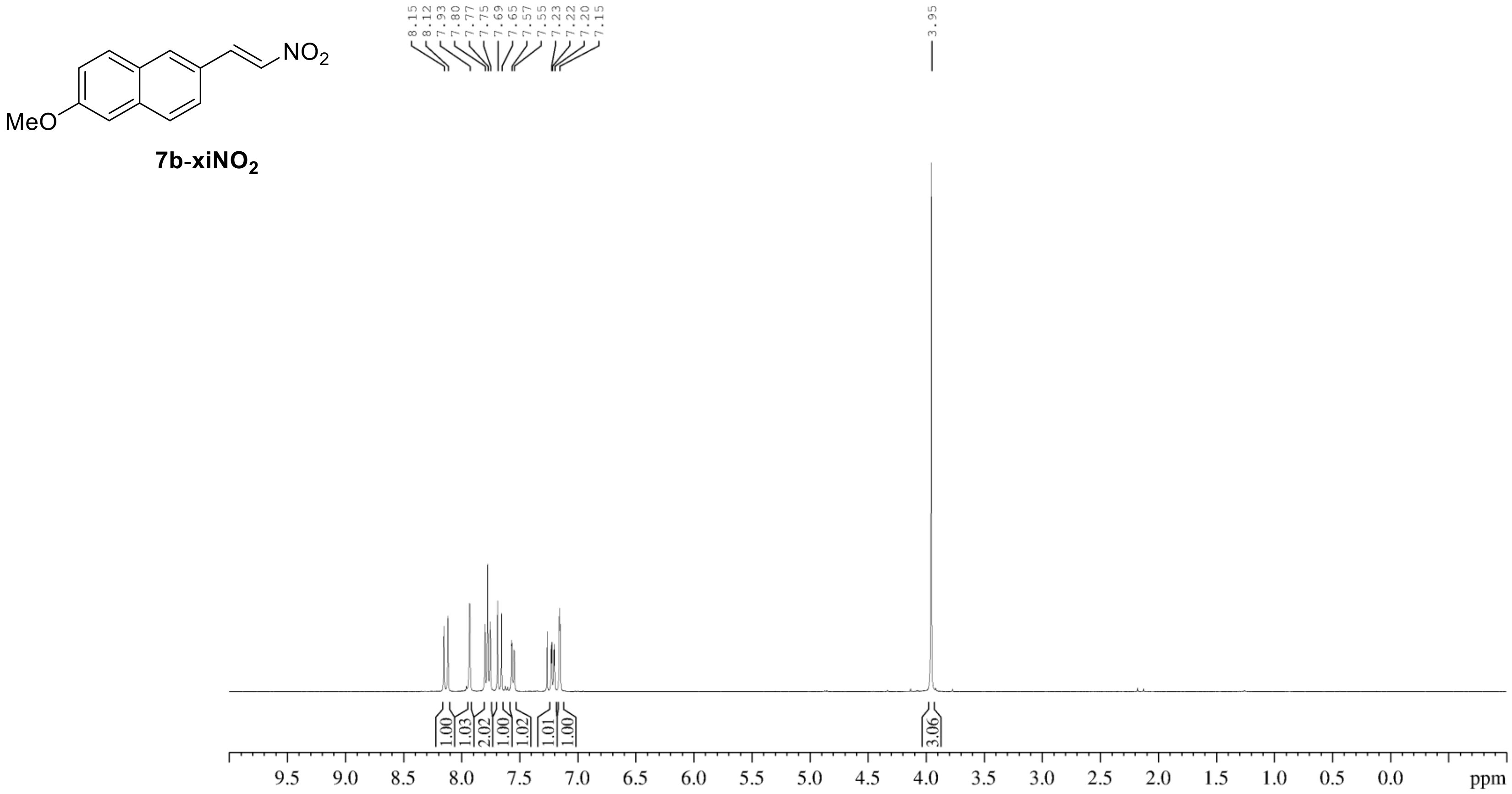

Figure S80. ^{13}**C NMR** (126 MHz, $CDCl_3$) of **7b-xiNO$_2$.**

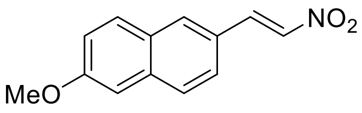

7b-xiNO$_2$

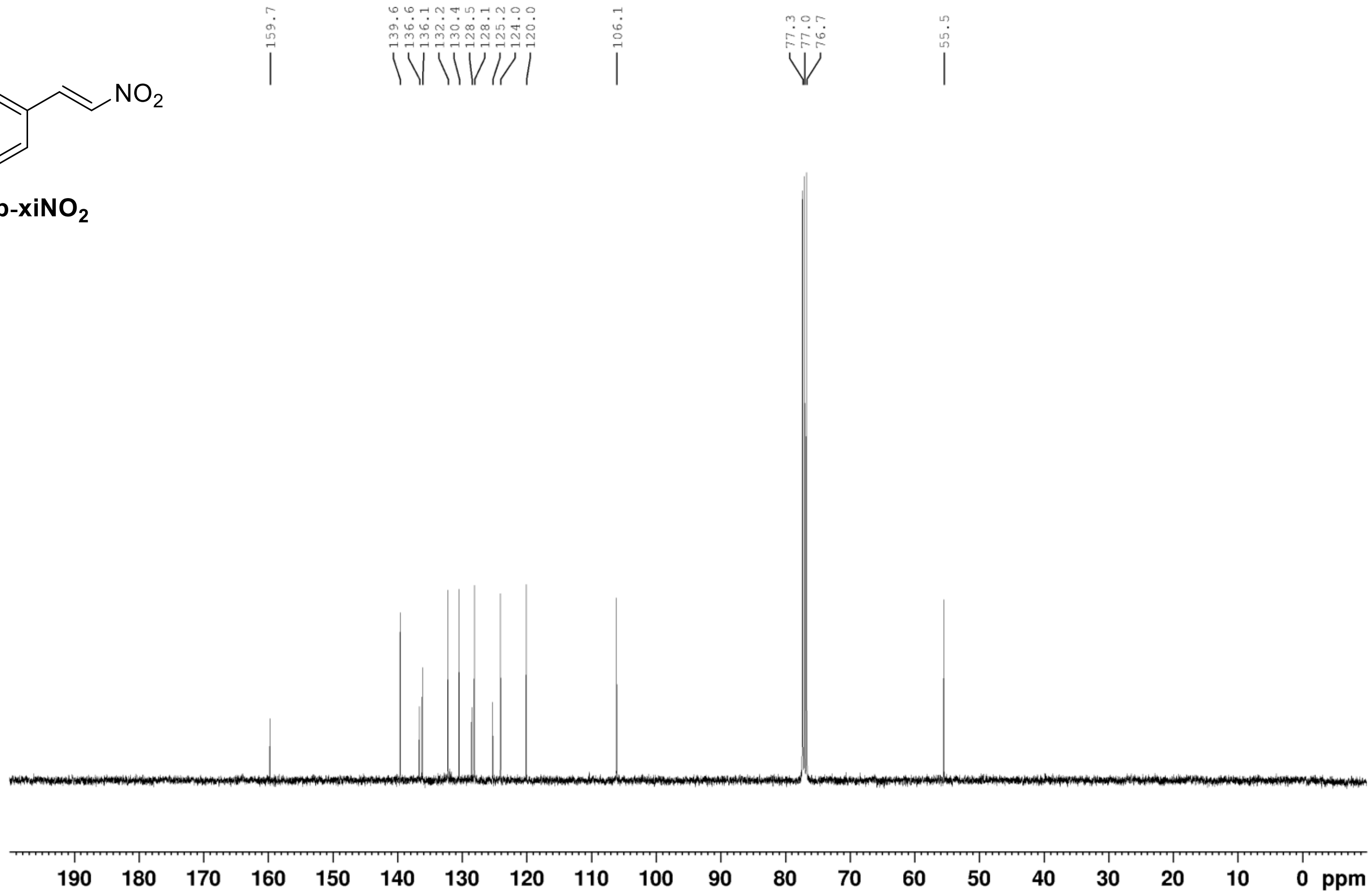

Figure S81. **^{1}H NMR** (500 MHz, $CDCl_3$) of **7b**.

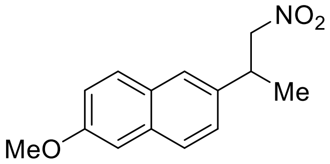

7b

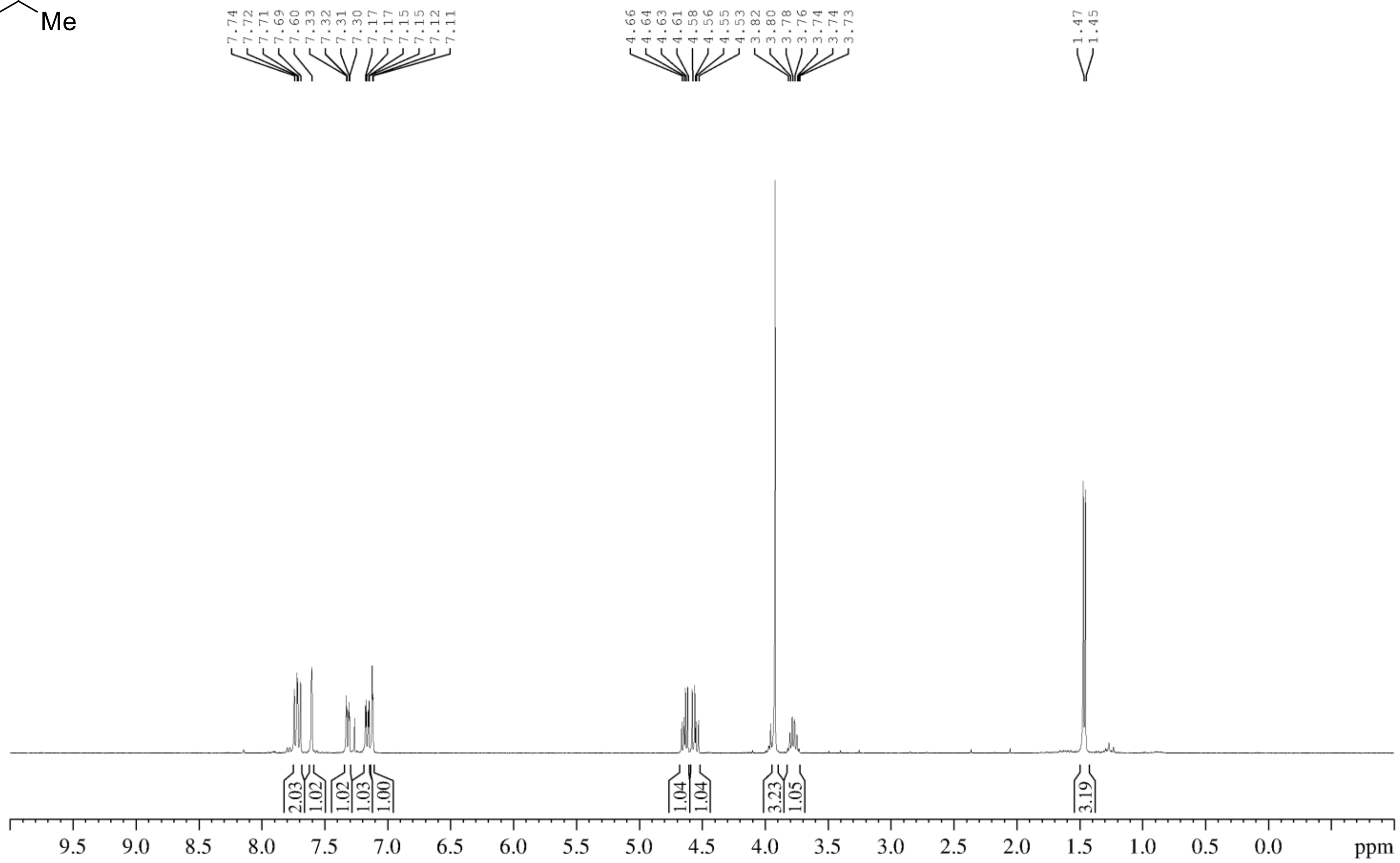

Figure S82. **^{13}C NMR** (126 MHz, $CDCl_3$) of **7b**.

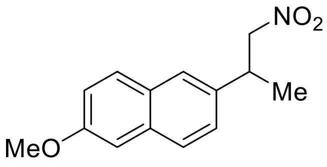

7b

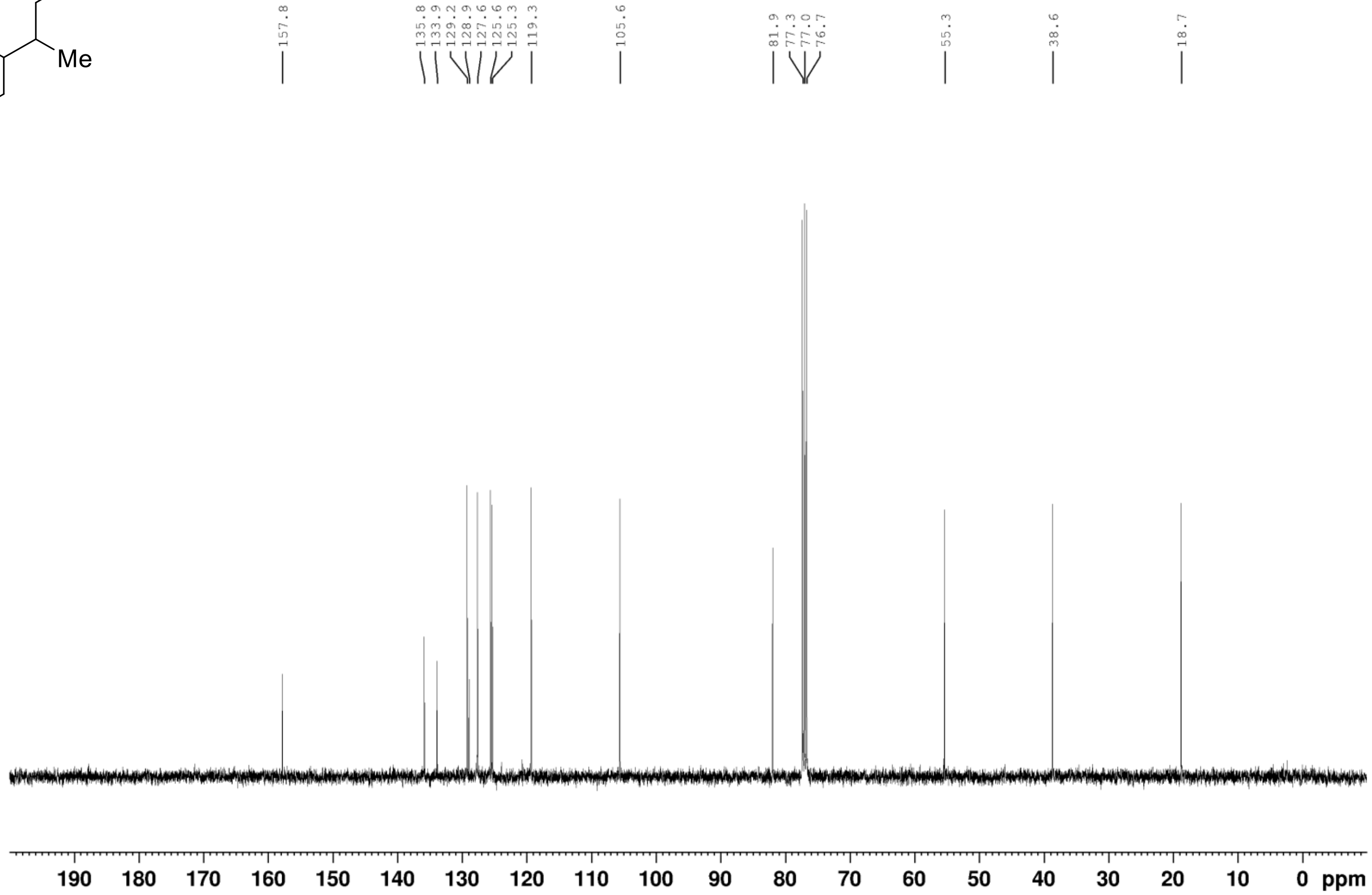

Figure S83. **^{1}H NMR** (500 MHz, $CDCl_3$) of **7c**.

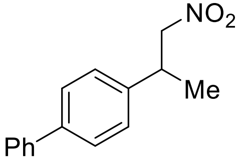

7c

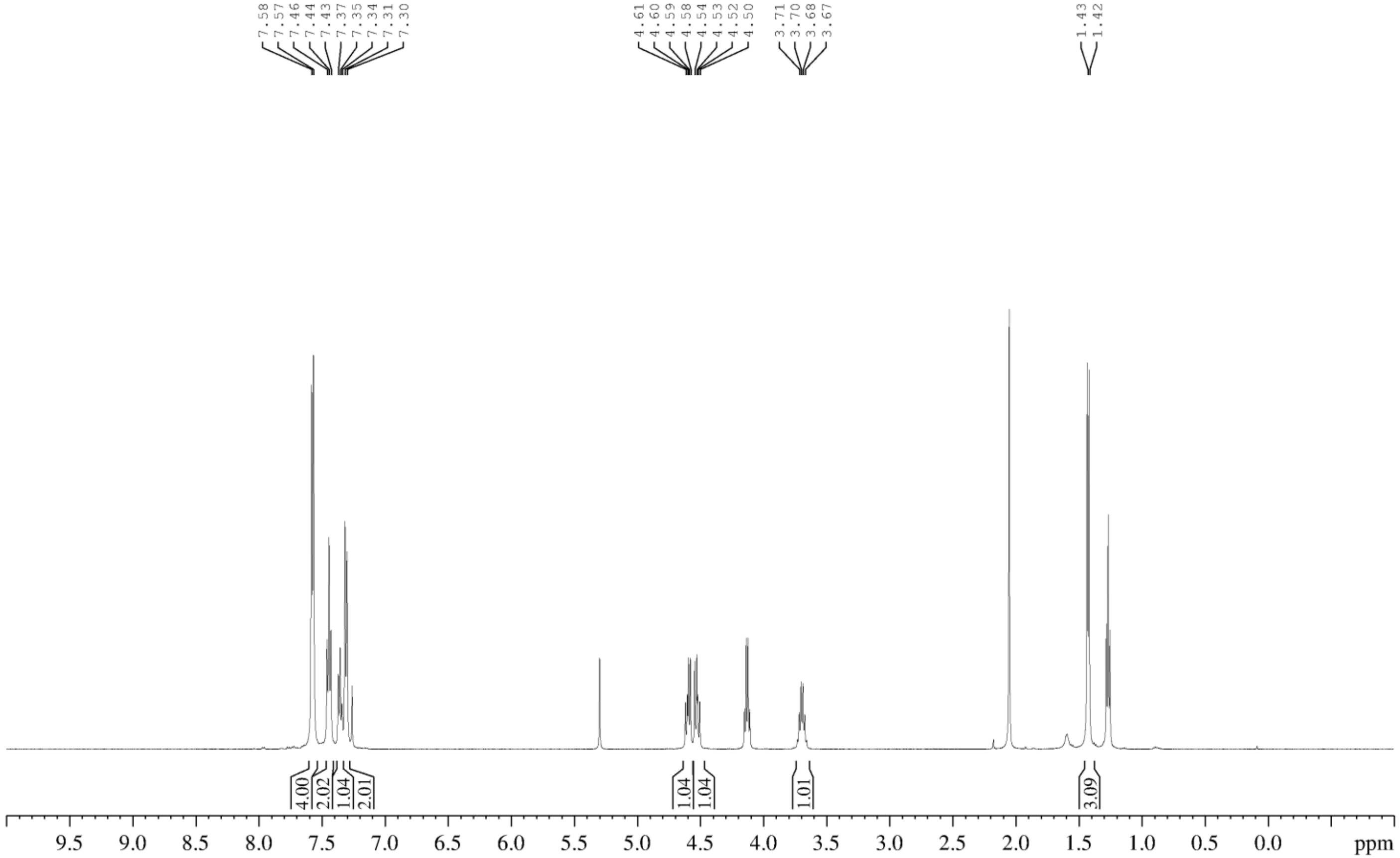

Figure S84. **^{13}C NMR** (126 MHz, $CDCl_3$) of **7c**.

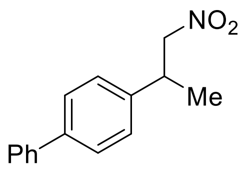

7c

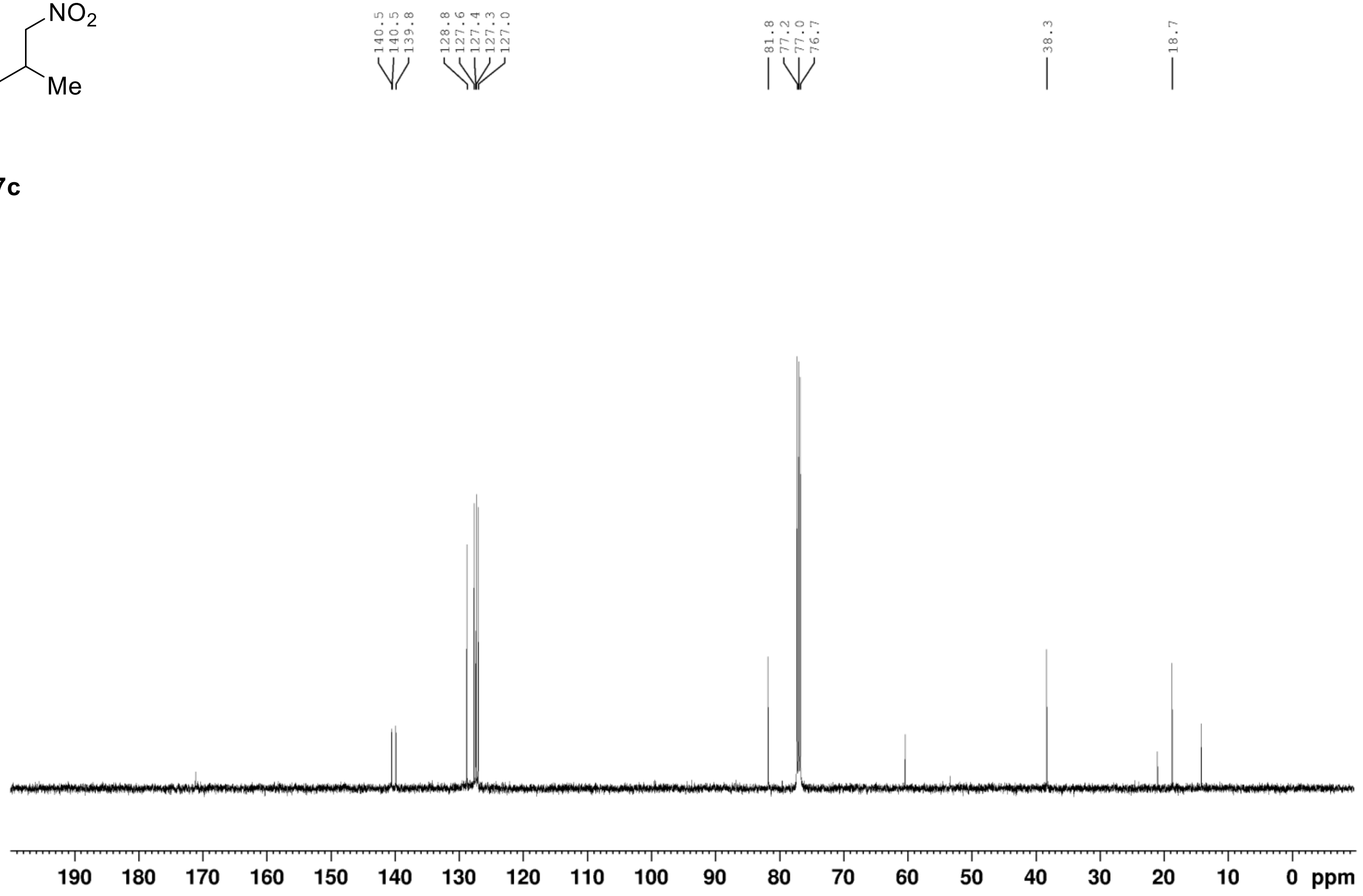

Figure S85. **^{1}H NMR** (500 MHz, $CDCl_3$) of **9**.

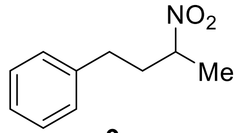

9

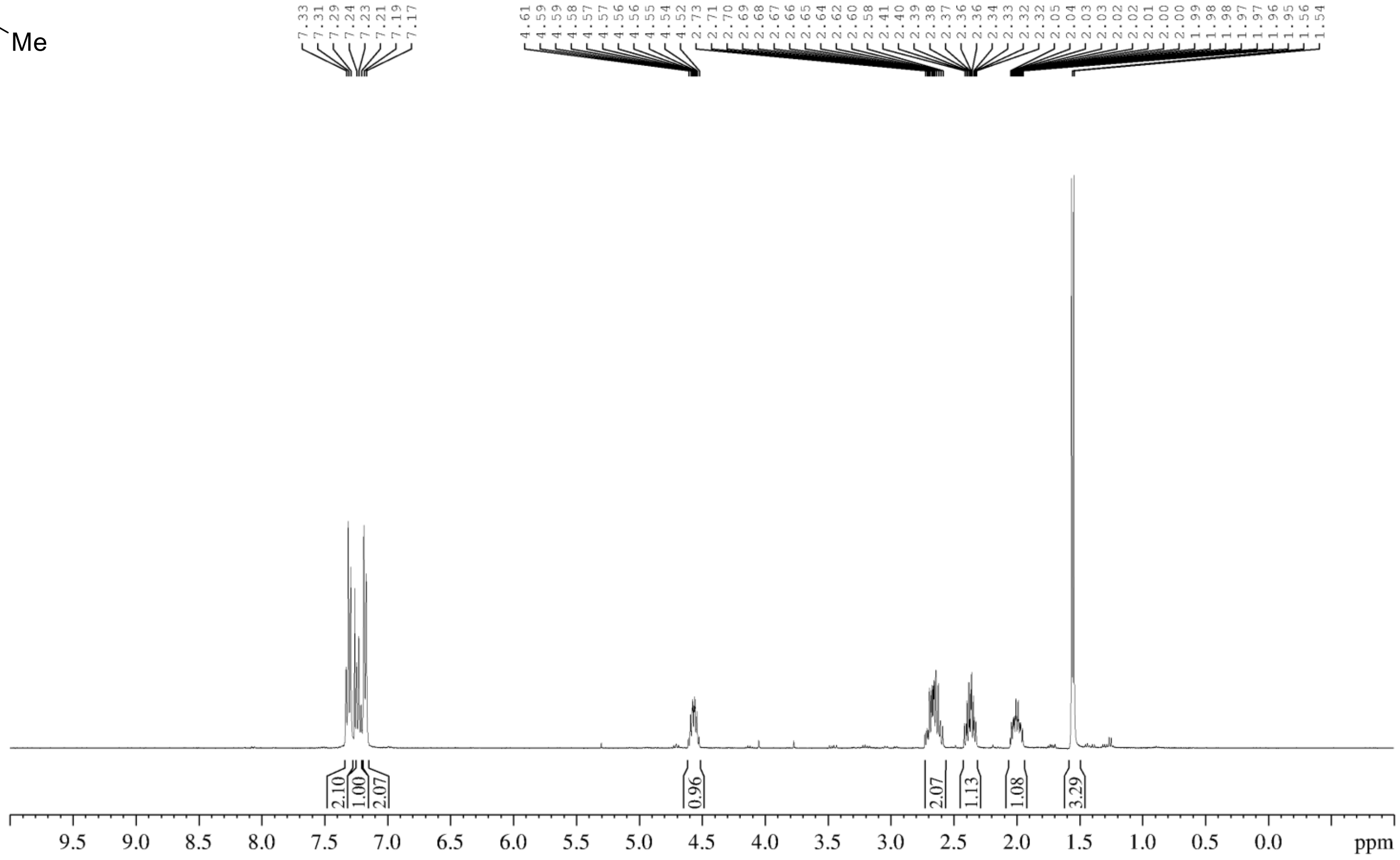

Figure S86. **^{13}C NMR** (126 MHz, $CDCl_3$) of **9**.

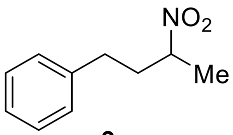

9

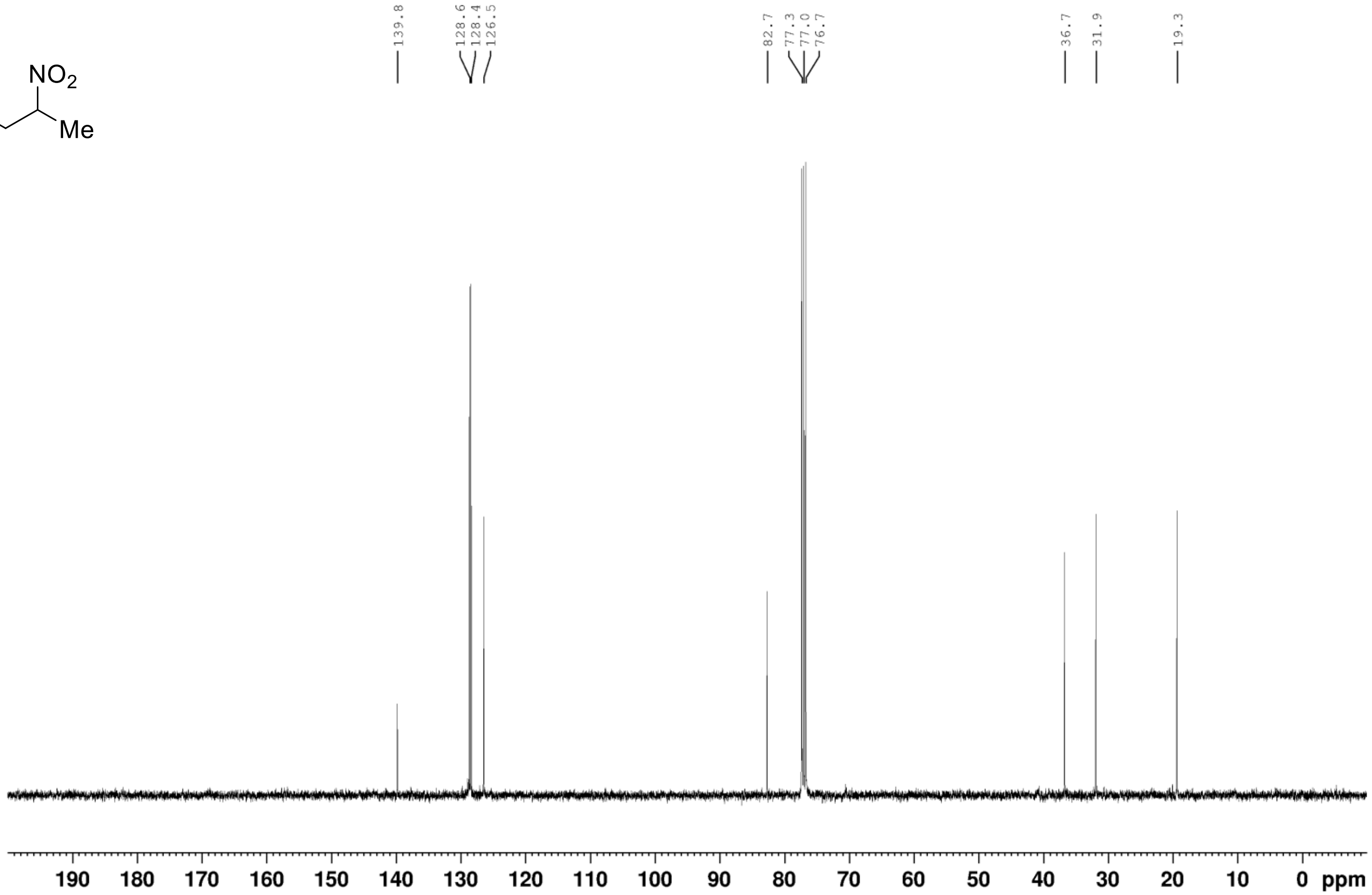

Figure S87. **^{1}H NMR** (500 MHz, $CDCl_3$) of **2a**.

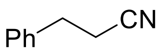

2a

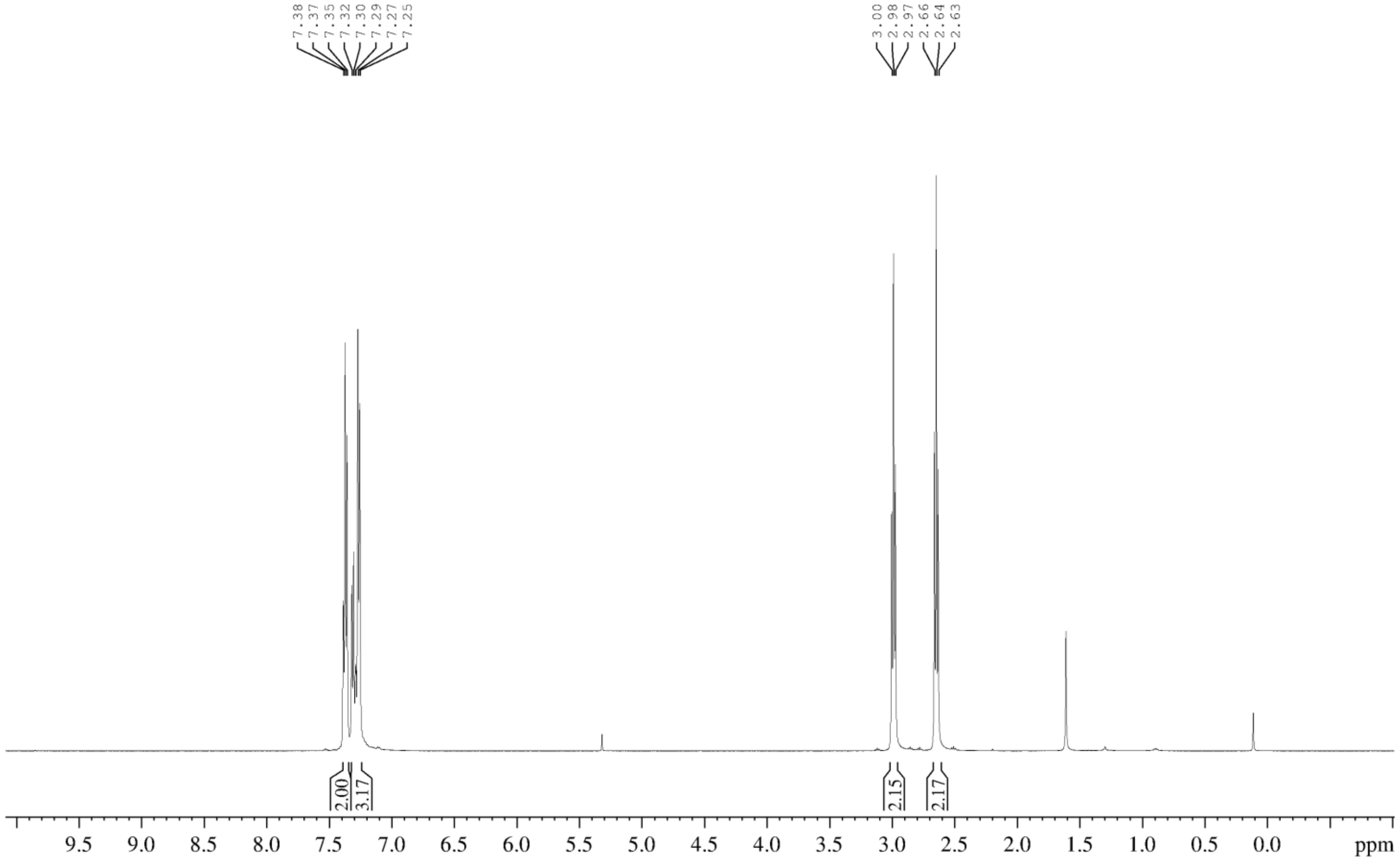

Figure S88. **^{13}C NMR** (126 MHz, $CDCl_3$) of **2a**.

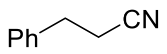

2a

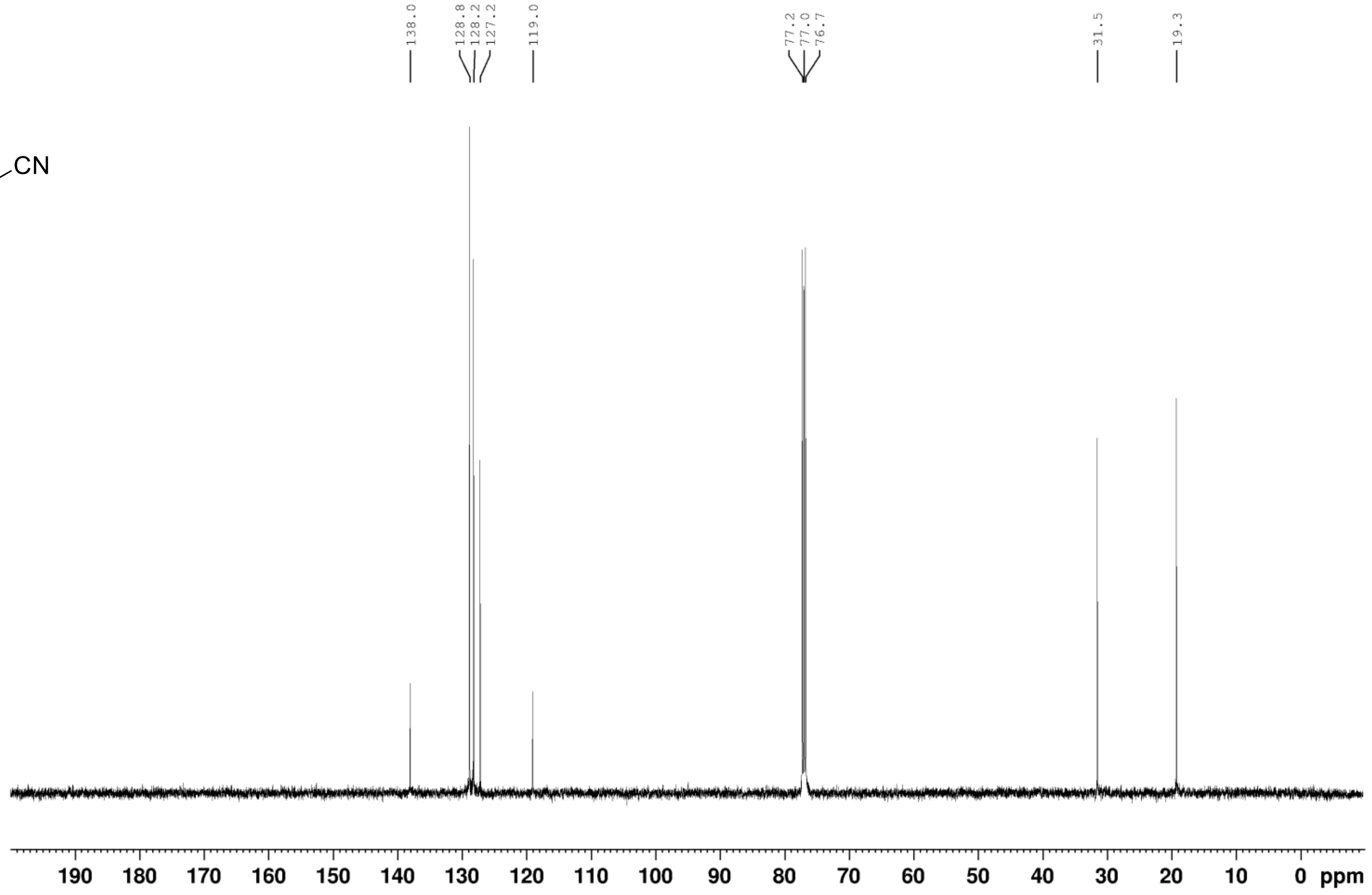

Figure S89. **^{1}H NMR** (500 MHz, $CDCl_3$) of **2b**.

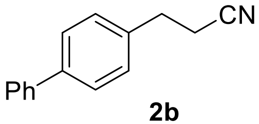

2b

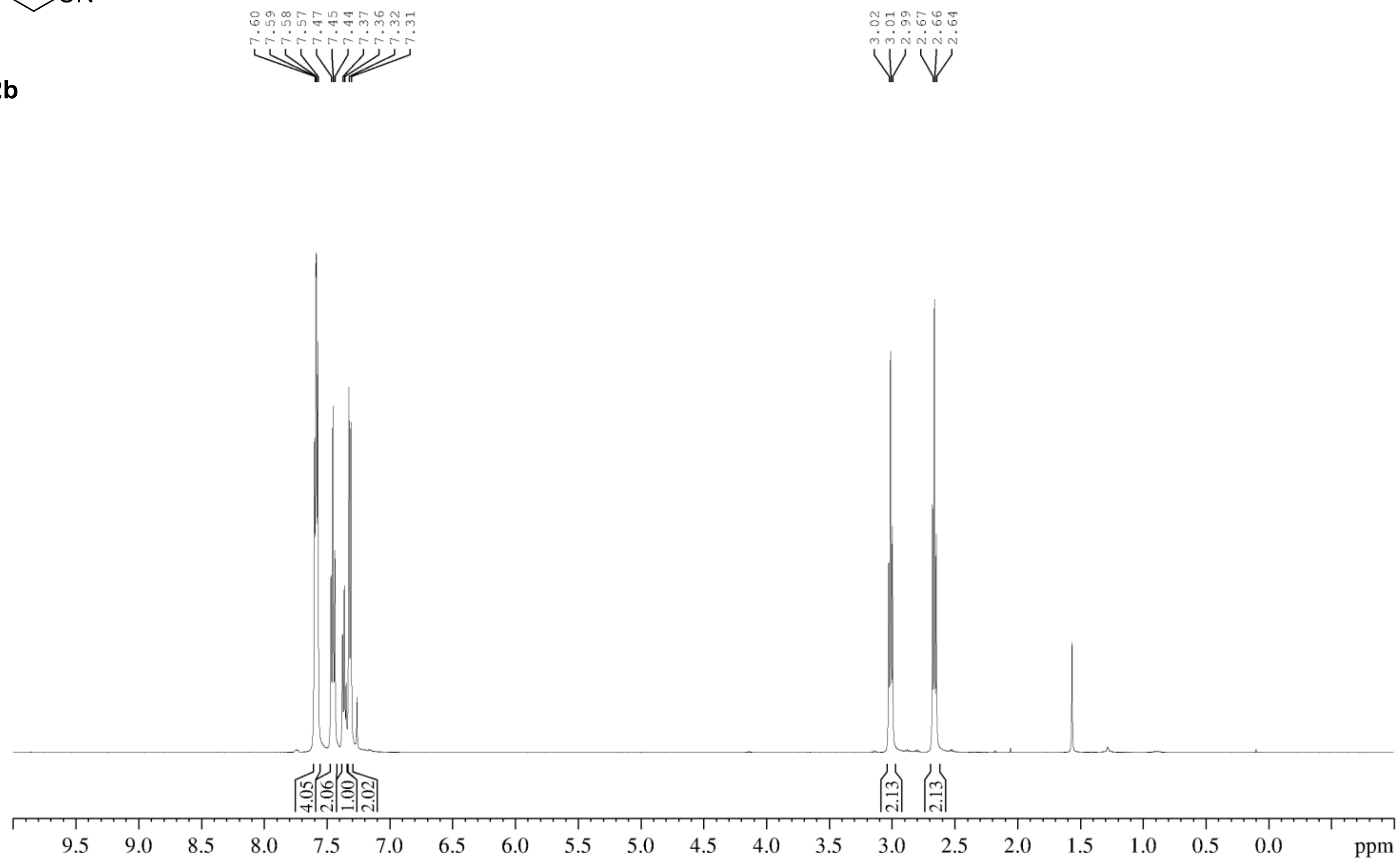

Figure S90. ^{13}**C NMR** (126 MHz, $CDCl_3$) of **2b**.

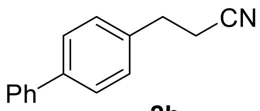

2b

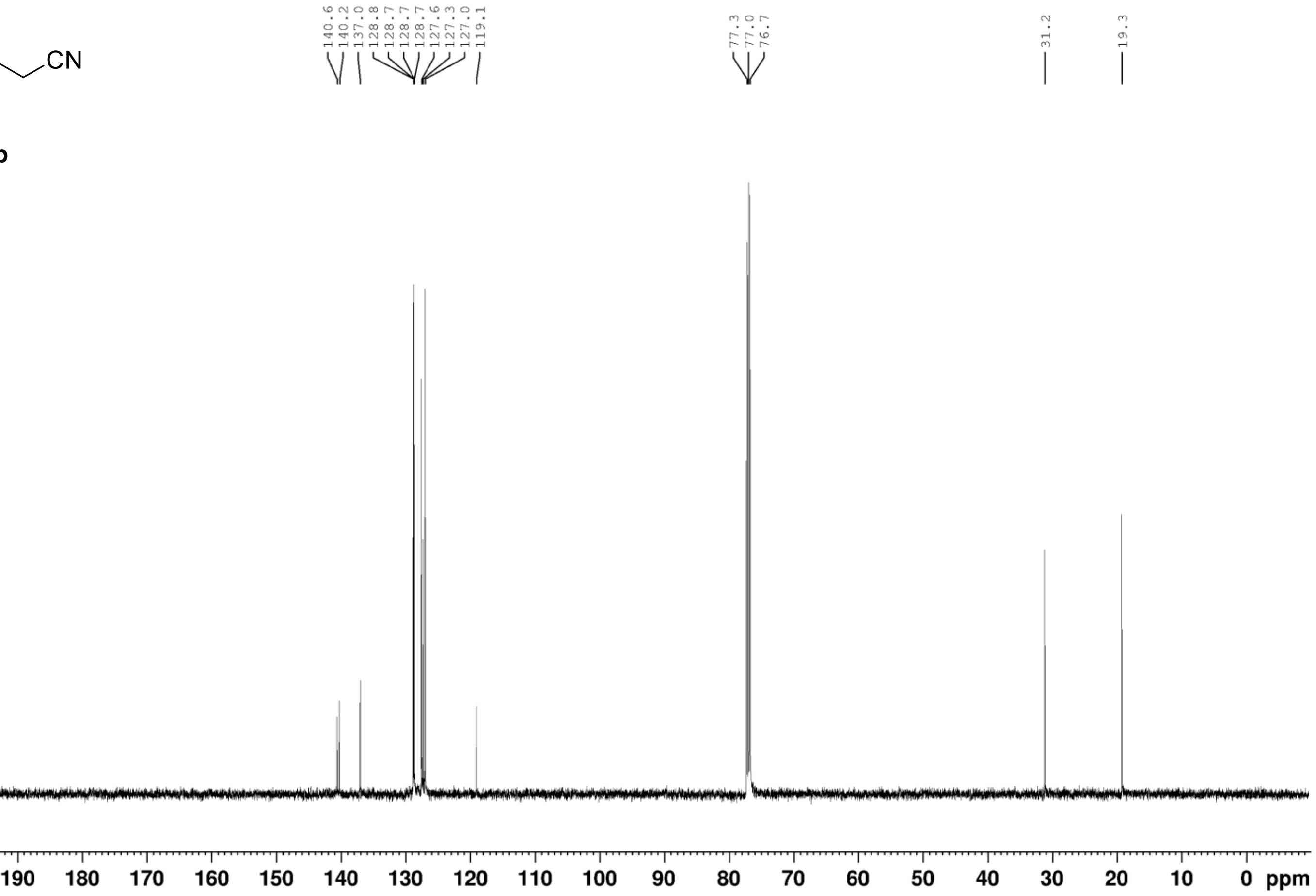

Figure S91. **¹H NMR** (500 MHz, $CDCl_3$) of **2c**.

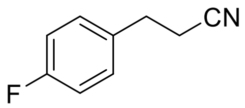

2c

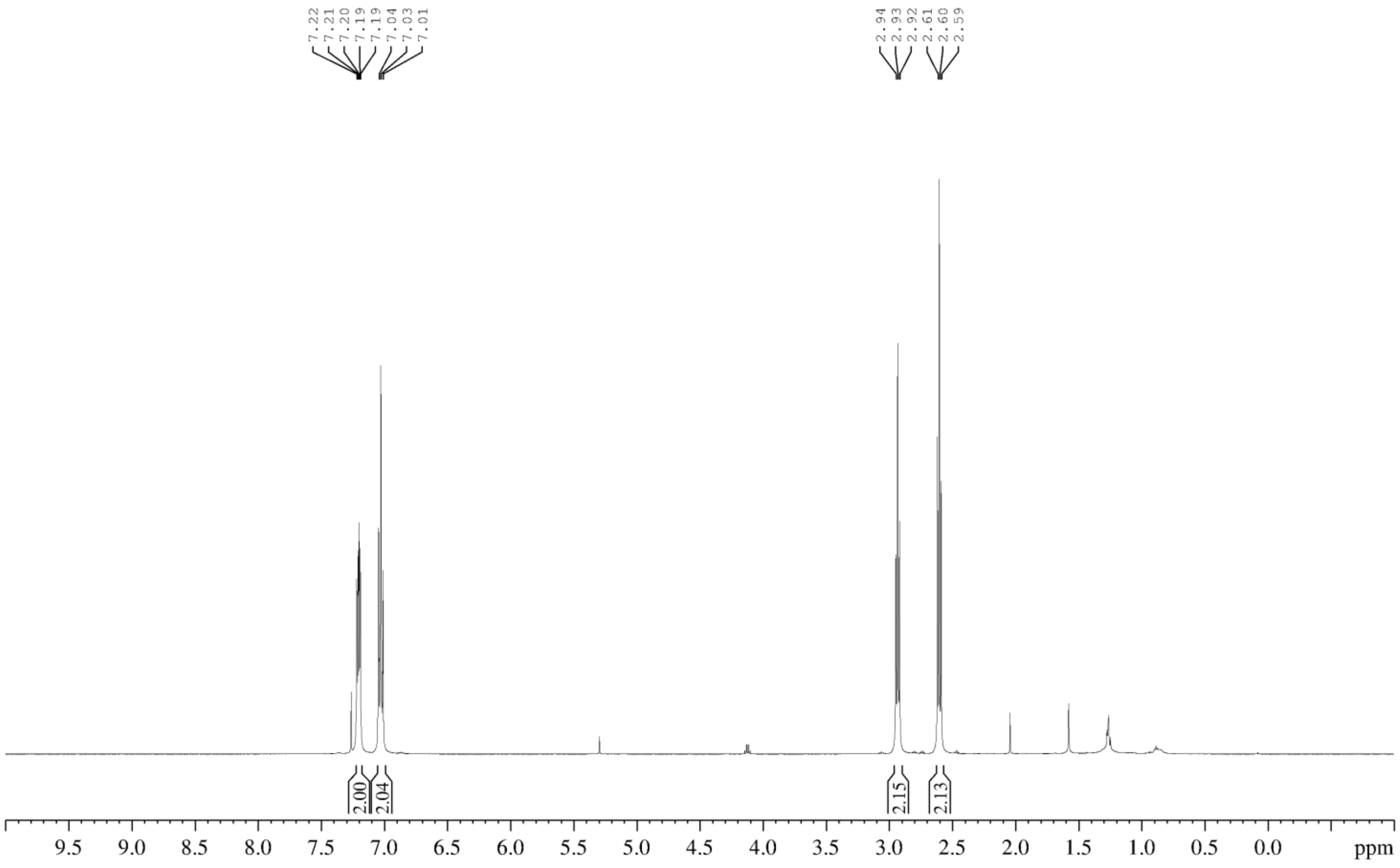

Figure S92. ^{13}**C NMR** (126 MHz, $CDCl_3$) of **2c**.

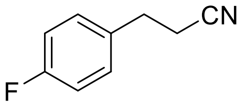

2c

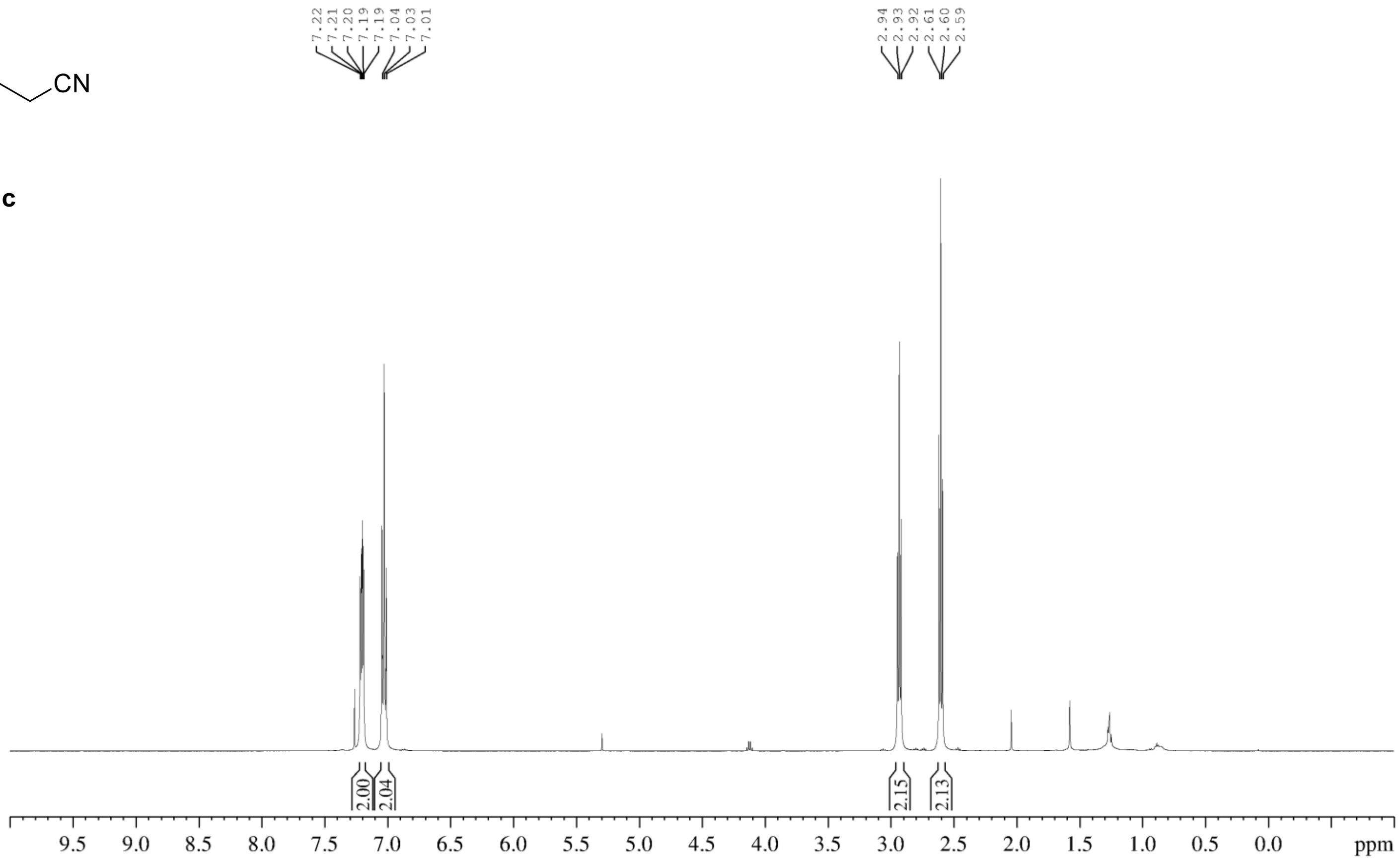

Figure S93. **¹⁹F NMR** (471 MHz, CDCl$_3$) of **2c**.

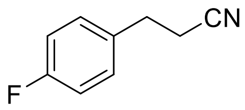

2c

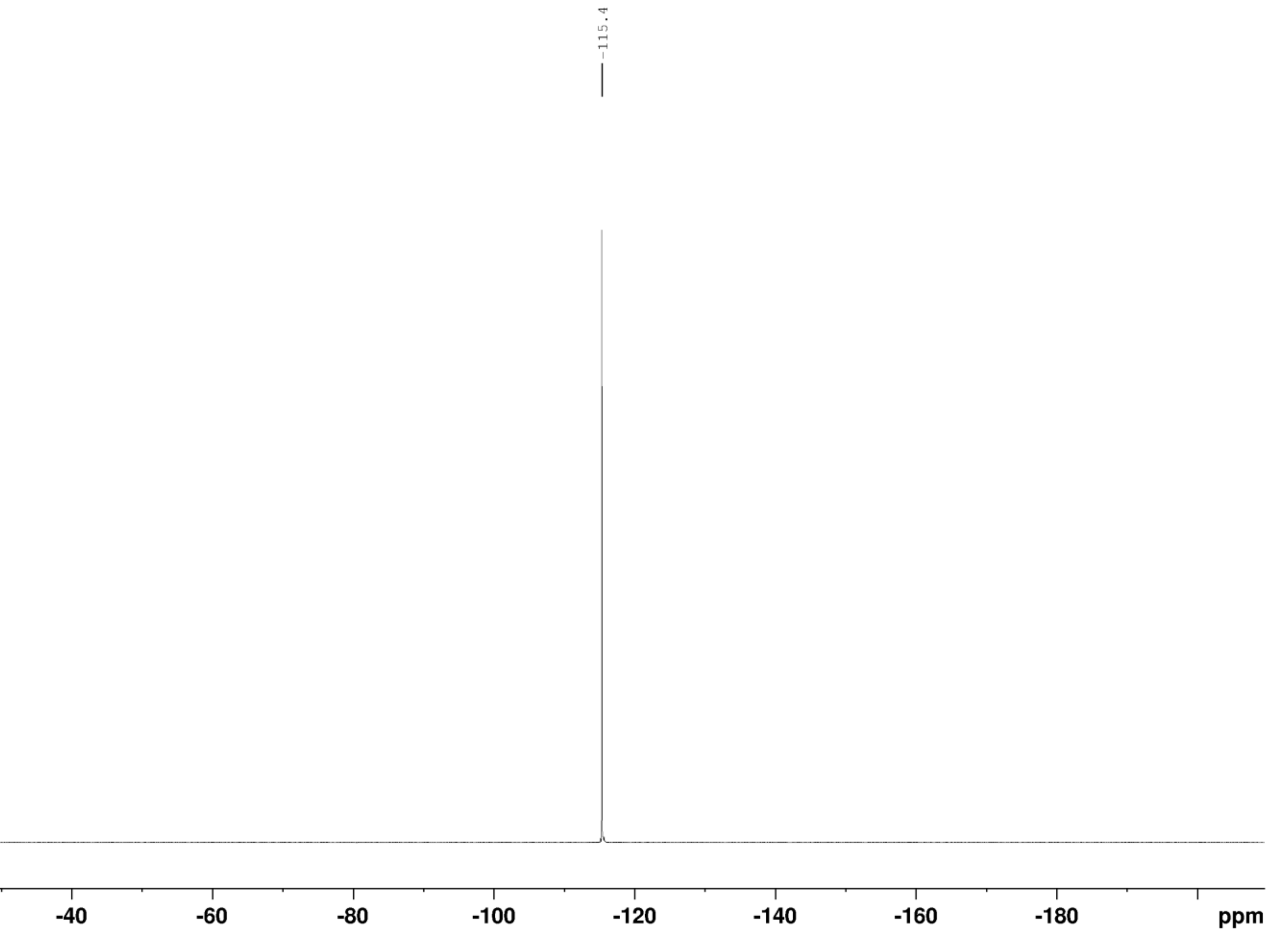

Figure S94. **^{1}H NMR** (500 MHz, $CDCl_3$) of **2d**.

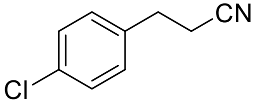

2d

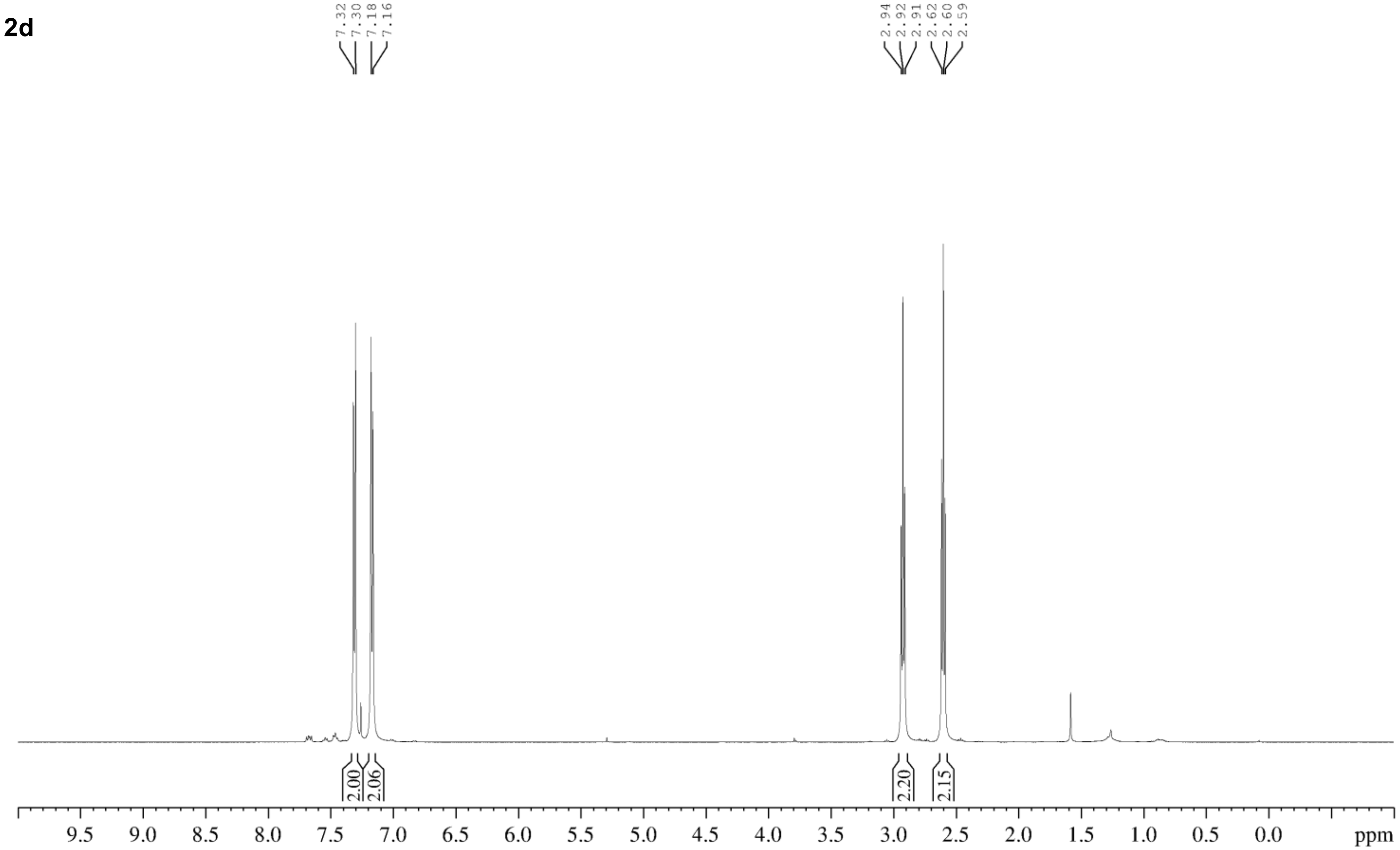

Figure S95. ^{13}**C NMR** (126 MHz, $CDCl_3$) of **2d**.

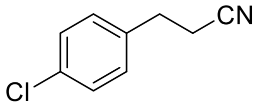

2d

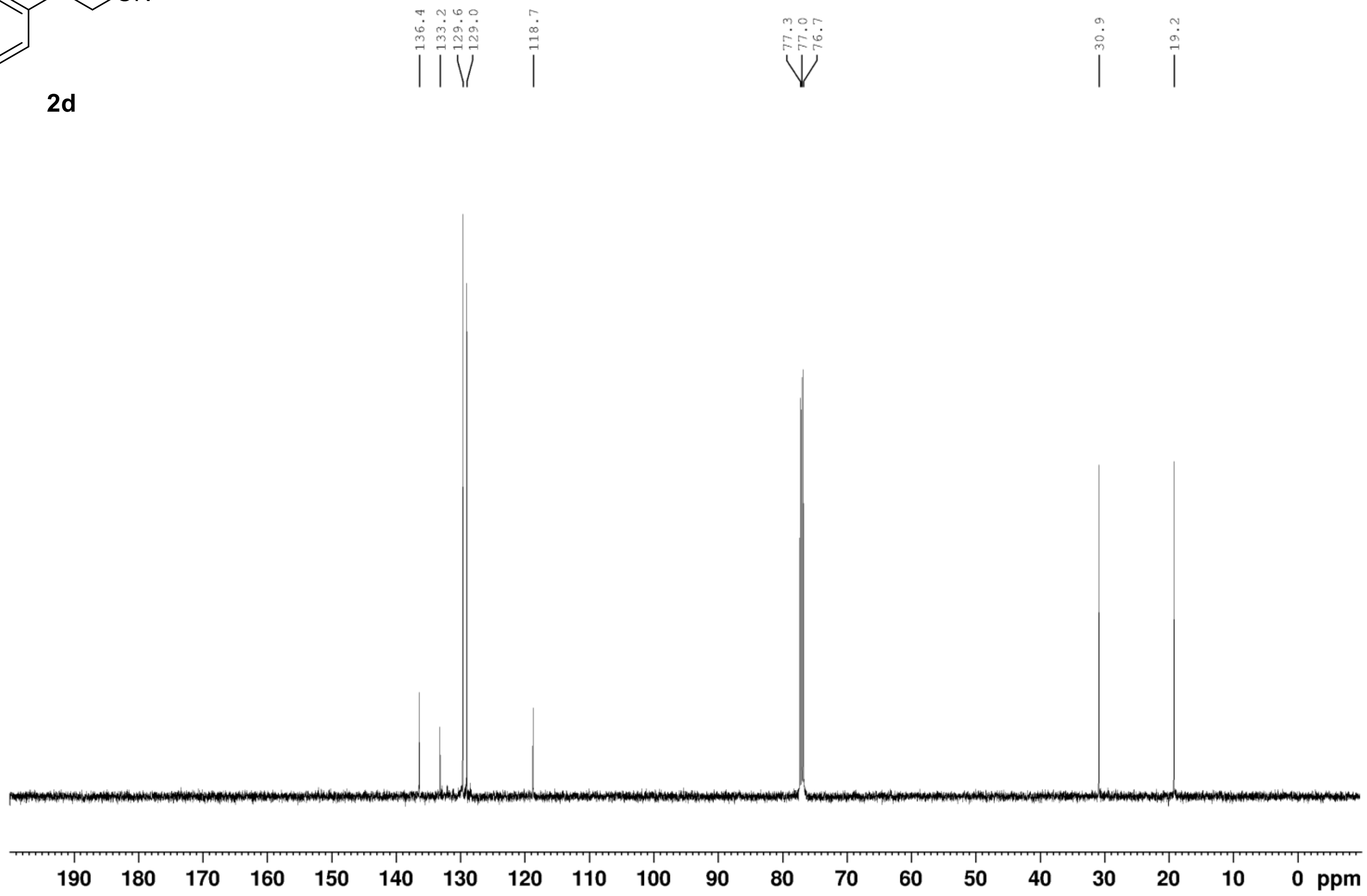

Figure S96. ^{1}**H NMR** (500 MHz, $CDCl_3$) of **2e**.

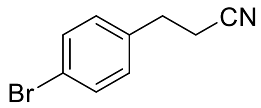

2e

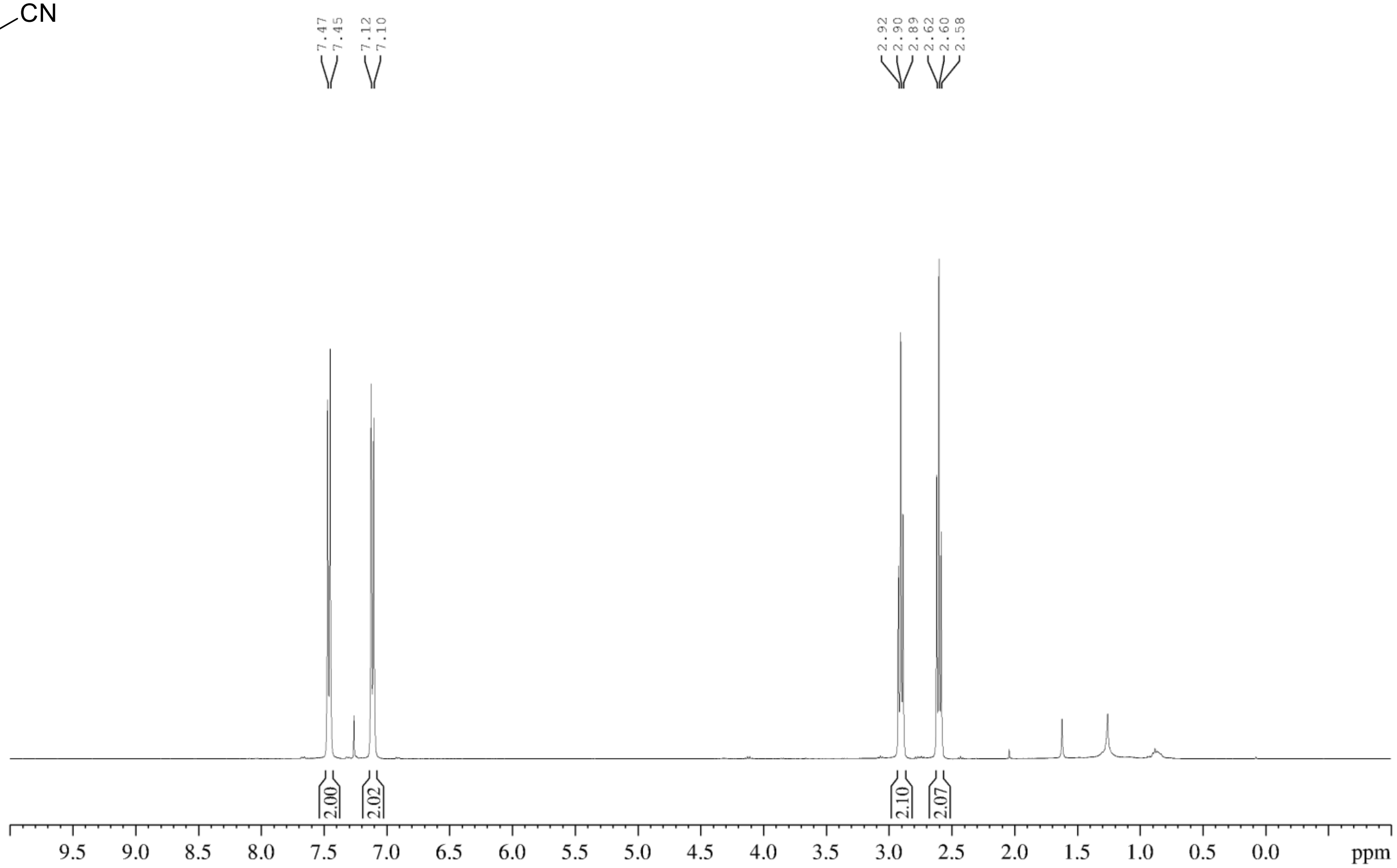

Figure S97. ^{13}**C NMR** (126 MHz, $CDCl_3$) of **2e**.

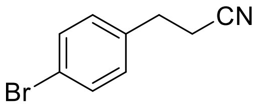

2e

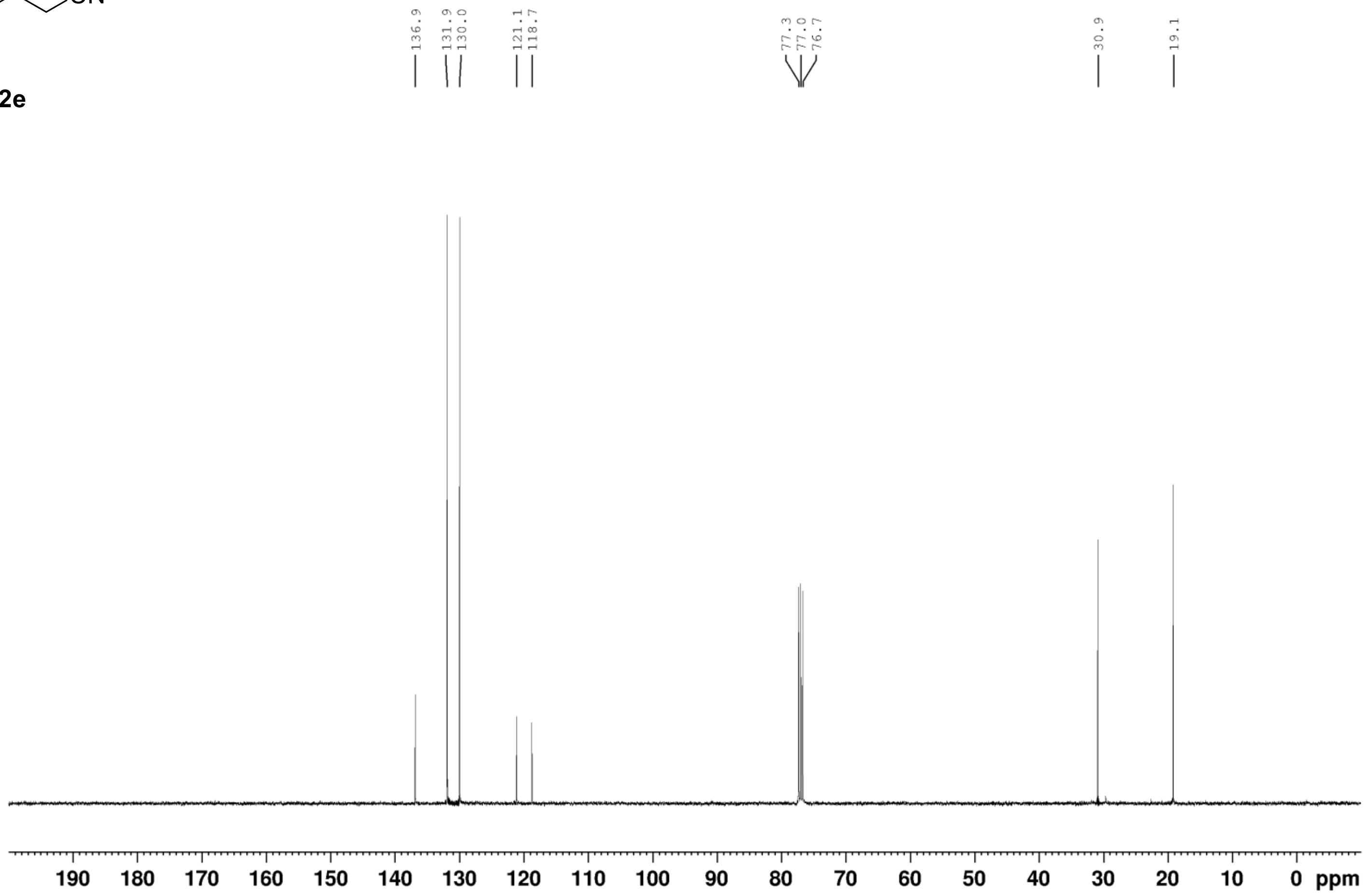

Figure S98. **^{1}H NMR** (500 MHz, $CDCl_3$) of **2f**.

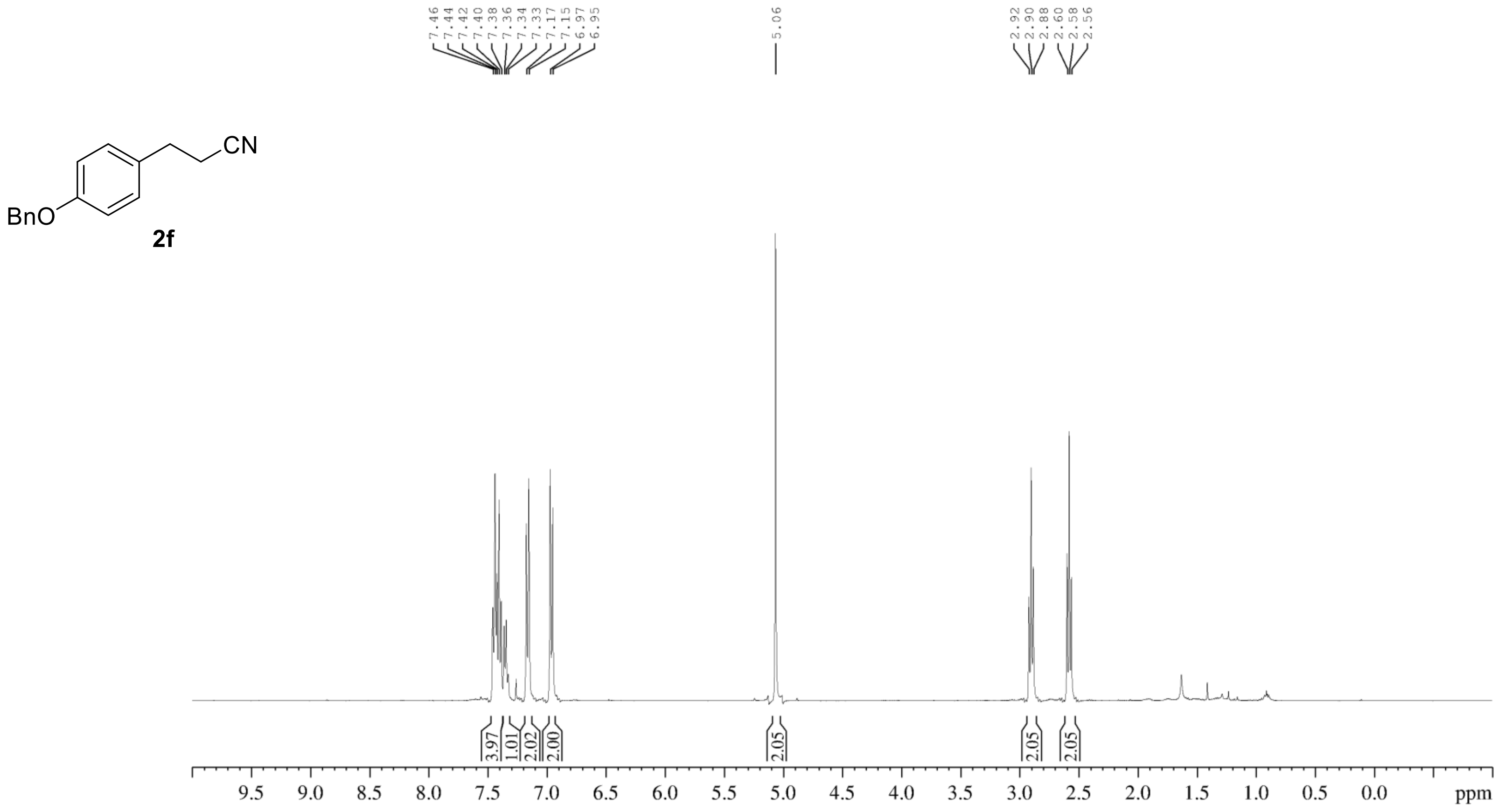

Figure S99. **^{13}C NMR** (126 MHz, $CDCl_3$) of **2f**.

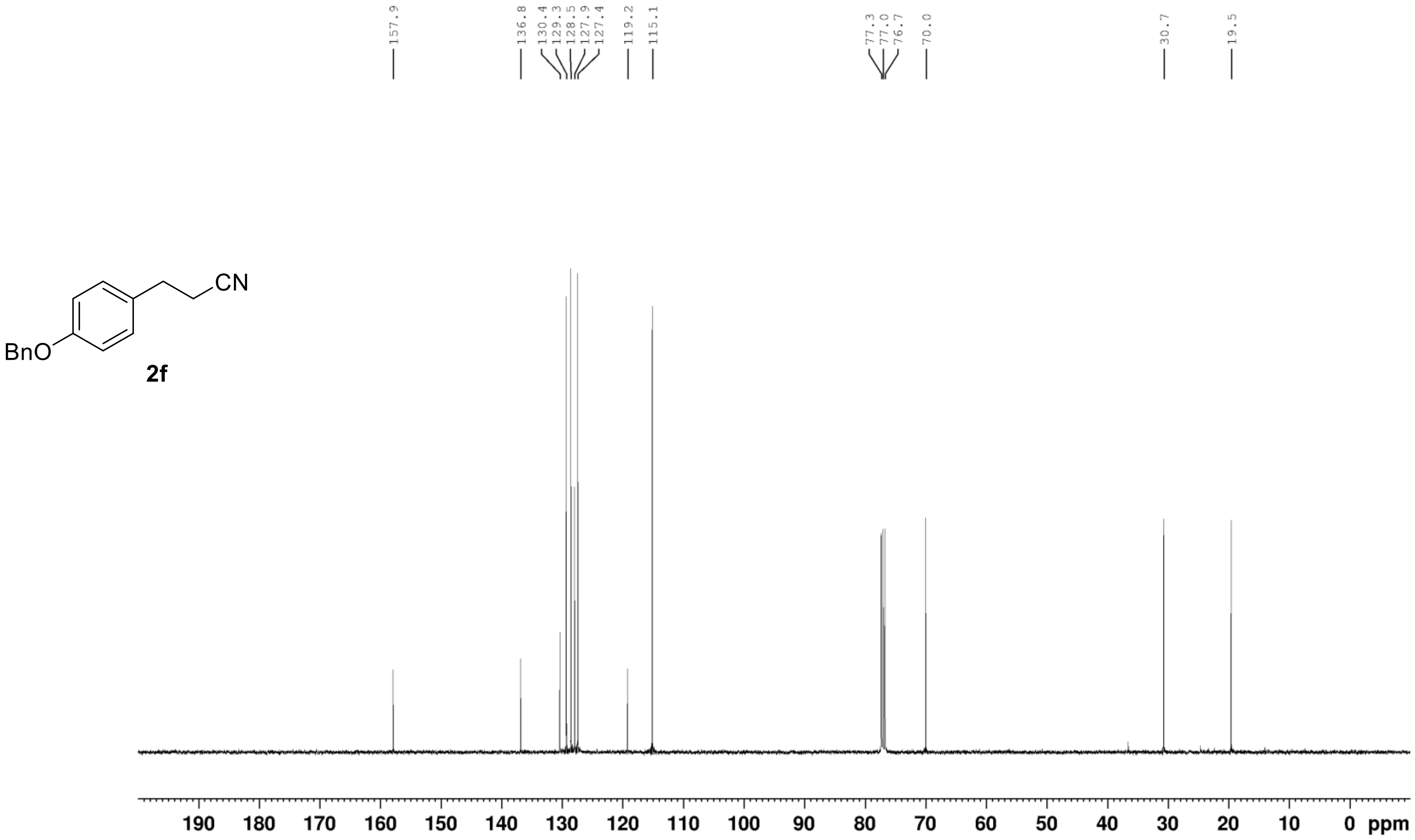

Figure S100. ^{1}H NMR (500 MHz, $CDCl_3$) of **2g**.

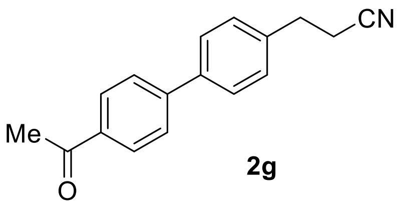

2g

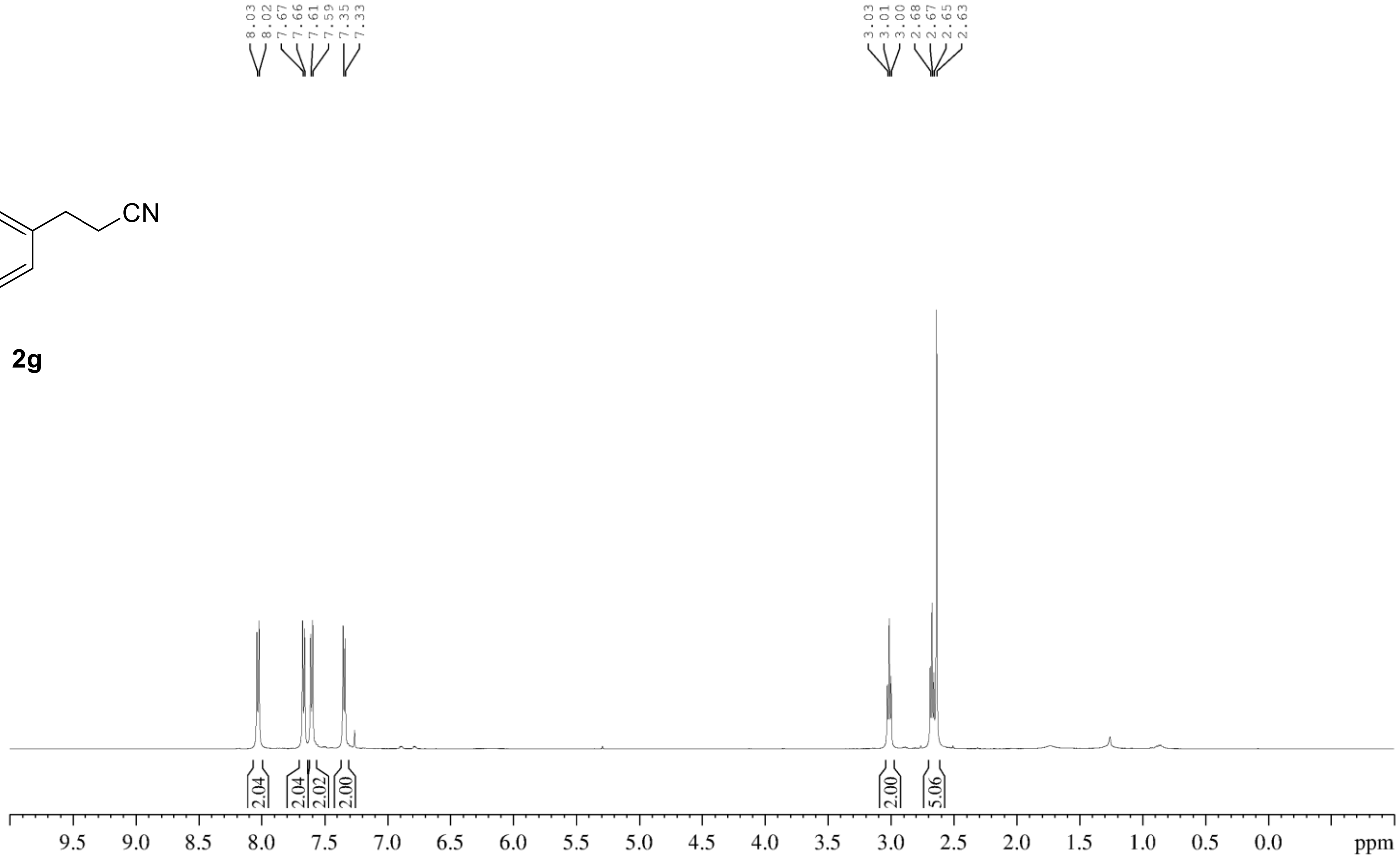

Figure S101 ^{13}**C NMR** (126 MHz, $CDCl_3$) of **2g**.

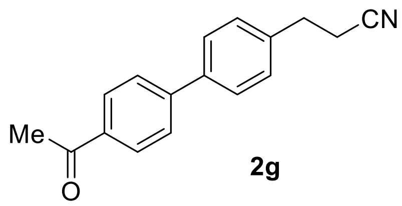

2g

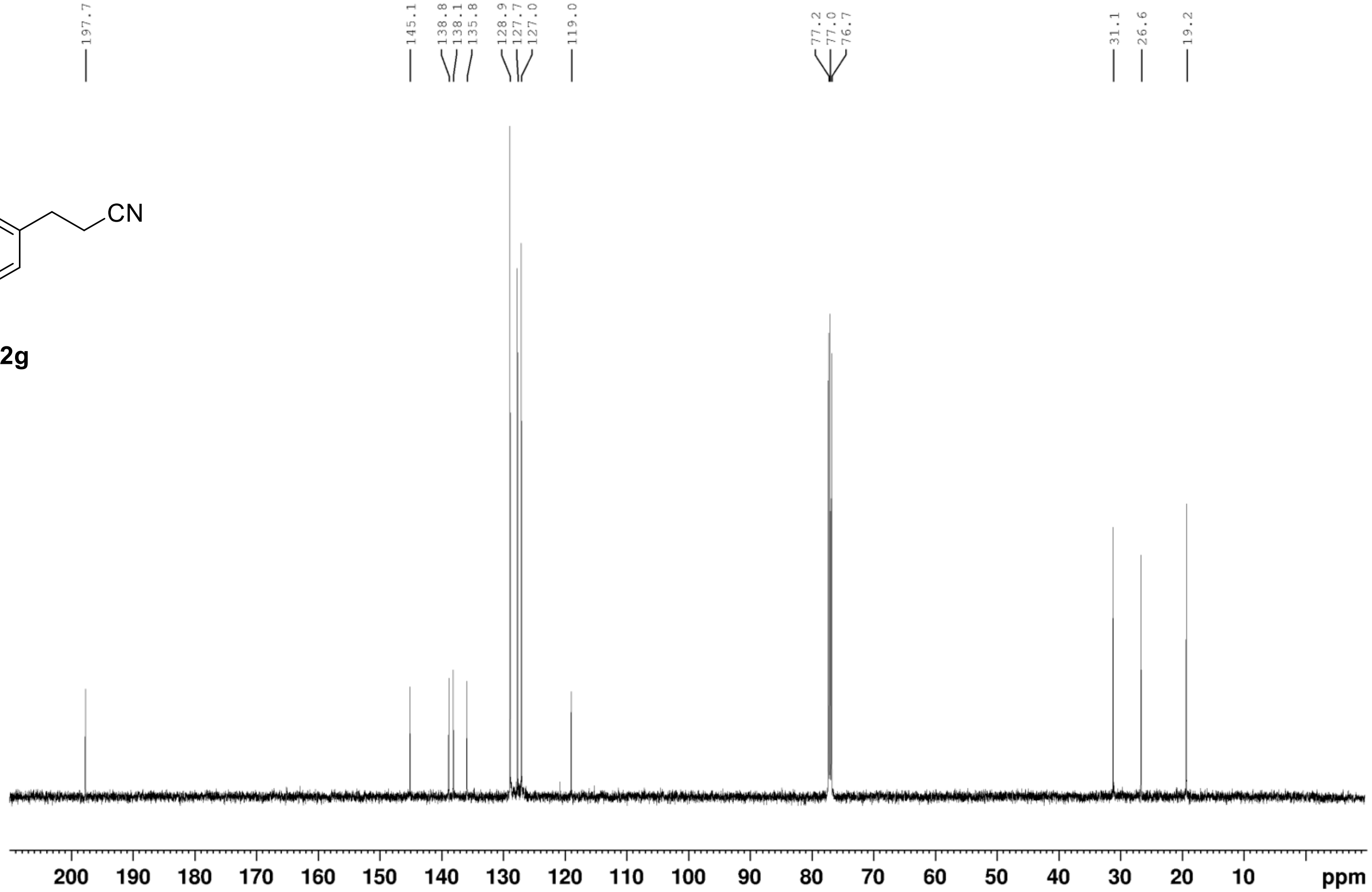

Figure S102. **^{1}H NMR** (500 MHz, $CDCl_3$) of **2i**.

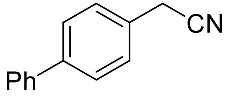

2i

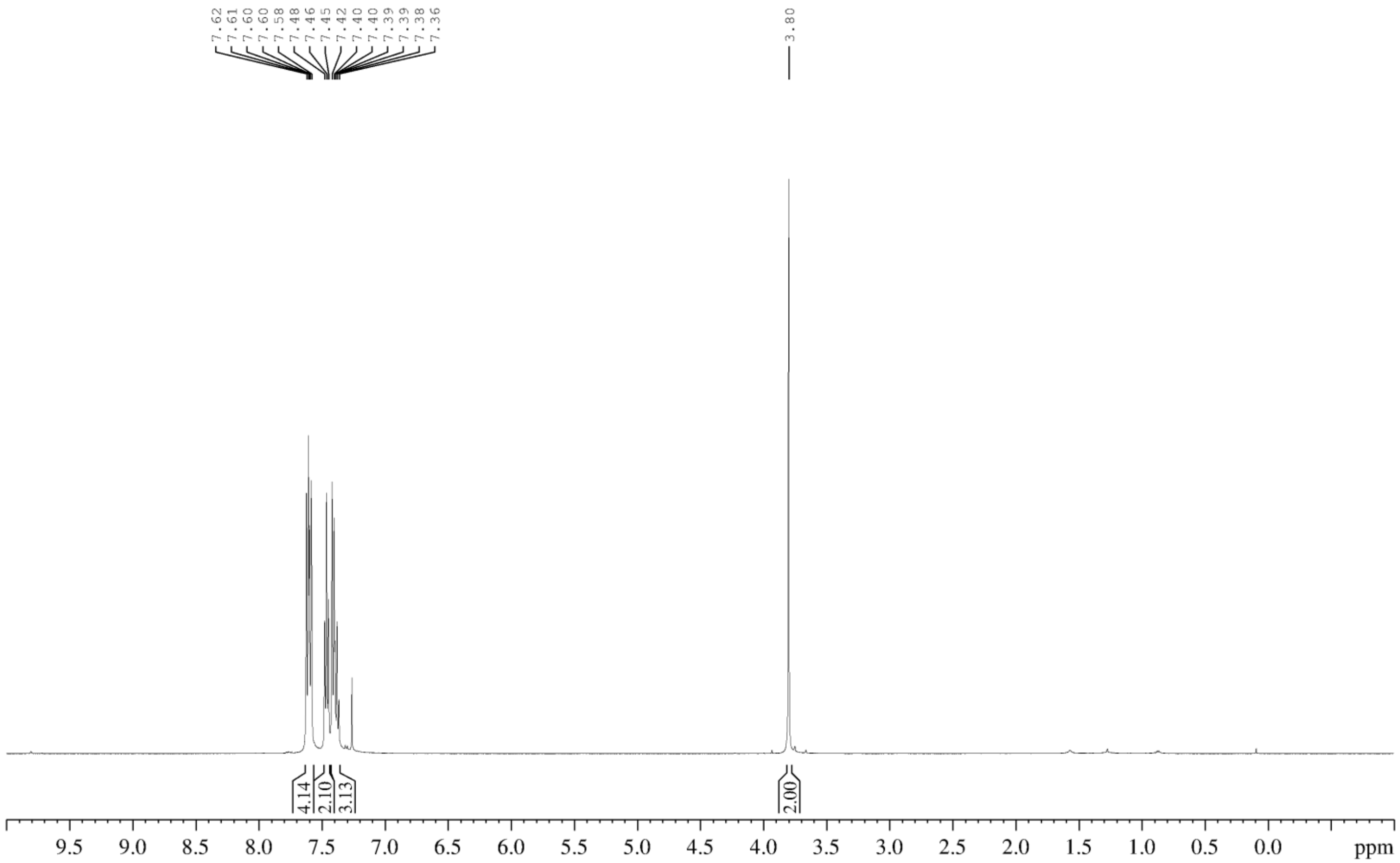

Figure S103. **^{13}C NMR** (126 MHz, $CDCl_3$) of **2i**.

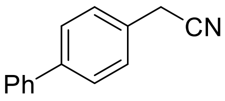

2i

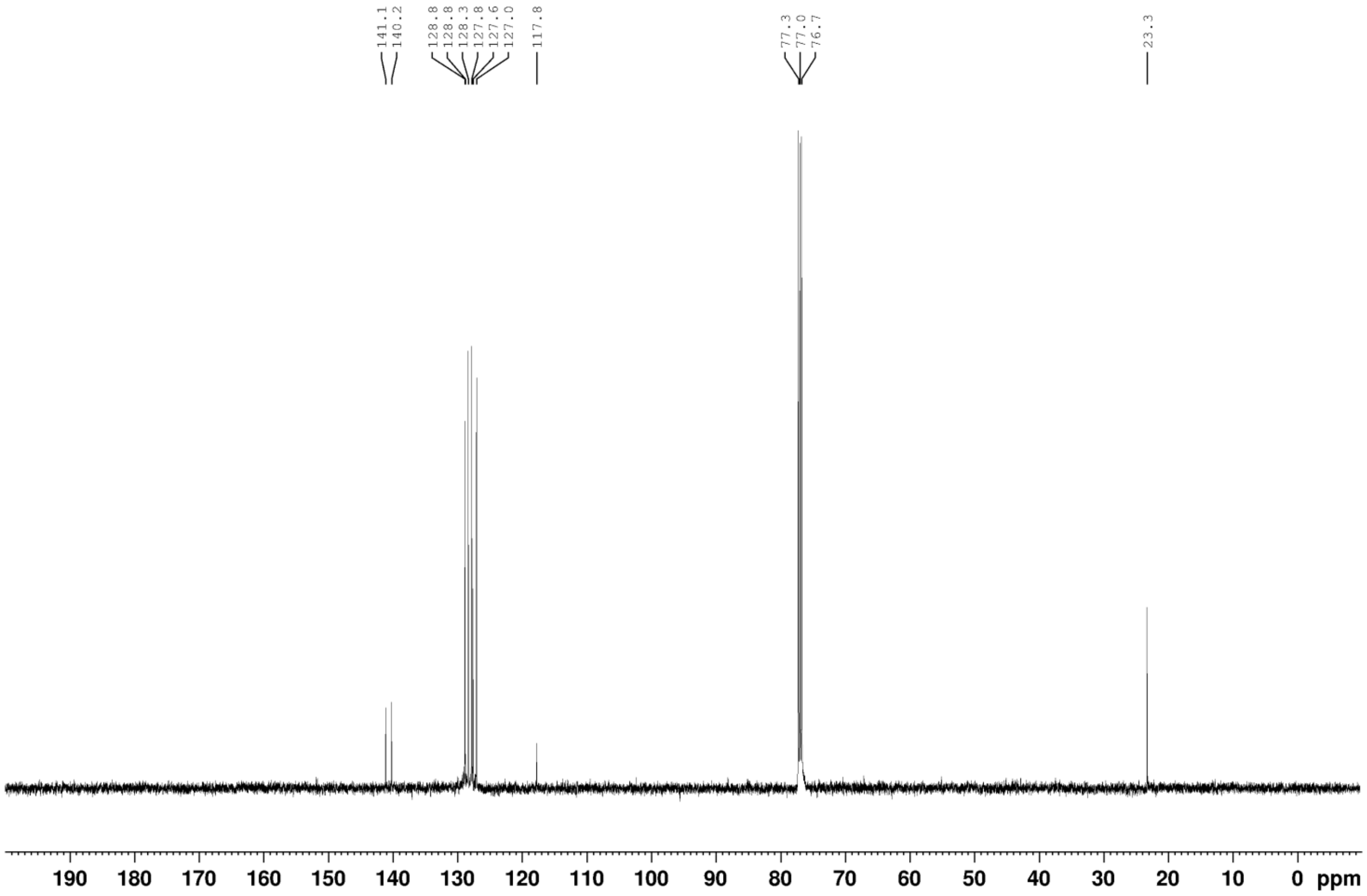

Figure S104. **¹H NMR** (500 MHz, $CDCl_3$) of **2j**.

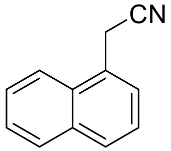

2j

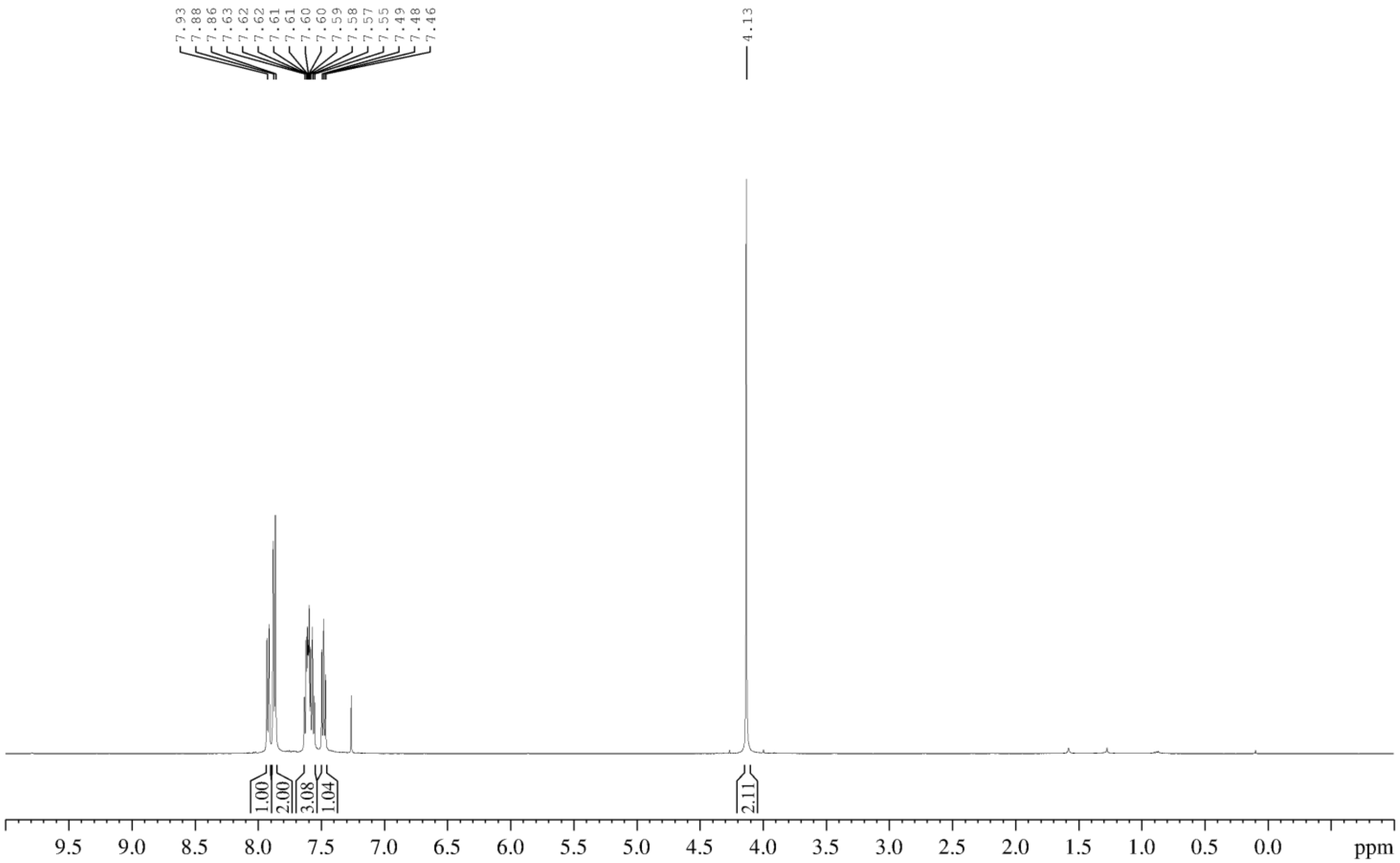

Figure S105. ^{13}**C NMR** (126 MHz, $CDCl_3$) of **2j**.

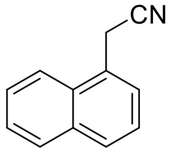

2j

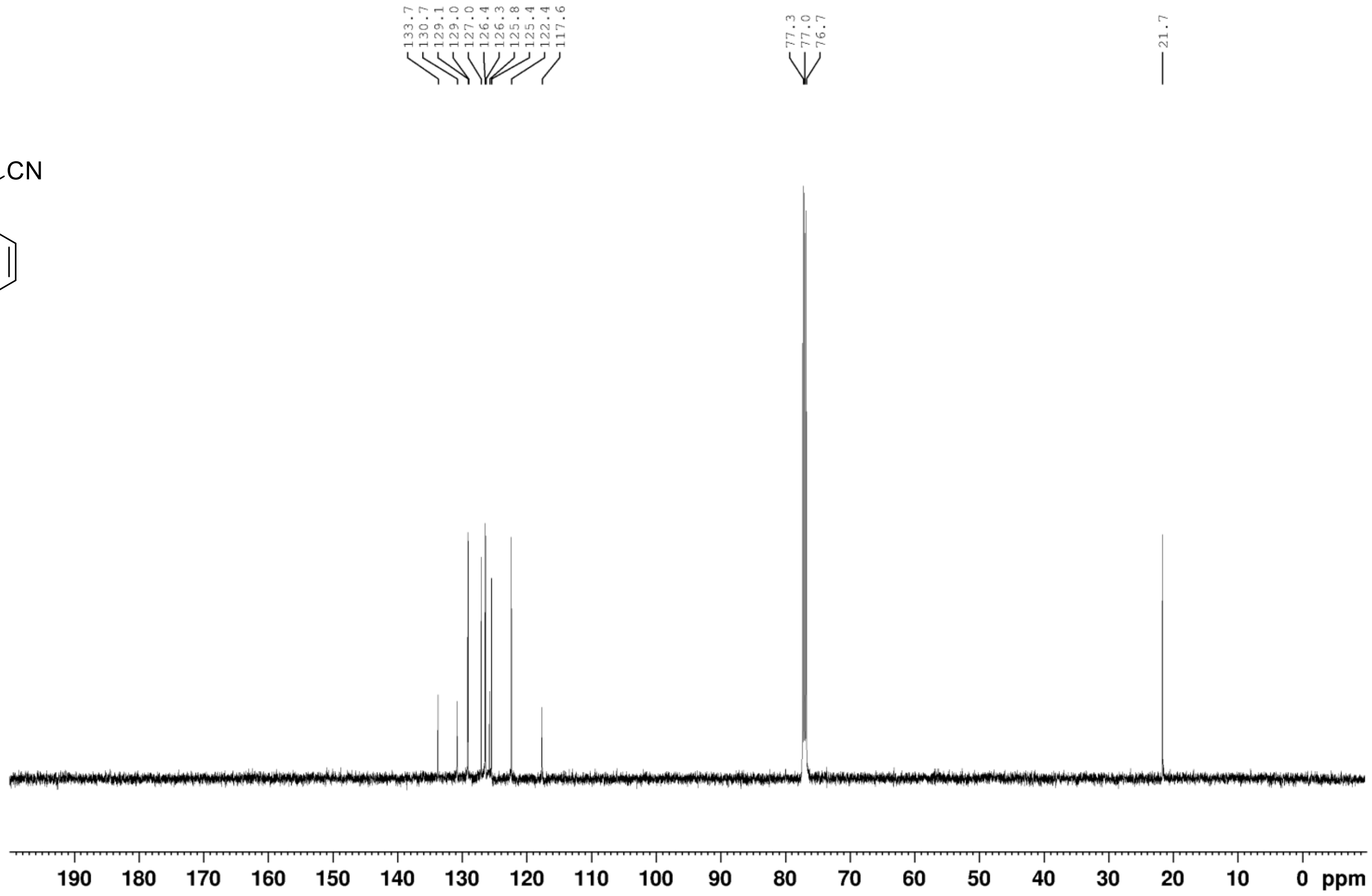

Figure S106. **^{1}H NMR** (500 MHz, $CDCl_3$) of **2k**.

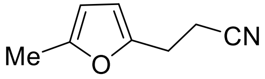

2k

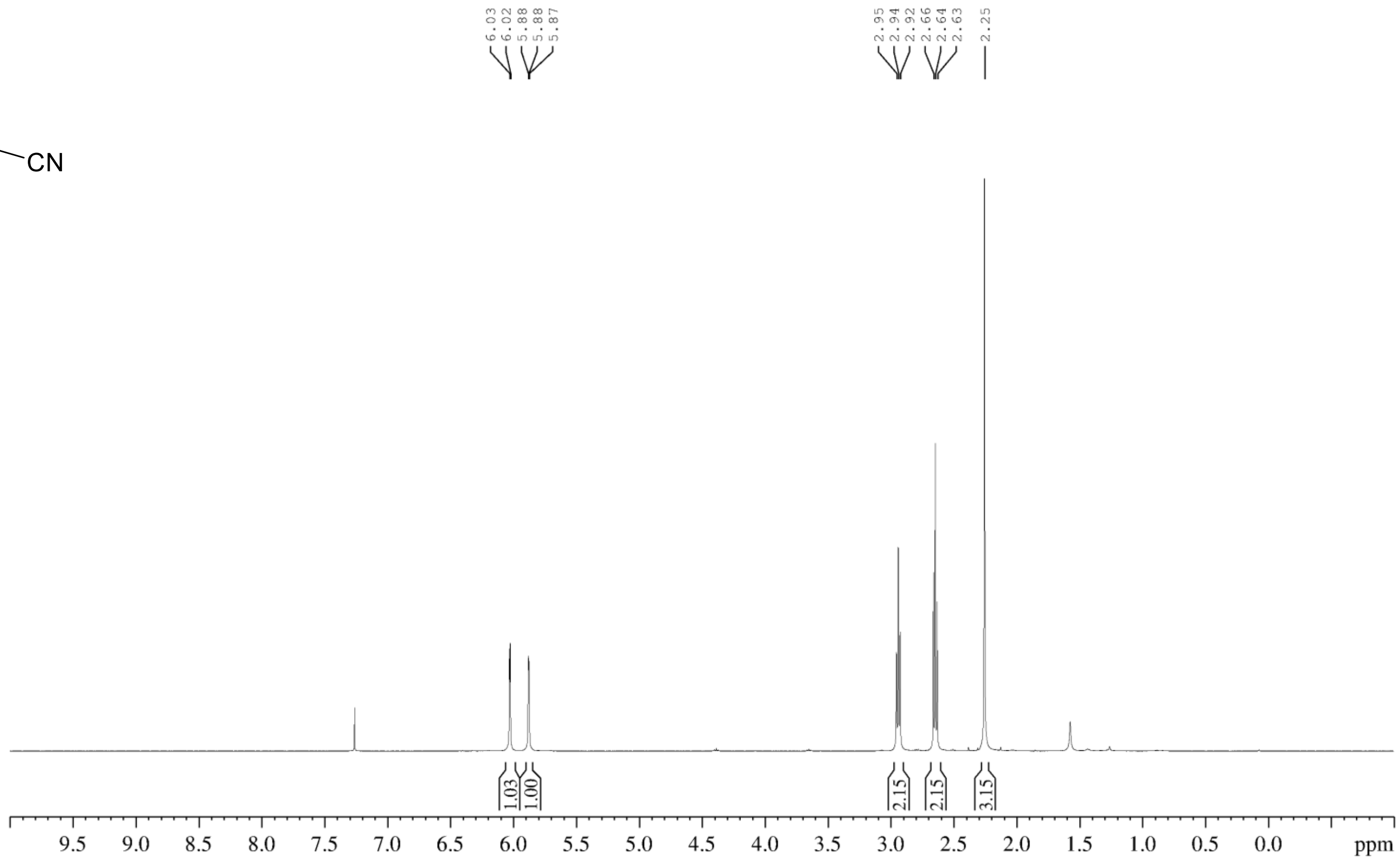

Figure S107. **^{13}C NMR** (126 MHz, $CDCl_3$) of **2k**.

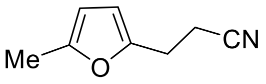

2k

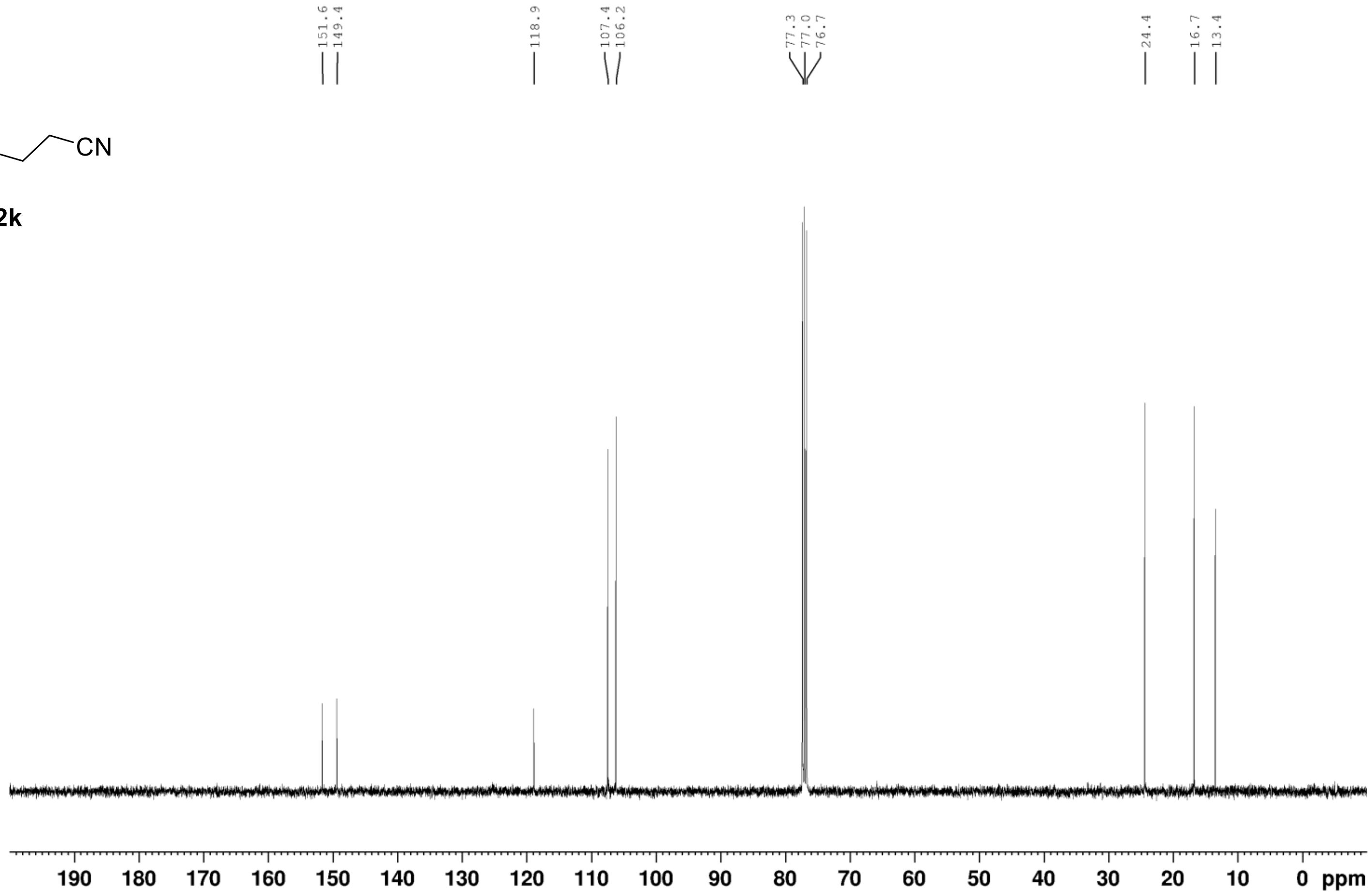

Figure S108. **¹H NMR** (500 MHz, $CDCl_3$) of **2l**.

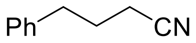

2l

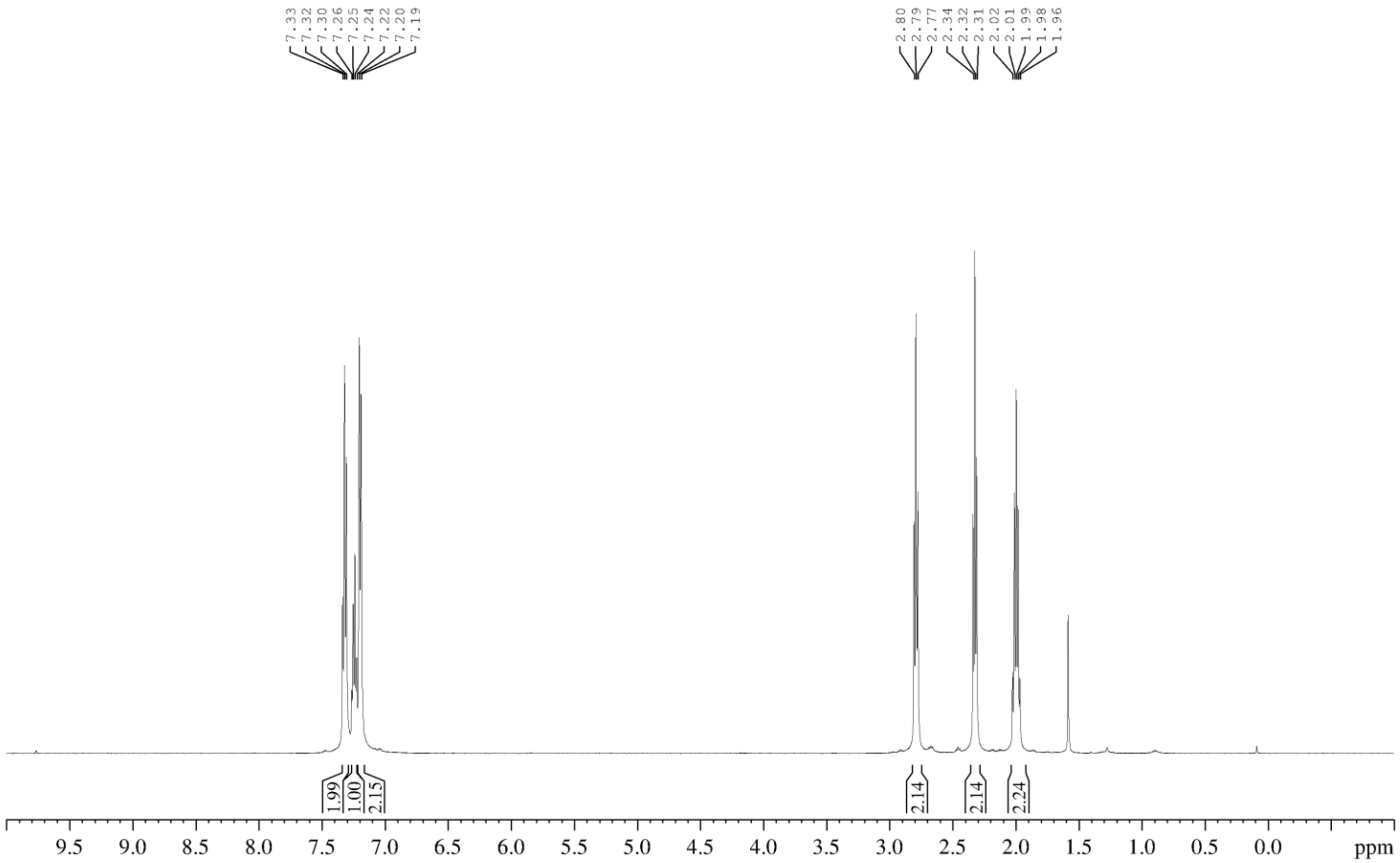

Figure S109. **^{13}C NMR** (126 MHz, $CDCl_3$) of **2l**.

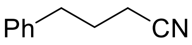

2l

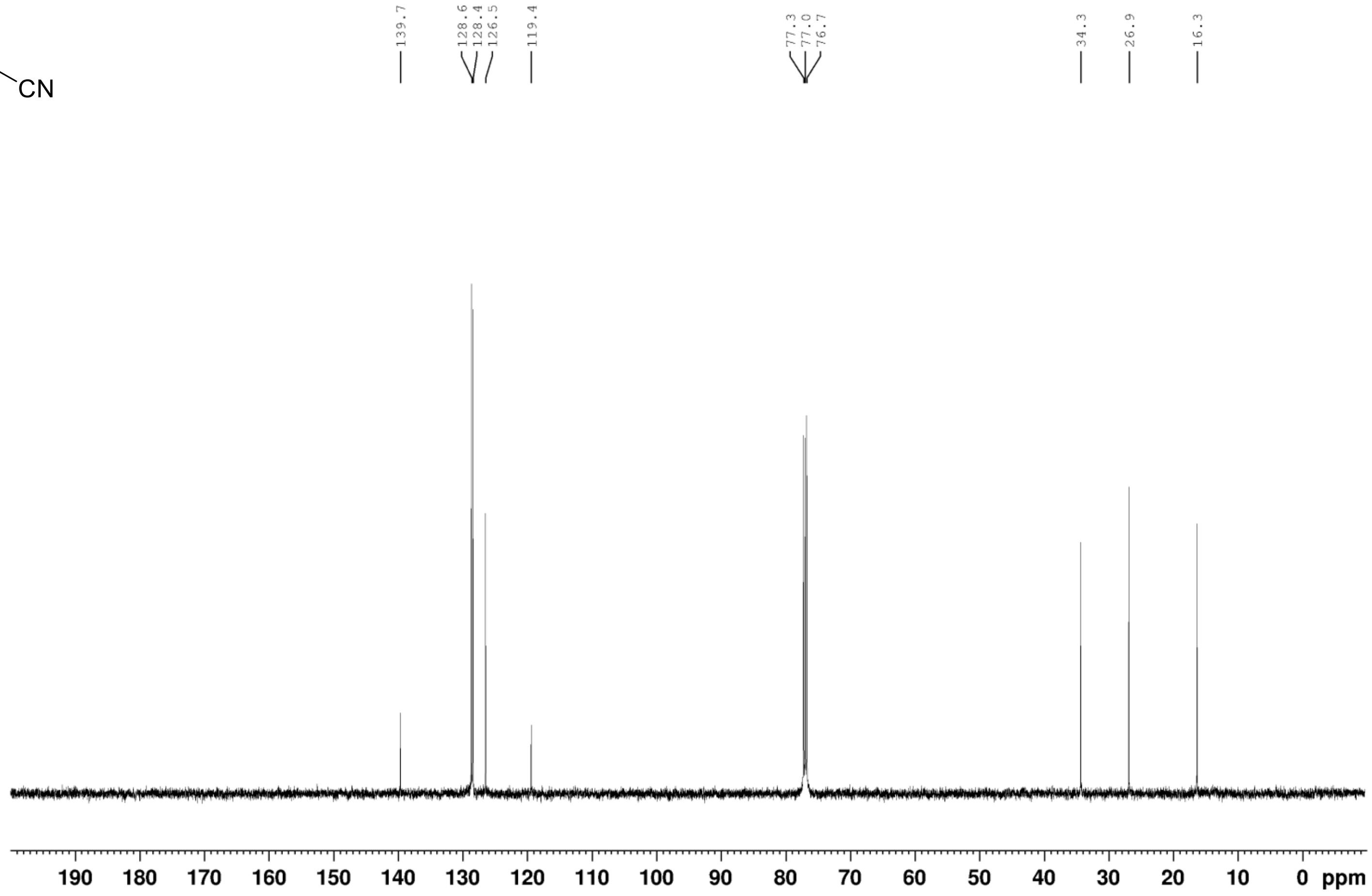

Figure S110. **^{1}H NMR** (500 MHz, $CDCl_3$) of **2m**.

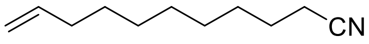

2m

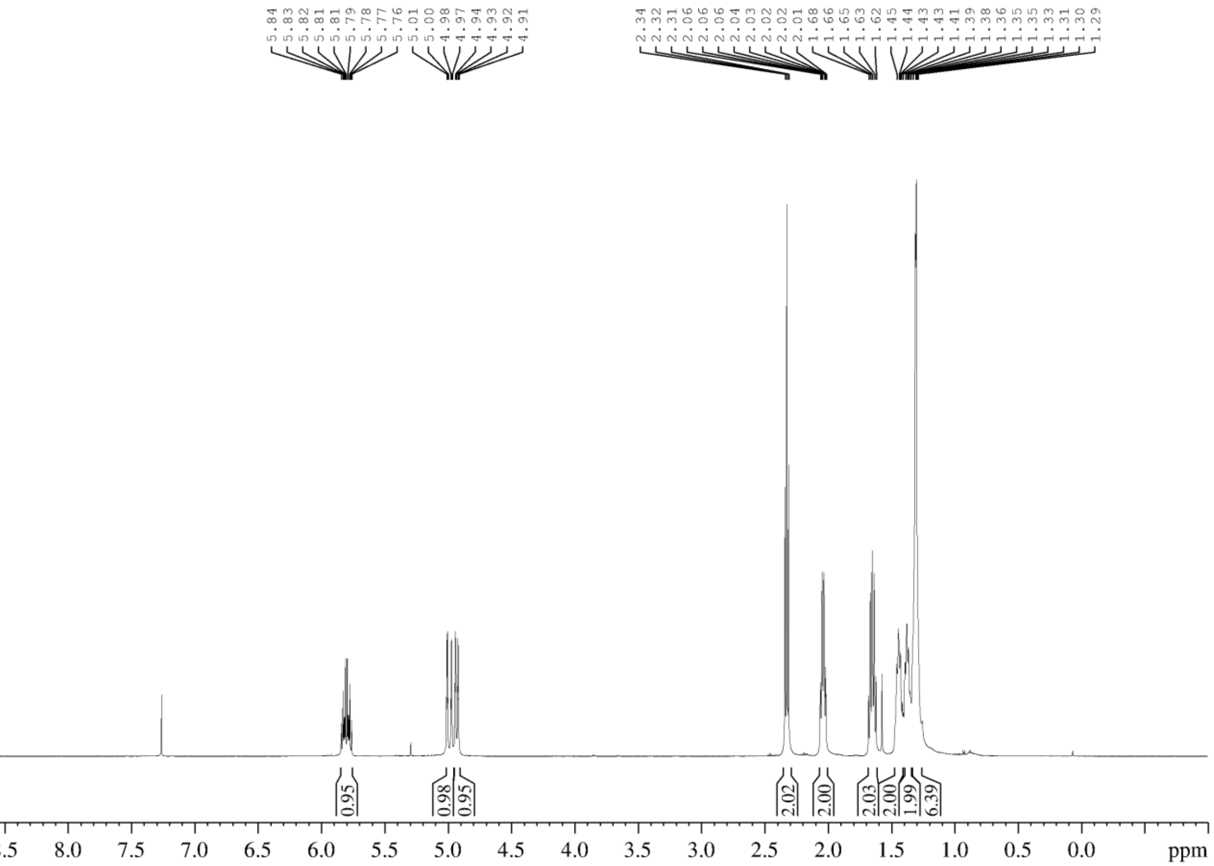

Figure S111. **^{13}C NMR** (126 MHz, $CDCl_3$) of **2m**.

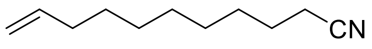

2m

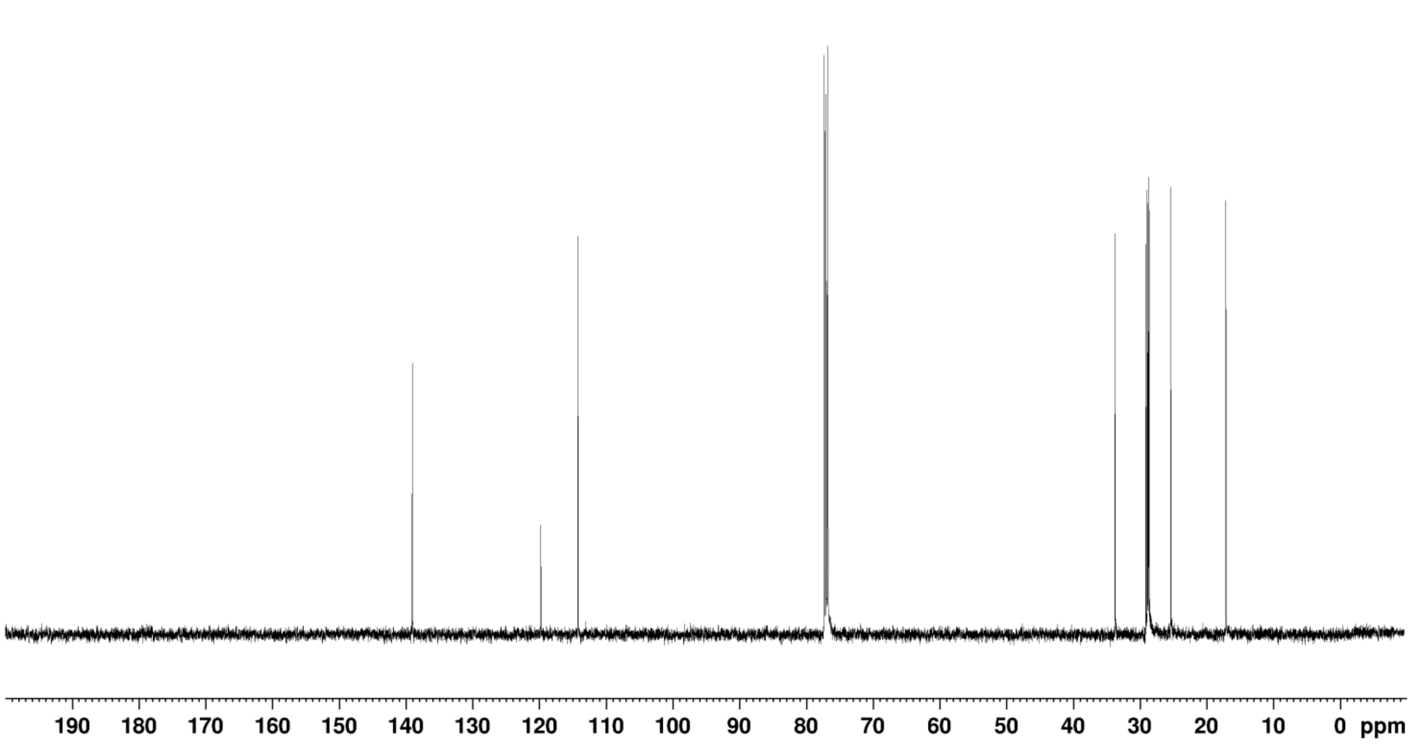

Figure S112. **¹H NMR** (500 MHz, $CDCl_3$) of **2n**.

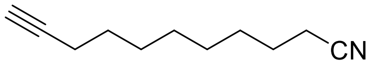

2n

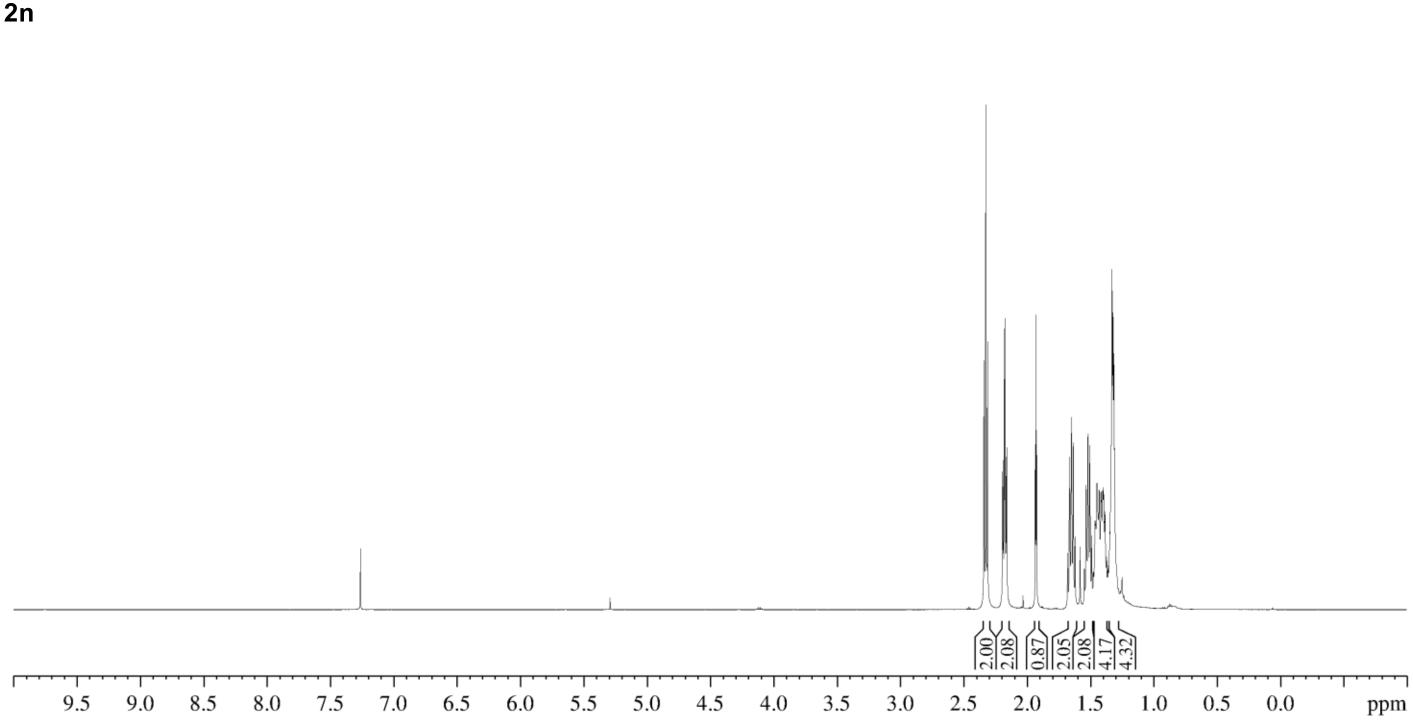

Figure S113. **^{13}C NMR** (126 MHz, $CDCl_3$) of **2n**.

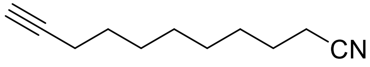

2n

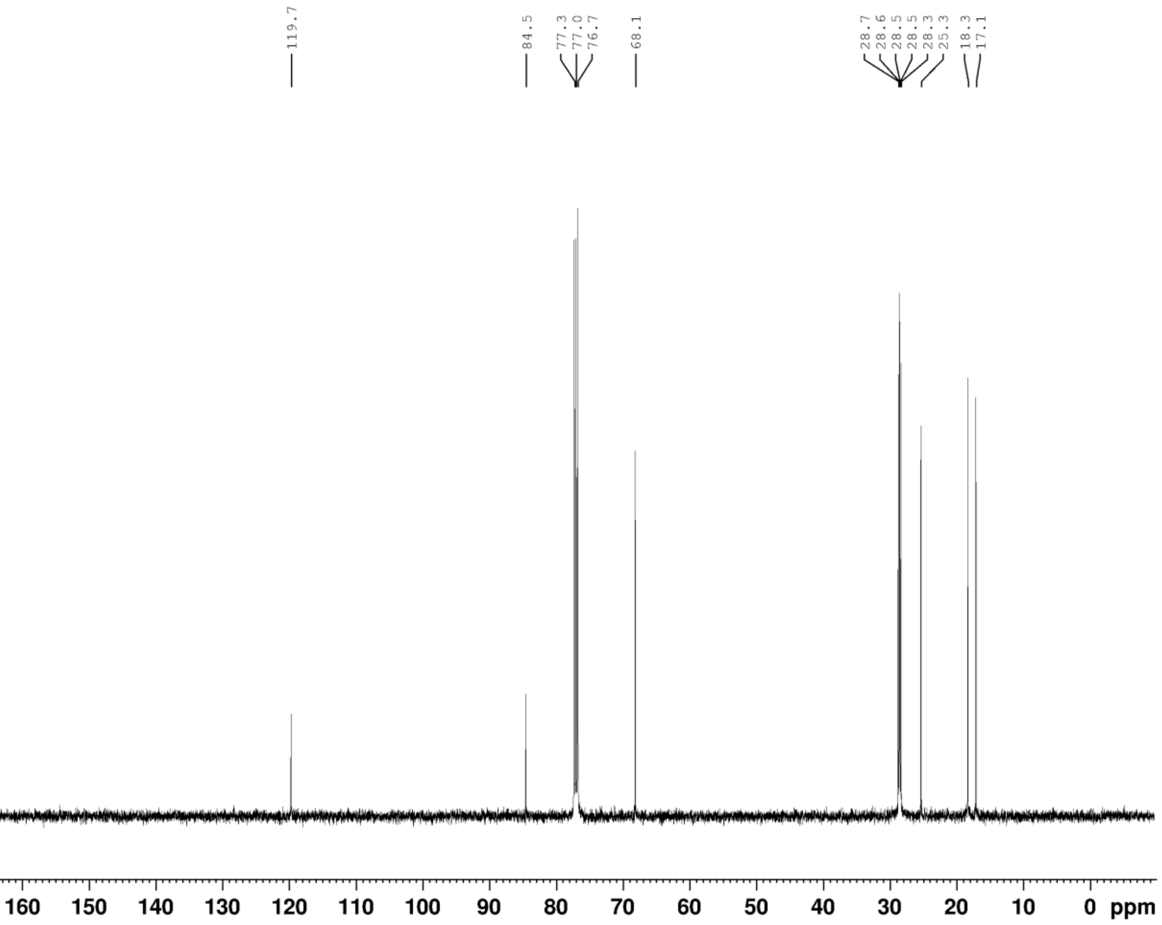

Figure S114. **¹H NMR** (500 MHz, $CDCl_3$) of **2o**.

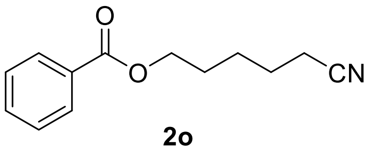

2o

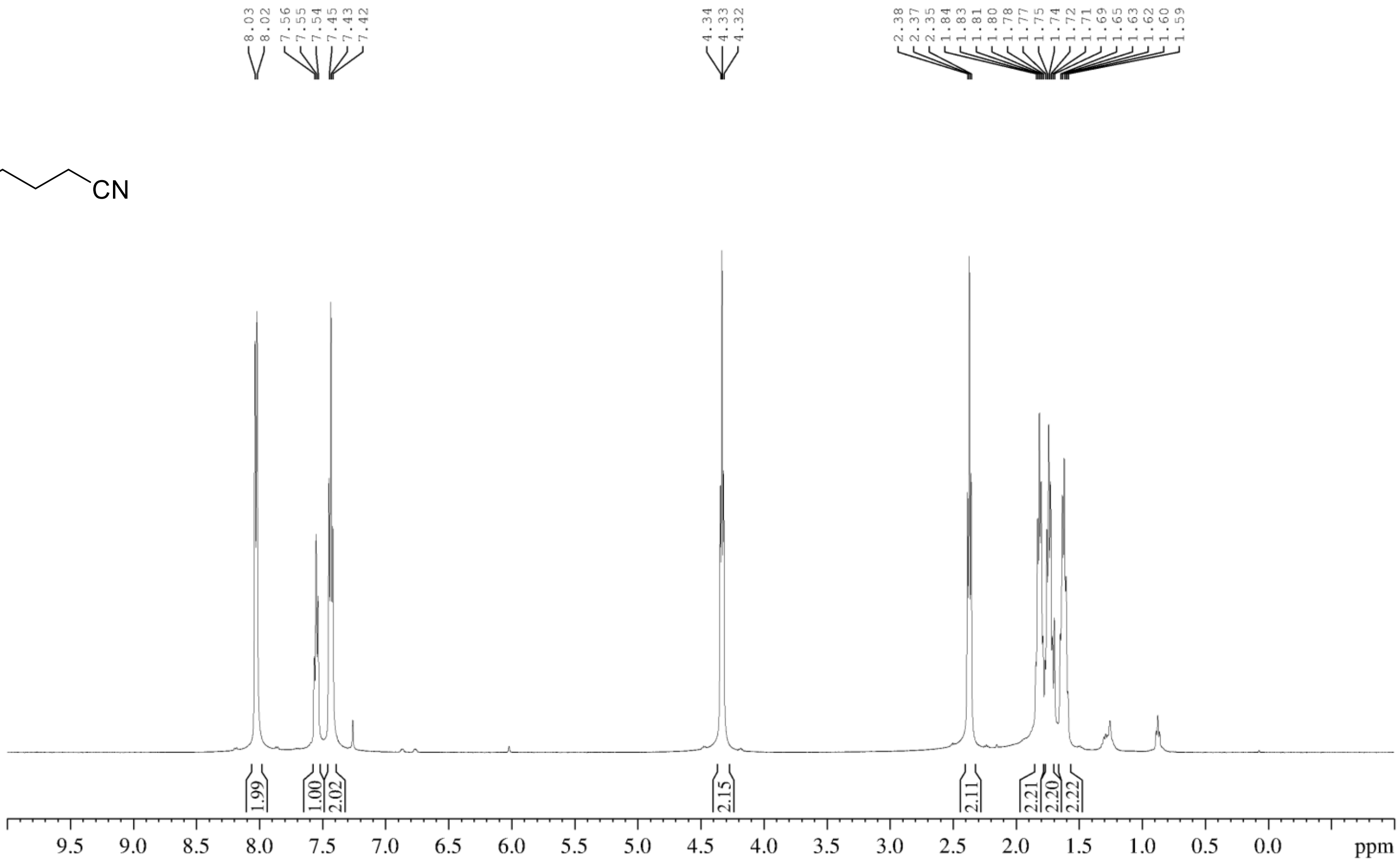

Figure S115. ^{13}**C NMR** (126 MHz, $CDCl_3$) of **2o**.

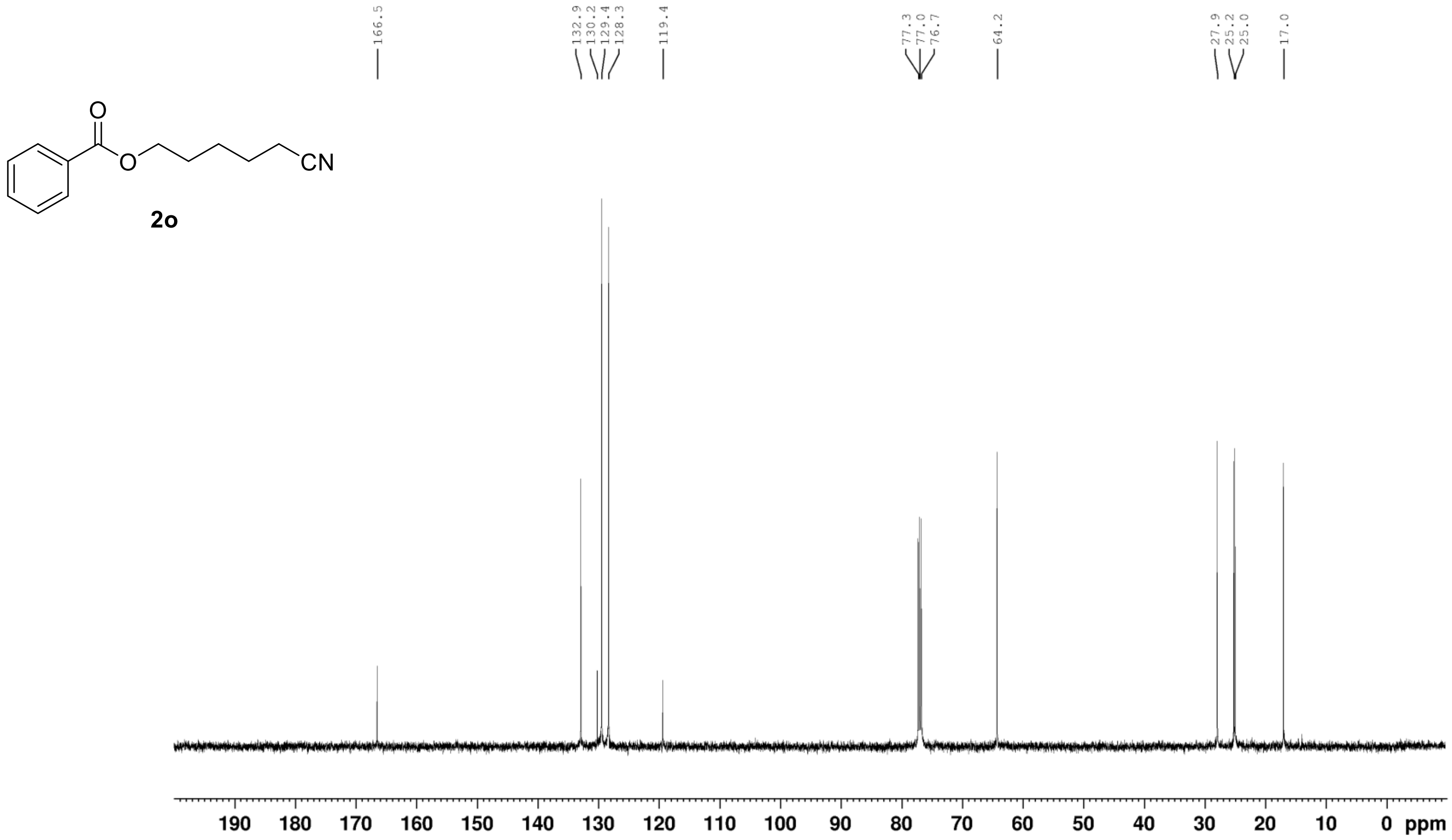

Figure S116. **^{1}H NMR** (500 MHz, $CDCl_3$) of **2p**.

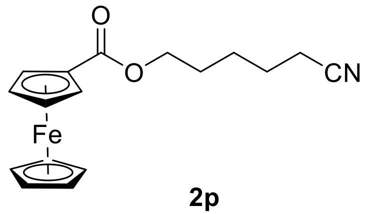

2p

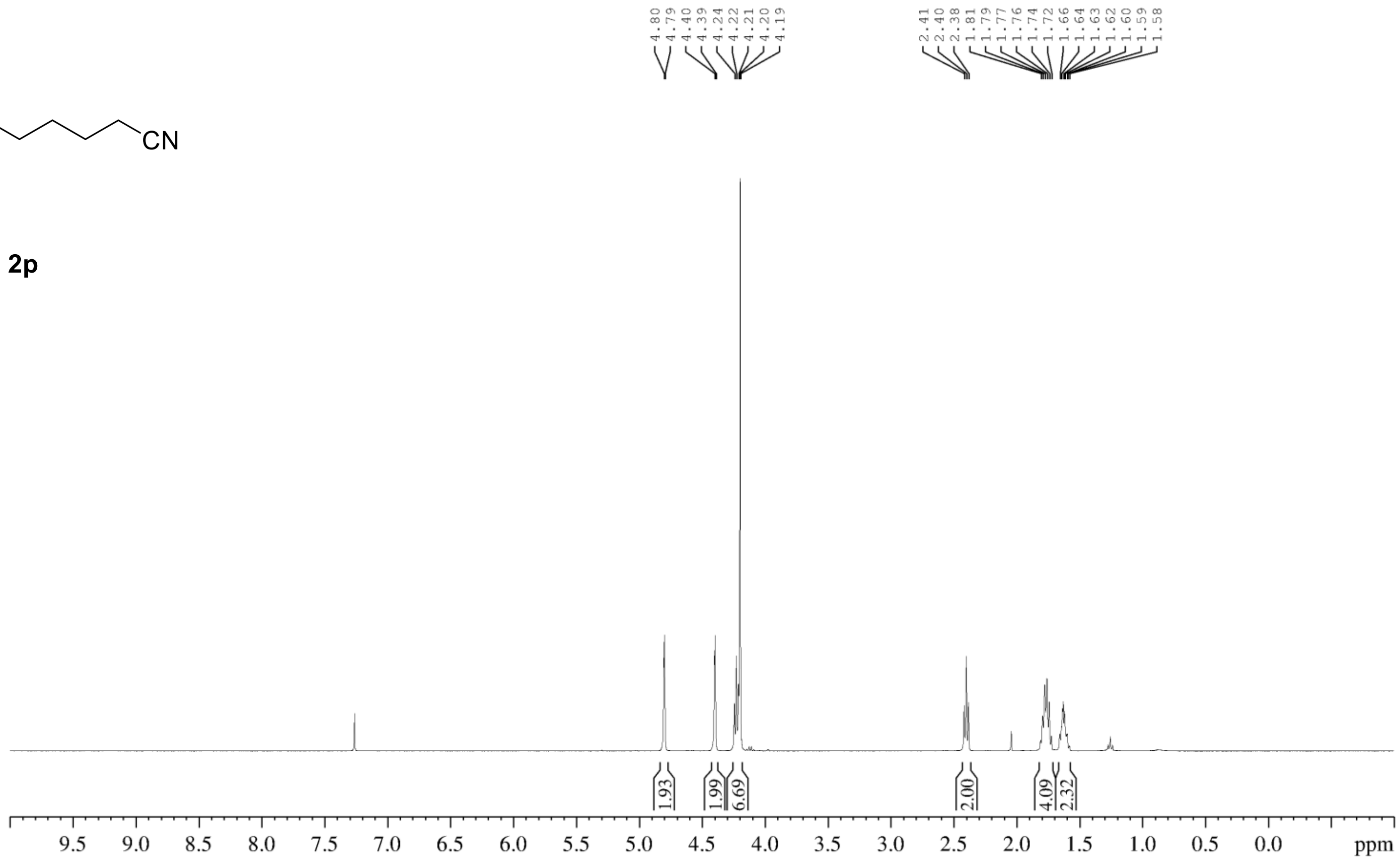

Figure S117. **^{13}C NMR** (126 MHz, $CDCl_3$) of **2p**.

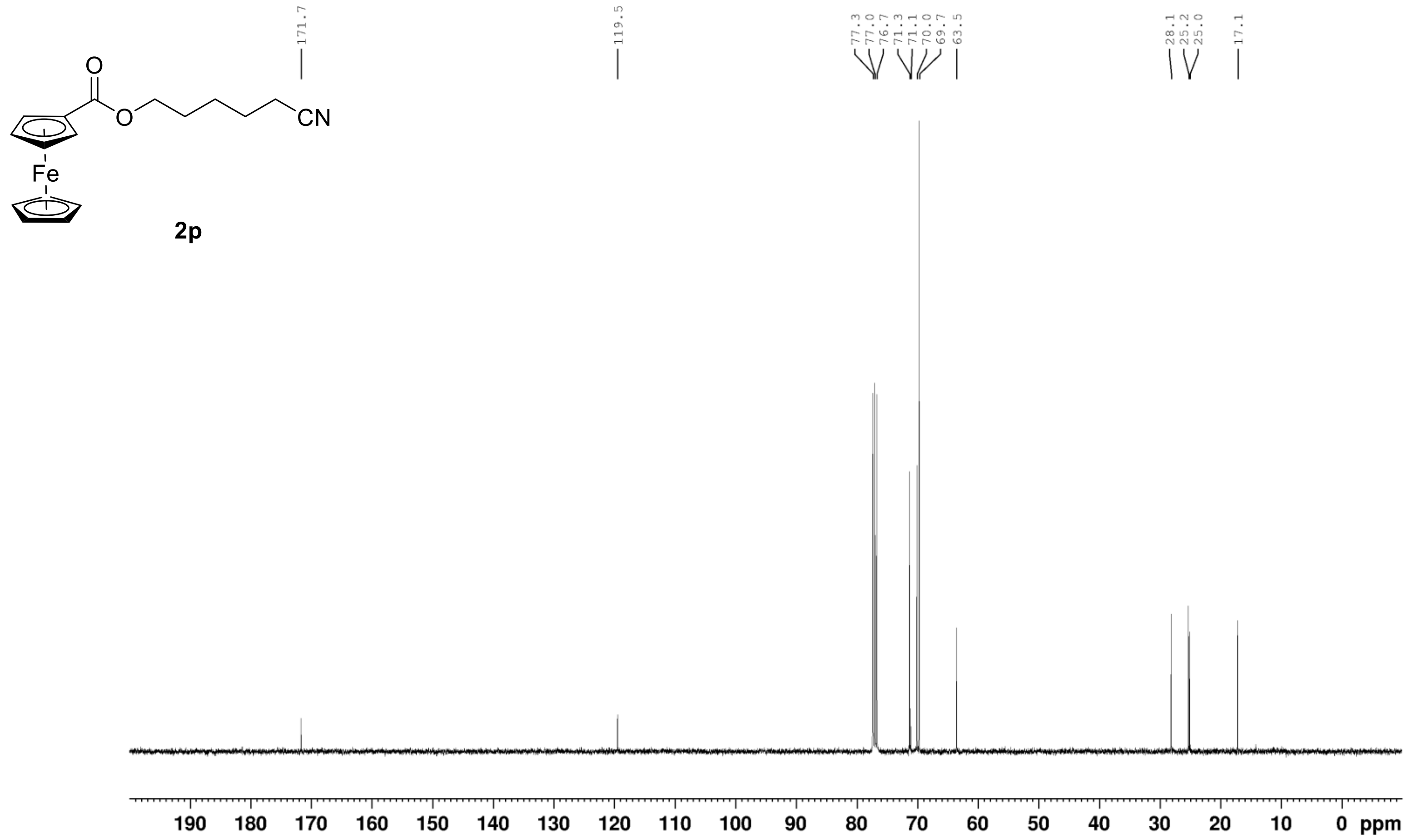

Figure S118. **^{1}H NMR** (500 MHz, $CDCl_3$) of **2q**.

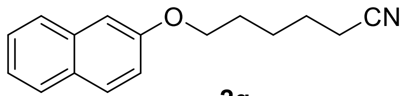

2q

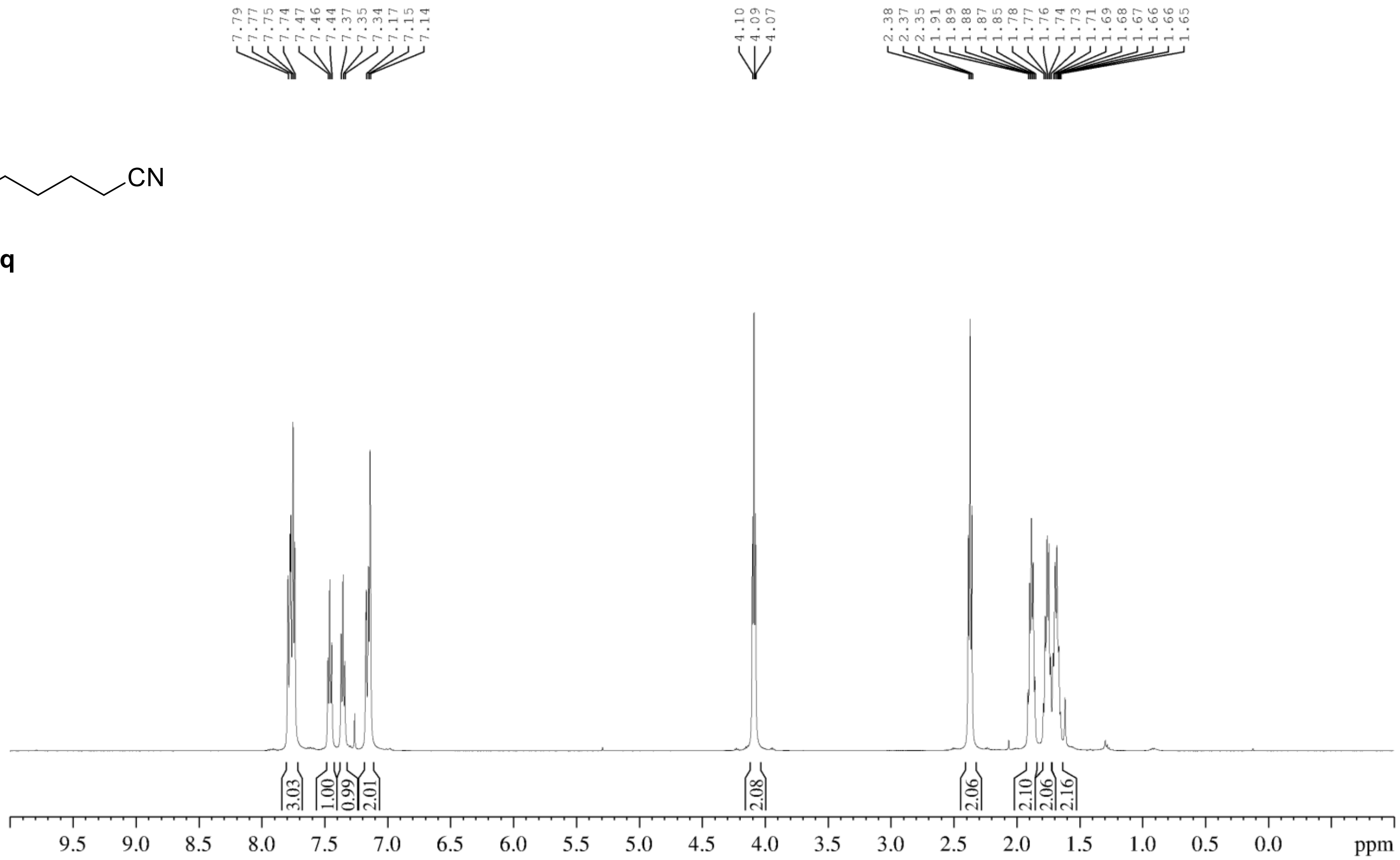

Figure S119. **^{13}C NMR** (126 MHz, $CDCl_3$) of **2q**.

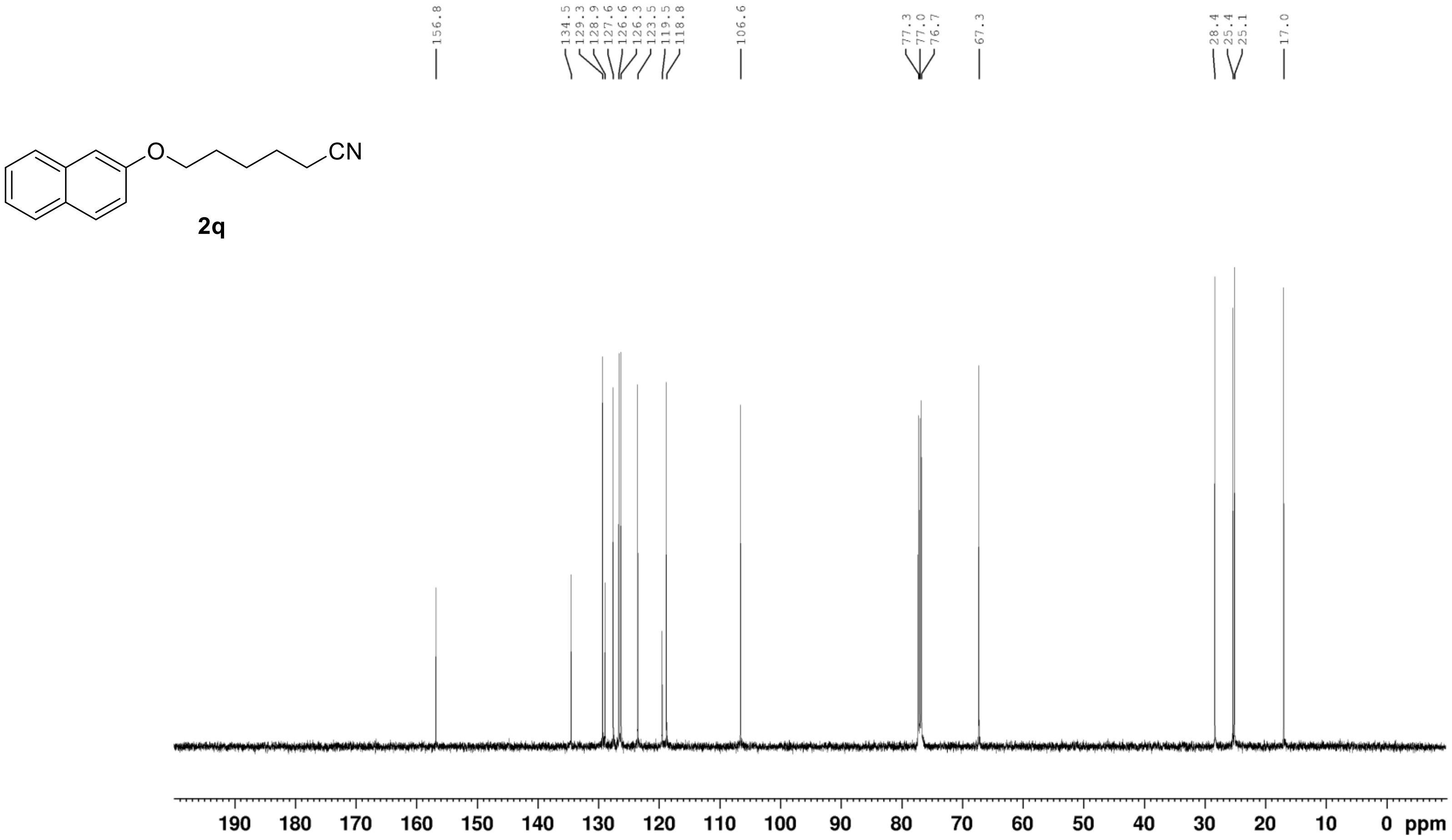

2q

Figure S120. **^{1}H NMR** (500 MHz, $CDCl_3$) of **2r**.

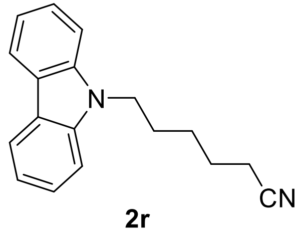

2r

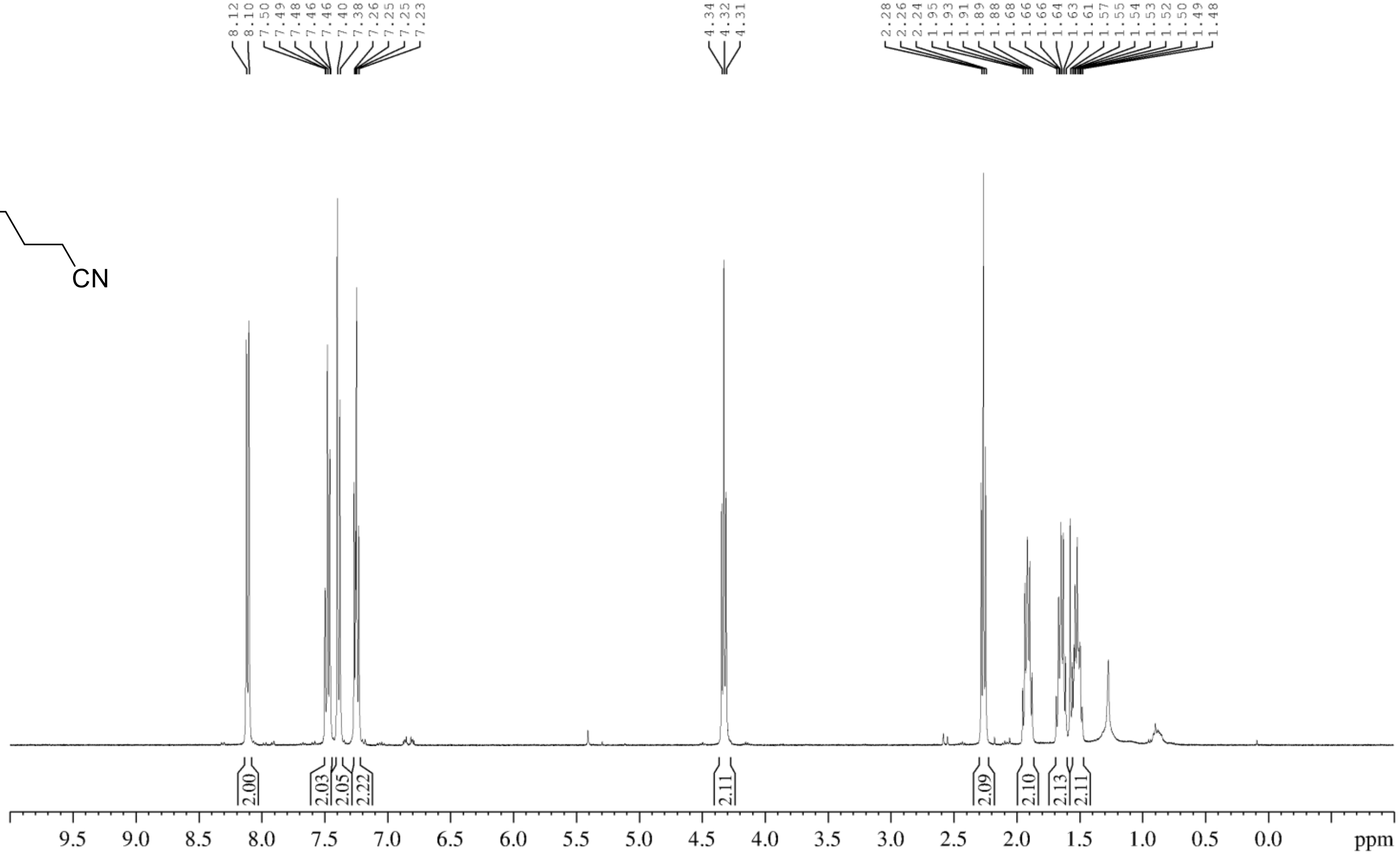

Figure S121. **^{13}C NMR** (126 MHz, $CDCl_3$) of **2r**.

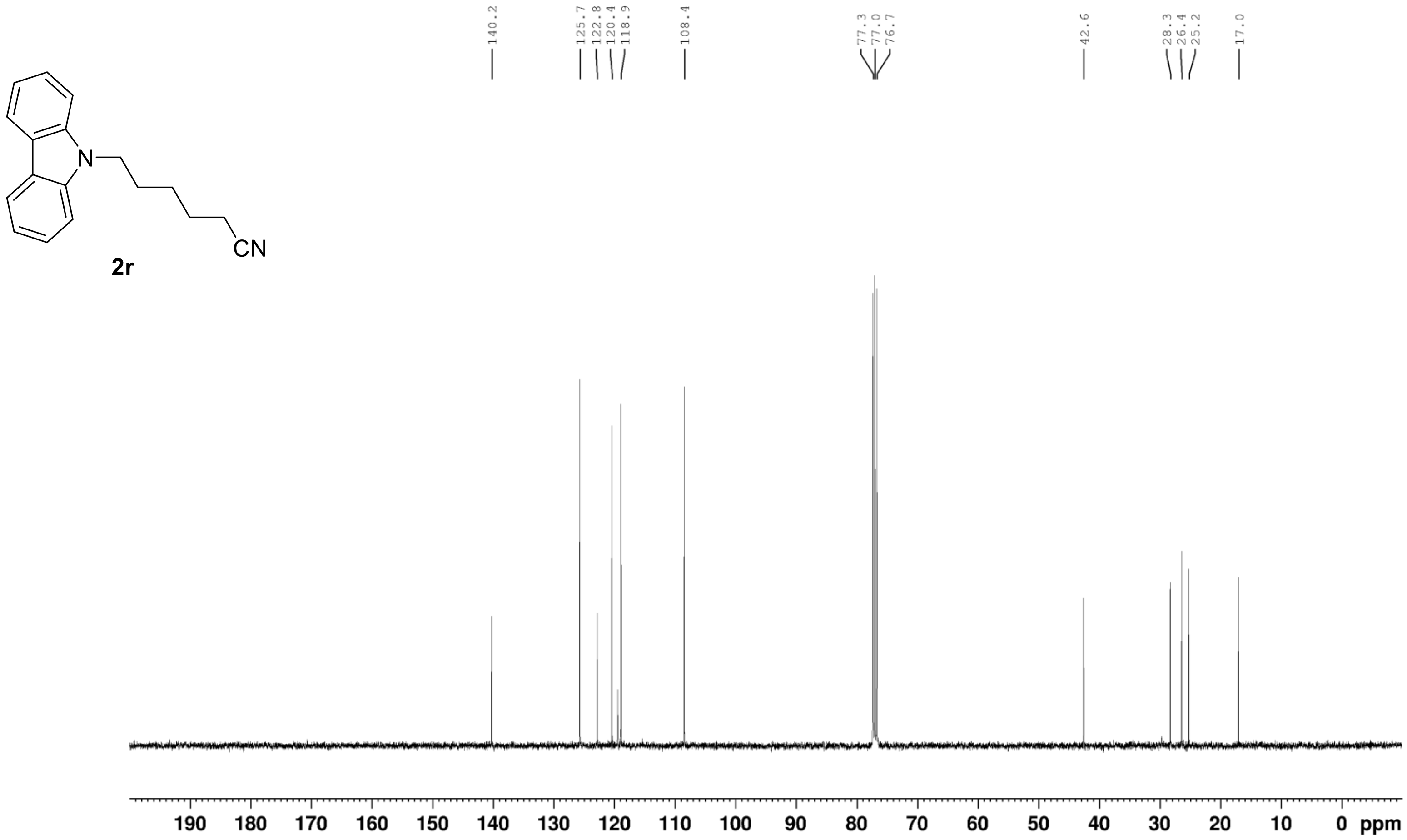

Figure S122. **¹H NMR** (500 MHz, $CDCl_3$) of **2s**.

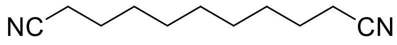

2s

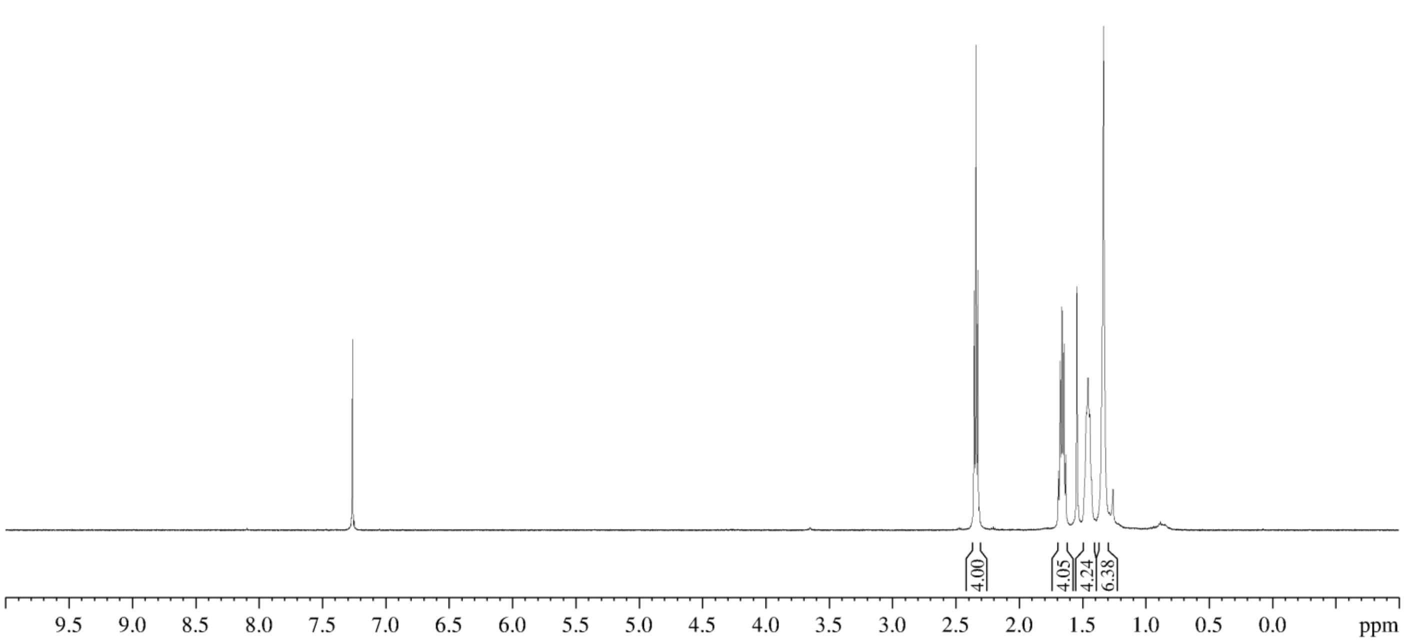

Figure S123. **^{13}C NMR** (126 MHz, $CDCl_3$) of **2s**.

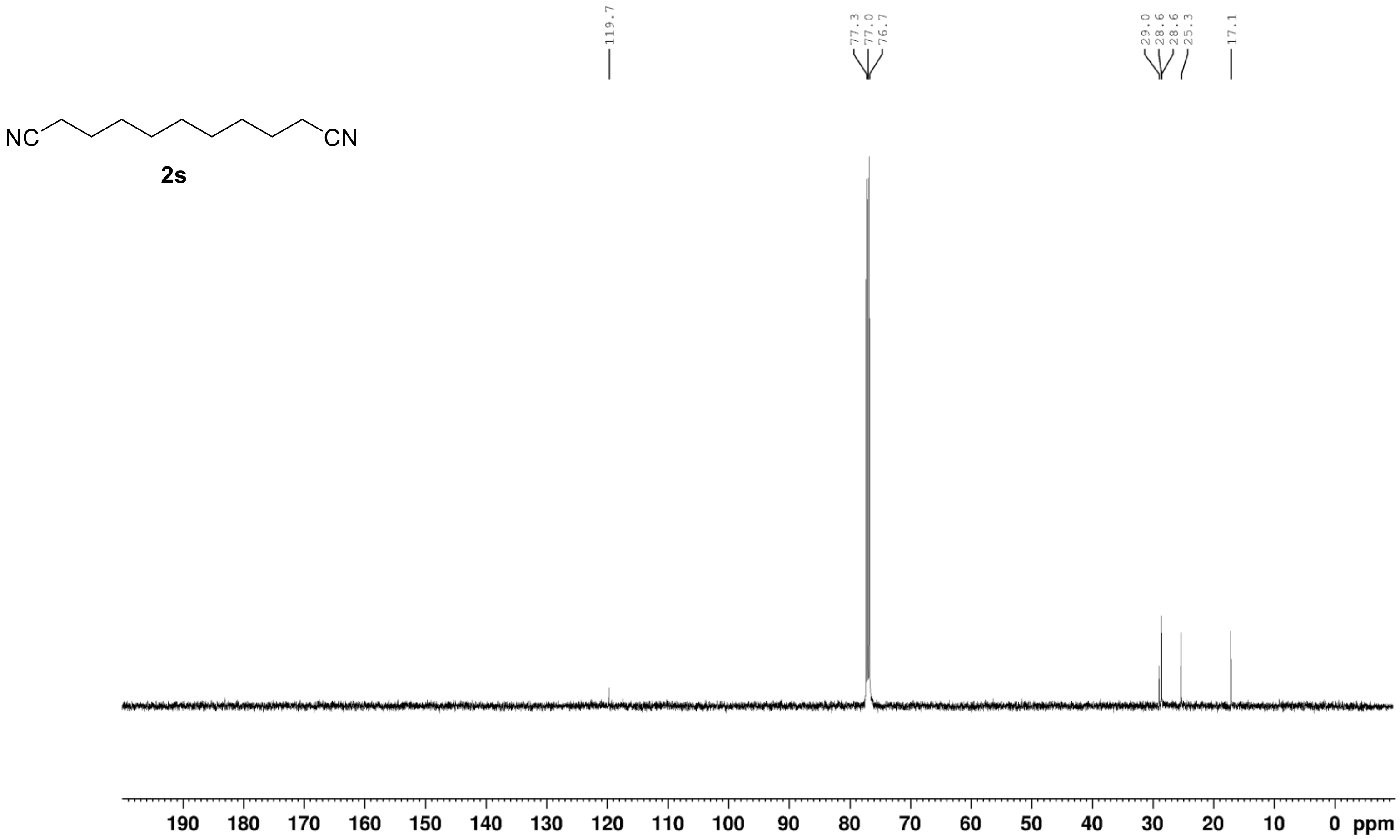

Figure S124. **^{1}H NMR** (500 MHz, $CDCl_3$) of **2t**.

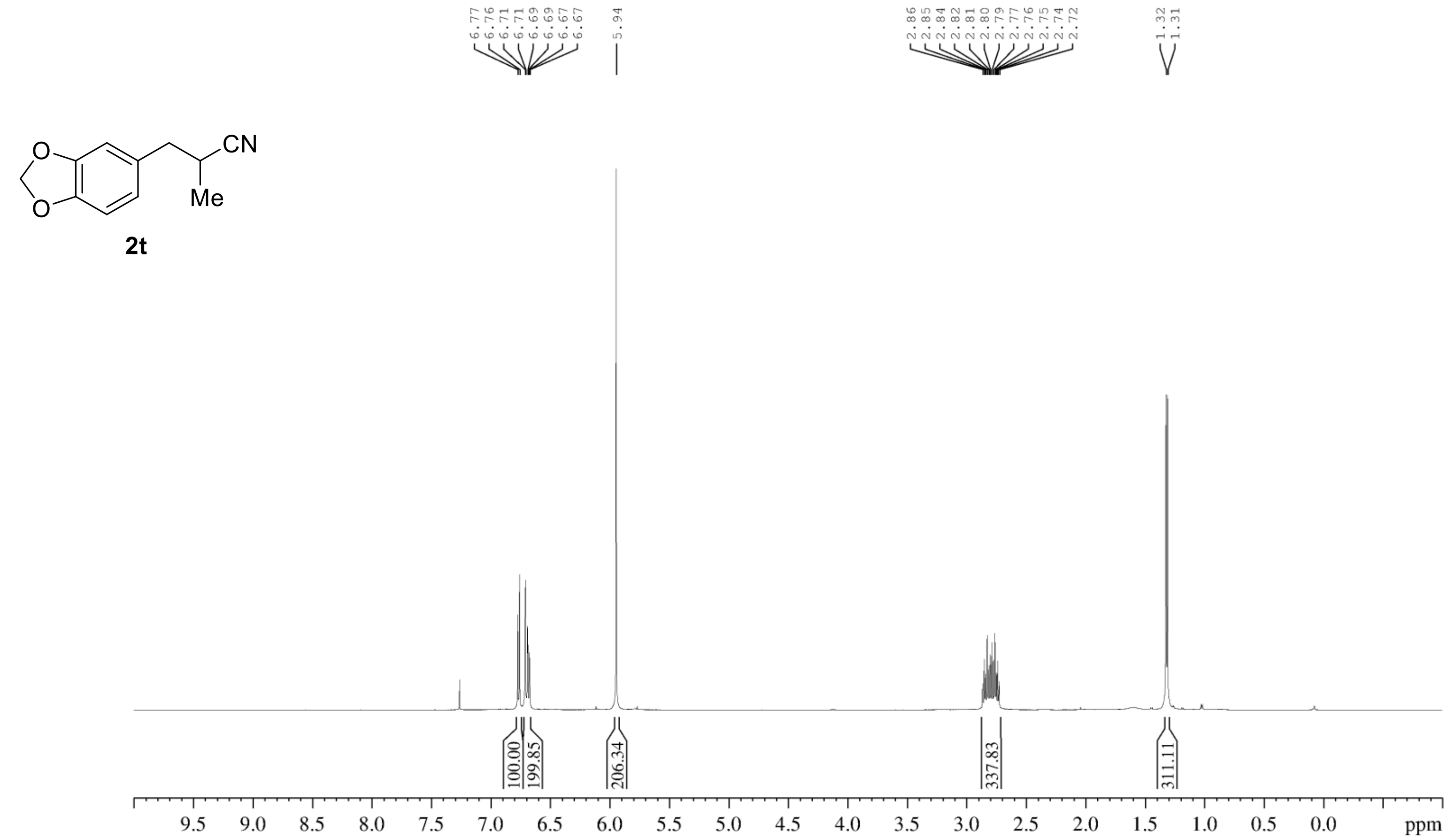

Figure S125. ^{13}**C NMR** (126 MHz, $CDCl_3$) of **2t**.

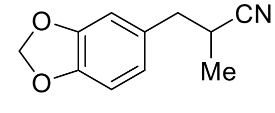

2t

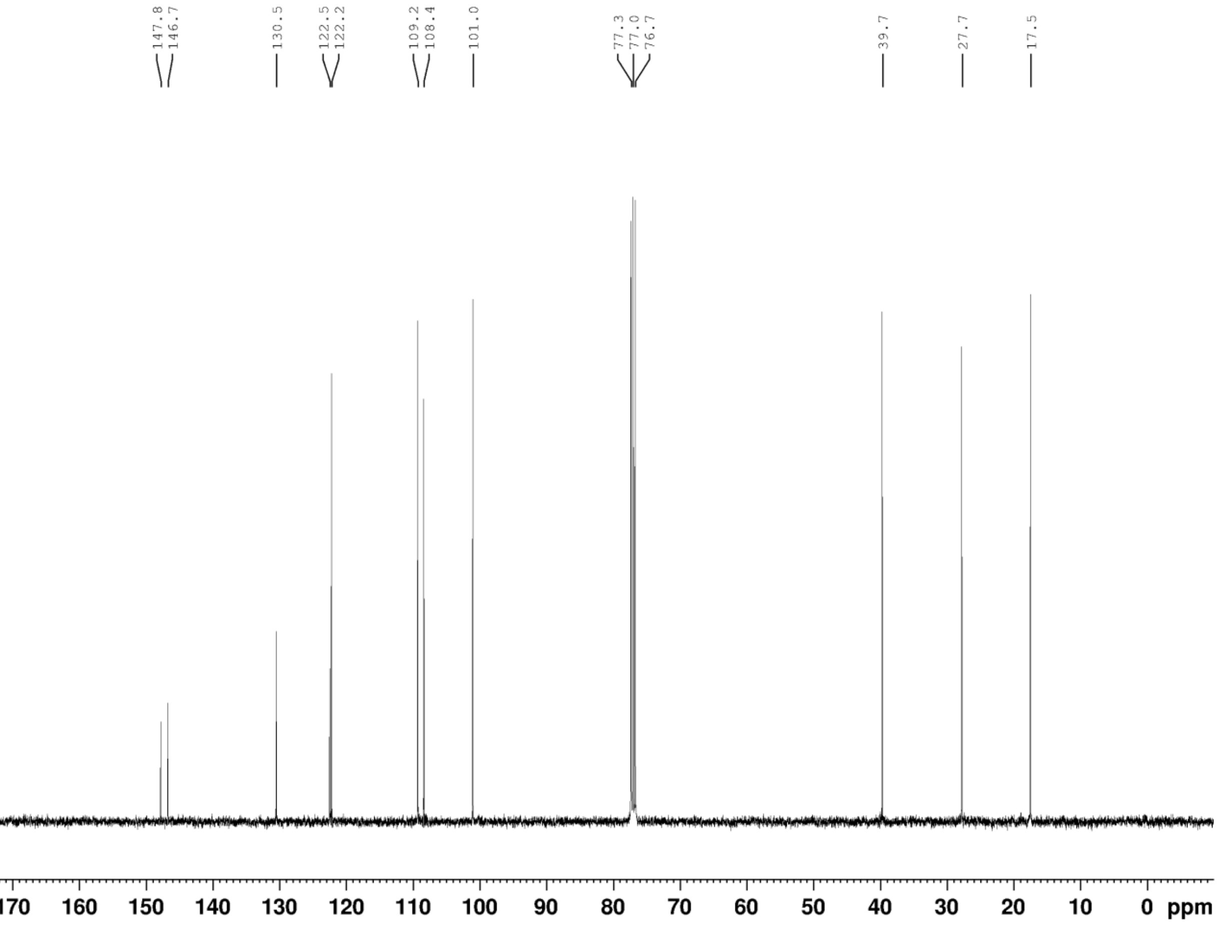

Figure S126. **¹H NMR** (500 MHz, $CDCl_3$) of **2u-rac**.

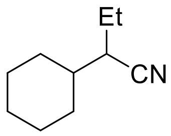

2u-rac

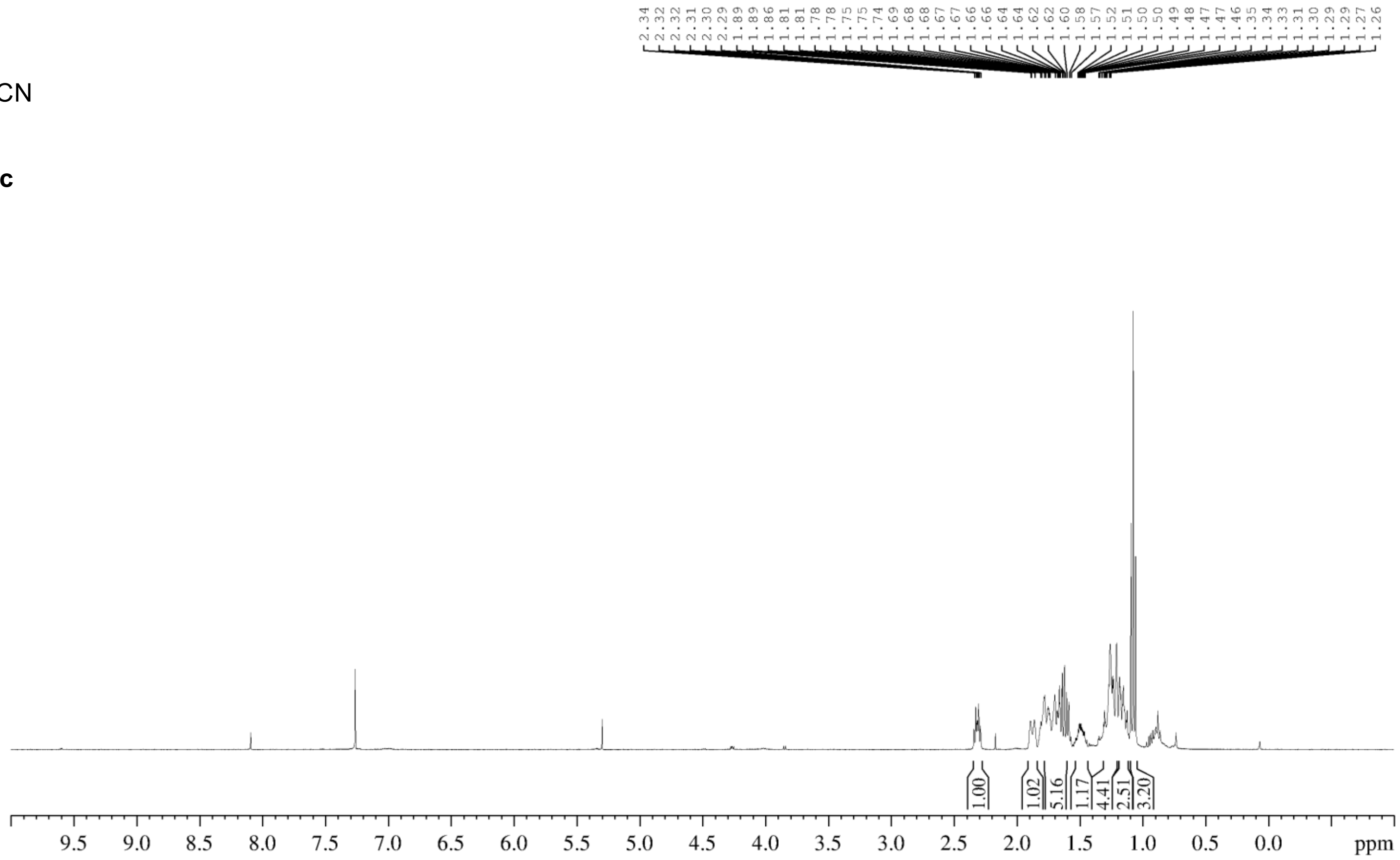

Figure S127. **^{13}C NMR** (126 MHz, $CDCl_3$) of **2u-rac**.

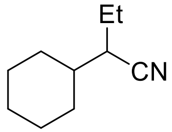

2u-rac

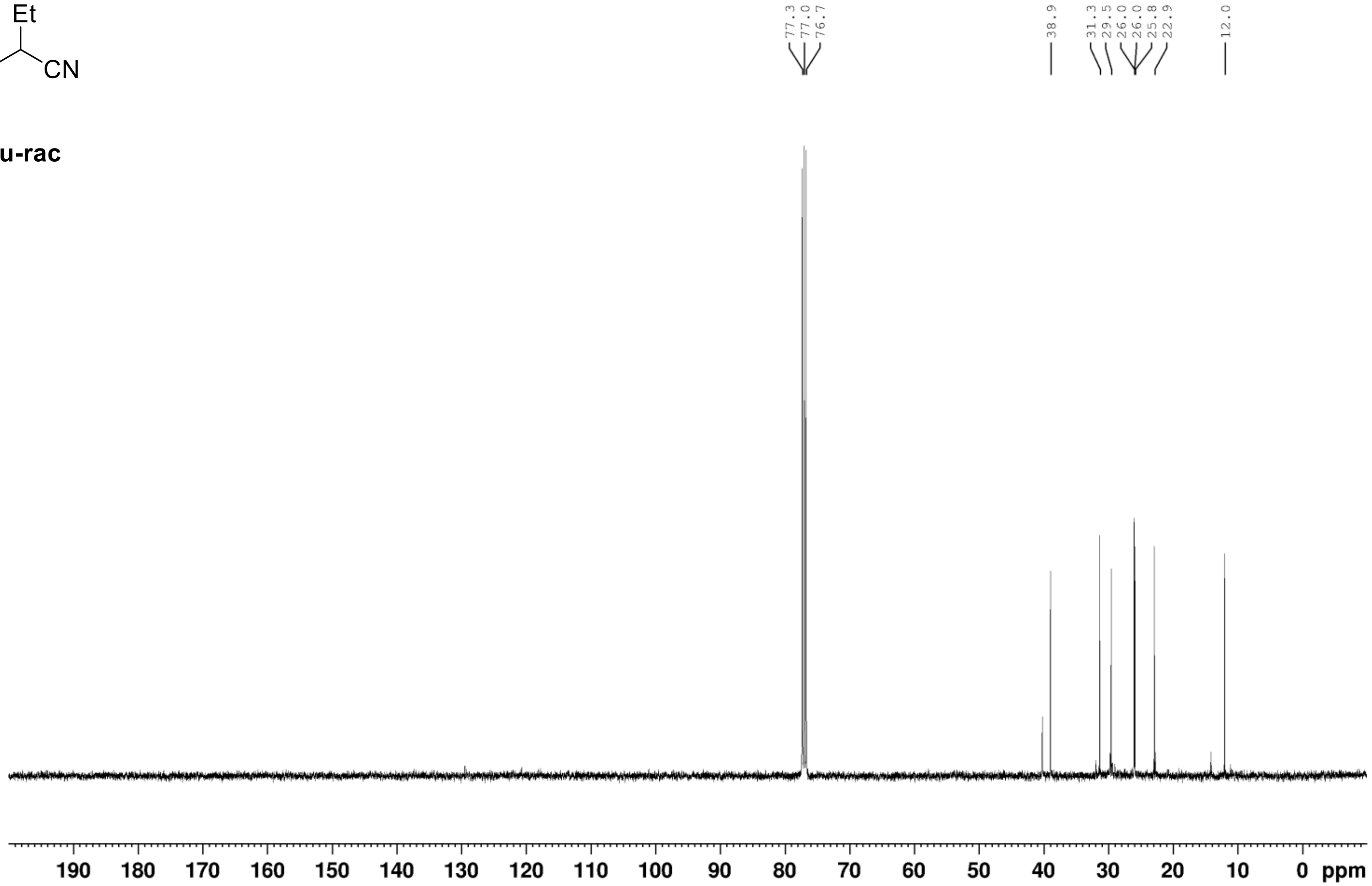

Figure S128. **^{1}H NMR** (500 MHz, $CDCl_3$) of **2u**.

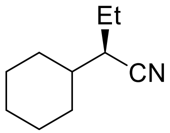

2u

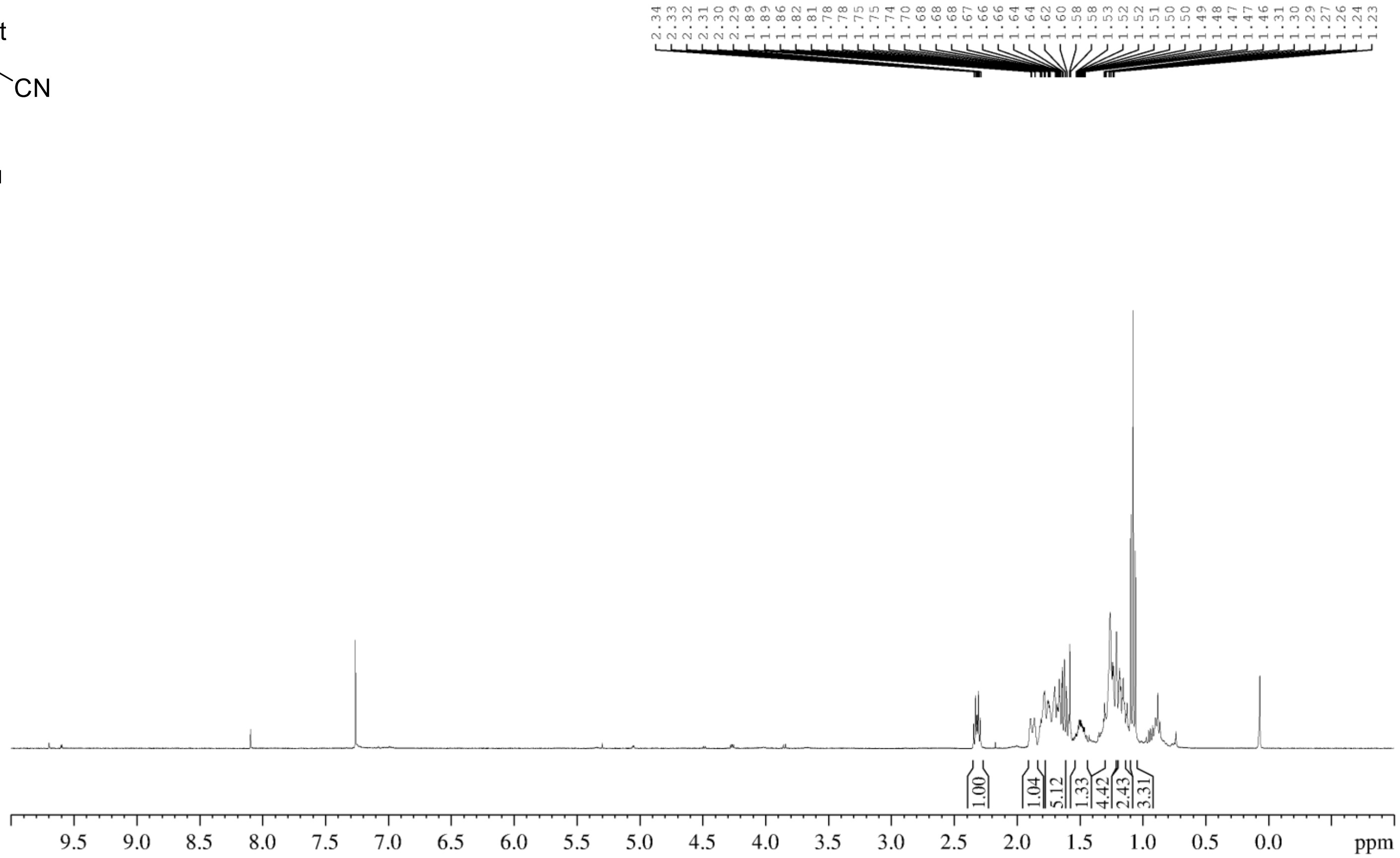

Figure S129. ^{13}**C NMR** (126 MHz, $CDCl_3$) of **2u**.

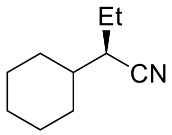

2u

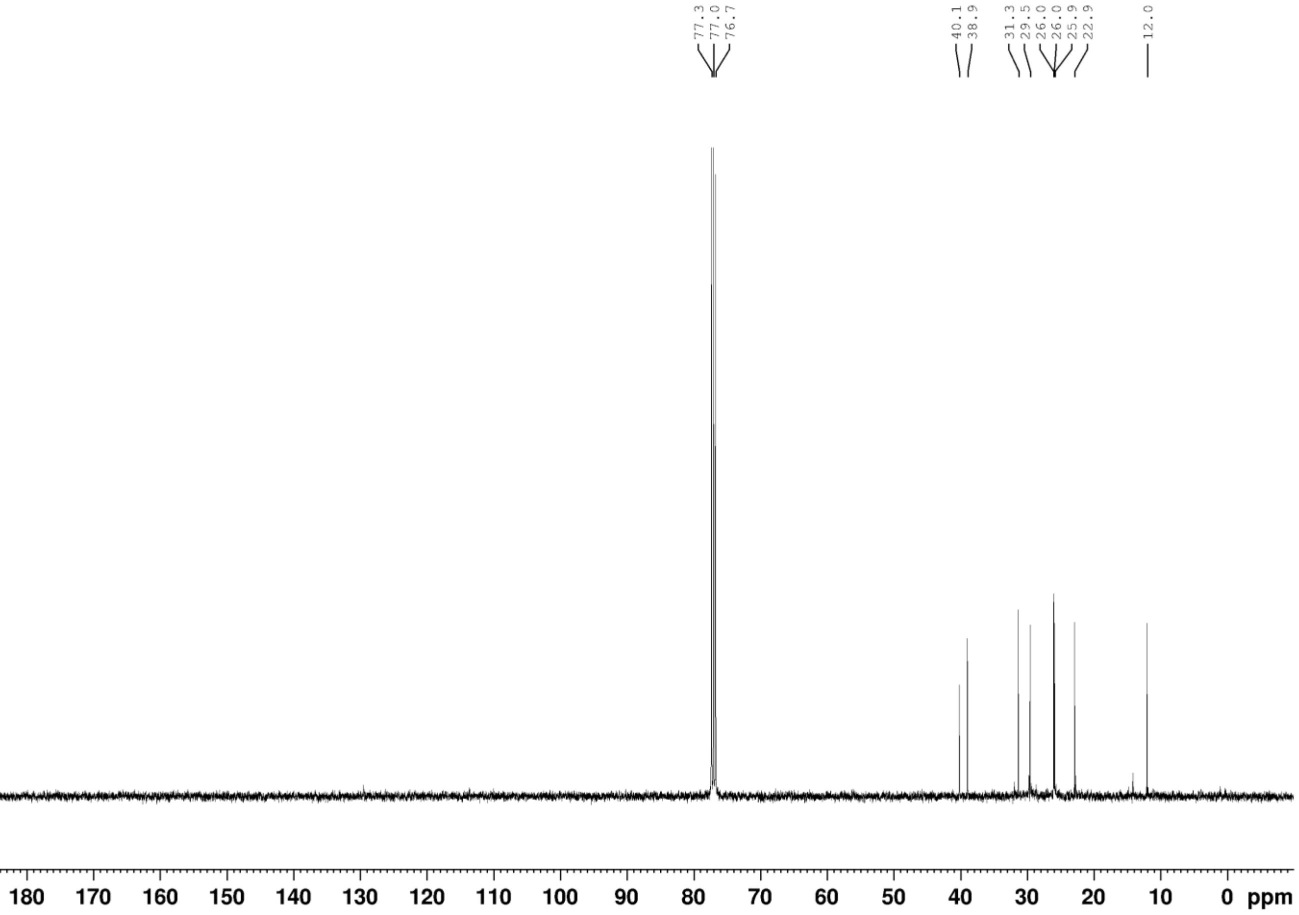

Figure S130. **^{1}H NMR** (500 MHz, $CDCl_3$) of **4a**.

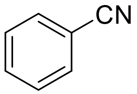

4a

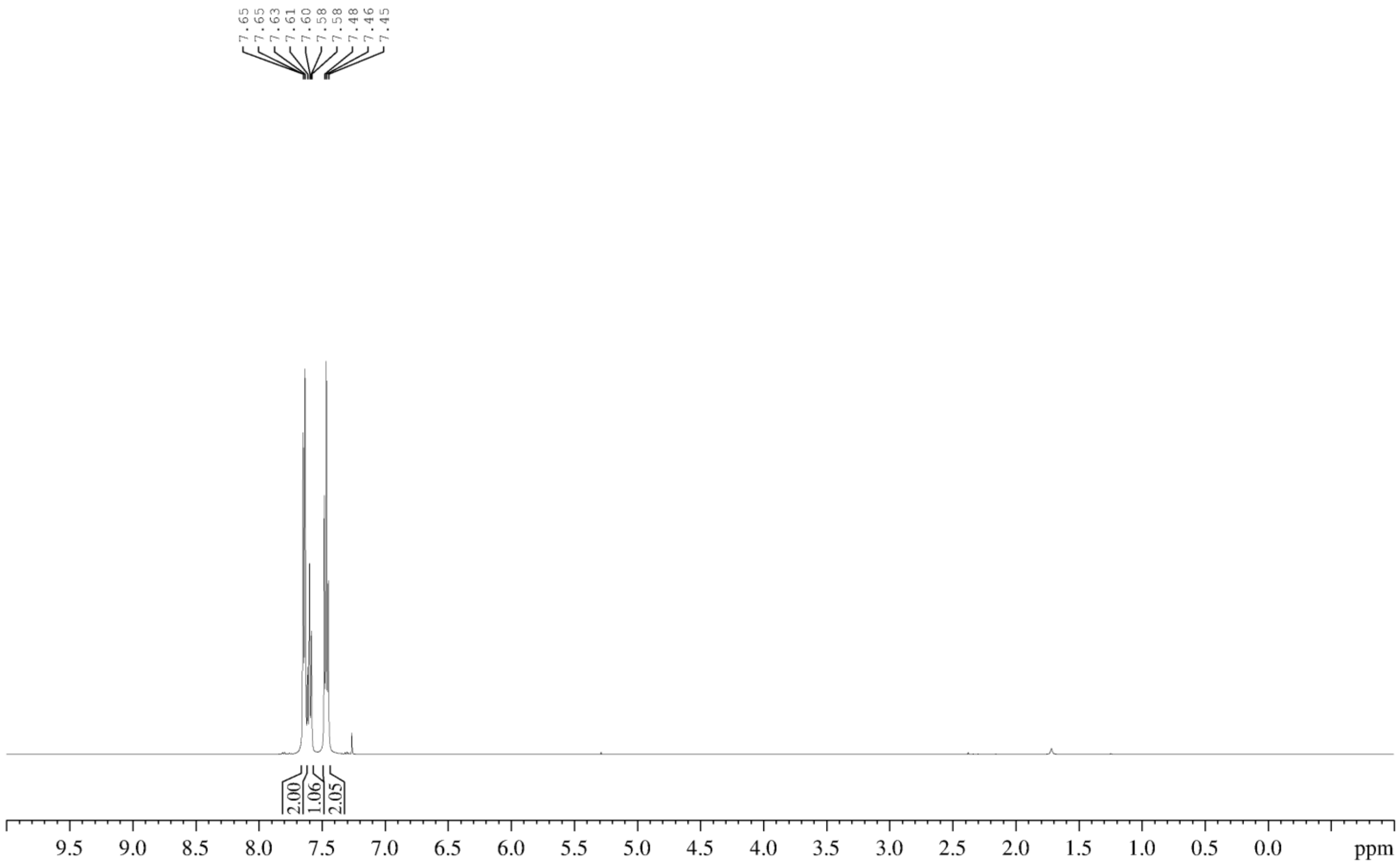

Figure S131. ^{13}**C NMR** (126 MHz, $CDCl_3$) of **4a**.

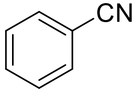

4a

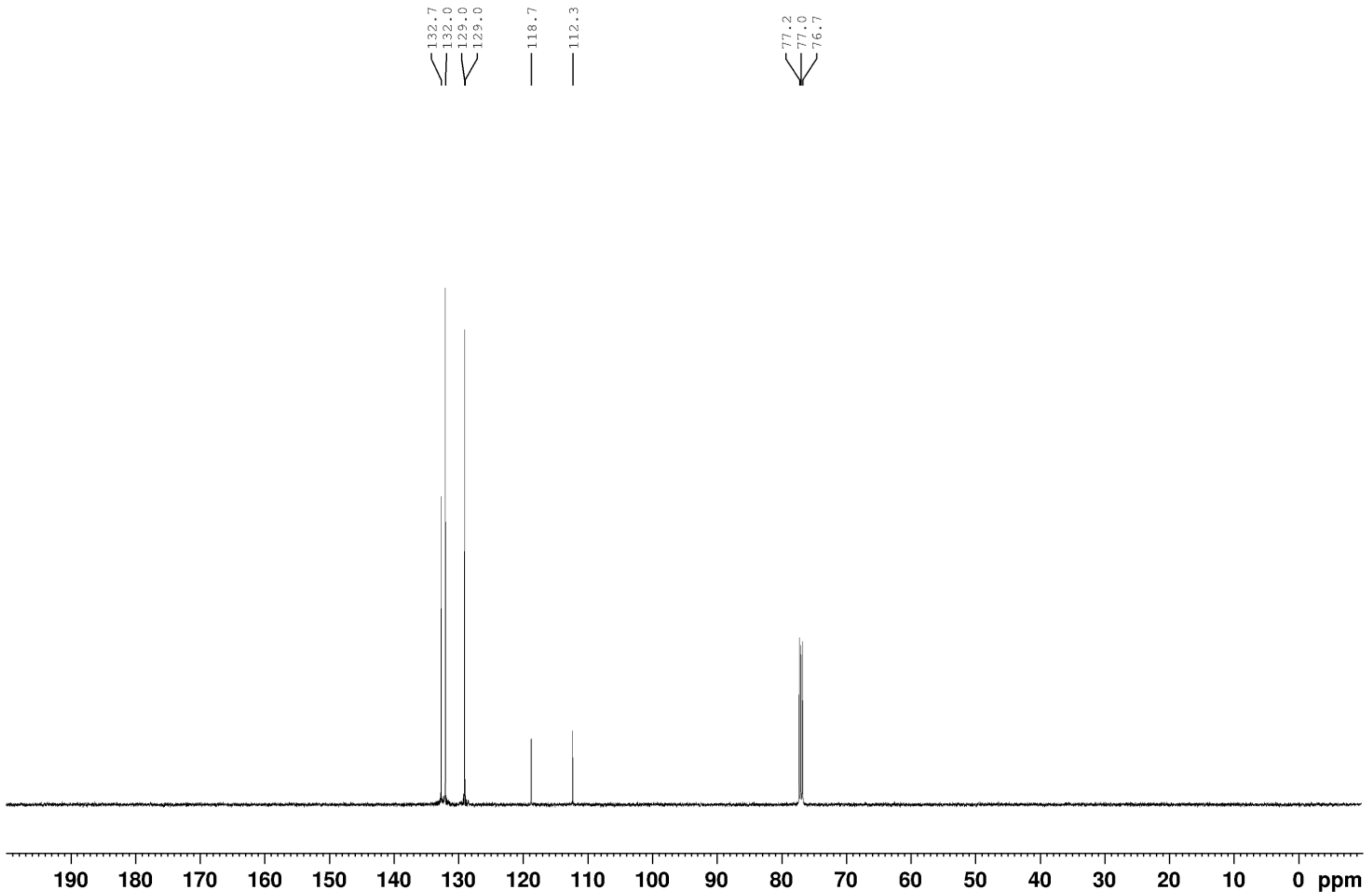

Figure S132. **¹H NMR** (500 MHz, $CDCl_3$) of **4b**.

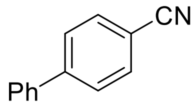

4b

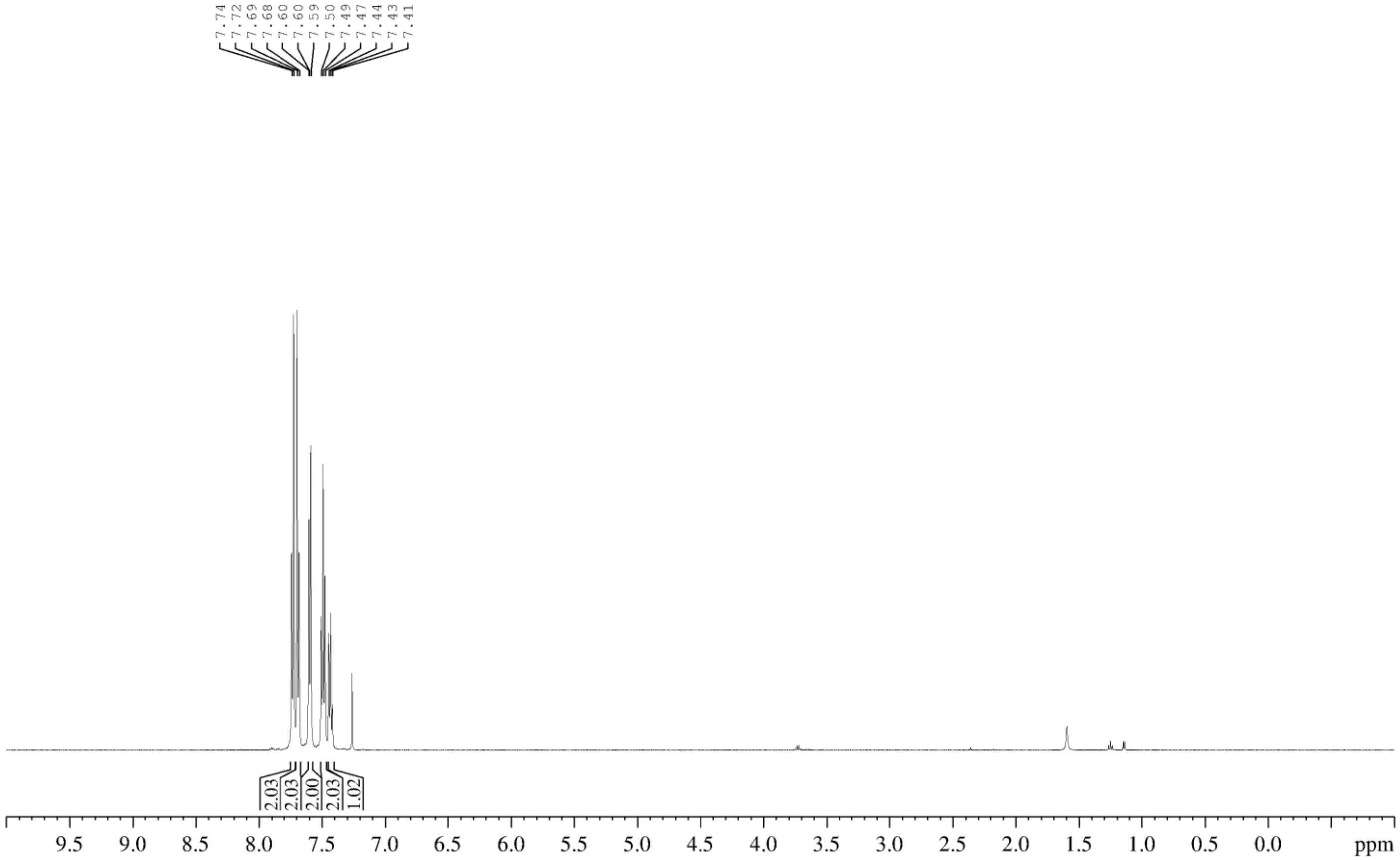

Figure S133. ^{13}**C NMR** (126 MHz, $CDCl_3$) of **4b**.

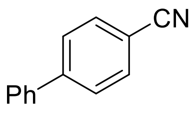

4b

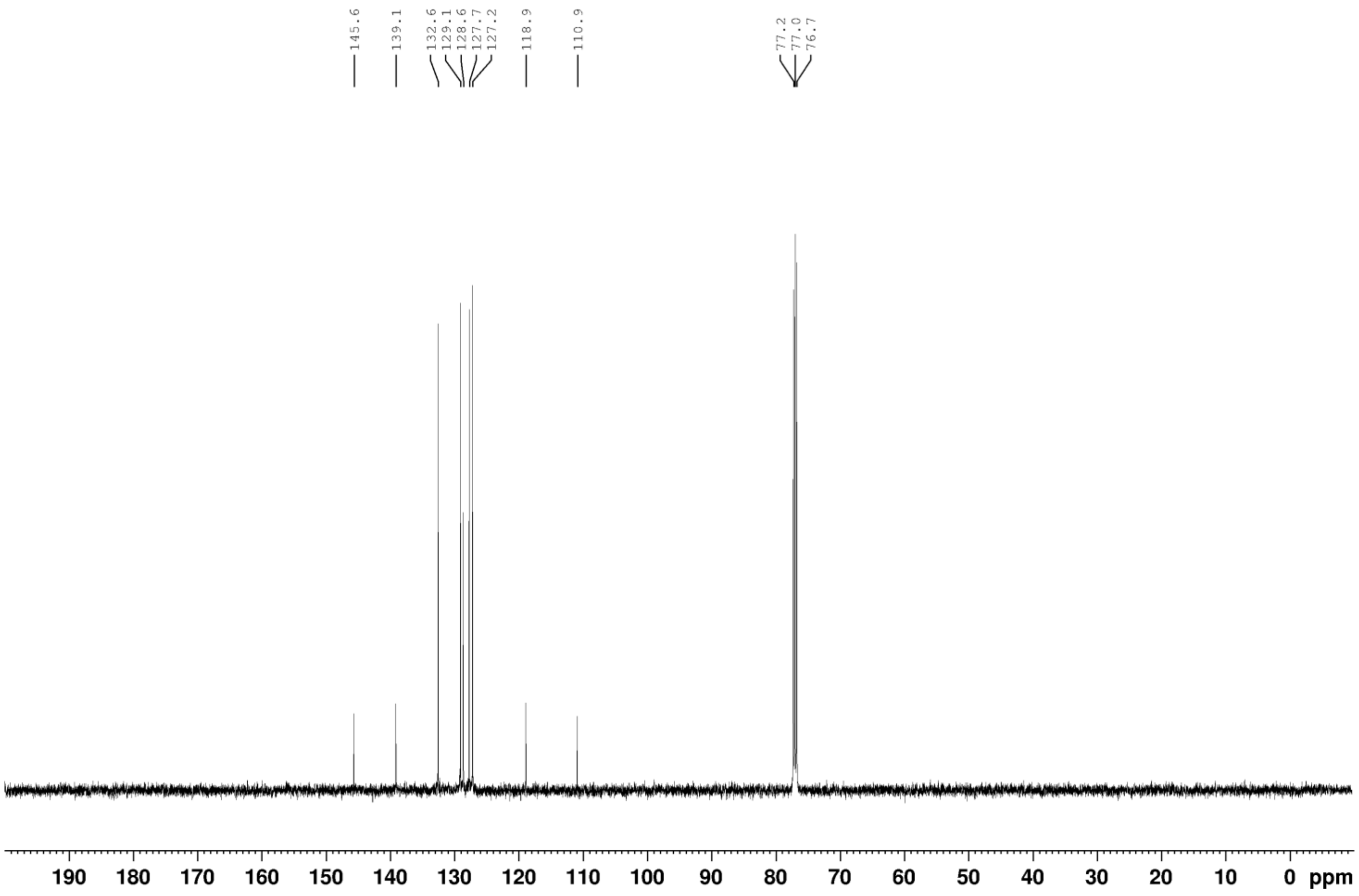

Figure S134. ^{1}**H NMR** (500 MHz, $CDCl_3$) of **4c**.

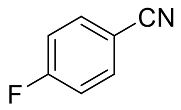

4c

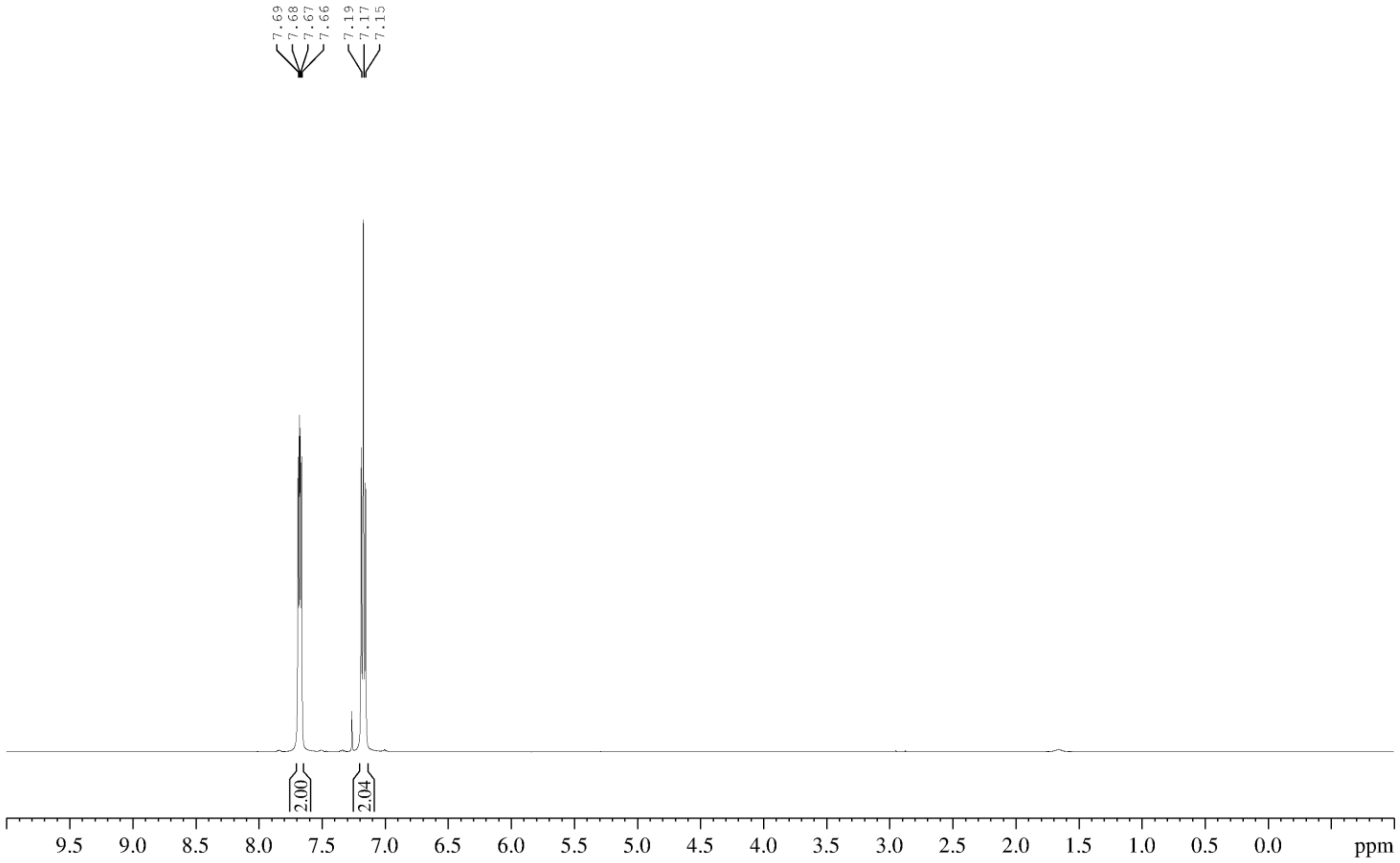

Figure S135. ^{13}**C NMR** (126 MHz, $CDCl_3$) of **4c**.

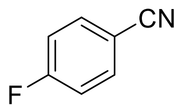

4c

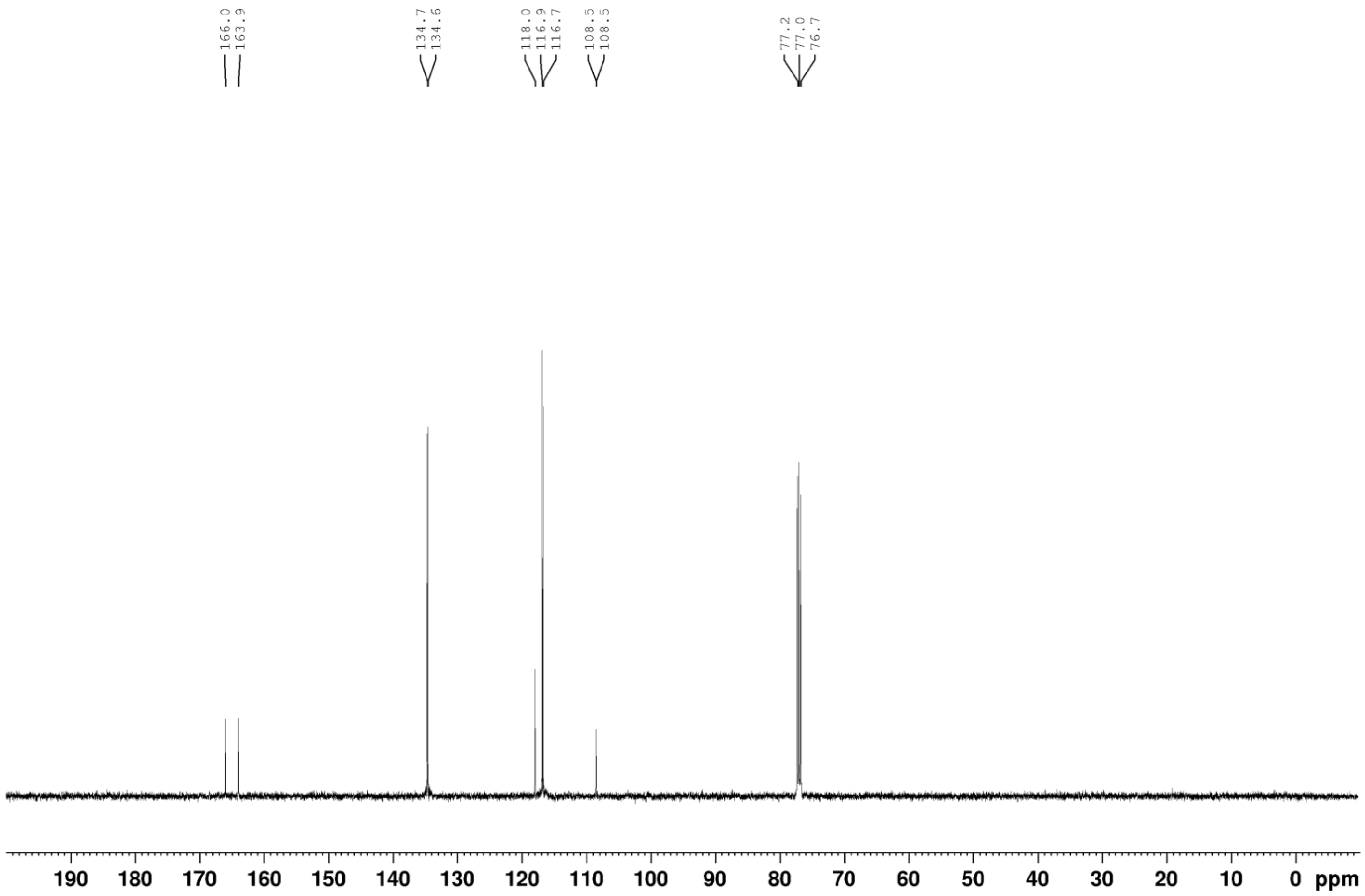

Figure S136. **^{19}F NMR** (471 MHz, $CDCl_3$) of **4c**.

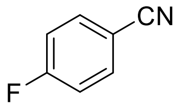

4c

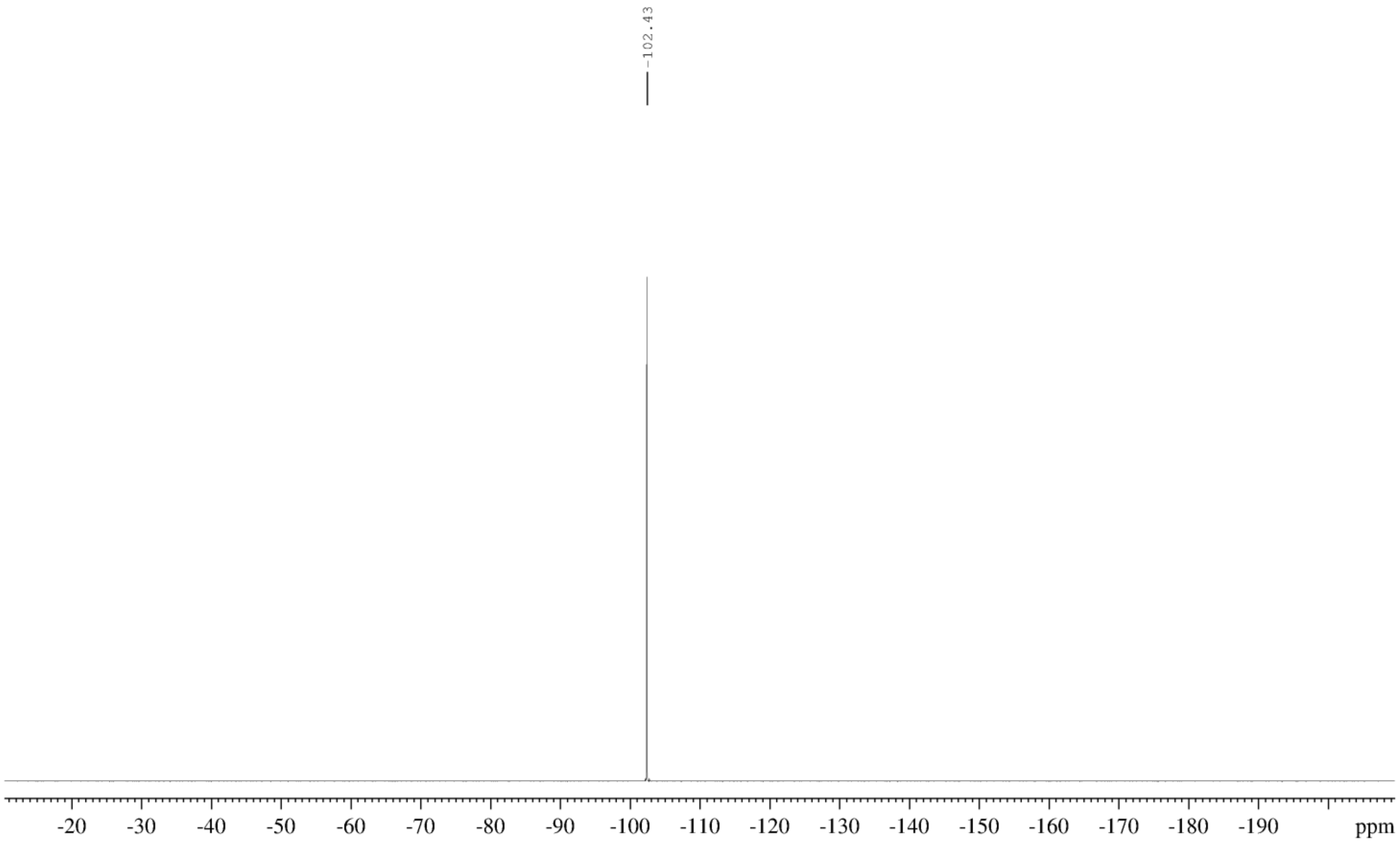

Figure S137. **^{1}H NMR** (500 MHz, $CDCl_3$) of **4d**.

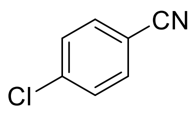

4d

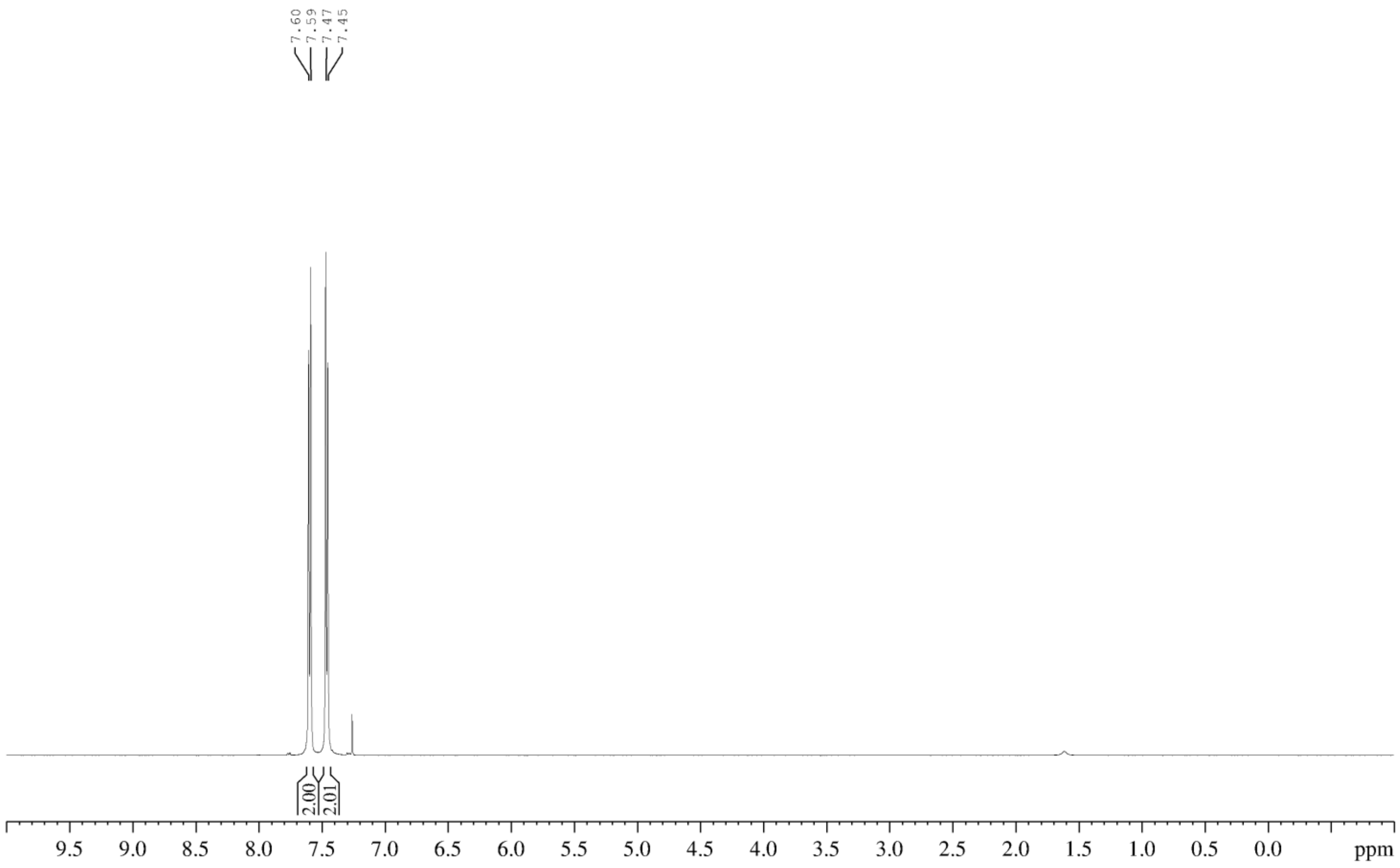

Figure S138. ^{13}**C NMR** (126 MHz, $CDCl_3$) of **4d**.

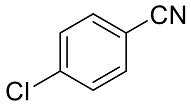

4d

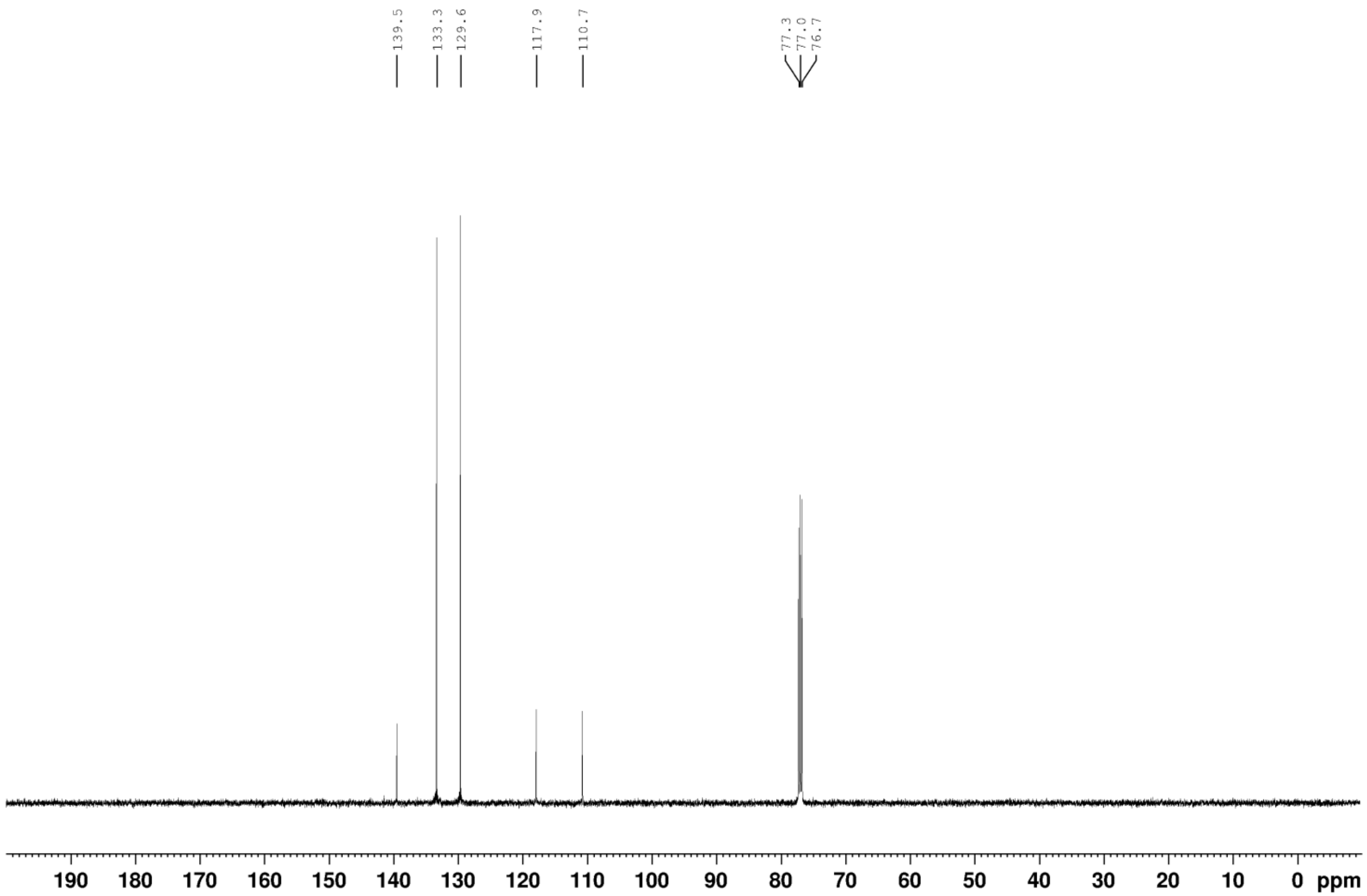

Figure S139. ^{1}H NMR (500 MHz, CDCl$_3$) of **4e**.

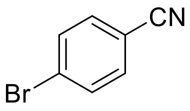

4e

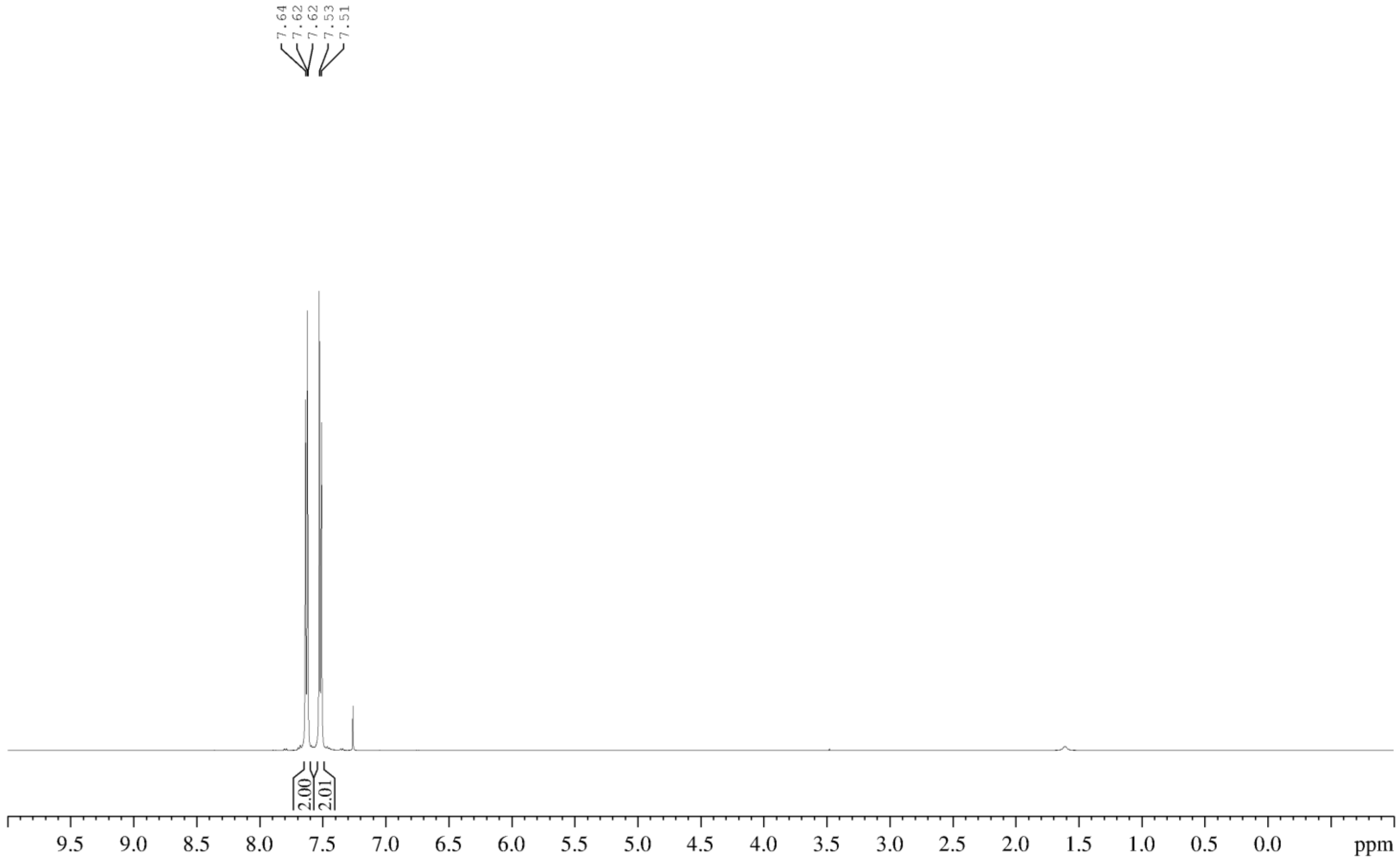

Figure S140. ^{13}**C NMR** (126 MHz, $CDCl_3$) of **4e**.

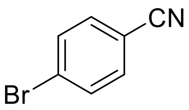

4e

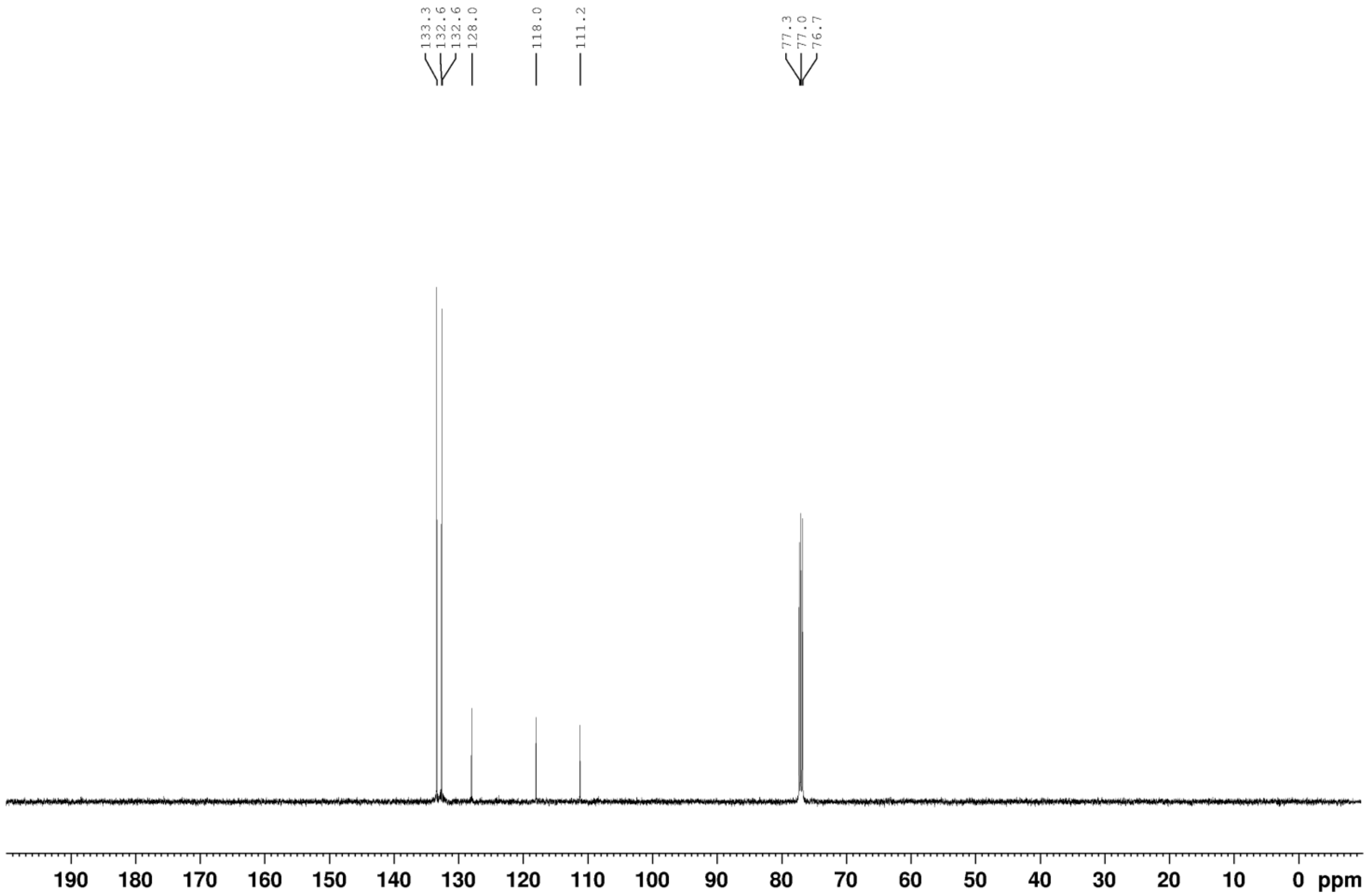

Figure S141. **^{1}H NMR** (500 MHz, $CDCl_3$) of **4f**.

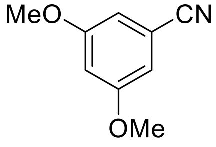

4f

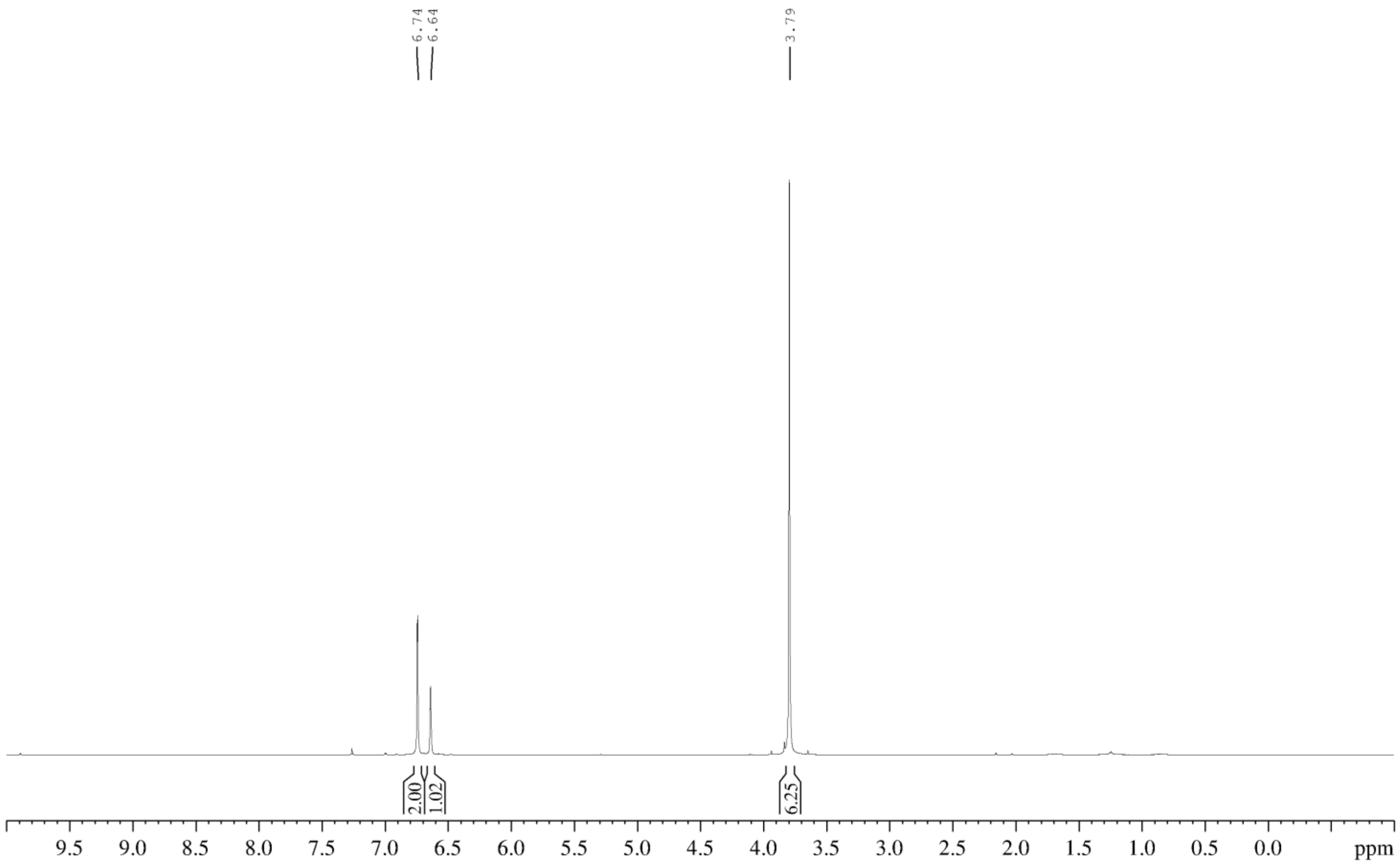

Figure S142. ^{13}**C NMR** (126 MHz, $CDCl_3$) of **4f**.

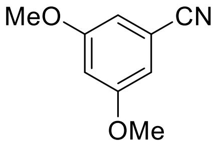

4f

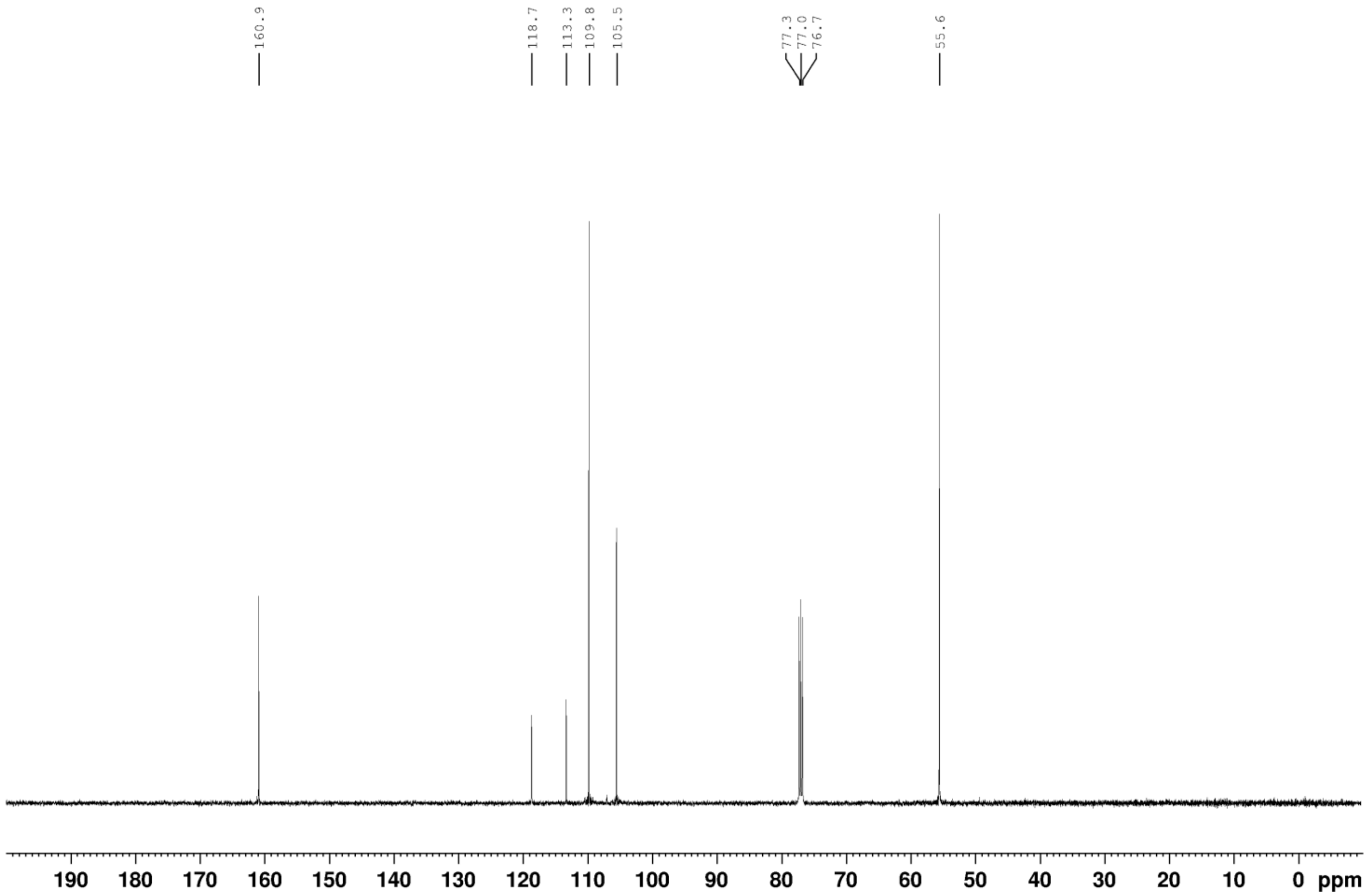

Figure S143. **¹H NMR** (500 MHz, $CDCl_3$) of **4g**.

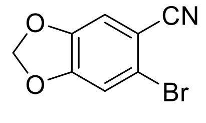

4g

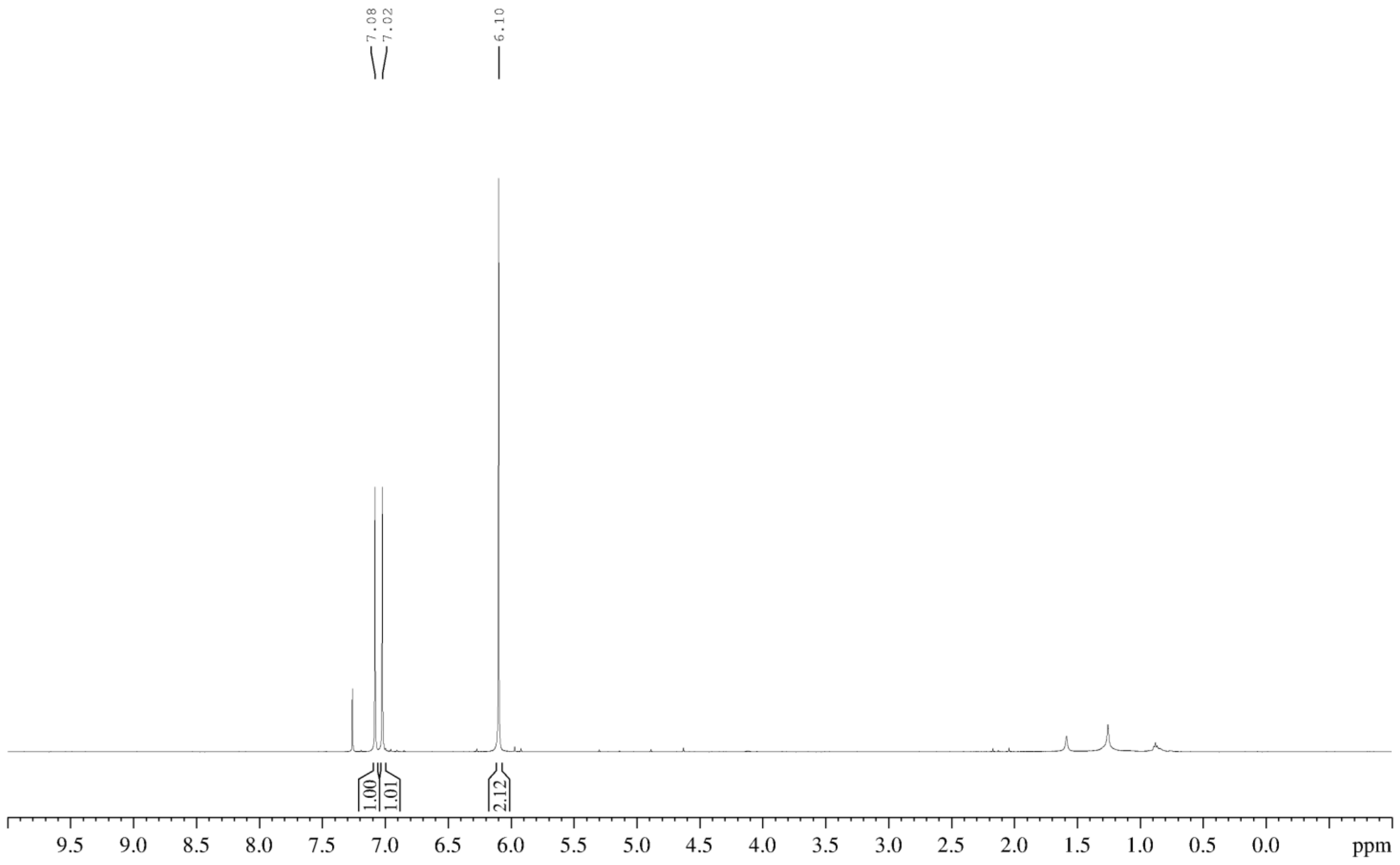

Figure S144. **^{13}C NMR** (126 MHz, $CDCl_3$) of **4g**.

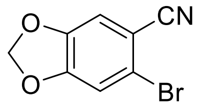

4g

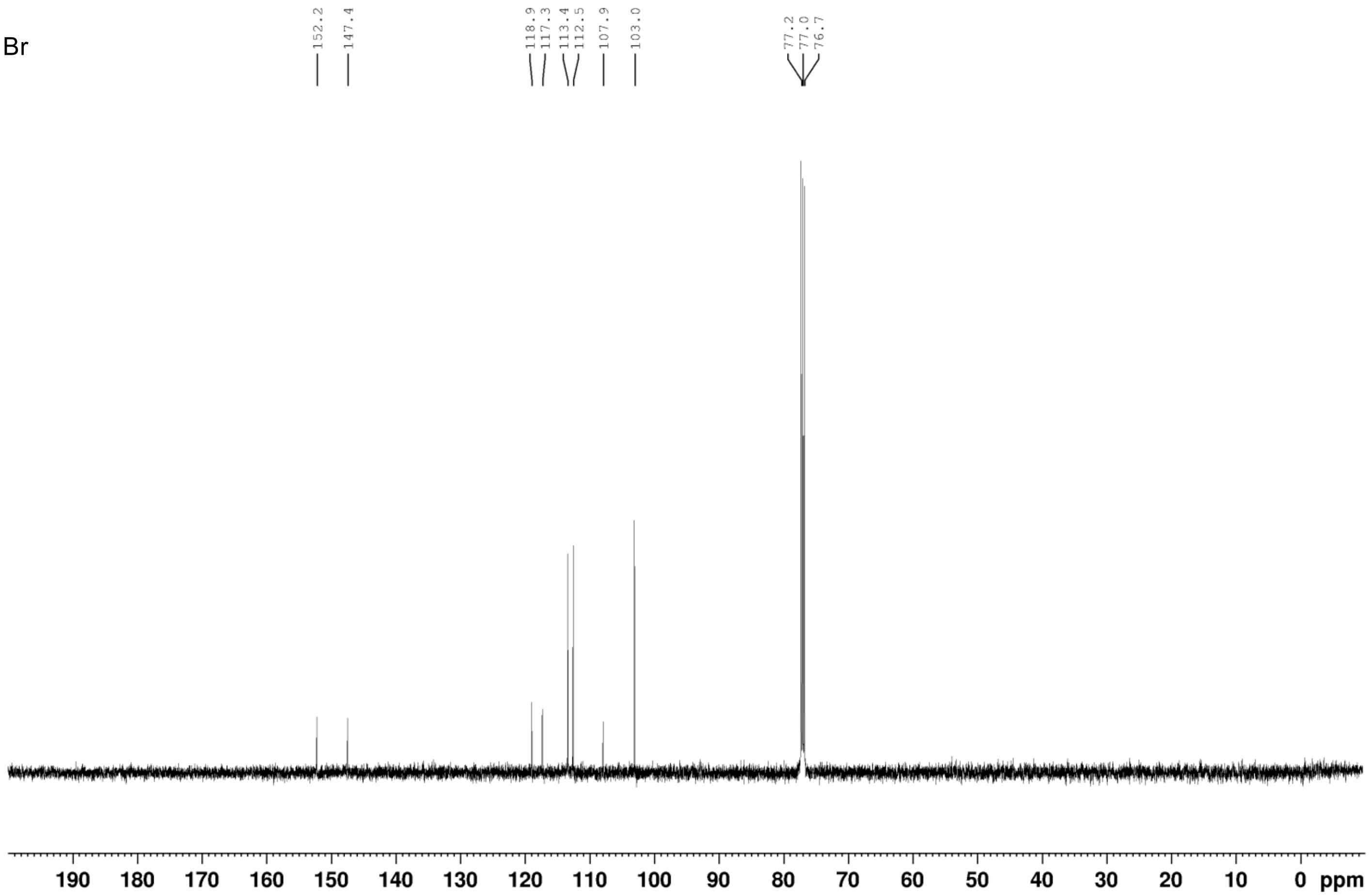

Figure S145. **^{1}H NMR** (500 MHz, $CDCl_3$) of **6a**.

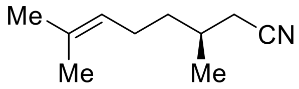

6a

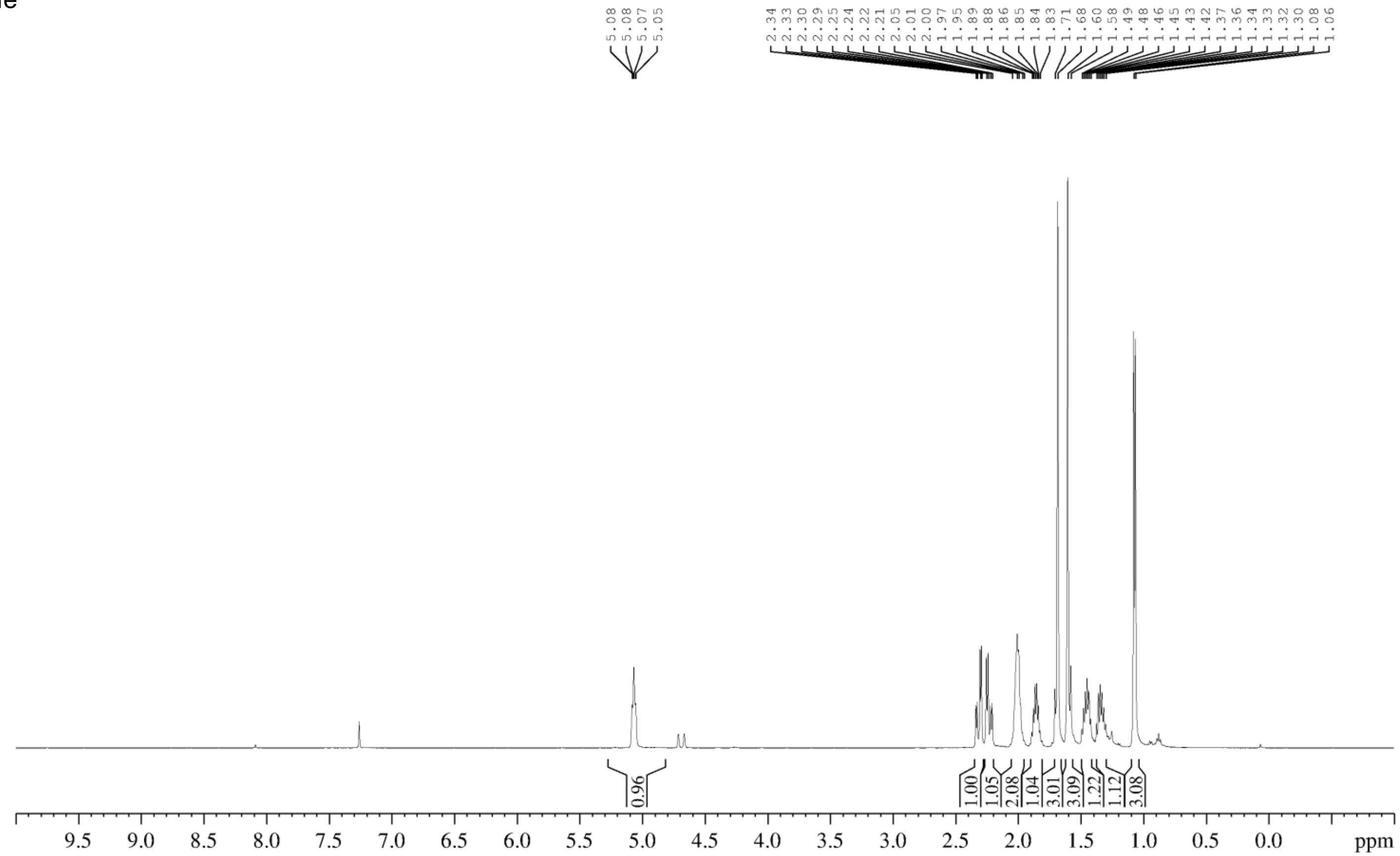

Figure S146. **^{13}C NMR** (126 MHz, $CDCl_3$) of **6a**.

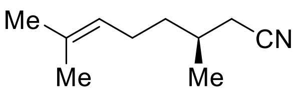

6a

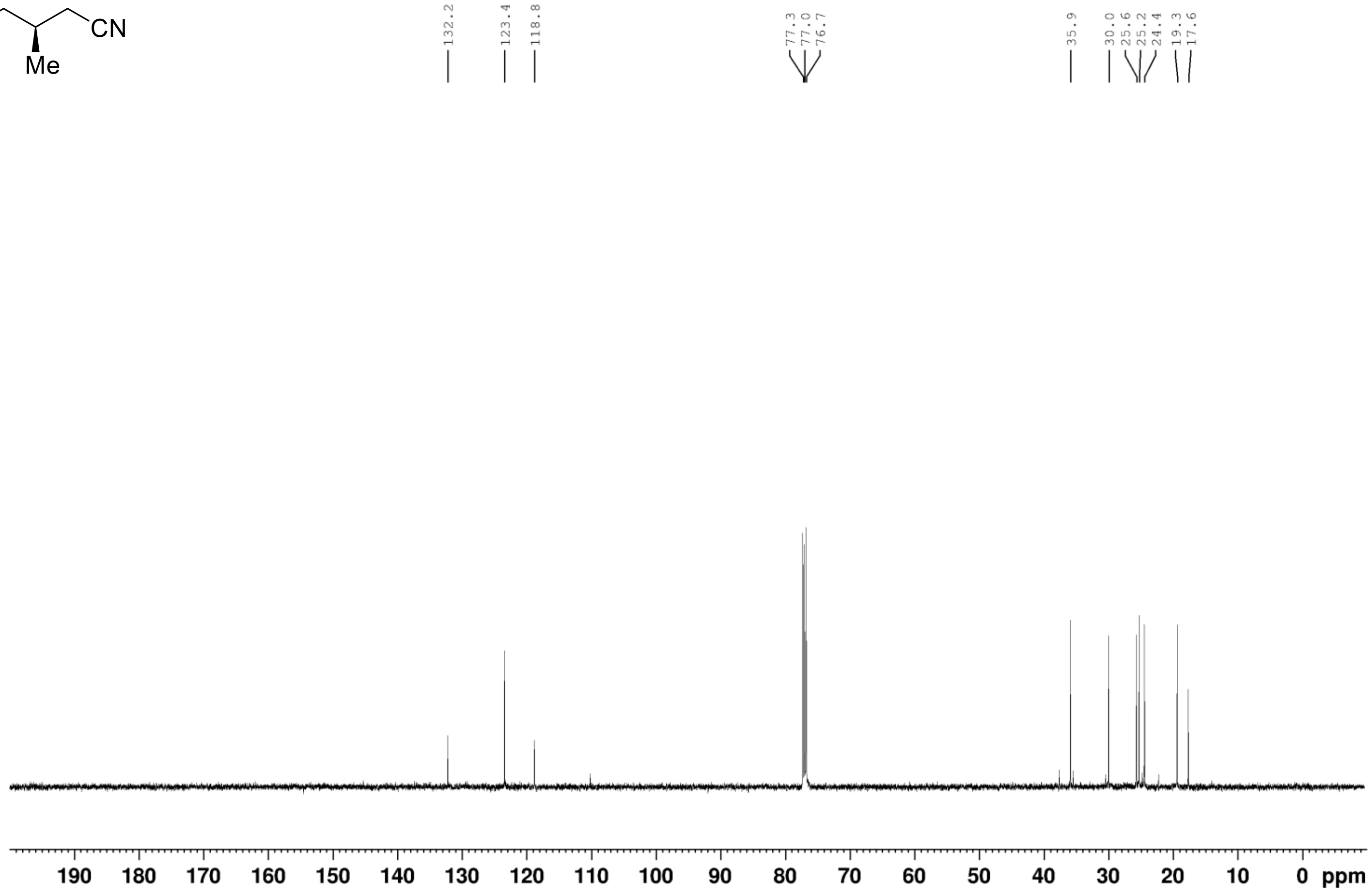

Figure S147. **¹H NMR** (500 MHz, $CDCl_3$) of **6b**.

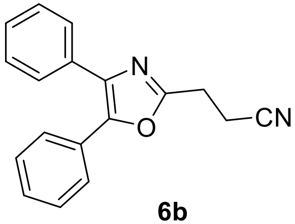

6b

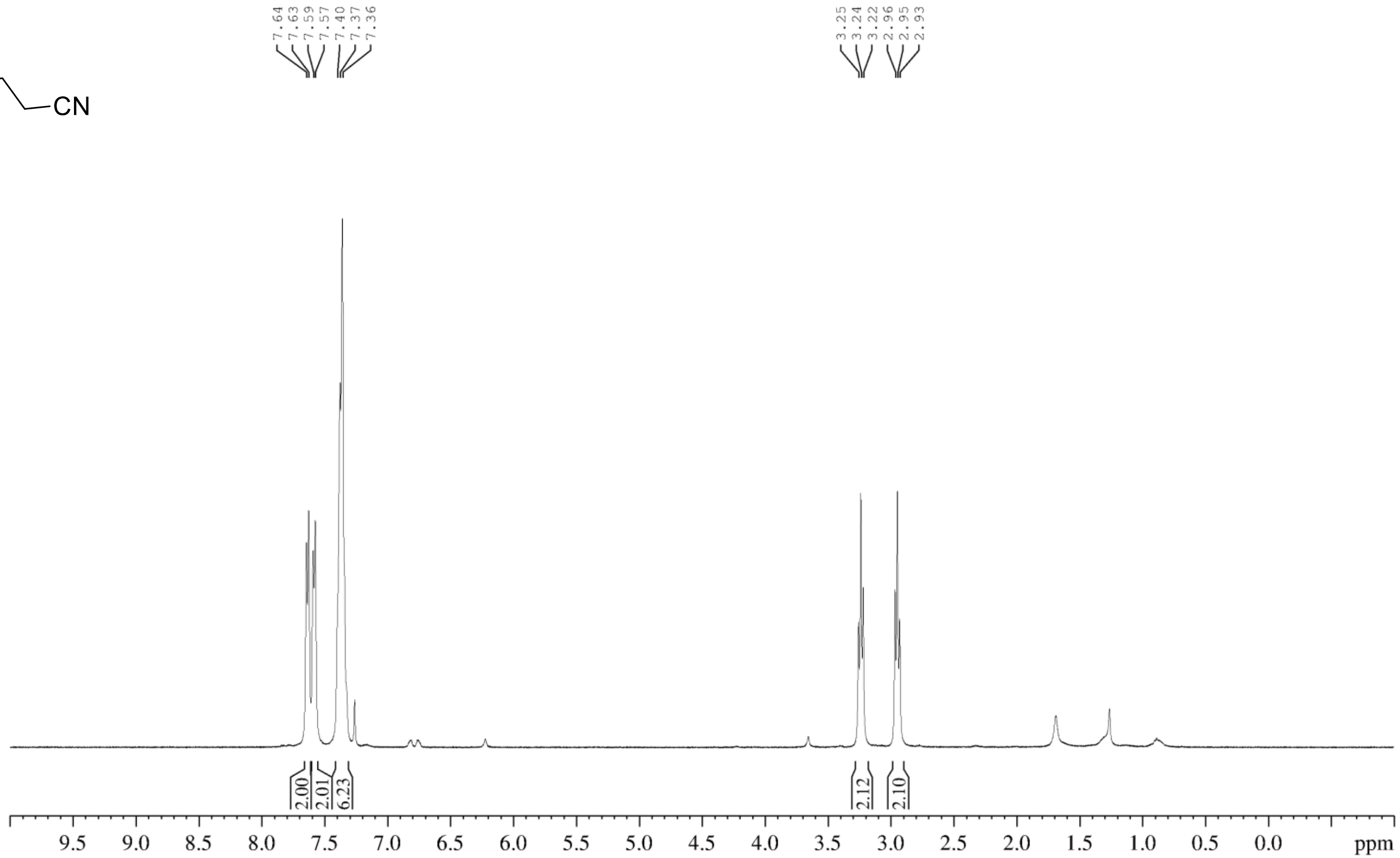

Figure S148. ^{13}C NMR (126 MHz, $CDCl_3$) of **6b**.

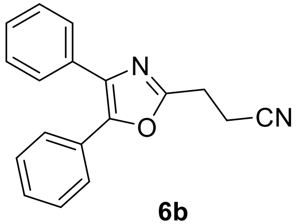

6b

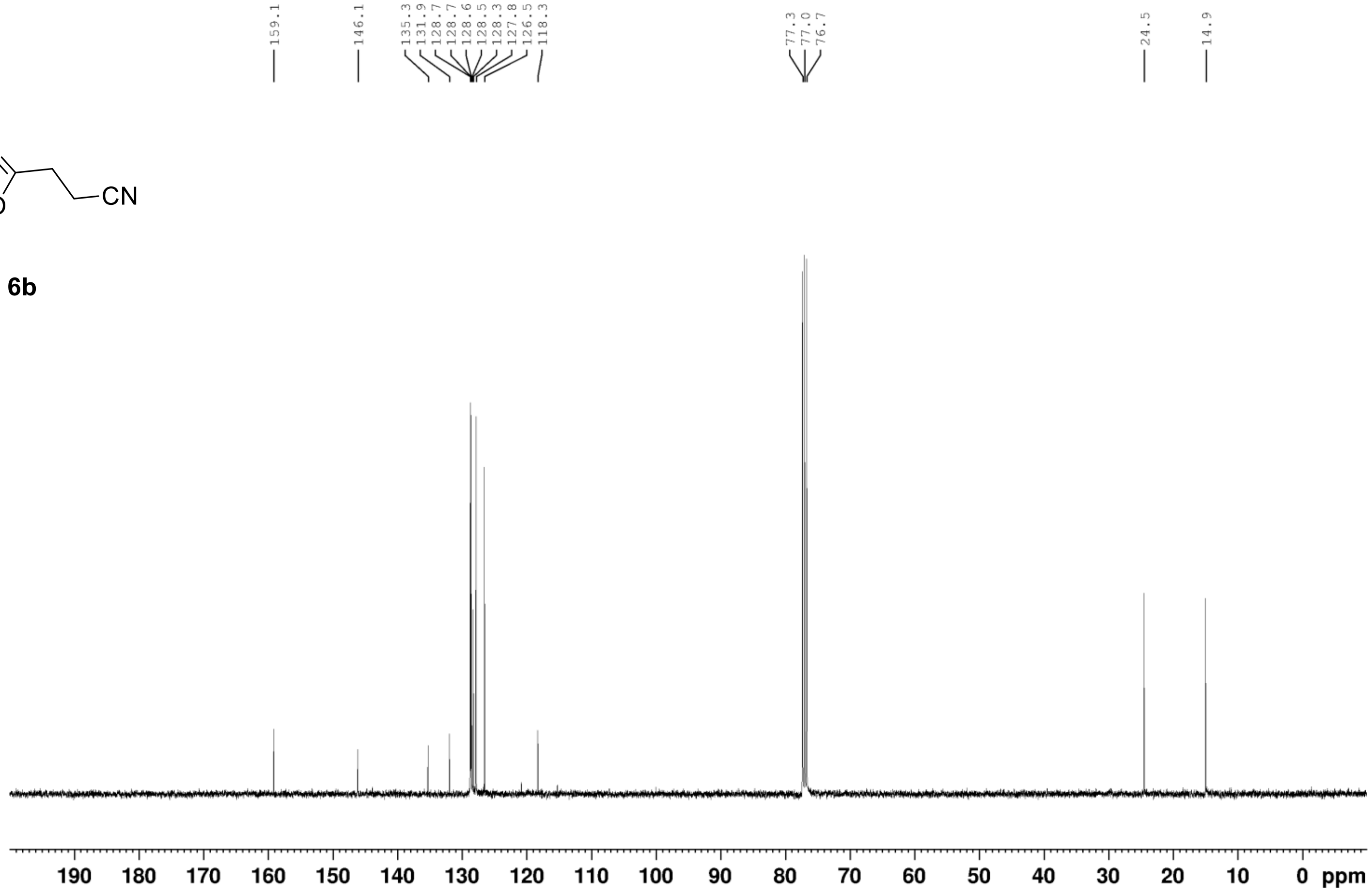

Figure S149. **^{1}H NMR** (500 MHz, $CDCl_3$) of **6c**.

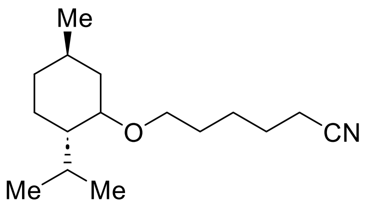

6c

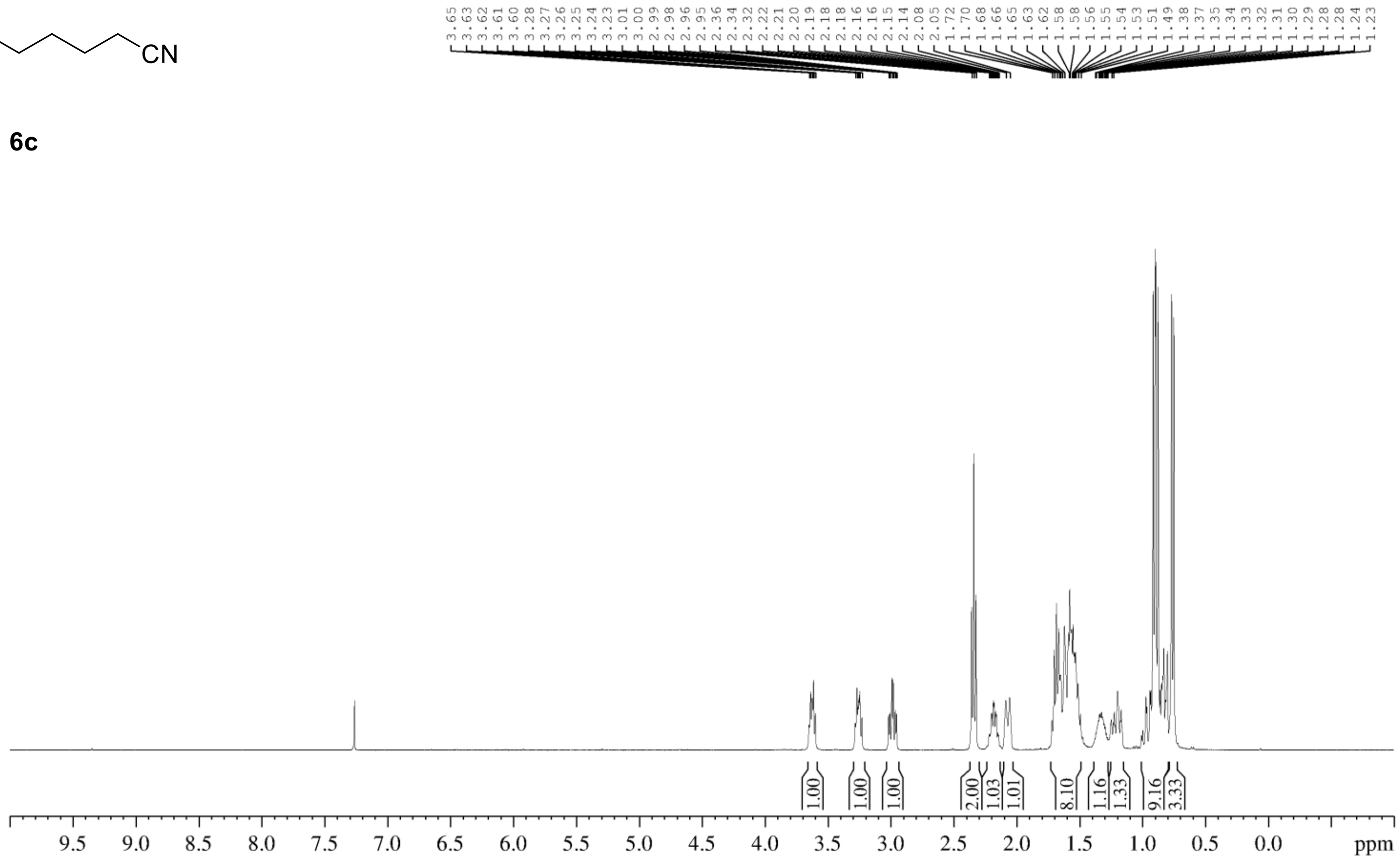

Figure S150. ^{13}**C NMR** (126 MHz, $CDCl_3$) of **6c**.

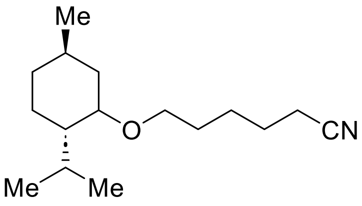

6c

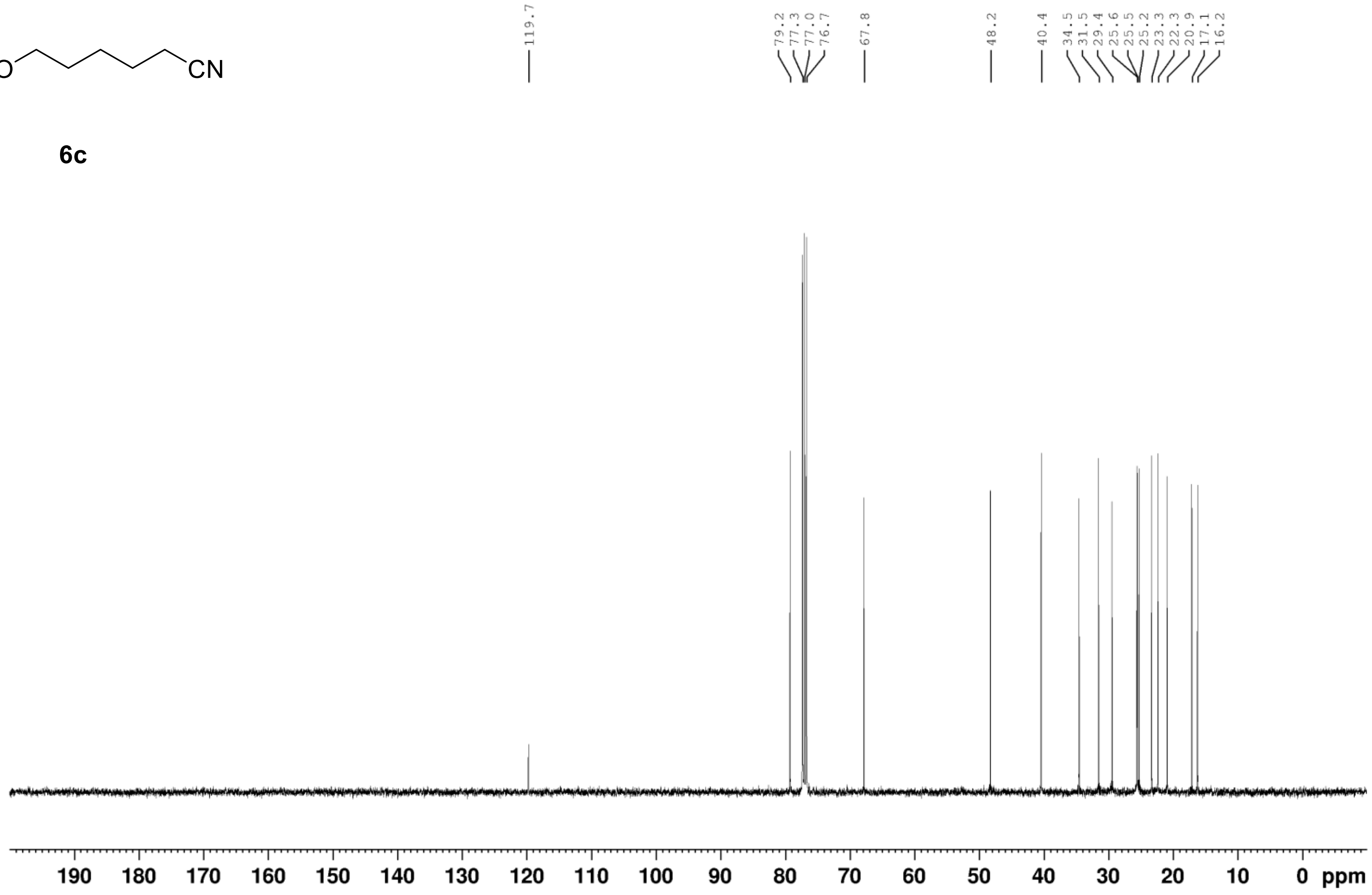

Figure S151. **^{1}H NMR** (500 MHz, $CDCl_3$) of **6d**.

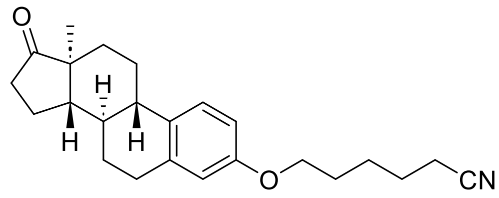

6d

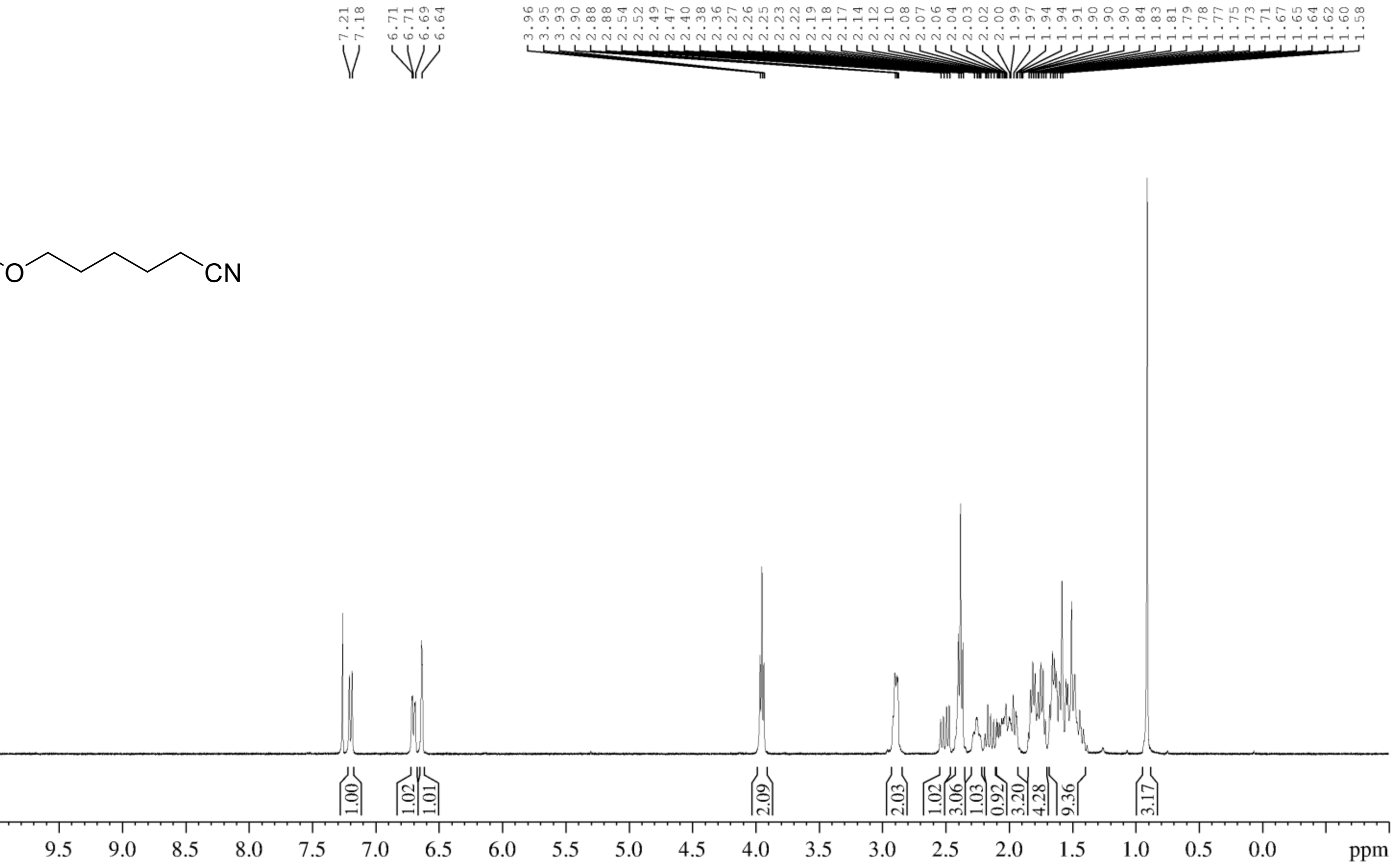

Figure S152. ^{13}**C NMR** (126 MHz, $CDCl_3$) of **6d**.

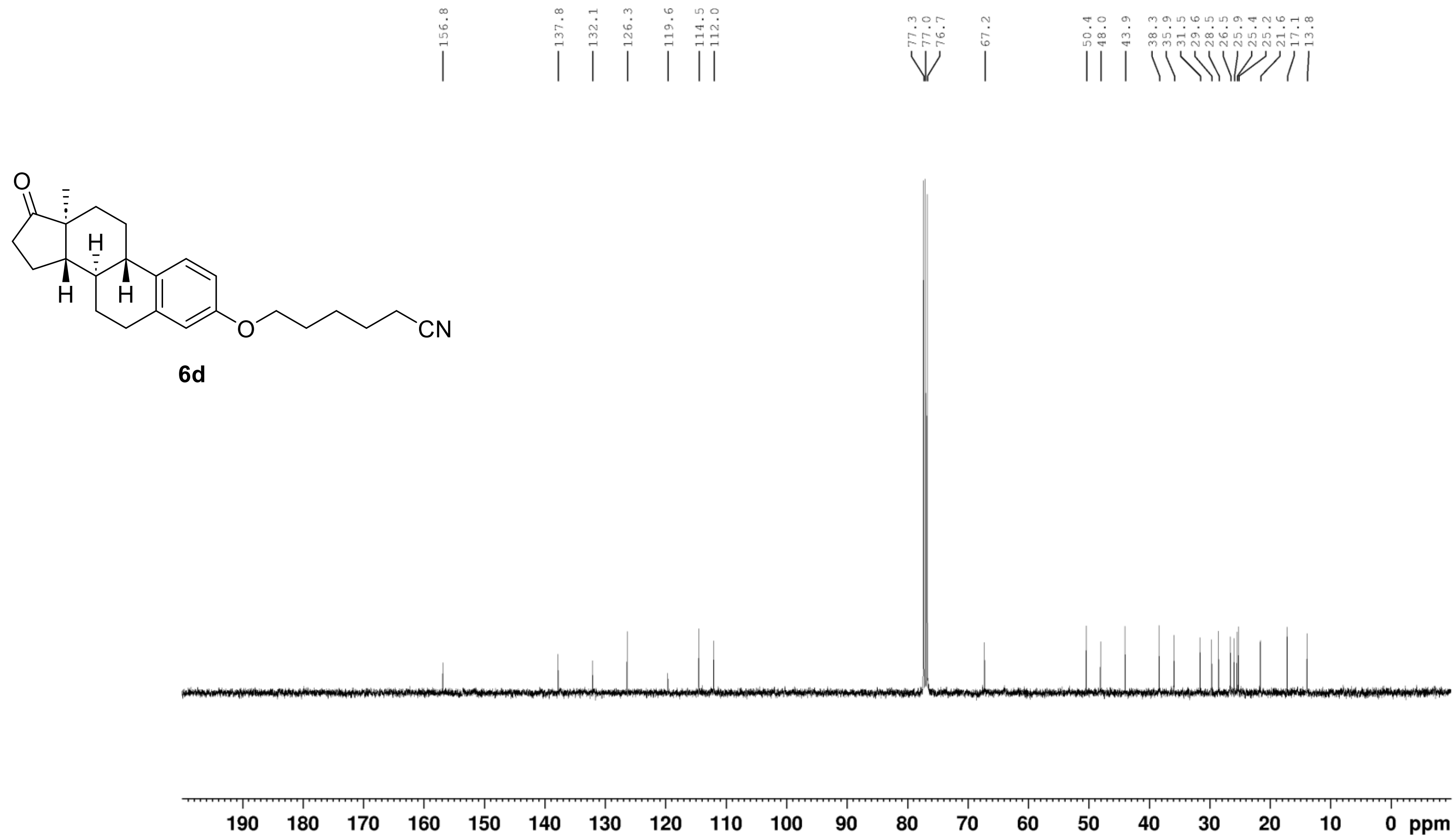

Figure S153. **^{1}H NMR** (500 MHz, $CDCl_3$) of **8a**.

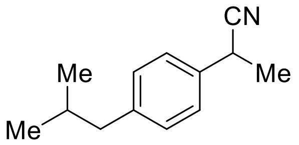

8a

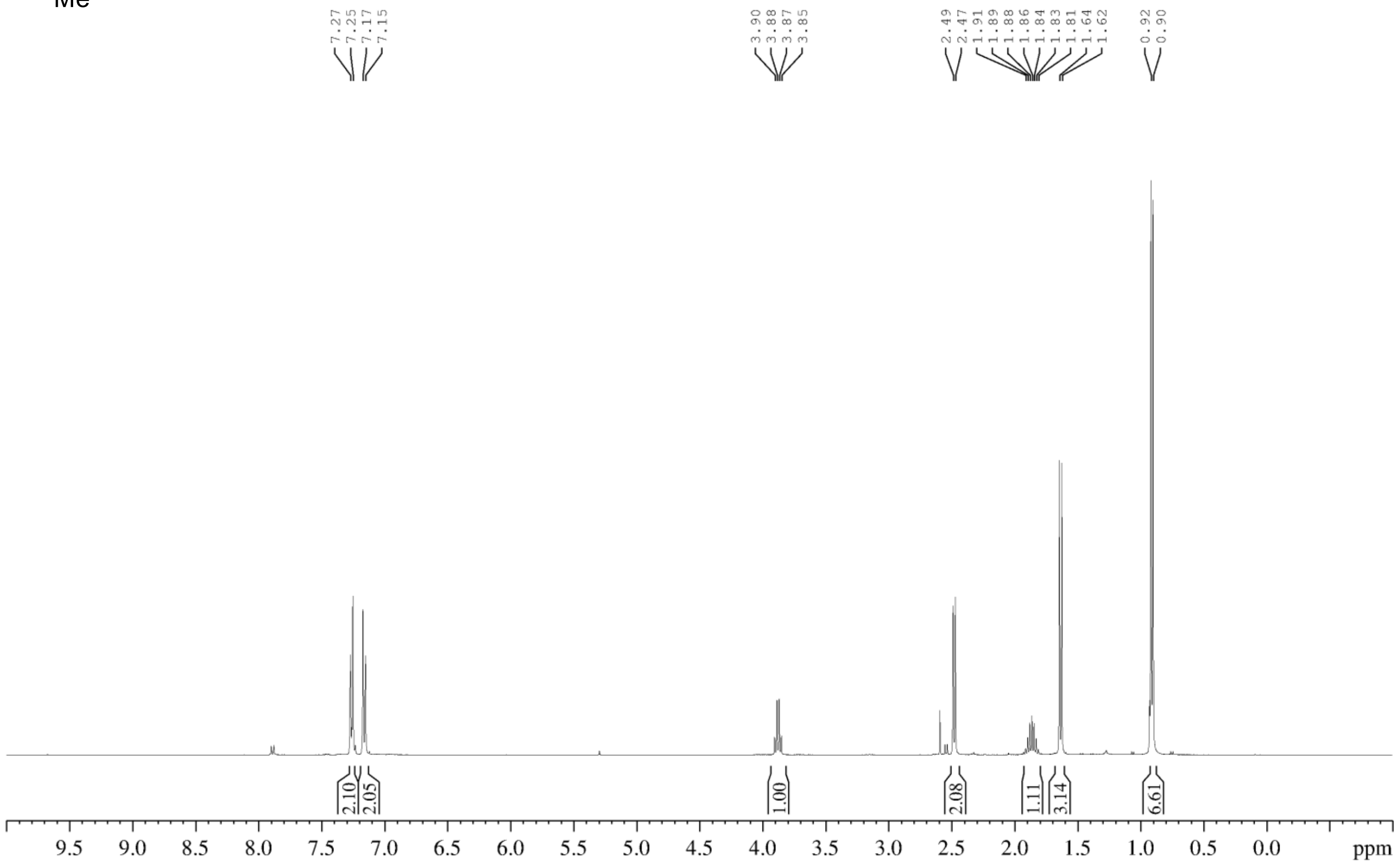

Figure S154. ^{13}**C NMR** (126 MHz, $CDCl_3$) of **8a**.

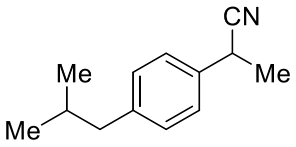

8a

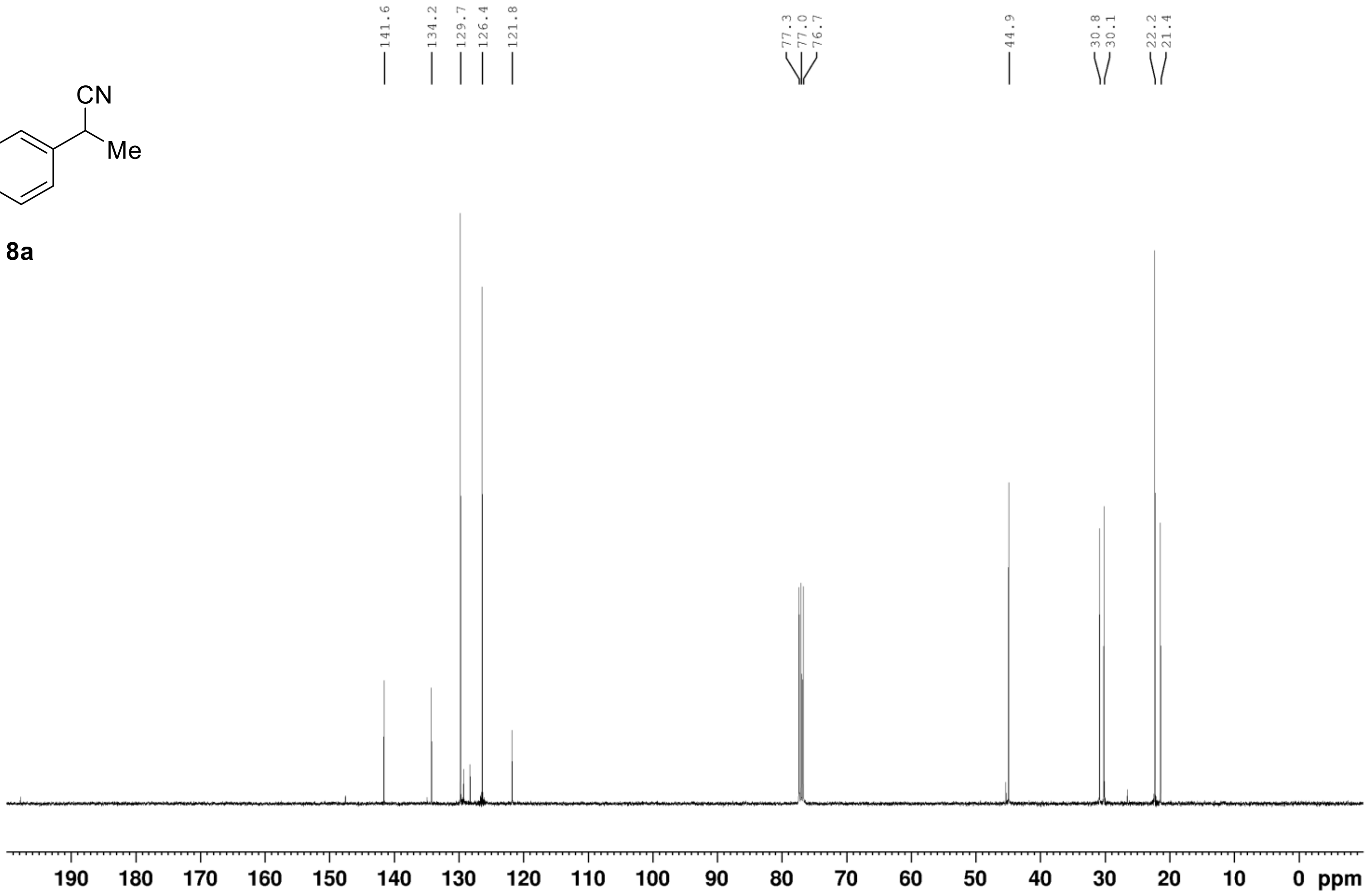

Figure S155. **^{1}H NMR** (500 MHz, $CDCl_3$) of **8b**.

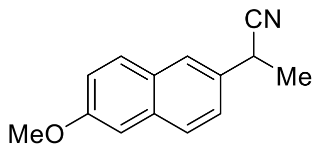

8b

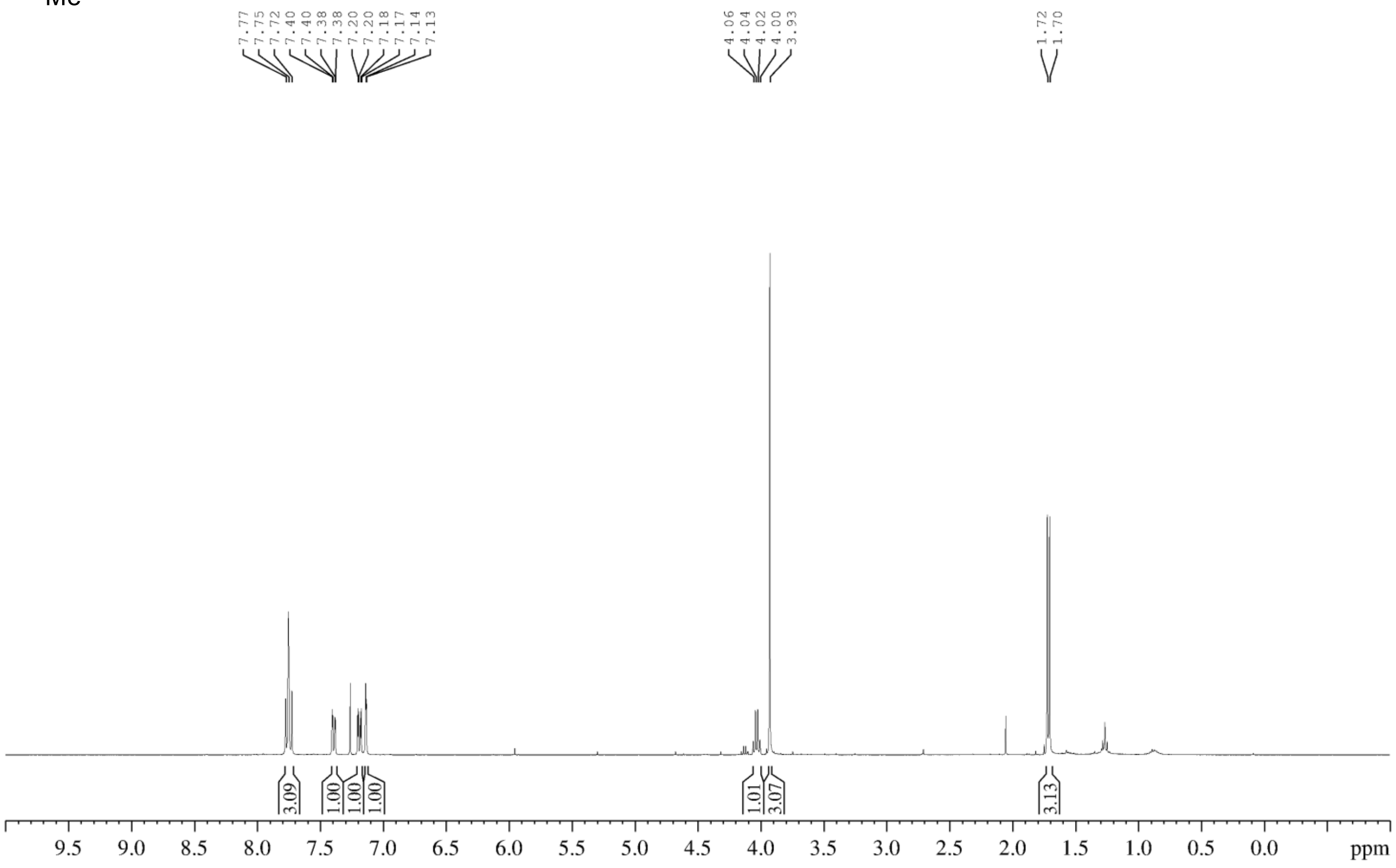

Figure S156. ^{13}**C NMR** (126 MHz, $CDCl_3$) of **8b**.

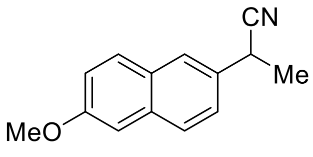

8b

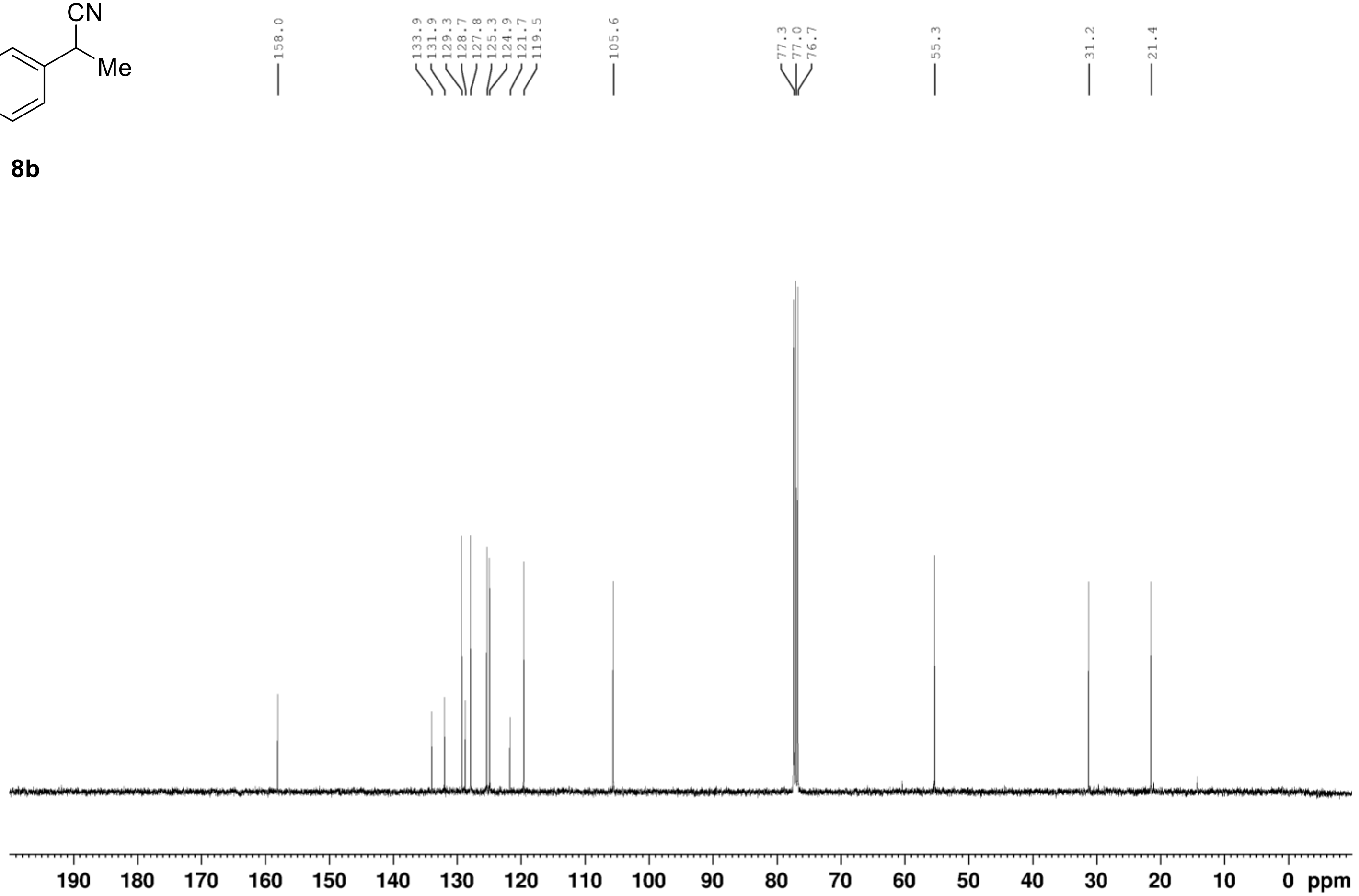

Figure S157. **^{1}H NMR** (500 MHz, $CDCl_3$) of **8c**.

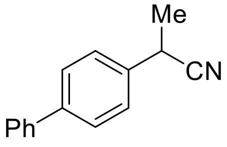

8c

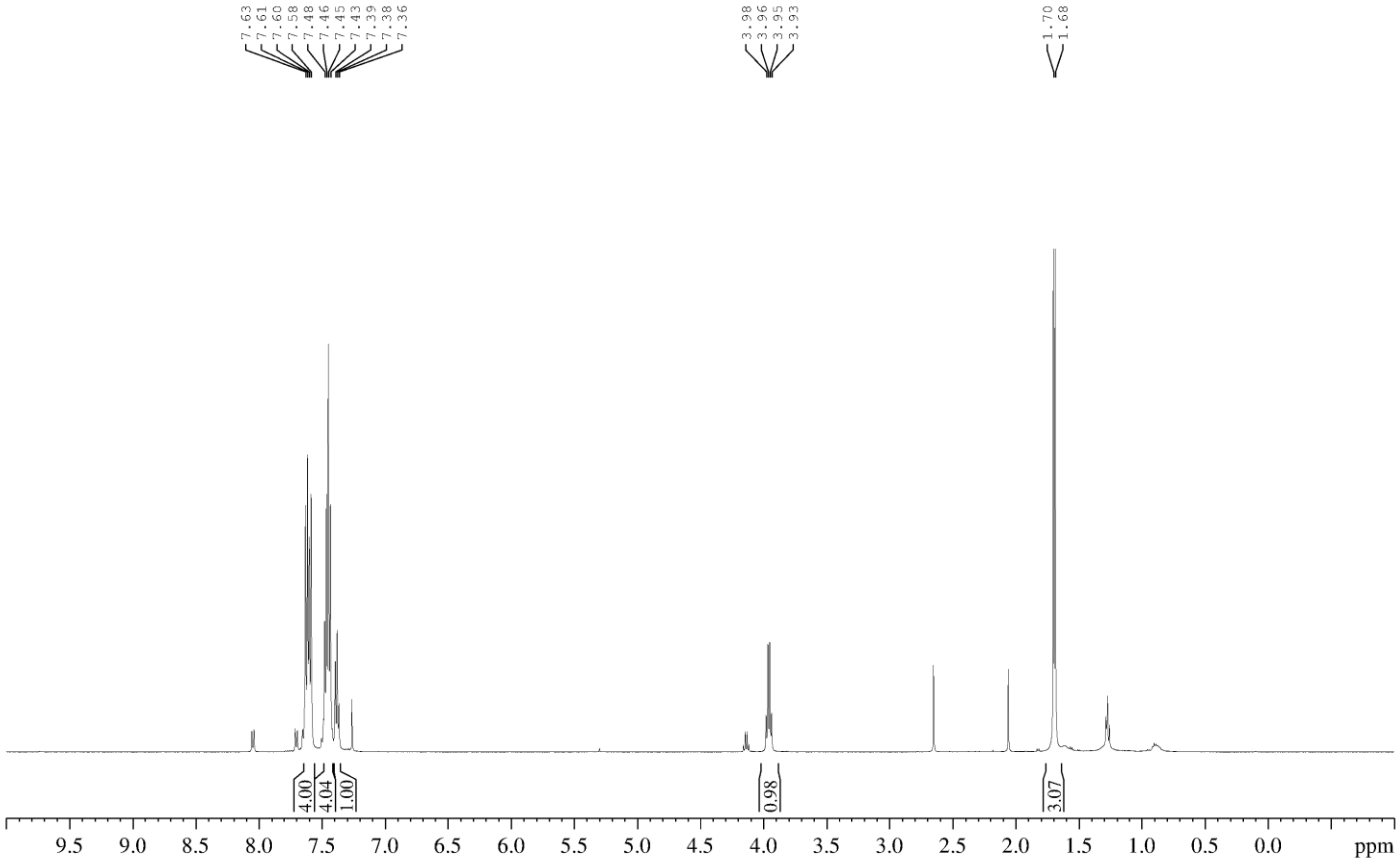

Figure S158. ^{13}**C NMR** (126 MHz, $CDCl_3$) of **8c**.

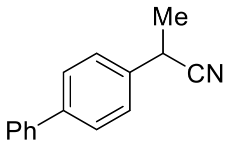

8c

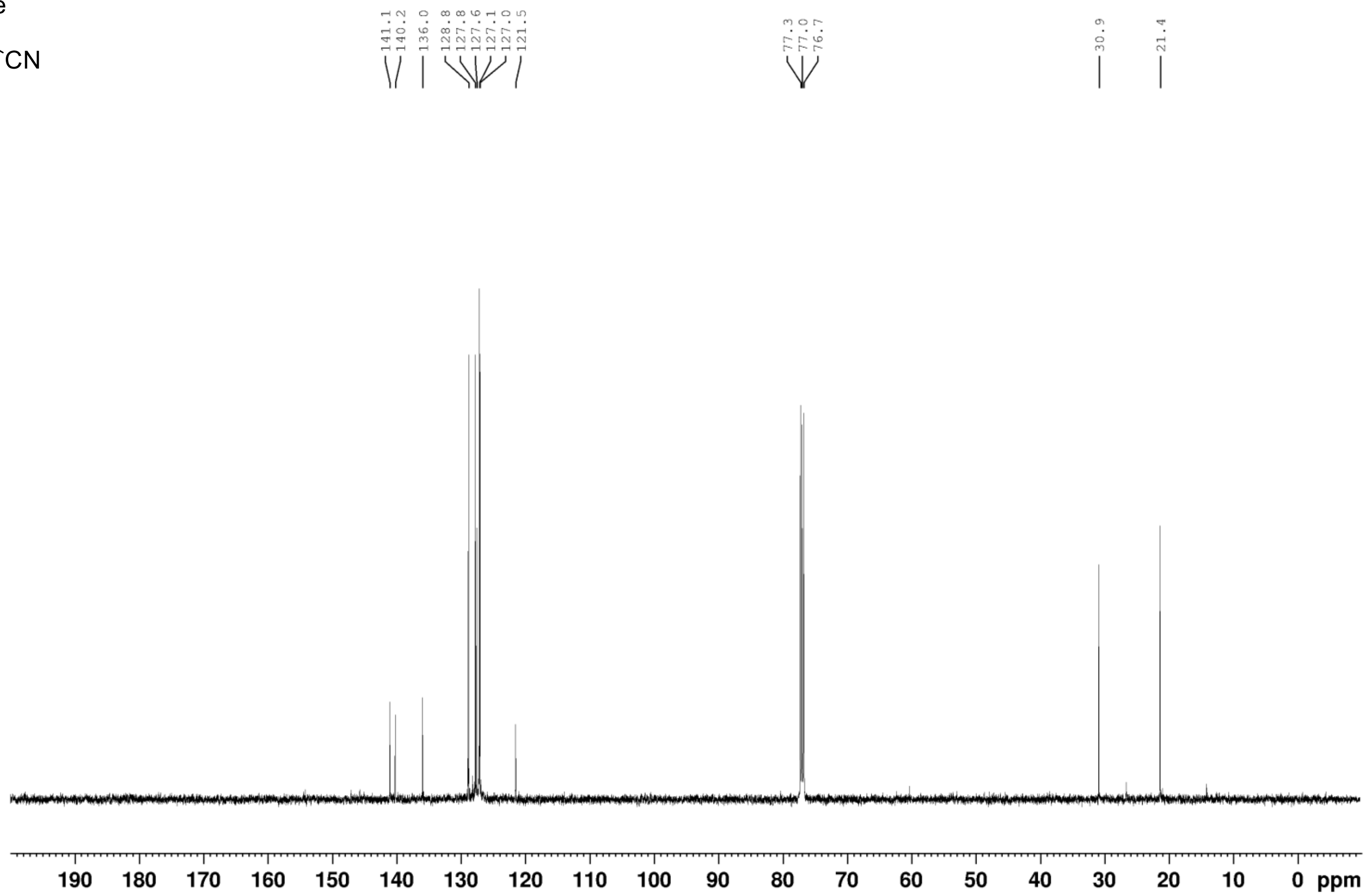

Figure S159. ^{1}**H NMR** (500 MHz, $CDCl_3$) of **10**.

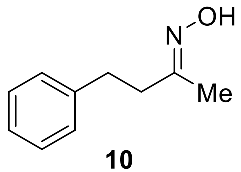

10

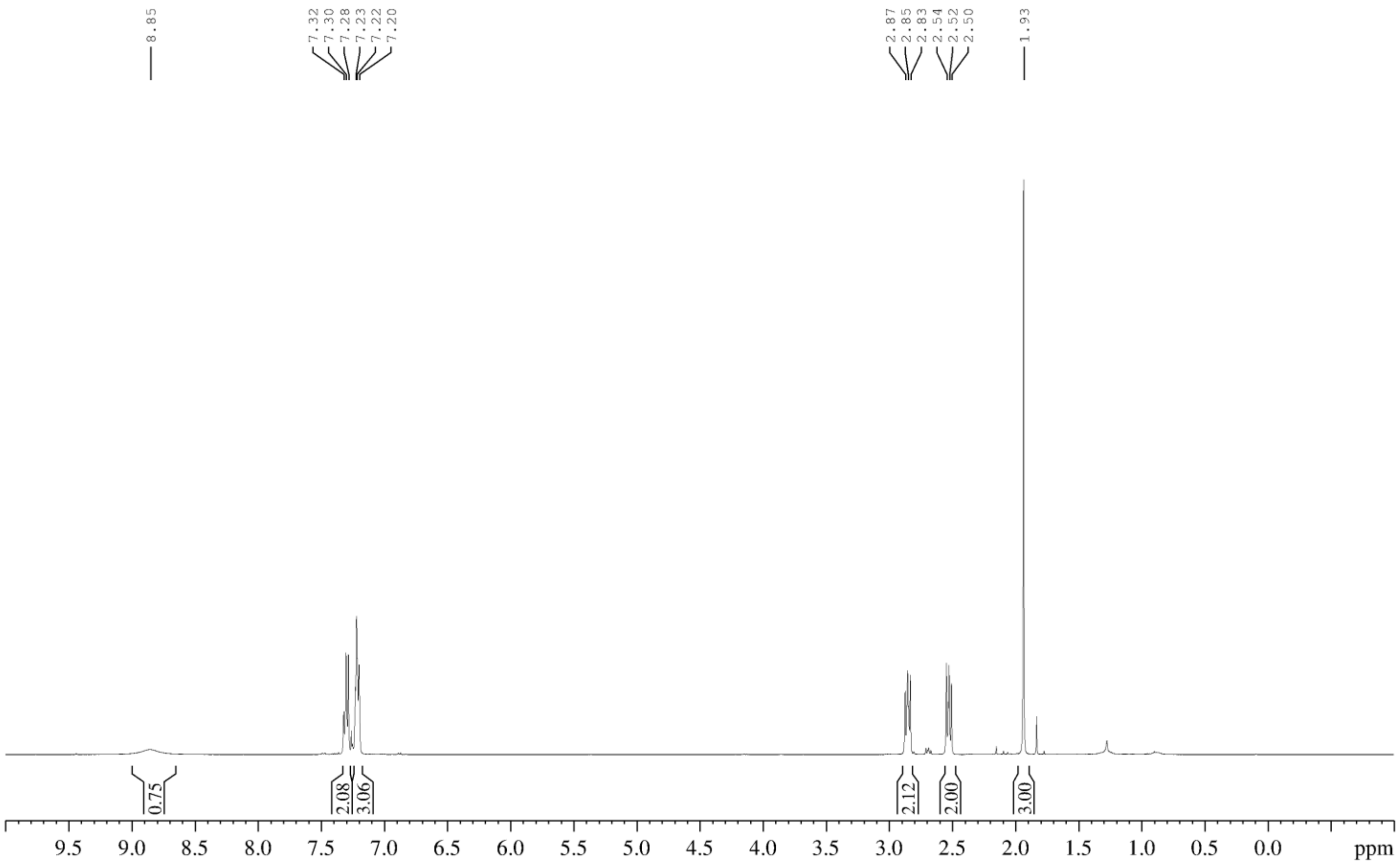

Figure S160. **^{13}C NMR** (126 MHz, $CDCl_3$) of **10**.

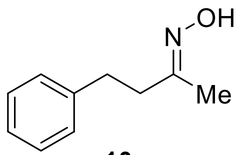

10

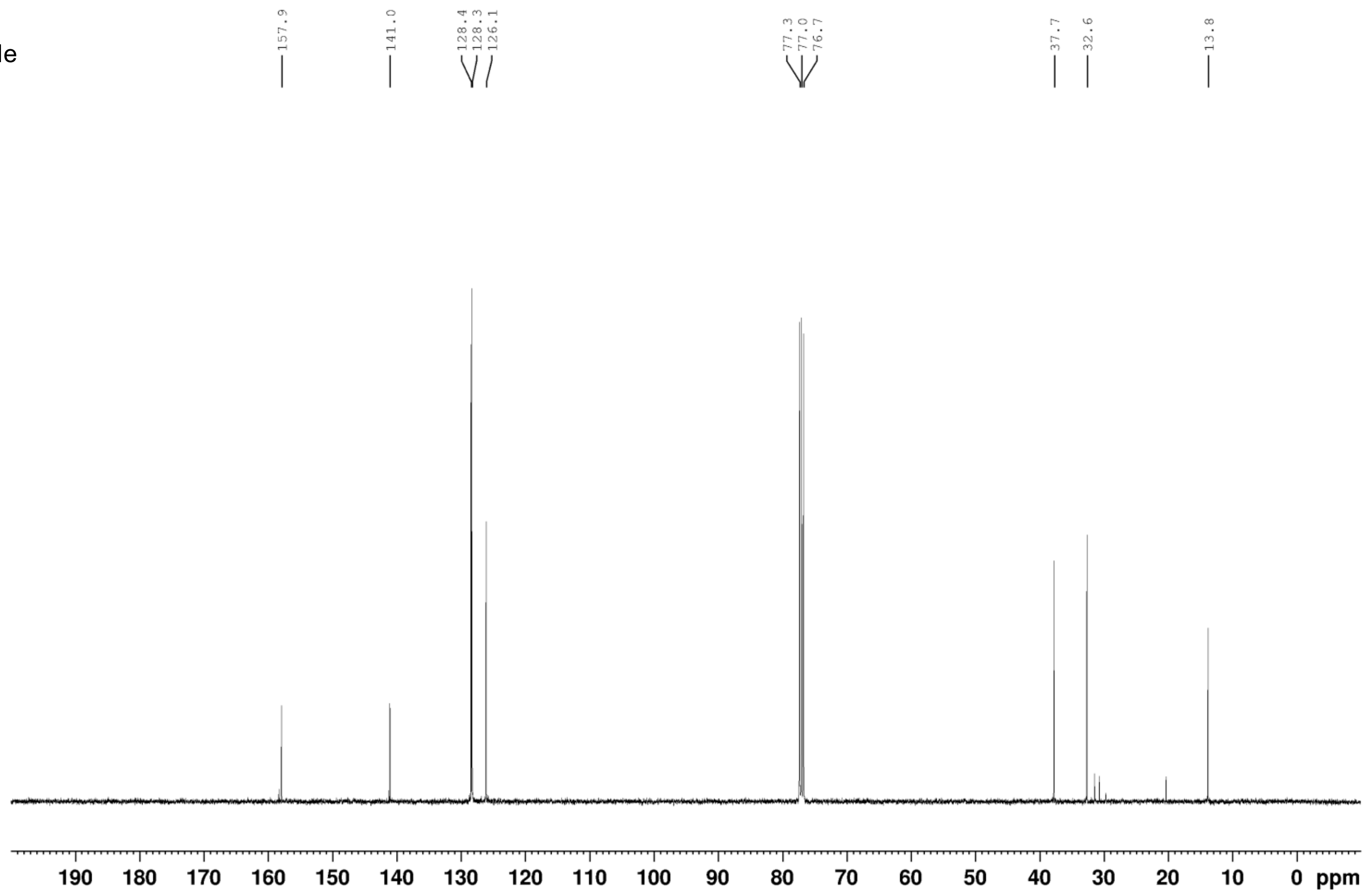

7 References

[S1] P. Reddy and R. Bandichhor, *Tetrahedron Lett.*, 2013, **54**, 3911–3915.

[S2] N. A. White, D. A. DiRocco and T. Rovis, *J. Am. Chem. Soc.*, 2013, **135**, 8504–8507.

[S3] A. Alexakis and C. Benhaim, *Org. Lett.*, 2000, **2**, 2579−2581.

[S4] S. G. Agalave, M. B. Chaudhari, G. S. Bisht and B. Gnanaprakasam, *ACS Sustainable Chem. Eng.* 2018, **6**, 12845−12854.
